# Annotated checklist for stony corals of American Sāmoa with reference to mesophotic depth records

**DOI:** 10.3897/zookeys.849.34763

**Published:** 2019-05-21

**Authors:** Anthony D. Montgomery, Douglas Fenner, Robert J. Toonen

**Affiliations:** 1 Hawaiʻi Institute of Marine Biology, University of Hawaiʻi at Mānoa, Kāneʻohe, HI 96744, USA University of Hawaiʻi at Mānoa Kāneʻohe United States of America; 2 U.S. Fish and Wildlife Service, Pacific Islands Fish and Wildlife Office, 300 Ala Moana Blvd. Honolulu, HI 96850, USA U.S. Fish and Wildlife Service Honolulu United States of America; 3 Ocean Associates, Inc., NOAA Fisheries Service, Pacific Islands Regional Office, Pago Pago, AS, USA NOAA Fisheries Service, Pacific Islands Regional Office Pago Pago American Samoa

**Keywords:** Helioporidae, mesophotic coral ecosystems, Milleporidae, new records, Scleractinia, Stylasteridae, WoRMS, World List of Scleractinia

## Abstract

An annotated checklist of the stony corals (Scleractinia, Milleporidae, Stylasteridae, and Helioporidae) of American Sāmoa is presented. A total of 377 valid species has been reported from American Sāmoa with 342 species considered either present (251) or possibly present (91). Of these 342 species, 66 have a recorded geographical range extension and 90 have been reported from mesophotic depths (30–150 m). Additionally, four new species records (*Acanthastreasubechinata* Veron, 2000, *Favitesparaflexuosus* Veron, 2000, *Echinophylliaechinoporoides* Veron & Pichon, 1980, *Turbinariairregularis* Bernard, 1896) are presented. Coral species of concern include species listed under the US Endangered Species Act (ESA) and the International Union for Conservation of Nature’s (IUCN) Red List of threatened species. Approximately 17.5% of the species present or possibly present are categorized as threatened by IUCN compared to 27% of the species globally. American Sāmoa has seven ESA-listed or ESA candidate species, including *Acroporaglobiceps* (Dana, 1846), *Acroporajacquelineae* Wallace, 1994, *Acroporaretusa* (Dana, 1846), *Acroporaspeciosa* (Quelch, 1886), *Fimbriaphylliaparadivisa* (Veron, 1990), *Isoporacrateriformis* (Gardiner, 1898), and *Pocilloporameandrina* Dana, 1846. There are two additional species possibly present, i.e., *Pavonadiffluens* (Lamarck, 1816) and *Poritesnapopora* Veron, 2000.

## Introduction

**Figure 1.**
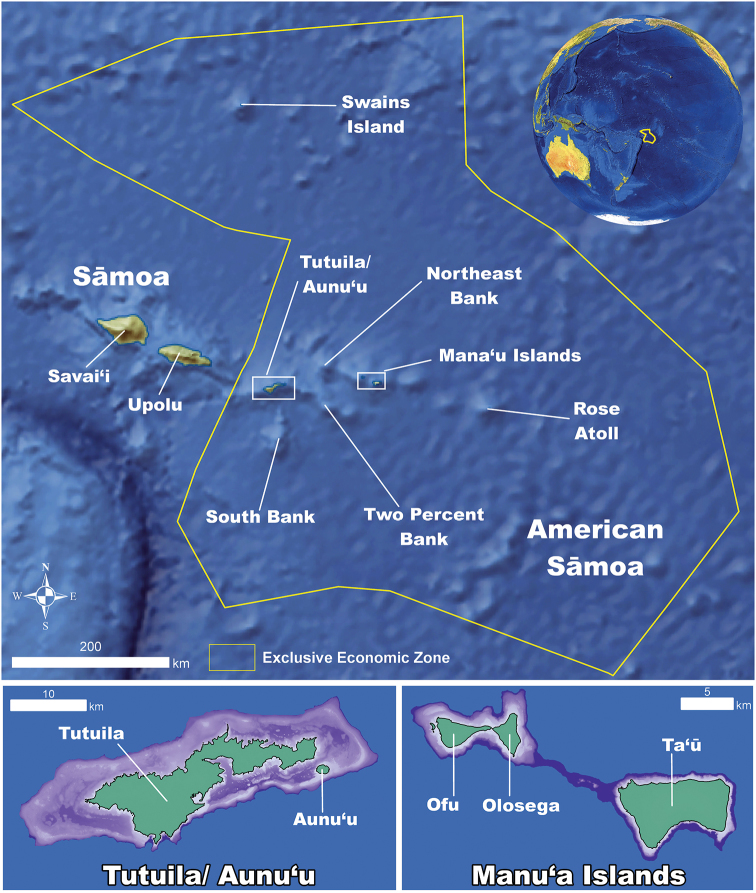
Map of American Sāmoa. A map of American Sāmoa showing its proximity to Independent Sāmoa and the distances between all the island groups (in green) and shallow (< 150 meter depth) banks (in purple) within the territory.

American Sāmoa is an unincorporated territory of the United States and lies between Hawaiʻi and New Zealand in the Southern Pacific Ocean (Figure 1). The Samoan Archipelago includes American Sāmoa, which consists of five high islands (Tutuila, Aunuʻu, Ofu, Olosega, and Taʻū), one low island (Swains Island), and an atoll (Rose), and the Independent state of Sāmoa at the west end of the archipelago with the two high islands (Upola and Savaiʻi) and eight smaller islands. Tonga lies approximately 900 km to the southwest and Tuvalu lies approximately 1,400 km to the northwest.

Coral reef research has been conducted in American Sāmoa since the early 1900s with work analyzing the growth rate of coral reefs and reporting the World’s second stony coral transect (Mayor 1924a, 1924b; Mayor 1918). Since then, there has been a series of studies around the territory documenting its coral communities (Hoffmeister 1925; Birkeland et al. 1987, 2003, 2013; Maragos et al. 1994) including coral species checklists (Lamberts 1983; Birkeland 2001, 2007a, 2007b; Coles et al. 2003; DiDonato et al. 2006; Lovell and McLardy 2008; Kenyon et al. 2011). While there have been several papers looking at the coral species of American Sāmoa, most are not peer-reviewed and none to date have considered the mesophotic zone explicitly. This has led to a large amount of documentation on the coral diversity across the territory, but not in a comprehensive manner that analyzes all available data and the scale of evidence for a complete coral species presence.

Previously referred to as deep coral reefs or the coral reef twilight zone (Pyle 1996, 1998, 2000), mesophotic coral ecosystems (MCE) are well defined in the literature: “Mesophotic coral ecosystems (MCEs) are characterized by the presence of light-dependent corals and associated communities that are typically found at depths ranging from 30 to 40 m and extending to over 150 m in tropical and subtropical regions. The dominant communities providing structural habitat in the mesophotic zone can be comprised of coral, sponge, and algal species” (Hinderstein et al. 2010). Previously thought to be marginal habitats, MCEs have been hypothesized as potential refugia for shallow water corals under the ‘deep reef refugia’ hypothesis (DRRH) (Glynn 1996; Hughes and Tanner 2000; Riegl and Piller 2003; Bak et al. 2005; Kahng et al. 2010; Bongaerts et al. 2010; Tenggardjaja et al. 2014; Holstein et al. 2015). Others have argued that MCEs host different communities of species, and make unlikely refugia from a warming ocean (Hurley et al. 2016; Smith et al. 2016; Bongaerts et al. 2017; Semmler et al. 2017). Bongaerts et al. (2010) reviewed the current literature regarding the DRRH for Caribbean reefs and concluded that the DRRH is more likely to apply to “depth generalist” species and may serve a greater importance in the upper range of MCEs (30–60 m). This was later exemplified on a Pacific reef in Okinawa, where the coral *Seriatoporahystrix* Dana, 1846 was extirpated from shallow water, and later discovered in an upper MCE at depths of 35 to 47 m (Sinniger et al. 2013). Here, we provide a notation for each species reported to be within the mesophotic zone in order to provide a common baseline on species occurrence.

The biogeography of corals has only been studied in the last few decades (Stehli and Wells 1971) with species level comparisons in the last two decades. Corals of the World (CoTW; Veron et al. (2019) recognizes 833 valid zooxanthellate scleractinian species globally and does not include the azooxanthellate dendrophylliid corals (Cairns 2001; Arrigoni et al. 2014) as well as the cave dwelling *Leptoseristroglodyta* Hoeksema, 2012 (Hoeksema 2012a). The World List of Scleractinia (WLS) reports 1,610 valid species with approximately half of those being zooxanthellate, hermatypic species (Hoeksema and Cairns 2019). Broad-scale biogeographic studies require regional-scale data that can be traced back to a consistent taxonomy (Veron 1995). The most comprehensive biogeographic analysis of scleractinians has been completed by Veron et al. (2015) and CoTW (Veron 2000; Veron et al. 2019). Together, these studies show a pattern of highest diversity in the Coral Triangle with decreasing diversity towards the north, east, and south (Hoeksema 2007; Veron et al. 2015). The ecoregion described by Veron et al. (2019) that includes American Sāmoa is the Sāmoa, Tuvalu, and Tonga ecoregion and includes the island groups of Tuvalu, Tokelau, Wallis and Futuna, Tonga, Niuē, and the Sāmoa Archipelago. Veron et al. (2019) reports this ecoregion has 313 reef coral species, while the neighboring ecoregions range from 16 to over 500 coral reef species and the Coral Triangle with 627 coral reef species (Table 1; Veron et al. 2019).

**Table 1.** Zooxanthellate, reef-dwelling scleractinian species richness for the ecoregions surrounding American Sāmoa based on Veron et al. (2019).

Ecoregion	Number of species
Coral Triangle	627
Bismarck Sea, New Guinea	538
Milne Bay, Papua New Guinea	523
Solomon Islands and Bougainville	516
New Caledonia	439
Fiji	395
Caroline Islands, Micronesia	395
Vanuatu	391
Pohnpei and Kosrae, Micronesia	384
Coral Sea	378
Kiribati west, Gilbert Islands	316
Sāmoa, Tuvalu, Tonga	313
Marshall Islands	309
Great Barrier Reef south	308
Kiribati, north-east Line Islands	194
Cook Islands, central Pacific	181
Kiribati central, Phoenix Islands	178
Society Islands, French Polynesia	176
Austral Islands, French Polynesia	153
Tuamotu Archipelago west, central Pacific	117
Kiribati, south-east Line Islands	112
Tuamotu Archipelago south-east and Pitcairn Islands	104
Hawaii east	58
Johnston Atoll, north central Pacific	37
Marquesas Islands, French Polynesia	23
Kermadec Islands, south Pacific	16

In order to make available the vast history of work completed in American Sāmoa, we present a detailed annotated analysis for the reported shallow and mesophotic stony coral species including scleractinian, milleporid, stylasterid, and helioporid species. This analysis presents the information in an open, transparent manner that allows the reader to judge any particular observation over and beyond our analysis. The goal of this study is to provide a foundation for a thorough species list for the Territory of American Sāmoa with a mechanism that allows the reader to trace back to the original recording of the species. This mechanism will allow different interpretations of the taxonomy, confidence of a species observation, or future analyses of species presence to be re-analyzed or questioned easily. Further, we believe this type of approach to a species checklist on a small regional scale can provide a valuable contribution to broader scale biogeographic analyses as discussed by Veron (1995).

## Materials and methods

Species occurrences in the study area were recorded from all available literature (Mayor 1924a, 1924b; Hoffmeister 1925; Dahl and Lamberts 1977; USACE 1980; Dahl 1981; Lamberts 1983; Birkeland et al. 1987, 2003, 2013; Itano and Buckley 1988; Hoeksema 1989; Hunter et al. 1993; Maragos et al. 1994, 1995; Mundy 1996; Green et al. 1997; Green and Hunter 1998; Green et al. 1999; Mundy and Green 1999; Birkeland and Belliveau 2000; Craig et al. 2001; Fisk and Birkeland 2002; Work and Rameyer 2002; Coles et al. 2003; Wolstenholme et al. 2003; Cornish and DiDonato 2004; DiDonato et al. 2006; Birkeland 2007a, 2007b; Fenner et al. 2008; Lovell and McLardy 2008; Forsman and Birkeland 2009; Forsman et al. 2009; Bare et al. 2010; Benzoni et al. 2010; Kenyon et al. 2010, 2011; CRED 2011; Corals NPAS 2016; Fenner and Sudek 2016; AM 2018; BPBM 2018; DMWR 2018; Fenner 2018; QM 2018; NMNH 2018; Creuwels 2019; Gross 2019; Montgomery et al. 2019; Paulay and Brown 2019). Each appearance of a species name was recorded with as much information as available including the exact spelling of the species name, identification qualifiers (e.g., cf., aff., ?, etc.), survey type, island, species location (e.g., site name, transect label, etc.), the reference of the appearance, if the species was referenced to a different source and its reference, and any other general notes or information. This information was collected in a Microsoft Excel spreadsheet and imported into R where the data were validated. All unique species names were cross-referenced with valid accepted names in the World Register of Marine Species (WoRMS) through their REST webservice (http://www.marinespecies.org/rest/). Names were queried for exact matches and missing matches were queried for fuzzy matches based on the Taxamatch algorithm (Rees 2014). Remaining names were queried again with older generic names (e.g., *Madrepora* instead of *Acropora*). All remaining names were retained as used.

Additional data validation steps included the elimination of duplicate returns from WoRMS, examining each individual fuzzy match return and dubious names, and standardizing nomenclature for unaccepted name explanations. The classification was updated based on the accepted valid name of a species and added to all records from the WoRMS database. Finally, any missing names that were unable to be matched with WoRMS records were added individually.

The checklist is arranged by species presence determination and then by order and alphabetically by family, genus and species for each valid name. Each species record starts with listing the valid species name as accepted in WoRMS (WoRMS Editorial Board 2018). The valid name is hyperlinked to the WoRMS species taxa webpage followed by the species authority and AphiaID. Under each valid name, species names as appeared in the literature are reported followed by the species authority and AphiaID. Species names that have grammatical misspellings are labeled with [sic]. If the species was a synonym, then it was labeled as a heterotypic or homotypic synonym. We use the term ‘homotypic synonym’ to refer to cases where the same species epithet is combined with different genera, and ‘heterotypic synonym’ in cases where different species epithets are regarded as subjective junior synonyms. While the terms ‘objective synonym’ and ‘subjective synonym’ are technically defined within the International Code of Zoological Nomenclature (ICZN) Code to mean essentially the same (http://www.nhm.ac.uk/hosted-sites/iczn/code/index.jsp?booksection=glossary&nfv=true), in our experience the term ‘objective synonym’ is most commonly used in cases of different species epithets that share the same type specimen (e.g., replacement names). Therefore, we believe the terms ‘homotypic synonym’ and ‘heterotypic synonym’ are more appropriate in the present context, which is consistent with how these terms are most commonly used for taxonomic purposes. Each species name is followed by the references that used that exact name and spelling. The references are separated into first-hand accounts labeled as reported and second or more hand accounts labeled as referenced.

All names (valid and synonymies) according to the World List of Scleractinia (Hoeksema and Cairns 2019) were cross-referenced with the accepted names by Veron et al. (2019), Wallace (1999), and Wallace et al. (2012) to provide the reader with a potentially different view of the species and highlight any differences. Any name that matched a name accepted by Wallace (1999) or Wallace et al. (2012) was noted by CCW. Any name that matched a name accepted by Veron et al. (2019) was notated as CoTW. The notation also provides a hyperlink to the factsheet available on the CoTW webpage (http://www.coralsoftheworld.org/page/home/). Veron et al. (2019) is an electronic source that has evolved from the printed worldwide overview of reef-dwelling Scleractinia by Veron (2000), while Hoeksema and Cairns (2019) is based on published taxonomic revisions of various scleractinian families and genera, partly based on molecular analyses and/or on the re-examination of type specimens and other museum material (Wallace 1999; Wallace et al. 2007, 2012; Hoeksema 2009, 2012a, 2012b, 2014; Benzoni et al. 2010, 2011, 2012a, 2014; Gittenberger et al. 2011; Huang et al. 2011, 2014a, 2014b, 2016; Budd et al. 2012; Arrigoni et al. 2014, 2015, 2016a, 2016b, 2017, 2018a; Kitano et al. 2014; Schmidt-Roach et al. 2014; Terraneo et al. 2016, 2017).

After all names and references are listed, we include our determination of the species presence in American Sāmoa. This determination was split into five categories: present, possibly present, uncertain, not likely present, and not present. This determination was made largely on the type of evidence available including the amount of references, the type of reference, the evidence the reference includes (e.g., in situ observation, photographic, sample identified, or type specimen), and taxonomic and identification certainty. The species presence determination is followed by information on the highest level of evidence available to support the species presence. Additionally, the annotation includes the reported species distribution within American Sāmoa as reported by the literature, the nearest confirmed ecoregion for the species presence according to Veron et al. (2019), the direction of a potential geographic range extension, the evidence of species vulnerability as documented by the International Union for Conservation of Nature’s (IUCN) Red List of threatened species (IUCN 2018) as assessed by Carpenter et al. (2008) and the US Endangered Species Act (ESA), and the depths and associated references for corals reported from mesophotic depths. Finally, we provide notes that discuss our justification of a species presence determination, other evidence not already listed, or other noteworthy comments. Each IUCN note is hyperlinked to the IUCN Red List species information webpage (http://www.iucnredlist.org/).

Species listed by the National Oceanic and Atmospheric Administration (NOAA) under the ESA are notated with the symbol 𝒯 for threatened and 𝒞 for candidate listing. The species status as listed by the IUCN Red List of Threatened Species is noted as Critically Endangered (CE), Endangered (EN), Vulnerable (VU), Near Threatened (NT), Least Concern (LC), Data Deficient (DD), and Not Evaluated (NE).

### Museum name abbreviations

**AM** Austalian Museum, New South Wales, Australia

**BPBM** Bernice P Bishop Museum, Honolulu, Hawaiʻi

**CRED** Coral Reef Ecosystem Division, NOAA, Honolulu, Hawaiʻi

**DMWR** Department of Marine and Wildlife Resources, Pago Pago, American Sāmoa

**NMNH** US National Museum of Natural History, Smithsonian Institution, Washington, DC

**QM** Queensland Museum, Brisbane, Australia

### Datasets

The data underpinning the analyses reported in this paper are deposited in the GBIF, the Global Biodiversity Information Facility (Citation: Montgomery A, Toonen R, Fenner D (2019) Annotated checklist for Stony Corals of American Samoa and Mesophotic Depth Records. https://doi.org/10.15468/07opwe).

## Checklist

### Present

#### Class Anthozoa Ehrenberg, 1834

##### Subclass Hexacorallia Haeckel, 1896

###### Order Scleractinia Bourne, 1900

####### Family Acroporidae Verrill, 1902

######## Genus *Acropora* Oken, 1815

***Acroporaabrotanoides* (Lamarck, 1816) (207083)** ^CoTW CCW^

*Acroporaabratanoides* (Lamarck, 1816) (207083) [sic]. Reported – Lamberts 1983.

*Acroporaabrotanioides* (Lamarck, 1816) (207083) [sic]. Reported – DMWR 2018.

*Acroporaabrotanoides* (Lamarck, 1816) (207083). ^CoTW CCW^ Reported – USACE 1980; Birkeland et al. 1987; Fisk and Birkeland 2002; Coles et al. 2003; DiDonato et al. 2006; Birkeland 2007a; Fenner et al. 2008; Kenyon et al. 2010; CRED 2011; Corals NPAS 2016; Fenner 2018; NMNH 2018; QM 2018. Referenced – Coles et al. 2003; DiDonato et al. 2006; Birkeland 2007a, 2007b; Lovell and McLardy 2008.

*Acroporaabrotenoides* (Lamarck, 1816) (207083) [sic]. Reported – Work and Rameyer 2002.

*Acroporadanai* (Milne Edwards, 1860) (206990) heterotypic synonym. Reported – Birkeland et al. 1987; Maragos et al. 1995; Mundy 1996; DiDonato et al. 2006; Corals NPAS 2016. Referenced – Fisk and Birkeland 2002; Coles et al. 2003; DiDonato et al. 2006.

*Acroporairregularis* (Brook, 1892) (206993) heterotypic synonym. Reported – Birkeland et al. 1987, 2003; Itano and Buckley 1988; Maragos et al. 1994; Corals NPAS 2016. Referenced – Green et al. 1999; Coles et al. 2003; DiDonato et al. 2006; Birkeland 2007a, 2007b.

*Acroporarotumana* (Gardiner, 1898) (207001) heterotypic synonym. Reported – Hoffmeister 1925; USACE 1980; Lamberts 1983. Referenced – Dahl and Lamberts 1977; Dahl 1981; Green et al. 1997.

*Acroporatutuilensis* Hoffmeister, 1925 (430656) heterotypic synonym. ^CoTW^ Reported – Mayor 1924b; Hoffmeister 1925; Lamberts 1983; Birkeland et al. 1987; Kenyon et al. 2010; NMNH 2018. Referenced – Green et al. 1999; Birkeland 2007b.

**American Sāmoa status** – Present. **Evidence** – Type specimen location (synonym *Acroporatutuilensis*). **Distribution** – American Sāmoa, Aunuʻu, Manuʻa Islands, Ofu, Ofu/Olosega, Olosega, Rose Atoll, Taʻū, Tutuila. **Nearest confirmed ecoregion** – Sāmoa, Tuvalu, and Tonga. **Vulnerability** – LC. **Notes** – This species has four synonyms with *Acroporatutuilensis* Hoffmeister, 1925 accepted as a valid species by Veron et al. (2019) and as a synonym of *A.abrotanoides* by Wallace (1999) and Wallace et al. (2012) suggesting some ambiguity in species identifications and species boundaries.

***Acroporaaculeus* (Dana, 1846) (206991)** ^CoTW CCW^

*Acroporaaculeus* (Dana, 1846) (206991). ^CoTW CCW^ Reported – USACE 1980; Lamberts 1983; Maragos et al. 1995; Mundy 1996; Craig et al. 2001; Fisk and Birkeland 2002; Birkeland 2007a; Corals NPAS 2016; Fenner 2018; QM 2018; Montgomery et al. 2019. Referenced – Fisk and Birkeland 2002; DiDonato et al. 2006; Birkeland 2007b; Lovell and McLardy 2008; Kenyon et al. 2011.

**American Sāmoa status** – Present. **Evidence** – Multiple specimen reports. **Distribution** – American Sāmoa, Aunuʻu, Ofu, Ofu/Olosega, Tutuila. **Nearest confirmed ecoregion** – Sāmoa, Tuvalu, and Tonga. **Vulnerability** – VU. **Mesophotic record** – 49 m depth (Montgomery et al. 2019).

***Acroporaacuminata* (Verrill, 1864) (207020)** ^CoTW CCW^

*Acroporaacuminata* (Verrill, 1864) (207020). ^CoTW CCW^ Reported – Birkeland et al. 1987, 2003; Maragos et al. 1994; Fisk and Birkeland 2002; Coles et al. 2003; Corals NPAS 2016; QM 2018. Referenced – Green et al. 1999; Coles et al. 2003; DiDonato et al. 2006; Birkeland 2007a, 2007b; Lovell and McLardy 2008; Kenyon et al. 2011.

**American Sāmoa status** – Present. **Evidence** – Single specimen report (identified by C Wallace). **Distribution** – American Sāmoa, Manuʻa Islands, Ofu, Ofu/Olosega, Tutuila. **Nearest confirmed ecoregion** – Sāmoa, Tuvalu, and Tonga. **Vulnerability** – VU. **Notes** – This is not an easy species to identify in the field. Photographic evidence from Corals NPAS (2016) is inconclusive, but a specimen identified by C Wallace is in the QM.

***Acroporaanthocercis* (Brook, 1893) (207024)** ^CoTW CCW^

*Acroporaanthocercis* (Brook, 1893) (207024). ^CoTW CCW^ Reported – Birkeland 2007a; QM 2018.

**American Sāmoa status** – Present. **Evidence** – Single specimen report (identified by J Wolstenholme). **Distribution** – Ofu. **Nearest confirmed ecoregion** – Sāmoa, Tuvalu, and Tonga. **Vulnerability** – VU.

***Acroporaaspera* (Dana, 1846) (207011)** ^CoTW CCW^

*Acroporaaspera* (Dana, 1846) (207011). ^CoTW CCW^ Reported – USACE 1980; Lamberts 1983; Mundy 1996; Birkeland 2007a; Kenyon et al. 2010; Birkeland et al. 2013; BPBM 2018; DMWR 2018; Fenner 2018; NMNH 2018; QM 2018. Referenced – Coles et al. 2003; Birkeland 2007a, 2007b; Lovell and McLardy 2008; Kenyon et al. 2011.

*Acroporacribripora* Dana, 1846 (741197) heterotypic synonym. Reported – Mayor 1924b.

*Acroporahebes* (Dana, 1846) (367984) heterotypic synonym. Reported – Mayor 1924b; Hoffmeister 1925; USACE 1980; Lamberts 1983; Birkeland et al. 1987. Referenced – Dahl and Lamberts 1977; Dahl 1981; Green et al. 1997; Birkeland 2007b.

**American Sāmoa status** – Present. **Evidence** – Multiple specimen reports. **Distribution** – American Sāmoa, Manuʻa Islands, Ofu, Ofu/Olosega, Olosega, Rose Atoll, Taʻū, Tutuila. **Nearest confirmed ecoregion** – Sāmoa, Tuvalu, and Tonga. **Vulnerability** – VU. **Notes** – Randall and Myers (1983) accepted *Acroporahebes* (Dana, 1846) as a valid species while Veron et al. (2019), Wallace (1999), and Wallace et al. (2012) did not.

***Acroporaaustera* (Dana, 1846) (207052)** ^CoTW CCW^

*Acroporaaustera* (Dana, 1846) (207052). ^CoTW CCW^ Reported – Craig et al. 2001; Fisk and Birkeland 2002; Coles et al. 2003; DiDonato et al. 2006; Birkeland 2007a; Fenner et al. 2008; Bare et al. 2010; Corals NPAS 2016; AM 2018; DMWR 2018; Fenner 2018; QM 2018. Referenced – Hoffmeister 1925; DiDonato et al. 2006; Lovell and McLardy 2008.

*Acroporaaustera* cf. (Dana, 1846) (207052). ^CoTW CCW^ Reported – Coles et al. 2003.

**American Sāmoa status** – Present. **Evidence** – Multiple specimen reports. **Distribution** – American Sāmoa, Aunuʻu, Manuʻa Islands, Ofu, Ofu/Olosega, Sāmoa Islands, Taʻū, Tutuila. **Nearest confirmed ecoregion** – Sāmoa, Tuvalu, and Tonga. **Vulnerability** – NT. **Notes** – The photo of this species reported in Corals NPAS (2016) appears to show a specimen of *Acroporapagoensis* Hoffmeister, 1925.

***Acroporabatunai* Wallace, 1997 (288187)** ^CoTW CCW^

*Acroporabatunai* Wallace, 1997 (288187). ^CoTW CCW^ Reported – DMWR 2018.

**American Sāmoa status** – Present. **Evidence** – Single specimen report (identified by D Fenner). **Distribution** – Tutuila. **Nearest confirmed ecoregion** – Solomon Islands and Bougainville. **Geographical range extension** – East. **Vulnerability** – VU.

***Acroporacarduus* (Dana, 1846) (288189)** ^CoTW CCW^

*Acroporacarduus* (Dana, 1846) (288189). ^CoTW CCW^ Reported – Craig et al. 2001; Birkeland 2007a; Corals NPAS 2016. Referenced – DiDonato et al. 2006; Lovell and McLardy 2008.

*Acroporaprolixa* (Verrill, 1866) (1261651) heterotypic synonym. Reported – Hoffmeister 1925; Lamberts 1983; NMNH 2018.

**American Sāmoa status** – Present. **Evidence** – Multiple specimen reports. **Distribution** – American Sāmoa, Ofu, Tutuila. **Nearest confirmed ecoregion** – Sāmoa, Tuvalu, and Tonga. **Vulnerability** – NT.

***Acroporacerealis* (Dana, 1846) (207016)** ^CoTW CCW^

*Acroporaceralis* (Dana, 1846) (207016) [sic]. Reported – USACE 1980.

*Acroporacerealis* (Dana, 1846) (207016). ^CoTW CCW^ Reported – Lamberts 1983; Birkeland et al. 1987, 2003; Itano and Buckley 1988; Maragos et al. 1994; Birkeland 2007a; Fenner et al. 2008; Kenyon et al. 2010; DMWR 2018; Fenner 2018; NMNH 2018; QM 2018. Referenced – Green et al. 1999; Birkeland 2007b; Lovell and McLardy 2008.

*Acroporacerealis* cf. (Dana, 1846) (207016). ^CoTW CCW^ Reported – DMWR 2018.

*Acroporacerialis* (Dana, 1846) (207016) [sic]. Reported – Mundy 1996. Referenced – Fisk and Birkeland 2002.

*Acroporacymbicyathus* (Brook, 1893) (207109) heterotypic synonym. Reported – Mayor 1924b; Hoffmeister 1925. Referenced – Hoffmeister 1925.

*Acroporasymbicyathus* (Brook, 1893) (207109) [sic] heterotypic synonym. Reported – Lamberts 1983.

**American Sāmoa status** – Present. **Evidence** – Multiple specimen reports. **Distribution** – American Sāmoa, Aunuʻu, Manuʻa Islands, Ofu, Ofu/Olosega, Rose Atoll, Sāmoa Islands, Taʻū, Tutuila. **Nearest confirmed ecoregion** – Sāmoa, Tuvalu, and Tonga. **Vulnerability** – LC.

***Acroporachesterfieldensis* Veron & Wallace, 1984 (288191)** ^CoTW CCW^

*Acroporachesterfieldensis* Veron & Wallace, 1984 (288191). ^CoTW CCW^ Reported – Fenner 2018.

**American Sāmoa status** – Present. **Evidence** – Single photographic record. **Distribution** – Ofu/Olosega. **Nearest confirmed ecoregion** – Sāmoa, Tuvalu, and Tonga. **Vulnerability** – LC. **Notes** – This species is documented by clear photographic evidence (Fenner 2018) to support its presence in American Sāmoa.

***Acroporaclathrata* (Brook, 1891) (207075)** ^CoTW CCW^

*Acroporaclathrata* (Brook, 1891) (207075). ^CoTW CCW^ Reported – USACE 1980; Lamberts 1983; Birkeland et al. 1987; Maragos et al. 1994; Mundy 1996; Fisk and Birkeland 2002; Coles et al. 2003; Fenner et al. 2008; Bare et al. 2010; Corals NPAS 2016; Fenner and Sudek 2016; DMWR 2018; Fenner 2018; QM 2018. Referenced – Fisk and Birkeland 2002; DiDonato et al. 2006; Birkeland 2007a, 2007b; Lovell and McLardy 2008.

*Acroporacomplanata* (Brook, 1893) (207071) heterotypic synonym. Reported – Birkeland et al. 1987; Corals NPAS 2016. Referenced – Coles et al. 2003; DiDonato et al. 2006; Birkeland 2007b.

*Acroporavasiformis* (Brook, 1893) (207073) heterotypic synonym. Reported – Birkeland et al. 1987.

**American Sāmoa status** – Present. **Evidence** – Multiple specimen reports. **Distribution** – American Sāmoa, Aunuʻu, Ofu, Ofu/Olosega, Taʻū, Tutuila. **Nearest confirmed ecoregion** – Sāmoa, Tuvalu, and Tonga. **Vulnerability** – LC.

***Acroporacytherea* (Dana, 1846) (207095)** ^CoTW CCW^

*Acroporaarcuata* (Brook, 1892) (207089) heterotypic synonym. Referenced – Hoffmeister 1925.

*Acroporaarmata* (Brook, 1892) (206992) heterotypic synonym. Referenced – Hoffmeister 1925.

*Acroporacorymbosa* (Lamarck, 1816) (207018) possible heterotypic synonym. Reported – Mayor 1924b; Hoffmeister 1925; USACE 1980; Lamberts 1983; BPBM 2018.

*Acroporacytharea* (Dana, 1846) (207095) [sic]. Reported – Mundy 1996. Referenced – Fisk and Birkeland 2002.

*Acroporacytherea* (Dana, 1846) (207095). ^CoTW CCW^ Reported – USACE 1980; Lamberts 1983; Birkeland et al. 1987, 2003; Itano and Buckley 1988; Maragos et al. 1994; Fisk and Birkeland 2002; Work and Rameyer 2002; Coles et al. 2003; DiDonato et al. 2006; Fenner et al. 2008; CRED 2011; Corals NPAS 2016; AM 2018; DMWR 2018; Fenner 2018; NMNH 2018; QM 2018. Referenced – Green et al. 1999; Coles et al. 2003; DiDonato et al. 2006; Birkeland 2007a, 2007b; Lovell and McLardy 2008.

*Acroporareticulata* (Brook, 1892) (207021) heterotypic synonym. Reported – Birkeland et al. 1987.

*Acroporareticulata* cf. (Brook, 1892) (207021) heterotypic synonym. Reported – Birkeland et al. 1987.

*Acroporasymmetrica* (Brook, 1891) (207005) heterotypic synonym. Reported – Birkeland et al. 1987. Referenced – Birkeland 2007b.

**American Sāmoa status** – Present. **Evidence** – Multiple specimen reports. **Distribution** – American Sāmoa, Aunuʻu, Manuʻa Islands, Ofu, Ofu/Olosega, Olosega, Sāmoa Islands, Tutuila. **Nearest confirmed ecoregion** – Sāmoa, Tuvalu, and Tonga. **Vulnerability** – LC. **Notes** – The photo of this species reported in Corals NPAS (2016) appears to be incorrect.

***Acroporadigitifera* (Dana, 1846) (207045)** ^CoTW CCW^

*Acroporadigitifera* (Dana, 1846) (207045). ^CoTW CCW^ Reported – USACE 1980; Birkeland et al. 1987, 2003; Itano and Buckley 1988; Maragos et al. 1994; Craig et al. 2001; Fisk and Birkeland 2002; Work and Rameyer 2002; Coles et al. 2003; Wolstenholme et al. 2003; DiDonato et al. 2006; Birkeland 2007a; Kenyon et al. 2010; Corals NPAS 2016; DMWR 2018; Fenner 2018; NMNH 2018; QM 2018. Referenced – Green et al. 1999; Coles et al. 2003; DiDonato et al. 2006; Birkeland 2007a, 2007b; Lovell and McLardy 2008.

*Acroporadigitifera* ? (Dana, 1846) (207045). ^CoTW CCW^ Reported – DMWR 2018.

*Acroporadigitifera* cf. (Dana, 1846) (207045). ^CoTW CCW^ Reported – USACE 1980; Birkeland et al. 2003.

*Acroporaleptocyathus* (Brook, 1891) (207025) heterotypic synonym. Reported – Mayor 1924a, 1924b; Hoffmeister 1925; Dahl and Lamberts 1977; Lamberts 1983; BPBM 2018. Referenced – Hoffmeister 1925; Green et al. 1997.

*Acroporaschmitti* ? Wells, 1950 (288245) heterotypic synonym. ^CoTW^ Referenced – Coles et al. 2003.

*Acroporaschmitti* Wells, 1950 (288245) heterotypic synonym. ^CoTW^ Reported – USACE 1980; Lamberts 1983.

*Acroporawardii* Verrill, 1902 (740141) heterotypic synonym. Reported – Birkeland et al. 1987.

**American Sāmoa status** – Present. **Evidence** – Multiple specimen reports. **Distribution** – American Sāmoa, Aunuʻu, Manuʻa Islands, Ofu, Ofu/Olosega, Olosega, Rose Atoll, Sāmoa Islands, Taʻū, Tutuila. **Nearest confirmed ecoregion** – Sāmoa, Tuvalu, and Tonga. **Vulnerability** – NT. **Notes** – This species includes the synonym *Acroporaschmitti* Wells, 1950 that is recognized by Veron et al. (2019), but not by Wallace (1999) nor Wallace et al. (2012). Randall and Myers (1983) recognized *Acroporawardii* Verrill, 1902 as a valid species that appears to be different from *A.digitifera*.

***Acroporadivaricata* (Dana, 1846) (207106)** ^CoTW CCW^

*Acroporadivaricata* (Dana, 1846) (207106). ^CoTW CCW^ Reported – Birkeland et al. 1987; Mundy 1996; Fisk and Birkeland 2002; DiDonato et al. 2006; Fenner et al. 2008; Corals NPAS 2016; QM 2018. Referenced – Fisk and Birkeland 2002; Coles et al. 2003; DiDonato et al. 2006; Birkeland 2007b; Lovell and McLardy 2008.

**American Sāmoa status** – Present. **Evidence** – Single specimen report (identified by C Wallace). **Distribution** – American Sāmoa, Aunuʻu, Tutuila. **Nearest confirmed ecoregion** – Sāmoa, Tuvalu, and Tonga. **Vulnerability** – NT. **Notes** – This species has been reported by several studies with one of them providing photographic evidence (Corals NPAS 2016) and one specimen report by C Wallace from QM (2018). The coral documented by Corals NPAS (2016) is an uncertain identification. This species can be difficult to identify, but based on the identification by C Wallace we conclude that this species is present.

***Acroporadonei* Veron & Wallace, 1984 (288198)** ^CoTW CCW^

*Acroporaakajimensis* cf. Veron, 1990 (288183) heterotypic synonym. ^CoTW^ Reported – Montgomery et al. 2019.

*Acroporaakajimensis* Veron, 1990 (288183) heterotypic synonym. ^CoTW^ Reported – Fisk and Birkeland 2002; Birkeland 2007a; Fenner 2018. Referenced – Birkeland 2007b.

*Acroporadonei* ? Veron & Wallace, 1984 (288198). ^CoTW CCW^ Reported – Coles et al. 2003.

*Acroporadonei* Veron & Wallace, 1984 (288198). ^CoTW CCW^ Reported – Craig et al. 2001; DiDonato et al. 2006; Birkeland 2007a; Corals NPAS 2016. Referenced – DiDonato et al. 2006; Lovell and McLardy 2008; Kenyon et al. 2011.

**American Sāmoa status** – Present. **Evidence** – Single specimen report (identified by A Montgomery and D Fenner). **Distribution** – American Sāmoa, Ofu, Taʻū, Tutuila. **Nearest confirmed ecoregion** – Sāmoa, Tuvalu, and TongaSāmoa, Tuvalu, and Tonga. **Vulnerability** – VU. **Mesophotic record** – 41 m depth (Montgomery et al. 2019). **Notes** – *Acroporaakajimensis* is a synonym of *A.donei*, but Veron et al. (2019) recognize *A.akajimensis* as a valid species while Wallace (1999) and Wallace et al. (2012) do not. The *A.akajimensis* reported in Montgomery et al. (2019) was based on a skeletal analysis of a sample that matched the description of *A.akajimensis* very closely, and not *A.donei*. *A.donei* is neat and tidy with the radial corallites and branches being relatively blunt and relatively uniform in thickness. *A.akajimensis* appears much more jagged and disorganized, with pointy corallites and branches. More taxonomic research is needed for these species. The nearest confirmed ecoregion for *A.akajimensis* is New Caledonia (Veron et al. 2019).

***Acroporaeurystoma* (Klunzinger, 1879) (207108)** ^CoTW CCW^

*Acroporaeurystoma* (Klunzinger, 1879) (207108). ^CoTW CCW^ Reported – NMNH 2018.

*Acroporapagoenis* Hoffmeister, 1925 (411144) [sic] heterotypic synonym. Reported – Birkeland et al. 1987.

*Acroporapagoensis* Hoffmeister, 1925 (411144) heterotypic synonym. Reported – Hoffmeister 1925; USACE 1980; Lamberts 1983; Birkeland et al. 1987, 2003; Fenner et al. 2008; DMWR 2018; Fenner 2018; NMNH 2018. Referenced – Green et al. 1999; Coles et al. 2003; Birkeland 2007b.

**American Sāmoa status** – Present. **Evidence** – Type (synonym *Acroporapagoensis*). **Distribution** – American Sāmoa, Aunuʻu, Ofu/Olosega, Taʻū, Tutuila. **Nearest confirmed ecoregion** – Red Sea north-central. **Geographical range extension** – Southeast. **Notes** – In Hoffmeisterʻs (1925) original description of *A.pagoensis*, he cites it as similar to *A.eurystoma*. However, Wallace (1999) and Veron et al. (2019) consider this species a probable synonym of *Acroporatenuis* (Dana, 1846). All citations have used the name *A.pagoensis* except for NMNH (2018) in which one specimen from American Sāmoa was identified by S Cairns as *A.eurystoma*. Hoffmeister (1925) further states this species is distinctive from *A.eurystoma*. We consider *A.eurystoma* present based on *A.pagoensis* being synonymized under *A.eurystoma*, however, we believe *A.pagoensis* may be a valid species and *A.eurystoma* is not likely a valid name for the American Sāmoa observations. Veron et al. (2019) and Wallace (1999) consider this species to be endemic to the Red Sea. We believe more taxonomic investigation into this species is warranted, which should include colonies collected from American Sāmoa.

***Acroporagemmifera* (Brook, 1892) (207097)** ^CoTW CCW^

*Acroporagemmifera* (Brook, 1892) (207097). ^CoTW CCW^ Reported – Birkeland et al. 1987, 2003, 2013; Itano and Buckley 1988; Maragos et al. 1994, 1995; Mundy 1996; Craig et al. 2001; Fisk and Birkeland 2002; Work and Rameyer 2002; Coles et al. 2003; Wolstenholme et al. 2003; DiDonato et al. 2006; Birkeland 2007a; Fenner et al. 2008; Kenyon et al. 2010; Corals NPAS 2016; DMWR 2018; Fenner 2018; QM 2018. Referenced – Fisk and Birkeland 2002; Coles et al. 2003; DiDonato et al. 2006; Birkeland 2007a, 2007b; Lovell and McLardy 2008.

*Acroporagemmifera* cf. (Brook, 1892) (207097). ^CoTW CCW^ Reported – Birkeland et al. 1987, 2003. Referenced – Green et al. 1999; Coles et al. 2003.

**American Sāmoa status** – Present. **Evidence** – Multiple specimen reports. **Distribution** – American Sāmoa, Aunuʻu, Manuʻa Islands, Ofu, Ofu/Olosega, Olosega, Rose Atoll, Taʻū, Tutuila. **Nearest confirmed ecoregion** – Sāmoa, Tuvalu, and TongaSāmoa, Tuvalu, and Tonga. **Vulnerability** – LC.

***Acroporaglobiceps* (Dana, 1846) (430645)** ^CoTW CCW^

*Acroporaglobiceps* (Dana, 1846) (430645). ^CoTW CCW^ Reported – Fisk and Birkeland 2002; Birkeland 2007a; Kenyon et al. 2010; Corals NPAS 2016; Fenner and Sudek 2016; DMWR 2018; Fenner 2018. Referenced – Coles et al. 2003; Lovell and McLardy 2008; Kenyon et al. 2011.

*Acroporaglobiceps* ? (Dana, 1846) (430645). ^CoTW CCW^ Reported – DMWR 2018.

*Acroporaglobiceps* cf. (Dana, 1846) (430645). ^CoTW CCW^ Reported – Coles et al. 2003; Corals NPAS 2016. Referenced – DiDonato et al. 2006.

**American Sāmoa status** – Present. **Evidence** – Single specimen report (identified by D Fenner). **Distribution** – American Sāmoa, Aunuʻu, Manuʻa Islands, Ofu, Ofu/Olosega, Rose Atoll, Swains, Taʻū, Tutuila. **Nearest confirmed ecoregion** – Sāmoa, Tuvalu, and Tonga. **Vulnerability** – 𝒯, VU. **Notes** – Fenner (this study) has examined the type specimens of *Acroporahumilis* (Dana, 1846) and *A.globiceps*. All colonies examined (including the ones within the DMWR collection) belong clearly to *A.globiceps*. The name *A.globiceps* was forgotten until Wallace (1999) and Veron (2000) used it again. It appears likely that all reports of *A.humilis* from the Samoan Archipelago are actually *A.globiceps*.

***Acroporagranulosa* (Milne Edwards, 1860) (207093)** ^CoTW CCW^

*Acroporagranulosa* (Milne Edwards, 1860) (207093). ^CoTW CCW^ Reported – USACE 1980; Lamberts 1983; Maragos et al. 1994; Coles et al. 2003; Kenyon et al. 2010; Corals NPAS 2016; DMWR 2018. Referenced – Birkeland 2007b; Lovell and McLardy 2008.

*Acroporagranulosa* cf. (Milne Edwards, 1860) (207093). ^CoTW CCW^ Reported – Coles et al. 2003.

**American Sāmoa status** – Present. **Evidence** – Multiple specimen reports. **Distribution** – American Sāmoa, Rose Atoll, Taʻū, Tutuila. **Nearest confirmed ecoregion** – Sāmoa, Tuvalu, and Tonga. **Vulnerability** – NT.

***Acroporahyacinthus* (Dana, 1846) (207044)** ^CoTW CCW^

*Acroporaconferta* (Quelch, 1886) (207107) heterotypic synonym. Referenced – Hoffmeister 1925.

*Acroporahyacanthus* (Dana, 1846) (207044) [sic]. Reported – BPBM 2018.

*Acroporahyacinthus* (Dana, 1846) (207044). ^CoTW CCW^ Reported – Mayor 1924b; Hoffmeister 1925; USACE 1980; Lamberts 1983; Birkeland et al. 1987, 2003, 2013; Itano and Buckley 1988; Maragos et al. 1994; Mundy 1996; Birkeland and Belliveau 2000; Craig et al. 2001; Fisk and Birkeland 2002; Work and Rameyer 2002; Coles et al. 2003; DiDonato et al. 2006; Birkeland 2007a; Fenner et al. 2008; CRED 2011; Corals NPAS 2016; Fenner and Sudek 2016; DMWR 2018; Fenner 2018; NMNH 2018; QM 2018. Referenced – Dahl and Lamberts 1977; Dahl 1981; Green et al. 1997, 1999; Fisk and Birkeland 2002; Coles et al. 2003; DiDonato et al. 2006; Birkeland 2007a, 2007b; Lovell and McLardy 2008.

*Acroporasurculosa* (Dana, 1846) (207085) heterotypic synonym. Reported – USACE 1980; Lamberts 1983; Birkeland et al. 1987, 2003; Maragos et al. 1994; Corals NPAS 2016; Fenner 2018. Referenced – DiDonato et al. 2006; Birkeland 2007b.

*Acroporasurculosa* cf. (Dana, 1846) (207085) heterotypic synonym. Reported – Coles et al. 2003; DMWR 2018.

**American Sāmoa status** – Present. **Evidence** – Multiple specimen reports. **Distribution** – American Sāmoa, Aunuʻu, Manuʻa Islands, Ofu, Ofu/Olosega, Olosega, Rose Atoll, Sāmoa Islands, Taʻū, Tutuila. **Nearest confirmed ecoregion** – Sāmoa, Tuvalu, and Tonga. **Vulnerability** – NT. **Notes** – While Wallace (1999) and Veron et al. (2019) concur that *Acroporasurculosa* (Dana, 1846) is a synonym of *A.hyacinthus*, Randall and Myers (1983) considered *A.surculosa* to be valid. Randall (1995) reports both *A.surculosa* and *A.hyacinthus* from Palau. Colonies in American Sāmoa fit the type of *A.surculosa* (examined by D Fenner) and appears to be different from *A.hyacinthus*. *Acroporasurculosa* has smaller colonies with longer cone-shaped branchlets that more often appear to be fused or with multiple branch tips with long tentacles out during the day, while *A.hyacinthus* has larger colonies with shorter cylindrical branchlets that donʻt fuse or have multiple branch tips with much smaller tentacles if exposed during the day. Photographs show *A.surculosa* to have significant variation in both Guam (www.guamreeflife.com) and American Sāmoa. More research should pursue the potential for species distinction between these two species.

***Acroporaintermedia* (Brook, 1891) (207035)** ^CoTW CCW^

*Acroporaintermedia* (Brook, 1891) (207035). ^CoTW CCW^ Reported – USACE 1980; Lamberts 1983; Craig et al. 2001; Fisk and Birkeland 2002; BPBM 2018; Fenner 2018; QM 2018; Montgomery et al. 2019. Referenced – Lovell and McLardy 2008.

*Acroporavanderhorsti* Hoffmeister, 1925 (741178) heterotypic synonym. Reported – Mayor 1924b; Hoffmeister 1925; Lamberts 1983; NMNH 2018.

**American Sāmoa status** – Present. **Evidence** – Multiple specimen reports. **Distribution** – American Sāmoa, Aunuʻu, Ofu, Ofu/Olosega, Taʻū, Tutuila. **Nearest confirmed ecoregion** – Sāmoa, Tuvalu, and Tonga. **Mesophotic record** – 37 m depth (Montgomery et al. 2019). **Notes** – This species has also been called *Acroporanobilis* (Dana, 1846). Wallace (1999) describes the relationship between *A.intermedia*, *Acroporarobusta* (Dana, 1846), and *A.nobilis*.

***Acroporajacquelineae* Wallace, 1994 (288212)** ^CoTW CCW^

*Acroporajacquelineae* Wallace, 1994 (288212). ^CoTW CCW^ Reported – Fenner 2018. Referenced – Kenyon et al. 2011.

*Acroporajacquilinae* Wallace, 1994 (288212) [sic]. Reported – DMWR 2018.

**American Sāmoa status** – Present. **Evidence** – Single specimen report (identified by D Fenner). **Distribution** – Taʻū, Tutuila. **Nearest confirmed ecoregion** – Sāmoa, Tuvalu, and Tonga. **Vulnerability** – 𝒯, VU. **Notes** – There have been only two references that report this species, but T Hughes also reports seeing it on Taʻū (T Hughes, pers. comm.). An examination of an *A.jacquelineae* sample by D Luck concluded that it was not this species based on the fact that the axials were slightly smaller than reported in Wallace (1999) and that there were many small radial corallites while Wallace (1999) reported there are very few such radials in this species (Luck 2013). Examination of skeletal photographs in Wallace (1999) clearly show that corallites in most of the colony indeed have very few radials, but corallites near the edge of the colony have many radials. The sample analyzed by D Luck was taken from the edge of the colony by D Fenner and D Luck likely did not realize that the sample was taken from the edge. Given that this species is listed as threatened under the ESA, careful attention has been paid to the presence of this species and we believe the evidence in hand is sufficient to conclude its presence in American Sāmoa albeit likely as a rare species.

***Acroporalatistella* (Brook, 1892) (207039)** ^CoTW CCW^

*Acroporalatistella /azurea* (Brook, 1892) (207039). Reported – DMWR 2018.

*Acroporalatistella* (Brook, 1892) (207039). ^CoTW CCW^ Reported – USACE 1980; Lamberts 1983; Craig et al. 2001; Fisk and Birkeland 2002; Birkeland et al. 2003; Birkeland 2007a; Kenyon et al. 2010; Corals NPAS 2016; Fenner 2018; NMNH 2018; QM 2018; Montgomery et al. 2019. Referenced – DiDonato et al. 2006; Birkeland 2007b; Lovell and McLardy 2008.

*Acroporalatistella* ? (Brook, 1892) (207039). ^CoTW CCW^ Reported – Coles et al. 2003.

**American Sāmoa status** – Present. **Evidence** – Multiple specimen reports. **Distribution** – American Sāmoa, Aunuʻu, Manuʻa Islands, Ofu, Rose Atoll, Taʻū, Tutuila. **Nearest confirmed ecoregion** – Sāmoa, Tuvalu, and Tonga. **Vulnerability** – LC. **Mesophotic record** – 42 m depth (Montgomery et al. 2019).

***Acroporalisteri* (Brook, 1893) (207057)** ^CoTW CCW^

*Acroporalisteri* (Brook, 1893) (207057). ^CoTW CCW^ Reported – DiDonato et al. 2006; Kenyon et al. 2010; Corals NPAS 2016; QM 2018. Referenced – Coles et al. 2003; Lovell and McLardy 2008; Kenyon et al. 2011.

*Acroporalisteri* cf. (Brook, 1893) (207057). ^CoTW CCW^ Reported – DMWR 2018.

**American Sāmoa status** – Present. **Evidence** – Single specimen report (identified by C Wallace). **Distribution** – American Sāmoa, Rose Atoll, Taʻū, Tutuila. **Nearest confirmed ecoregion** – Sāmoa, Tuvalu, and Tonga. **Vulnerability** – VU. **Notes** – A photographic record exists for this species (Corals NPAS 2016); however, this may be incorrectly identified. In addition, there is a single specimen reported by QM (2018) identified by C Wallace.

***Acroporalongicyathus* (Milne Edwards, 1860) (207114)** ^CoTW CCW^

*Acroporalongicyathus* (Milne Edwards, 1860) (207114). ^CoTW CCW^ Reported – USACE 1980; Lamberts 1983; Kenyon et al. 2010. Referenced – Lovell and McLardy 2008.

*Acroporasyringodes* (Brook, 1892) (207014) heterotypic synonym. Reported – Mayor 1924b; Hoffmeister 1925; Lamberts 1983; NMNH 2018. Referenced – Hoffmeister 1925.

*Acroporasyringoides* (Brook, 1892) (207014) [sic] heterotypic synonym. Referenced – Birkeland 2007b.

**American Sāmoa status** – Present. **Evidence** – Multiple specimen reports. **Distribution** – American Sāmoa, Rose Atoll, Sāmoa Islands, Tutuila. **Nearest confirmed ecoregion** – Sāmoa, Tuvalu, and Tonga. **Vulnerability** – LC.

***Acroporalutkeni* Crossland, 1952 (206994)** ^CoTW CCW^

*Acroporalutkeni* cf. Crossland, 1952 (206994). ^CoTW CCW^ Reported – DMWR 2018.

*Acroporalutkeni* Crossland, 1952 (206994). ^CoTW CCW^ Reported – Maragos et al. 1994; Fisk and Birkeland 2002; Corals NPAS 2016; QM 2018. Referenced – DiDonato et al. 2006; Birkeland 2007b; Lovell and McLardy 2008.

**American Sāmoa status** – Present. **Evidence** – Single specimen report (identified by C Wallace). **Distribution** – American Sāmoa, Taʻū, Tutuila. **Nearest confirmed ecoregion** – Sāmoa, Tuvalu, and Tonga. **Vulnerability** – NT. **Notes** – QM (2018) reports this species identified by C Wallace.

***Acroporamicroclados* (Ehrenberg, 1834) (207101)** ^CoTW CCW^

*Acroporaassimilis* Brook, 1892 (741262) [sic] heterotypic synonym. Reported – NMNH 2018.

*Acroporamicroclados* (Ehrenberg, 1834) (207101). ^CoTW CCW^ Reported – Fisk and Birkeland 2002; DiDonato et al. 2006; Corals NPAS 2016; DMWR 2018. Referenced – Lovell and McLardy 2008; Kenyon et al. 2011.

*Acroporamicroclados* ? (Ehrenberg, 1834) (207101). ^CoTW CCW^ Reported – DMWR 2018.

**American Sāmoa status** – Present. **Evidence** – Multiple specimen reports. **Distribution** – American Sāmoa, Taʻū, Tutuila. **Nearest confirmed ecoregion** – Sāmoa, Tuvalu, and Tonga. **Vulnerability** – VU. **Notes** – The photographic record by Corals NPAS (2016) is incorrect, but there are multiple other specimen reports.

***Acroporamillepora* (Ehrenberg, 1834) (207023)** ^CoTW CCW^

*Acroporaconvexa* cf. (Dana, 1846) (367986) heterotypic synonym. ^CoTW^ Reported – Birkeland et al. 2003.

*Acroporamillepora* (Ehrenberg, 1834) (207023). ^CoTW CCW^ Reported – USACE 1980; Lamberts 1983; Birkeland 2007a; Birkeland et al. 2013; BPBM 2018. Referenced – Green et al. 1999; Coles et al. 2003; Lovell and McLardy 2008.

*Acroporamillepora* cf. (Ehrenberg, 1834) (207023). ^CoTW CCW^ Reported – DMWR 2018.

*Acroporaprostrata* (Dana, 1846) (207084) heterotypic synonym. Reported – Fisk and Birkeland 2002.

*Acroporaprostrata* ? (Dana, 1846) (207084) heterotypic synonym. Reported – Coles et al. 2003.

*Acroporasquamosa* Brook, 1892 (741205) heterotypic synonym. Reported – Birkeland et al. 1987. Referenced – Coles et al. 2003; Birkeland 2007b.

**American Sāmoa status** – Present. **Evidence** – Multiple specimen reports. **Distribution** – American Sāmoa, Ofu, Taʻū, Tutuila. **Nearest confirmed ecoregion** – Sāmoa, Tuvalu, and Tonga. **Vulnerability** – NT.

***Acroporamonticulosa* (Brüggemann, 1879) (207103)** ^CoTW CCW^

*Acroporamonticulosa* (Brüggemann, 1879) (207103). ^CoTW CCW^ Reported – Birkeland et al. 1987; Itano and Buckley 1988; Maragos et al. 1994, 1995; Mundy 1996; Coles et al. 2003; Wolstenholme et al. 2003; Birkeland 2007a; Corals NPAS 2016; DMWR 2018; Fenner 2018; QM 2018. Referenced – Green et al. 1999; Fisk and Birkeland 2002; Coles et al. 2003; DiDonato et al. 2006; Birkeland 2007b; Lovell and McLardy 2008.

**American Sāmoa status** – Present. **Evidence** – Multiple specimen reports. **Distribution** – American Sāmoa, Aunuʻu, Manuʻa Islands, Ofu, Ofu/Olosega, Olosega, Taʻū, Tutuila. **Nearest confirmed ecoregion** – Sāmoa, Tuvalu, and Tonga. **Vulnerability** – NT.

***Acroporamuricata* (Linnaeus, 1758) (207007)** ^CoTW CCW^

*Acroporaarbuscula* (Dana, 1846) (207003) heterotypic synonym. Reported – USACE 1980; Lamberts 1983.

*Acroporaformosagracilis* (Dana, 1846) (207036) heterotypic synonym. Reported – NMNH 2018.

Acroporaformosavar.brachiata (Dana, 1846) (207036) heterotypic synonym. Reported – Hoffmeister 1925. Referenced – Green et al. 1997.

Acroporaformosavar.gracilis (Dana, 1846) (207036) heterotypic synonym. Reported – Hoffmeister 1925. Referenced – Green et al. 1997.

Acroporaformosavar.gracilis aff. (Dana, 1846) (207036) heterotypic synonym. Reported – Mayor 1924b.

*Acroporaformosa* (Dana, 1846) (207036) heterotypic synonym. Reported – Mayor 1924a; Hoffmeister 1925; USACE 1980; Lamberts 1983; Birkeland et al. 1987; Maragos et al. 1994; Mundy 1996; Green and Hunter 1998; Mundy and Green 1999; Birkeland and Belliveau 2000; DiDonato et al. 2006; Fenner et al. 2008; Corals NPAS 2016; BPBM 2018; NMNH 2018. Referenced – Dahl and Lamberts 1977; Dahl 1981; Green et al. 1997; Coles et al. 2003; DiDonato et al. 2006.

*Acroporaformosa* cf. (Dana, 1846) (207036) heterotypic synonym. Reported – USACE 1980.

*Acroporagracilis* (Dana, 1846) (207060) heterotypic synonym. Reported – Kenyon et al. 2010. Referenced – Hoffmeister 1925.

*Acroporamuricata* (Linnaeus, 1758) (207007). ^CoTW CCW^ Reported – Mayor 1924a; Dahl and Lamberts 1977; Birkeland and Belliveau 2000; Craig et al. 2001; Fisk and Birkeland 2002; Coles et al. 2003; Birkeland 2007a; Birkeland et al. 2013; Corals NPAS 2016; Fenner and Sudek 2016; DMWR 2018; Fenner 2018; QM 2018. Referenced – DiDonato et al. 2006; Birkeland 2007a, 2007b; Lovell and McLardy 2008.

**American Sāmoa status** – Present. **Evidence** – Multiple specimen reports. **Distribution** – American Sāmoa, Aunuʻu, Ofu, Ofu/Olosega, Olosega, Sāmoa Islands, Taʻū, Tutuila. **Nearest confirmed ecoregion** – Sāmoa, Tuvalu, and Tonga.

***Acroporanana* (Studer, 1879) (207100)** ^CoTW CCW^

*Acroporaazura* Veron & Wallace, 1984 (288186) [sic] heterotypic synonym. Reported – Corals NPAS 2016.

*Acroporaazurea* Veron & Wallace, 1984 (288186) heterotypic synonym. ^CoTW^ Reported – Birkeland et al. 1987, 2003; Mundy 1996; Fenner et al. 2008. Referenced – Green et al. 1999; Fisk and Birkeland 2002; DiDonato et al. 2006; Birkeland 2007a, 2007b; Lovell and McLardy 2008.

*Acroporanana /valida* (Studer, 1879) (207100). Reported – DMWR 2018.

*Acroporanana* (Studer, 1879) (207100). ^CoTW CCW^ Reported – USACE 1980; Lamberts 1983; Birkeland et al. 1987, 2003, 2013; Maragos et al. 1994; Mundy 1996; Green et al. 1997; Birkeland and Belliveau 2000; Fenner et al. 2008; Kenyon et al. 2010; Corals NPAS 2016; Fenner and Sudek 2016; DMWR 2018; Fenner 2018; QM 2018. Referenced – Dahl and Lamberts 1977; Dahl 1981; Green et al. 1997; Fisk and Birkeland 2002; Coles et al. 2003; DiDonato et al. 2006; Birkeland 2007a, 2007b; Lovell and McLardy 2008.

*Acroporanana* cf. (Studer, 1879) (207100). ^CoTW CCW^ Reported – Birkeland et al. 1987. Referenced – Green et al. 1999; Coles et al. 2003.

**American Sāmoa status** – Present. **Evidence** – Multiple specimen reports. **Distribution** – American Sāmoa, Aunuʻu, Manuʻa Islands, Ofu, Ofu/Olosega, Olosega, Rose Atoll, Taʻū, Tutuila. **Nearest confirmed ecoregion** – Sāmoa, Tuvalu, and Tonga. **Vulnerability** – NT.

***Acroporanasuta* (Dana, 1846) (207009)** ^CoTW CCW^

*Acroporacanaliculata* (Klunzinger, 1879) (1262051) heterotypic synonym. Reported – NMNH 2018.

*Acroporanasuta* (Dana, 1846) (207009). ^CoTW CCW^ Reported – USACE 1980; Lamberts 1983; Birkeland et al. 1987, 2003; Itano and Buckley 1988; Maragos et al. 1994, 1995; Mundy 1996; Craig et al. 2001; Fisk and Birkeland 2002; Coles et al. 2003; DiDonato et al. 2006; Birkeland 2007a; Fenner et al. 2008; Bare et al. 2010; Kenyon et al. 2010; Corals NPAS 2016; AM 2018; DMWR 2018; Fenner 2018; QM 2018. Referenced – Green et al. 1999; Fisk and Birkeland 2002; Coles et al. 2003; DiDonato et al. 2006; Birkeland 2007a, 2007b; Lovell and McLardy 2008.

*Acroporanasuta* cf. (Dana, 1846) (207009). ^CoTW CCW^ Reported – DMWR 2018.

**American Sāmoa status** – Present. **Evidence** – Multiple specimen reports. **Distribution** – American Sāmoa, Aunuʻu, Manuʻa Islands, Ofu, Ofu/Olosega, Olosega, Rose Atoll, Taʻū, Tutuila. **Nearest confirmed ecoregion** – Sāmoa, Tuvalu, and Tonga. **Vulnerability** – NT.

***Acroporapalmerae* Wells, 1954 (207049)** ^CoTW CCW^

*Acroporapalmera* Wells, 1954 (207049) [sic]. Reported – Corals NPAS 2016.

*Acroporapalmerae* Wells, 1954 (207049). ^CoTW CCW^ Reported – USACE 1980; Lamberts 1983; Birkeland et al. 1987; Maragos et al. 1994; Coles et al. 2003; DMWR 2018; Fenner 2018. Referenced – Green et al. 1999; Coles et al. 2003; DiDonato et al. 2006; Birkeland 2007b; Lovell and McLardy 2008; Kenyon et al. 2011.

*Acroporapalmeri* Wells, 1954 (207049) [sic]. Reported – USACE 1980.

**American Sāmoa status** – Present. **Evidence** – Multiple specimen reports. **Distribution** – American Sāmoa, Aunuʻu, Ofu, Tutuila. **Nearest confirmed ecoregion** – Sāmoa, Tuvalu, and Tonga. **Vulnerability** – VU. **Notes** – Wallace (1999) and Veron (2000) point out that this species has no differences in corallites or coenosteum with *Acroporarobusta* (Dana, 1846), only the differences in colony shape (almost all branches versus almost all encrusting). This raises the question if these two are separate species or not.

***Acroporapaniculata* Verrill, 1902 (207008)** ^CoTW CCW^

*Acroporapanicualta* Verrill, 1902 (207008) [sic]. Reported – DMWR 2018.

*Acroporapaniculata* Verrill, 1902 (207008). ^CoTW CCW^ Reported – USACE 1980; Lamberts 1983; Maragos et al. 1994; Mundy 1996; Fisk and Birkeland 2002; Coles et al. 2003; Fenner et al. 2008; Kenyon et al. 2010; CRED 2011; Corals NPAS 2016; DMWR 2018; Fenner 2018; Montgomery et al. 2019. Referenced – Fisk and Birkeland 2002; DiDonato et al. 2006; Birkeland 2007a, 2007b; Lovell and McLardy 2008; Kenyon et al. 2011.

**American Sāmoa status** – Present. **Evidence** – Multiple specimen reports. **Distribution** – American Sāmoa, Aunuʻu, Ofu, Ofu/Olosega, Rose Atoll, Taʻū, Tutuila. **Nearest confirmed ecoregion** – Sāmoa, Tuvalu, and Tonga. **Vulnerability** – VU. **Mesophotic record** – 41 m depth (Montgomery et al. 2019). **Notes** – The photographic record by Corals NPAS (2016) is incorrect, but there are multiple specimen reports.

***Acroporapolystoma* (Brook, 1891) (207050)** ^CoTW CCW^

*Acroporamassawensis* von Marenzeller, 1907 (207004) heterotypic synonym. ^CoTW^ Reported – Mayor 1924b; Hoffmeister 1925; USACE 1980; Lamberts 1983.

*Acroporapolystoma* (Brook, 1891) (207050). ^CoTW CCW^ Reported – Maragos et al. 1994; Corals NPAS 2016; NMNH 2018; QM 2018. Referenced – DiDonato et al. 2006; Birkeland 2007a; Lovell and McLardy 2008; Kenyon et al. 2011.

**American Sāmoa status** – Present. **Evidence** – Multiple specimen reports. **Distribution** – American Sāmoa, Ofu, Ofu/Olosega, Tutuila. **Nearest confirmed ecoregion** – Sāmoa, Tuvalu, and Tonga. **Vulnerability** – VU.

***Acroporapulchra* (Brook, 1891) (207015)** ^CoTW CCW^

*Acroporapulchra* (Brook, 1891) (207015). ^CoTW CCW^ Reported – Mayor 1924b; USACE 1980; Lamberts 1983; Mundy 1996; Craig et al. 2001; Fisk and Birkeland 2002; DiDonato et al. 2006; Birkeland 2007a; Corals NPAS 2016; Fenner and Sudek 2016; BPBM 2018; DMWR 2018; Fenner 2018; QM 2018. Referenced – DiDonato et al. 2006; Birkeland 2007a; Lovell and McLardy 2008.

*Acroporapulchra* ? (Brook, 1891) (207015). ^CoTW CCW^ Reported – Coles et al. 2003.

**American Sāmoa status** – Present. **Evidence** – Multiple specimen reports. **Distribution** – American Sāmoa, Manuʻa Islands, Ofu, Ofu/Olosega, Tutuila. **Nearest confirmed ecoregion** – Sāmoa, Tuvalu, and Tonga. **Vulnerability** – LC.

***Acroporaretusa* (Dana, 1846) (430653)** ^CoTW CCW^

*Acroporaretusa* (Dana, 1846) (430653). ^CoTW CCW^ Reported – Fisk and Birkeland 2002; DiDonato et al. 2006; Birkeland 2007a; Kenyon et al. 2010; Corals NPAS 2016; Fenner 2018; QM 2018. Referenced – Lovell and McLardy 2008; Kenyon et al. 2011.

**American Sāmoa status** – Present. **Evidence** – Single specimen report (identified by C Wallace). **Distribution** – American Sāmoa, Ofu, Ofu/Olosega, Rose Atoll, Taʻū, Tutuila. **Nearest confirmed ecoregion** – Sāmoa, Tuvalu, and Tonga. **Vulnerability** – 𝒯, VU. **Notes** – The photographic record by Corals NPAS (2016) is incorrect, but Fenner (2018) shows clear evidence of its presence in addition to the QM (2018) specimen identified by C Wallace.

***Acroporarobusta* (Dana, 1846) (207000)** ^CoTW CCW^

*Acroporacuspidata* (Dana, 1846) (872427) heterotypic synonym. Reported – USACE 1980; Lamberts 1983.

*Acroporanobilis* (Dana, 1846) (207090) heterotypic synonym. Reported – Hoffmeister 1925; USACE 1980; Lamberts 1983; Birkeland et al. 1987, 2003; Maragos et al. 1994; Mundy 1996; DiDonato et al. 2006; Birkeland 2007a; Fenner et al. 2008; CRED 2011; Corals NPAS 2016; DMWR 2018. Referenced – Green et al. 1999; Fisk and Birkeland 2002; Coles et al. 2003; DiDonato et al. 2006; Birkeland 2007a, 2007b.

*Acroporanobilis* ? (Dana, 1846) (207090) heterotypic synonym. Reported – Coles et al. 2003.

*Acroporapacifica* (Brook, 1891) (207033) heterotypic synonym. Referenced – Hoffmeister 1925.

*Acroporapaxilligera* (Dana, 1846) (872424) heterotypic synonym. Reported – Birkeland et al. 1987, 2003. Referenced – Green et al. 1999; Coles et al. 2003; Birkeland 2007b.

*Acroporapinguis* Wells, 1950 (207070) heterotypic synonym. ^CoTW^ Reported – Lamberts 1983.

*Acroporapinquis* aff. Wells, 1950 (207070) [sic] heterotypic synonym. Reported – USACE 1980.

*Acroporapinquis* Wells, 1950 (207070) [sic] heterotypic synonym. Reported – USACE 1980.

*Acroporarobusta* (Dana, 1846) (207000). ^CoTW CCW^ Reported – USACE 1980; Lamberts 1983; Birkeland et al. 1987, 2003; Hunter et al. 1993; Maragos et al. 1994, 1995; Green and Hunter 1998; DiDonato et al. 2006; Bare et al. 2010; Corals NPAS 2016; DMWR 2018; Fenner 2018; NMNH 2018; QM 2018. Referenced – Green et al. 1999; Coles et al. 2003; DiDonato et al. 2006; Birkeland 2007a, 2007b; Lovell and McLardy 2008.

*Acroporarobusta* ? (Dana, 1846) (207000). ^CoTW CCW^ Reported – Coles et al. 2003. Referenced – Coles et al. 2003.

*Acroporarobusta* cf. (Dana, 1846) (207000). ^CoTW CCW^ Reported – Hunter et al. 1993.

*Acroporasmithi* (Brook, 1893) (368476) heterotypic synonym. Reported – Birkeland et al. 1987; Maragos et al. 1994. Referenced – Green et al. 1999; Coles et al. 2003; Birkeland 2007b.

**American Sāmoa status** – Present. **Evidence** – Multiple specimen reports. **Distribution** – American Sāmoa, Aunuʻu, Manuʻa Islands, Ofu, Ofu/Olosega, Olosega, Sāmoa Islands, Tutuila. **Nearest confirmed ecoregion** – Sāmoa, Tuvalu, and Tonga. **Vulnerability** – LC. **Notes** – *Acroporanobilis* has been synonymized with *A.robusta* by Wallace (1999) and accepted by Veron et al. (2019). Fenner has examined its type of *A.nobilis* in NMNH and agrees with Veron et al. (2019). Many reports of *A.intermedia* and *A.nobilis* in American Sāmoa may actually be *A.intermedia*. The photographic record by Corals NPAS (2016) is incorrect. However, given that multiple specimens have been identified from American Sāmoa, we believe that this species is present in American Sāmoa.

***Acroporasamoensis* (Brook, 1891) (207055)** ^CoTW CCW^

*Acroporasamoensis* (Brook, 1891) (207055). ^CoTW CCW^ Reported – Mayor 1924a, 1924b; Hoffmeister 1925; Dahl and Lamberts 1977; Lamberts 1983; Birkeland et al. 1987, 2003, 2013; Maragos et al. 1994, 1995; Mundy 1996; Craig et al. 2001; Fisk and Birkeland 2002; Work and Rameyer 2002; Coles et al. 2003; DiDonato et al. 2006; Birkeland 2007a; Fenner et al. 2008; Kenyon et al. 2010; CRED 2011; Corals NPAS 2016; BPBM 2018; NMNH 2018. Referenced – Hoffmeister 1925; Green et al. 1997, 1999; Fisk and Birkeland 2002; Coles et al. 2003; DiDonato et al. 2006; Birkeland 2007a, 2007b; Lovell and McLardy 2008.

*Acroporasamoensis* ? (Brook, 1891) (207055). ^CoTW CCW^ Reported – DMWR 2018.

*Acroporasamoensis* aff. (Brook, 1891) (207055). ^CoTW CCW^ Reported – Mayor 1924b.

*Acroporasamoensis* cf. (Brook, 1891) (207055). ^CoTW CCW^ Reported – DMWR 2018. Referenced – Coles et al. 2003.

**American Sāmoa status** – Present. **Evidence** – Multiple specimen reports. **Distribution** – American Sāmoa, Aunuʻu, Manuʻa Islands, Ofu, Ofu/Olosega, Olosega, Rose Atoll, Sāmoa Islands, Taʻū, Tutuila. **Nearest confirmed ecoregion** – Sāmoa, Tuvalu, and Tonga. **Vulnerability** – LC. **Notes** – The type locality of this species is “Sāmoa Islands.” This could be either American Sāmoa or Independent Sāmoa. These two political entities are parts of the same archipelago, and thus many species present in one are likely in the other. The photographic record by Corals NPAS (2016) is incorrect.

***Acroporasecale* (Studer, 1878) (207080)** ^CoTW CCW^

*Acroporadiversa* (Brook, 1891) (207054) heterotypic synonym. Reported – USACE 1980; Lamberts 1983.

*Acroporadiversa* cf. (Brook, 1891) (207054) heterotypic synonym. Reported – Coles et al. 2003.

*Acroporaquelchi* (Brook, 1893) (207022) heterotypic synonym. Reported – Hoffmeister 1925; Lamberts 1983. Referenced – Dahl and Lamberts 1977; Dahl 1981; Green et al. 1997.

*Acroporaquelchi* cf. (Brook, 1893) (207022) heterotypic synonym. Reported – Coles et al. 2003.

*Acroporasecale* (Studer, 1878) (207080). ^CoTW CCW^ Reported – Fisk and Birkeland 2002; DiDonato et al. 2006; Corals NPAS 2016; DMWR 2018; NMNH 2018; QM 2018. Referenced – Lovell and McLardy 2008.

*Acroporasecale* cf. (Studer, 1878) (207080). ^CoTW CCW^ Reported – DMWR 2018.

*Acropra secale /valida/kimbiensis* (Studer, 1878) (207080) [sic]. Reported – DMWR 2018.

**American Sāmoa status** – Present. **Evidence** – Multiple specimen reports. **Distribution** – American Sāmoa, Aunuʻu, Manuʻa Islands, Ofu, Olosega, Taʻū, Tutuila. **Nearest confirmed ecoregion** – Sāmoa, Tuvalu, and Tonga. **Vulnerability** – NT. **Notes** – This species is tough to identify in situ, but based on multiple specimens of this species and its synonyms we believe this species to be present.

***Acroporaselago* (Studer, 1879) (207040)** ^CoTW CCW^

*Acroporadelicatula* (Brook, 1891) (207082) [sic] heterotypic synonym. Reported – USACE 1980; Lamberts 1983; Maragos et al. 1994, 1995; Birkeland et al. 2003; Corals NPAS 2016. Referenced – DiDonato et al. 2006; Birkeland 2007b.

*Acroporainsignis* Nemenzo, 1967 (288211) possible heterotypic synonym. ^CoTW^ Reported – DMWR 2018; Fenner 2018.

*Acroporaselago* (Studer, 1879) (207040). ^CoTW CCW^ Reported – Fisk and Birkeland 2002; Coles et al. 2003; Birkeland 2007a; Kenyon et al. 2010. Referenced – Green et al. 1999; Coles et al. 2003; Lovell and McLardy 2008.

**American Sāmoa status** – Present. **Evidence** – Multiple specimen reports. **Distribution** – American Sāmoa, Aunuʻu, Ofu, Rose Atoll, Tutuila. **Nearest confirmed ecoregion** – Sāmoa, Tuvalu, and Tonga. **Vulnerability** – NT. **Notes** – Veron (2000) indicates that *A.insignis* is quite different from *A.selago*, which has more appressed radial corallites that are labellate and *A.insignis* has a distinctive coloration. This makes this species easy to distinguish in the water. Given the dispute with this synonym, more work should be conducted to look at the species variation in this group. Randall & Myers (1983) and Randall (1995, 2003) consider *Acroporadeliculata* (Brook, 1891) a valid species.

***Acroporasolitaryensis* Veron & Wallace, 1984 (288248)** ^CoTW CCW^

*Acroporasolitaryensis* Veron & Wallace, 1984 (288248). ^CoTW CCW^ Reported – QM 2018; Montgomery et al. 2019.

**American Sāmoa status** – Present. **Evidence** – Multiple specimen reports. **Distribution** – Tutuila. **Nearest confirmed ecoregion** – Vanuatu and Cook Islands, central Pacific. **Geographical range extension** – Between two disjunct ecoregions although Veron et al. (2019) strongly predicted the presence of this species in the Sāmoa, Tuvalu, and Tonga ecoregion. **Vulnerability** – VU. **Mesophotic record** – 44 m depth (Montgomery et al. 2019). **Notes** – This species is confirmed by multiple specimen reports (QM 2018, Montgomery et al. 2019).

***Acroporaspeciosa* (Quelch, 1886) (430655)** ^CoTW CCW^

*Acroporarambleri* (Bassett-Smith, 1890) (207088) possible heterotypic synonym. Reported – USACE 1980; Lamberts 1983; Birkeland et al. 1987. Referenced – Birkeland 2007b; Lovell and McLardy 2008.

*Acroporarayneri* Brook, 1892 (751726) [sic] heterotypic synonym. Reported – BPBM 2018.

*Acroporaspeciosa* (Quelch, 1886) (430655). ^CoTW CCW^ Reported – Bare et al. 2010; DMWR 2018; Fenner 2018; Montgomery et al. 2019.

**American Sāmoa status** – Present. **Evidence** – Multiple specimen reports. **Distribution** – American Sāmoa, Tutuila. **Nearest confirmed ecoregion** – Fiji and Society Islands, French Polynesia. **Geographical range extension** – Between two disjunct ecoregions. **Vulnerability** – 𝒯, VU. **Mesophotic record** – 46 m depth (Montgomery et al. 2019).

***Acroporatenuis* (Dana, 1846) (207105)** ^CoTW CCW^

*Acroporaafricana* (Brook, 1893) (207063) heterotypic synonym. Reported – Hoffmeister 1925; USACE 1980; Lamberts 1983.

*Acroporatenuis* (Dana, 1846) (207105). ^CoTW CCW^ Reported – Birkeland et al. 1987, 2003; Mundy 1996; Craig et al. 2001; Fisk and Birkeland 2002; Coles et al. 2003; DiDonato et al. 2006; Kenyon et al. 2010; Corals NPAS 2016; NMNH 2018; QM 2018. Referenced – Green et al. 1999; Fisk and Birkeland 2002; Coles et al. 2003; DiDonato et al. 2006; Birkeland 2007a, 2007b; Lovell and McLardy 2008.

**American Sāmoa status** – Present. **Evidence** – Multiple specimen reports. **Distribution** – American Sāmoa, Aunuʻu, Manuʻa Islands, Ofu, Olosega, Rose Atoll, Tutuila. **Nearest confirmed ecoregion** – Sāmoa, Tuvalu, and Tonga. **Vulnerability** – NT. **Notes** – The photographic record by Corals NPAS (2016) is incorrect, but there are multiple specimen reports.

***Acroporateres* (Verrill, 1866) (288255)**^CoTW^ taxon inquirendum

*Acroporateres* (Verrill, 1866) (288255). ^CoTW^ Reported – Mayor 1924b; Hoffmeister 1925; USACE 1980; Lamberts 1983; Birkeland 2007a; NMNH 2018. Referenced – Coles et al. 2003.

*Acroporateres* cf. (Verrill, 1866) (288255). ^CoTW^ Reported – USACE 1980.

**American Sāmoa status** – Present. **Evidence** – Multiple specimen reports. **Distribution** – American Sāmoa, Ofu, Tutuila. **Nearest confirmed ecoregion** – Marshall Islands and Solomon Islands and Bougainville. **Geographical range extension** – Southeast. **Vulnerability** – DD. **Notes** – *Acroporateres* (Verrill, 1886) has not been recorded in American Sāmoa since Hoffmeister (1925) and Lamberts (1983), except for Birkeland (2007a). The observations by Birkeland (2007a) in Ofu Lagoon concerned preliminary identifications without verification making the present record unverifiable. Given that there have been multiple specimen reports documenting this species, it is possible that this species has been extirpated and is no longer present in American Sāmoa.

***Acroporavalida* (Dana, 1846) (207072)** ^CoTW CCW^

*Acroporavalida* (Dana, 1846) (207072). ^CoTW CCW^ Reported – Mayor 1924b; Hoffmeister 1925; USACE 1980; Lamberts 1983; Birkeland et al. 1987, 2003; Itano and Buckley 1988; Maragos et al. 1994, 1995; Mundy 1996; Birkeland and Belliveau 2000; Craig et al. 2001; Fisk and Birkeland 2002; Coles et al. 2003; DiDonato et al. 2006; Birkeland 2007a; Fenner et al. 2008; Kenyon et al. 2010; CRED 2011; Corals NPAS 2016; NMNH 2018; QM 2018. Referenced – Green et al. 1999; Coles et al. 2003; DiDonato et al. 2006; Birkeland 2007a, 2007b; Lovell and McLardy 2008.

*Acroporavalida* aff. (Dana, 1846) (207072). ^CoTW CCW^ Reported – Coles et al. 2003.

*Acroporavalida* cf. (Dana, 1846) (207072). ^CoTW CCW^ Reported – Maragos et al. 1994.

*Acroporavariabilis* (Klunzinger, 1879) (207028) heterotypic synonym. ^CoTW^ Reported – USACE 1980; Lamberts 1983; Maragos et al. 1994; BPBM 2018. Referenced – Birkeland 2007b.

*Acroporavariabilis* aff. (Klunzinger, 1879) (207028) heterotypic synonym. ^CoTW^ Reported – USACE 1980.

**American Sāmoa status** – Present. **Evidence** – Multiple specimen reports. **Distribution** – American Sāmoa, Aunuʻu, Manuʻa Islands, Ofu, Ofu/Olosega, Olosega, Rose Atoll, Taʻū, Tutuila. **Nearest confirmed ecoregion** – Sāmoa, Tuvalu, and Tonga. **Vulnerability** – LC.

***Acroporaverweyi* Veron & Wallace, 1984 (288263)** ^CoTW CCW^

*Acroporaverweyi* cf. Veron & Wallace, 1984 (288263). ^CoTW CCW^ Reported – Mundy 1996. Referenced – Fisk and Birkeland 2002.

*Acroporaverweyi* Veron & Wallace, 1984 (288263). ^CoTW CCW^ Reported – Birkeland and Belliveau 2000; Craig et al. 2001; Fisk and Birkeland 2002; Birkeland et al. 2003; Coles et al. 2003; DiDonato et al. 2006; Birkeland 2007a; Corals NPAS 2016; Fenner 2018; QM 2018. Referenced – Green et al. 1999; Coles et al. 2003; DiDonato et al. 2006; Birkeland 2007a, 2007b; Lovell and McLardy 2008; Kenyon et al. 2011.

**American Sāmoa status** – Present. **Evidence** – Single specimen report (identified by C Wallace). **Distribution** – American Sāmoa, Aunuʻu, Manuʻa Islands, Ofu, Ofu/Olosega, Olosega, Tutuila. **Nearest confirmed ecoregion** – Sāmoa, Tuvalu, and Tonga. **Vulnerability** – VU.

######## Genus *Alveopora* Blainville, 1830

***Alveoporaallingi* Hoffmeister, 1925 (207192)** ^CoTW^

*Alveoporaallingi* Hoffmeister, 1925 (207192). ^CoTW^ Reported – Hoffmeister 1925; USACE 1980; Lamberts 1983; Maragos et al. 1994; Mundy 1996; Corals NPAS 2016; NMNH 2018. Referenced – Fisk and Birkeland 2002; Coles et al. 2003; DiDonato et al. 2006; Birkeland 2007b; Lovell and McLardy 2008; Kenyon et al. 2011.

**American Sāmoa status** – Present. **Evidence** – Type specimen location. **Distribution** – American Sāmoa, Manuʻa Islands, Tutuila. **Nearest confirmed ecoregion** – Sāmoa, Tuvalu, and Tonga. **Vulnerability** – VU. **Mesophotic record** – 31, 35 m depth (Hoffmeister 1925; Lamberts 1983). **Notes** – American Sāmoa is the type locality of this species.

***Alveoporatizardi* Bassett-Smith, 1890 (207195)** ^CoTW^

*Alveoporatizardi* Bassett-Smith, 1890 (207195). ^CoTW^ Reported – Fenner 2018.

*Alveoporatizardi* cf. Bassett-Smith, 1890 (207195). ^CoTW^ Reported – DMWR 2018.

**American Sāmoa status** – Present. **Evidence** – Single photographic record. **Distribution** – American Sāmoa, Tutuila. **Nearest confirmed ecoregion** – Vanuatu. **Vulnerability** – LC. **Notes** – This species has skeletal features very similar to *A.excelsa*, but is nodular instead of horizontal branches.

***Alveoporaverrilliana* Dana, 1846 (207201)** ^CoTW^

*Alveoporaverriliana* Dana, 1846 (207201) [sic]. Reported – USACE 1980.

*Alveoporaverrilliana* Dana, 1846 (207201). ^CoTW^ Reported – Hoffmeister 1925; Lamberts 1983; Kenyon et al. 2010; BPBM 2018; NMNH 2018. Referenced – Green et al. 1997; Lovell and McLardy 2008; Kenyon et al. 2011.

**American Sāmoa status** – Present. **Evidence** – Multiple specimen reports. **Distribution** – American Sāmoa, Rose Atoll, Taʻū, Tutuila. **Nearest confirmed ecoregion** – Sāmoa, Tuvalu, and Tonga. **Vulnerability** – VU.

***Alveoporaviridis* Quoy & Gaimard, 1833 (207203)** ^CoTW^

*Alveoporavirdis* Quoy & Gaimard, 1833 (207203) [sic]. Reported – Birkeland et al. 2003.

*Alveoporaviridis* Quoy & Gaimard, 1833 (207203). ^CoTW^ Reported – Lamberts 1983; Birkeland et al. 1987. Referenced – Green et al. 1999; Coles et al. 2003; Birkeland 2007b.

**American Sāmoa status** – Present. **Evidence** – Single specimen report (identified by A Lamberts). **Distribution** – Aunuʻu, Tutuila. **Nearest confirmed ecoregion** – Caroline Islands, Micronesia. **Geographical range extension** – Southeast. **Vulnerability** – NT.

######## Genus *Astreopora* Blainville, 1830

***Astreoporacucullata* Lamberts, 1980 (287943)** ^CoTW^

*Astreoporacuccullata* Lamberts, 1980 (287943) [sic]. Reported – BPBM 2018.

*Astreoporacucullata* Lamberts, 1980 (287943). ^CoTW^ Reported – USACE 1980; Lamberts 1983; Maragos et al. 1994; Fisk and Birkeland 2002; Coles et al. 2003; Birkeland 2007a; Kenyon et al. 2010; Corals NPAS 2016; DMWR 2018; Fenner 2018. Referenced – Birkeland 2007b; Lovell and McLardy 2008; Kenyon et al. 2011.

**American Sāmoa status** – Present. **Evidence** – Type specimen location. **Distribution** – American Sāmoa, Manuʻa Islands, Ofu, Ofu/Olosega, Olosega, Rose Atoll, Taʻū, Tutuila. **Nearest confirmed ecoregion** – Sāmoa, Tuvalu, and Tonga. **Vulnerability** – VU. **Notes** – American Sāmoa is the type locality of this species.

***Astreoporagracilis* Bernard, 1896 (207124)** ^CoTW^

*Astreoporagracilis* Bernard, 1896 (207124). ^CoTW^ Reported – Birkeland et al. 1987; Fisk and Birkeland 2002; Birkeland 2007a; Corals NPAS 2016; DMWR 2018; Fenner 2018. Referenced – DiDonato et al. 2006; Birkeland 2007a, 2007b; Lovell and McLardy 2008.

*Astreoporagracilis* cf. Bernard, 1896 (207124). ^CoTW^ Reported – Mundy 1996. Referenced – Fisk and Birkeland 2002.

*Astreoporagracillis* Bernard, 1896 (207124) [sic]. Reported – Birkeland et al. 2003.

**American Sāmoa status** – Present. **Evidence** – Single specimen report (identified by D Fenner). **Distribution** – American Sāmoa, Aunuʻu, Manuʻa Islands, Ofu, Ofu/Olosega, Olosega, Taʻū, Tutuila. **Nearest confirmed ecoregion** – Sāmoa, Tuvalu, and Tonga. **Vulnerability** – LC.

***Astreoporalisteri* Bernard, 1896 (207125)** ^CoTW^

*Astreoporalistera* Bernard, 1896 (207125) [sic]. Reported – Corals NPAS 2016.

*Astreoporalisteri* Bernard, 1896 (207125). ^CoTW^ Reported – USACE 1980; Lamberts 1983; Maragos et al. 1994; Mundy 1996; Green and Hunter 1998; Fisk and Birkeland 2002; Coles et al. 2003; Birkeland 2007a; Fenner 2018; Montgomery et al. 2019. Referenced – Fisk and Birkeland 2002; DiDonato et al. 2006; Birkeland 2007a, 2007b; Lovell and McLardy 2008.

**American Sāmoa status** – Present. **Evidence** – Multiple specimen reports. **Distribution** – American Sāmoa, Aunuʻu, Manuʻa Islands, Ofu, Ofu/Olosega, Olosega, Rose Atoll, Taʻū, Tutuila. **Nearest confirmed ecoregion** – Sāmoa, Tuvalu, and Tonga. **Vulnerability** – LC. **Mesophotic record** – 53 m depth (Montgomery et al. 2019).

***Astreoporamyriophthalma* (Lamarck, 1816) (207128)** ^CoTW^

*Astrcoporamyriopthalma* (Lamarck, 1816) (207128) [sic]. Reported – Hunter et al. 1993.

*Astreoporaeliptica* Yabe & Sugiyama, 1941 (430659) heterotypic synonym. Reported – DMWR 2018. Referenced – Coles et al. 2003.

*Astreoporaelliptica* Yabe & Sugiyama, 1941 (430659) [sic] heterotypic synonym. Reported – Birkeland et al. 1987; Maragos et al. 1994; Corals NPAS 2016; Fenner 2018. Referenced – DiDonato et al. 2006; Birkeland 2007a, 2007b; Lovell and McLardy 2008.

*Astreoporamicrophthalma* (Lamarck, 1816) (207128) [sic]. Reported – Fenner et al. 2008.

*Astreoporamyriophthalma /listeri* (Lamarck, 1816) (207128). Reported – DMWR 2018.

*Astreoporamyriophthalma /suggesta* (Lamarck, 1816) (207128). Reported – DMWR 2018.

*Astreoporamyriophthalma* (Lamarck, 1816) (207128). ^CoTW^ Reported – USACE 1980; Lamberts 1983; Birkeland et al. 1987, 2003; Maragos et al. 1994; Craig et al. 2001; Fisk and Birkeland 2002; Coles et al. 2003; DiDonato et al. 2006; Birkeland 2007a; Kenyon et al. 2010; CRED 2011; Corals NPAS 2016; DMWR 2018; Fenner 2018. Referenced – Fisk and Birkeland 2002; Coles et al. 2003; DiDonato et al. 2006; Birkeland 2007a, 2007b; Lovell and McLardy 2008.

*Astreoporamyriopthalma* (Lamarck, 1816) (207128) [sic]. Reported – Hunter et al. 1993; Green and Hunter 1998.

*Astreoporaprofunda* Verrill, 1872 (207126) heterotypic synonym. Reported – Hoffmeister 1925; Lamberts 1983; NMNH 2018.

**American Sāmoa status** – Present. **Evidence** – Multiple specimen reports. **Distribution** – American Sāmoa, Aunuʻu, Manuʻa Islands, Ofu, Ofu/Olosega, Olosega, Rose Atoll, South Bank, Taʻū, Tutuila. **Nearest confirmed ecoregion** – Sāmoa, Tuvalu, and Tonga. **Vulnerability** – LC.

***Astreoporarandalli* Lamberts, 1980 (207127)** ^CoTW^

*Astreoporarandalli* Lamberts, 1980 (207127). ^CoTW^ Reported – Birkeland et al. 1987, 2003; Coles et al. 2003; CRED 2011; Corals NPAS 2016; DMWR 2018; Fenner 2018; Montgomery et al. 2019. Referenced – Coles et al. 2003; DiDonato et al. 2006; Birkeland 2007b; Lovell and McLardy 2008.

**American Sāmoa status** – Present. **Evidence** – Multiple specimen reports. **Distribution** – American Sāmoa, Aunuʻu, Taʻū, Tutuila. **Nearest confirmed ecoregion** – Sāmoa, Tuvalu, and Tonga. **Vulnerability** – LC. **Mesophotic record** – 42 m depth (Montgomery et al. 2019).

***Astreoporascabra* Lamberts, 1982 (430660)** ^CoTW^

*Astreoporascabra* Lamberts, 1982 (430660). ^CoTW^ Reported – Lamberts 1983.

**American Sāmoa status** – Present. **Evidence** – Single specimen report (identified by A Lamberts). **Distribution** – Tutuila. **Nearest confirmed ecoregion** – Milne Bay, Papua New Guinea. **Geographical range extension** – East. **Vulnerability** – LC.

***Astreoporasuggesta* Wells, 1954 (287948)** ^CoTW^

*Astreoporasuggesta* Wells, 1954 (287948). ^CoTW^ Reported – Montgomery et al. 2019.

**American Sāmoa status** – Present. **Evidence** – Single photographic record. **Distribution** – Tutuila. **Nearest confirmed ecoregion** – Fiji. **Geographical range extension** – East. **Vulnerability** – LC. **Mesophotic record** – 46 m depth (Montgomery et al. 2019).

######## Genus *Isopora* Studer, 1879

***Isoporabrueggemanni* (Brook, 1893) (730688)** ^CoTW CCW^

*Acroporabrueggemanni* (Brook, 1893) (207067) homotypic synonym. Reported – Lamberts 1983; Maragos et al. 1994; Birkeland 2007a. Referenced – Birkeland 2007b.

*Acroporabruggemanni* (Brook, 1893) (207067) [sic] homotypic synonym. Reported – USACE 1980.

*Acroporabruggemanni* cf. (Brook, 1893) (207067) [sic] homotypic synonym. Reported – USACE 1980.

*Isoporabrueggemanni* (Brook, 1893) (730688). ^CoTW CCW^ Reported – Kenyon et al. 2010.

**American Sāmoa status** – Present. **Evidence** – Single specimen report (identified by A Lamberts). **Distribution** – American Sāmoa, Ofu, Rose Atoll, Taʻū, Tutuila. **Nearest confirmed ecoregion** – Sāmoa, Tuvalu, and Tonga. **Vulnerability** – VU. **Notes** – Live colonies are fairly distinctive.

***Isoporacrateriformis* (Gardiner, 1898) (730691)** ^CoTW CCW^

*Acroporacarterformis* (Gardiner, 1898) (288193) [sic] homotypic synonym. Reported – Birkeland et al. 2003.

*Acroporacarteriformis* (Gardiner, 1898) (288193) [sic] homotypic synonym. Reported – Birkeland et al. 2003.

*Acroporacraterformis* (Gardiner, 1898) (288193) [sic] homotypic synonym. Reported – Birkeland et al. 2003; Coles et al. 2003.

*Acroporacrateriformis* (Gardiner, 1898) (288193) homotypic synonym. Reported – Hoffmeister 1925; USACE 1980; Lamberts 1983; Birkeland et al. 1987, 2003; Itano and Buckley 1988; Mundy 1996; Craig et al. 2001; Fisk and Birkeland 2002; Coles et al. 2003; DiDonato et al. 2006; Birkeland 2007a; Fenner et al. 2008; Bare et al. 2010; Corals NPAS 2016; BPBM 2018. Referenced – Green et al. 1999; Fisk and Birkeland 2002; Coles et al. 2003; DiDonato et al. 2006; Birkeland 2007a, 2007b; Lovell and McLardy 2008.

*Isoporacrateriformis* (Gardiner, 1898) (730691). ^CoTW CCW^ Reported – CRED 2011; Fenner and Sudek 2016; DMWR 2018; Fenner 2018; NMNH 2018; QM 2018; Paulay and Brown 2019. Referenced – Kenyon et al. 2011.

**American Sāmoa status** – Present. **Evidence** – Multiple specimen reports. **Distribution** – American Sāmoa, Aunuʻu, Manuʻa Islands, Ofu, Ofu/Olosega, Olosega, Taʻū, Tutuila. **Nearest confirmed ecoregion** – Sāmoa, Tuvalu, and Tonga. **Vulnerability** – 𝒯, VU. **Notes** – Wallace (1999) and Veron (2000) note that the only difference between this species and *Isoporacuneata* (Dana, 1846) is colony shape. *Isoporacrateriformis* is encrusting while *I.cuneata* is cuneate to branching. Fenner has found only the encrusting/plate shape of *I.crateriformis* in American Sāmoa. *Isoporacrateriformis* is abundant to dominant in shallow reef slopes on southwest Tutuila.

***Isoporapalifera* (Lamarck, 1816) (730686)** ^CoTW CCW^

*Acroporapaiifera* (Lamarck, 1816) (207037) [sic] homotypic synonym. Reported – Hunter et al. 1993.

*Acroporapalifera* (Lamarck, 1816) (207037) homotypic synonym. Reported – Hoffmeister 1925; USACE 1980; Lamberts 1983; Birkeland et al. 1987, 2003; Itano and Buckley 1988; Hunter et al. 1993; Maragos et al. 1994; Coles et al. 2003; Fenner et al. 2008; Corals NPAS 2016. Referenced – Green et al. 1999; Coles et al. 2003; DiDonato et al. 2006; Birkeland 2007a, 2007b; Lovell and McLardy 2008.

*Isoporapalifera* (Lamarck, 1816) (730686). ^CoTW CCW^ Reported – Kenyon et al. 2010; CRED 2011; DMWR 2018; Fenner 2018; NMNH 2018; QM 2018.

**American Sāmoa status** – Present. **Evidence** – Multiple specimen reports. **Distribution** – American Sāmoa, Ofu, Ofu/Olosega, Rose Atoll, Taʻū, Tutuila. **Nearest confirmed ecoregion** – Sāmoa, Tuvalu, and Tonga. **Vulnerability** – NT.

######## Genus *Montipora* Blainville, 1830

***Montiporaaequituberculata* Bernard, 1897 (207144)** ^CoTW^

*Montiporaaequituberculata* ? Bernard, 1897 (207144). ^CoTW^ Reported – Coles et al. 2003.

*Montiporaaequituberculata* Bernard, 1897 (207144). ^CoTW^ Reported – Maragos et al. 1994; Green and Hunter 1998; Fisk and Birkeland 2002; Coles et al. 2003; Birkeland 2007a; Kenyon et al. 2010; Corals NPAS 2016; Fenner 2018; Montgomery et al. 2019. Referenced – Fisk and Birkeland 2002; Coles et al. 2003; DiDonato et al. 2006; Birkeland 2007a, 2007b; Lovell and McLardy 2008.

*Montiporaaquituberculata* Bernard, 1897 (207144) [sic]. Reported – DMWR 2018.

*Montiporacomposita* Crossland, 1952 (759845) heterotypic synonym. Reported – USACE 1980; Lamberts 1983; Birkeland et al. 1987. Referenced – Birkeland 2007b.

**American Sāmoa status** – Present. **Evidence** – Single specimen report (identified by A Montgomery and D Fenner). **Distribution** – American Sāmoa, Aunuʻu, Ofu, Ofu/Olosega, Rose Atoll, Tutuila. **Nearest confirmed ecoregion** – Sāmoa, Tuvalu, and Tonga. **Vulnerability** – LC. **Mesophotic record** – 34 m depth (Montgomery et al. 2019).

***Montiporaberryi* Hoffmeister, 1925 (869368)**

*Montiporaberryi* Hoffmeister, 1925 (869368). Reported – Hoffmeister 1925; USACE 1980; Lamberts 1983; Birkeland et al. 1987, 2003; Coles et al. 2003; Corals NPAS 2016; NMNH 2018. Referenced – Green et al. 1999; Coles et al. 2003; DiDonato et al. 2006; Birkeland 2007b.

**American Sāmoa status** – Present. **Evidence** – Type specimen location. **Distribution** – American Sāmoa, Ofu, Tutuila. **Nearest confirmed ecoregion** – Not available. **Notes** – Veron et al. (2019) consider this a possible synonym of *Montiporainformis* Bernard, 1897.

***Montiporacaliculata* (Dana, 1846) (287696)** ^CoTW^

*Montiporacaliculata /foveolata* (Dana, 1846) (287696). Reported – DMWR 2018.

*Montiporacaliculata* (Dana, 1846) (287696). ^CoTW^ Reported – USACE 1980; Lamberts 1983; Birkeland et al. 1987, 2003; Maragos et al. 1994; Fisk and Birkeland 2002; DiDonato et al. 2006; Birkeland 2007a; Kenyon et al. 2010; CRED 2011; Corals NPAS 2016; DMWR 2018; Fenner 2018. Referenced – Green et al. 1999; Coles et al. 2003; DiDonato et al. 2006; Birkeland 2007a, 2007b; Lovell and McLardy 2008; Kenyon et al. 2011.

**American Sāmoa status** – Present. **Evidence** – Multiple specimen reports. **Distribution** – American Sāmoa, Aunuʻu, Manuʻa Islands, Ofu, Ofu/Olosega, Olosega, Rose Atoll, Taʻū, Tutuila. **Nearest confirmed ecoregion** – Sāmoa, Tuvalu, and Tonga. **Vulnerability** – VU.

***Montiporacapitata* (Dana, 1846) (287697)** ^CoTW^

*Montiporacapitata* (Dana, 1846) (287697). ^CoTW^ Reported – Corals NPAS 2016; Montgomery et al. 2019.

**American Sāmoa status** – Present. **Evidence** – Single specimen report (identified by A Montgomery and D Fenner). **Distribution** – American Sāmoa, Tutuila. **Nearest confirmed ecoregion** – Fiji and Kiribati, south-east Line Islands. **Geographical range extension** – Between two disjunct ecoregions although Veron et al. (2019) strongly predicted the presence of this species in the Sāmoa, Tuvalu, and Tonga ecoregion. **Vulnerability** – NT. **Mesophotic record** – 49 m depth (Montgomery et al. 2019). **Notes** – While its presence is supported by a sample, more analysis should be done by a comparison with samples from Hawaii. Also, see note for *Montiporaverrucosa* (Lamarck, 1816).

***Montiporaefflorescens* Bernard, 1897 (207163)** ^CoTW^

*Montiporaefflorescens* ? Bernard, 1897 (207163). ^CoTW^ Reported – DMWR 2018.

*Montiporaefflorescens* Bernard, 1897 (207163). ^CoTW^ Reported – Mundy 1996; Birkeland and Belliveau 2000; Craig et al. 2001; Fisk and Birkeland 2002; DiDonato et al. 2006; Birkeland 2007a; Fenner et al. 2008; Kenyon et al. 2010; Corals NPAS 2016. Referenced – Fisk and Birkeland 2002; DiDonato et al. 2006; Birkeland 2007a, 2007b; Lovell and McLardy 2008.

*Montiporatrabeculata* Bernard, 1897 (759819) heterotypic synonym. Reported – Hoffmeister 1925; USACE 1980; Lamberts 1983; NMNH 2018. Referenced – Green et al. 1997.

**American Sāmoa status** – Present. **Evidence** – Multiple specimen reports. **Distribution** – American Sāmoa, Aunuʻu, Manuʻa Islands, Ofu, Olosega, Rose Atoll, Taʻū, Tutuila. **Nearest confirmed ecoregion** – Sāmoa, Tuvalu, and Tonga. **Vulnerability** – NT. **Notes** – This species is difficult to identify.

***Montiporaehrenbergi* Verrill, 1872 (207155)**

*Montiporaehrenbergi* Verrill, 1872 (207155). Reported – USACE 1980.

*Montiporaehrenbergii* Verrill, 1872 (207155) [sic]. Reported – Hoffmeister 1925; Lamberts 1983; Birkeland et al. 1987, 2003; Maragos et al. 1994; Green et al. 1997; Coles et al. 2003; Kenyon et al. 2010; Corals NPAS 2016; NMNH 2018. Referenced – Green et al. 1997, 1999; Coles et al. 2003; DiDonato et al. 2006; Birkeland 2007a, 2007b.

**American Sāmoa status** – Present. **Evidence** – Multiple specimen reports. **Distribution** – American Sāmoa, Aunuʻu, Ofu, Ofu/Olosega, Taʻū, Tutuila. **Nearest confirmed ecoregion** – Not available. **Notes** – Veron et al. (2019) believe this species is a probable synonym of *Montiporahispida* (Dana, 1846).

***Montiporafoliosa* (Pallas, 1766) (207182)** ^CoTW^

*Montiporaacutata* Bernard, 1897 (759840) [sic] heterotypic synonym. Reported – Lamberts 1983.

*Montiporafoliosa* (Pallas, 1766) (207182). ^CoTW^ Reported – Birkeland et al. 1987; Maragos et al. 1994; Coles et al. 2003; Kenyon et al. 2010; Corals NPAS 2016. Referenced – Coles et al. 2003; DiDonato et al. 2006; Birkeland 2007a, 2007b; Lovell and McLardy 2008.

*Montiporapulcherrima* Bernard, 1897 (759835) heterotypic synonym. Reported – USACE 1980.

*Montiporapulcherrima* cf. Bernard, 1897 (759835) heterotypic synonym. Reported – Lamberts 1983.

*Montiporascutata* Bernard, 1897 (759840) heterotypic synonym. Reported – USACE 1980.

**American Sāmoa status** – Present. **Evidence** – Single specimen report (synonym *Montiporaacutata* identified by A Lamberts). **Distribution** – American Sāmoa, Ofu, Ofu/Olosega, Rose Atoll, Tutuila. **Nearest confirmed ecoregion** – Sāmoa, Tuvalu, and Tonga. **Vulnerability** – NT. **Mesophotic record** – 30 m depth (Lamberts 1983).

***Montiporafoveolata* (Dana, 1846) (207133)** ^CoTW^

*Montiporafoveolata* (Dana, 1846) (207133). ^CoTW^ Reported – USACE 1980; Lamberts 1983; Birkeland et al. 1987; Maragos et al. 1994; Mundy 1996; Green and Hunter 1998; Birkeland and Belliveau 2000; Craig et al. 2001; Fisk and Birkeland 2002; Birkeland 2007a; Fenner et al. 2008; Kenyon et al. 2010; CRED 2011; Corals NPAS 2016; DMWR 2018. Referenced – Green et al. 1999; Fisk and Birkeland 2002; Coles et al. 2003; DiDonato et al. 2006; Birkeland 2007a, 2007b; Lovell and McLardy 2008.

*Montiporafoveolata* cf. (Dana, 1846) (207133). ^CoTW^ Reported – Hunter et al. 1993.

*Montiporasocialis* Bernard, 1897 (207173) heterotypic synonym. Reported – USACE 1980; Lamberts 1983; Birkeland et al. 1987; Coles et al. 2003; Corals NPAS 2016. Referenced – Coles et al. 2003; DiDonato et al. 2006; Birkeland 2007b.

*Montiporasocislis* Bernard, 1897 (207173) [sic] heterotypic synonym. Reported – Birkeland et al. 1987.

**American Sāmoa status** – Present. **Evidence** – Multiple specimen reports. **Distribution** – American Sāmoa, Aunuʻu, Manuʻa Islands, Ofu, Ofu/Olosega, Olosega, Rose Atoll, Taʻū, Tutuila. **Nearest confirmed ecoregion** – Sāmoa, Tuvalu, and Tonga. **Vulnerability** – NT.

***Montiporagrisea* Bernard, 1897 (287709)** ^CoTW^

*Montiporagrisea* Bernard, 1897 (287709). ^CoTW^ Reported – Mundy 1996; Green et al. 1997; Mundy and Green 1999; Fisk and Birkeland 2002; Birkeland et al. 2003; Coles et al. 2003; DiDonato et al. 2006; Birkeland 2007a; Fenner et al. 2008; Corals NPAS 2016; Fenner 2018; Montgomery et al. 2019. Referenced – Green et al. 1999; Fisk and Birkeland 2002; Coles et al. 2003; DiDonato et al. 2006; Birkeland 2007a, 2007b; Lovell and McLardy 2008.

**American Sāmoa status** – Present. **Evidence** – Multiple photographic records. **Distribution** – American Sāmoa, Aunuʻu, Manuʻa Islands, Ofu, Ofu/Olosega, Olosega, Rose Atoll, Swains, Taʻū, Tutuila. **Nearest confirmed ecoregion** – Sāmoa, Tuvalu, and Tonga. **Vulnerability** – LC. **Mesophotic record** – 53 m depth (Montgomery et al. 2019).

***Montiporaincrassata* (Dana, 1846) (287714)** ^CoTW^

*Montiporaincrassata* (Dana, 1846) (287714). ^CoTW^ Reported – Fisk and Birkeland 2002; Kenyon et al. 2010; CRED 2011; Fenner 2018.

*Montiporaincrassita* cf. (Dana, 1846) (287714) [sic]. Reported – Montgomery et al. 2019.

**American Sāmoa status** – Present. **Evidence** – Single photographic record. **Distribution** – Manuʻa Islands, Olosega, Rose Atoll, Tutuila. **Nearest confirmed ecoregion** – Sāmoa, Tuvalu, and Tonga. **Vulnerability** – NT. **Mesophotic record** – 33 m depth (Montgomery et al. 2019).

***Montiporainformis* Bernard, 1897 (207186)** ^CoTW^

*Montiporainformis* Bernard, 1897 (207186). ^CoTW^ Reported – Birkeland et al. 1987; Maragos et al. 1994, 1995; Mundy 1996; Fisk and Birkeland 2002; DiDonato et al. 2006; Birkeland 2007a; Fenner et al. 2008; Kenyon et al. 2010; Corals NPAS 2016; DMWR 2018; Fenner 2018. Referenced – Fisk and Birkeland 2002; Coles et al. 2003; DiDonato et al. 2006; Birkeland 2007a, 2007b; Lovell and McLardy 2008.

*Montiporainformis* cf. Bernard, 1897 (207186). ^CoTW^ Reported – Hunter et al. 1993; DMWR 2018.

**American Sāmoa status** – Present. **Evidence** – Single specimen report (identified by D Fenner). **Distribution** – American Sāmoa, Aunuʻu, Manuʻa Islands, Ofu, Ofu/Olosega, Olosega, Rose Atoll, Taʻū, Tutuila. **Nearest confirmed ecoregion** – Sāmoa, Tuvalu, and Tonga. **Vulnerability** – LC.

***Montiporamarshallensis* Wells, 1954 (1263761)**

*Montiporamarshallensis* Wells, 1954 (1263761). Reported – USACE 1980; Lamberts 1983; Birkeland et al. 1987; Corals NPAS 2016. Referenced – Coles et al. 2003; DiDonato et al. 2006; Birkeland 2007b.

**American Sāmoa status** – Present. **Evidence** – Single specimen report (identified by A Lamberts). **Distribution** – American Sāmoa, Tutuila. **Nearest confirmed ecoregion** – Not available. **Notes** – Veron et al. (2019) believe this name is probably synonymous with *Montiporacrassituberculata* Bernard, 1897. It has otherwise not been reported from American SāmoA Lamberts (1983) reported this species to be rare and Birkeland et al. (1987) reported this species from a single site in 1979.

***Montiporaspumosa* (Lamarck, 1816) (207138)** ^CoTW^

*Montiporaspumosa* (Lamarck, 1816) (207138). ^CoTW^ Reported – USACE 1980; Lamberts 1983; Maragos et al. 1994; Green and Hunter 1998; Birkeland et al. 2003; Kenyon et al. 2010. Referenced – Birkeland 2007b; Lovell and McLardy 2008.

**American Sāmoa status** – Present. **Evidence** – Single specimen report (identified by A Lamberts). **Distribution** – American Sāmoa, Rose Atoll, Tutuila. **Nearest confirmed ecoregion** – Sāmoa, Tuvalu, and Tonga. **Vulnerability** – LC.

***Montiporatuberculosa* (Lamarck, 1816) (207156)** ^CoTW^

*Montiporatuberculosa* (Lamarck, 1816) (207156). ^CoTW^ Reported – Hoffmeister 1925; USACE 1980; Lamberts 1983; Birkeland et al. 1987, 2003; Hunter et al. 1993; Maragos et al. 1994, 1995; Mundy 1996; Craig et al. 2001; Fisk and Birkeland 2002; Coles et al. 2003; DiDonato et al. 2006; Birkeland 2007a; Fenner et al. 2008; Kenyon et al. 2010; Corals NPAS 2016; DMWR 2018; Fenner 2018; NMNH 2018; Montgomery et al. 2019. Referenced – Green et al. 1999; Fisk and Birkeland 2002; Coles et al. 2003; DiDonato et al. 2006; Birkeland 2007a, 2007b; Lovell and McLardy 2008.

**American Sāmoa status** – Present. **Evidence** – Multiple specimen reports. **Distribution** – American Sāmoa, Aunuʻu, Manuʻa Islands, Ofu, Ofu/Olosega, Olosega, Rose Atoll, Taʻū, Tutuila. **Nearest confirmed ecoregion** – Sāmoa, Tuvalu, and Tonga. **Vulnerability** – LC. **Mesophotic record** – 49 m depth (Montgomery et al. 2019). **Notes** – The photograph of this species reported in Corals NPAS (2016) is too blurry for identification, and seems unlikely to be correct.

***Montiporaturgescens* Bernard, 1897 (207142)** ^CoTW^

*Montiporaturgescens* ? Bernard, 1897 (207142). ^CoTW^ Reported – Coles et al. 2003.

*Montiporaturgescens* Bernard, 1897 (207142). ^CoTW^ Reported – Mundy 1996; Green and Hunter 1998; Mundy and Green 1999; Craig et al. 2001; Birkeland et al. 2003; Coles et al. 2003; Birkeland 2007a; Fenner et al. 2008; CRED 2011; Corals NPAS 2016; DMWR 2018; Fenner 2018. Referenced – Green et al. 1999; Fisk and Birkeland 2002; Coles et al. 2003; DiDonato et al. 2006; Birkeland 2007a, 2007b; Lovell and McLardy 2008.

**American Sāmoa status** – Present. **Evidence** – Single specimen report (identified by D Fenner). **Distribution** – American Sāmoa, Aunuʻu, Manuʻa Islands, Ofu, Ofu/Olosega, Olosega, Swains, Taʻū, Tutuila. **Nearest confirmed ecoregion** – Fiji. **Geographical range extension** – East although Veron et al. (2019) strongly predicted the presence of this species in the Sāmoa, Tuvalu, and Tonga ecoregion. **Vulnerability** – LC.

***Montiporaturtlensis* Veron & Wallace, 1984 (287731)** ^CoTW^

*Montiporaturtlensis* Veron & Wallace, 1984 (287731). ^CoTW^ Reported – Work and Rameyer 2002; DiDonato et al. 2006; Birkeland 2007a; Corals NPAS 2016; Fenner 2018. Referenced – Lovell and McLardy 2008.

**American Sāmoa status** – Present. **Evidence** – Multiple photographic records. **Distribution** – American Sāmoa, Ofu, Ofu/Olosega, Tutuila. **Nearest confirmed ecoregion** – Sāmoa, Tuvalu, and Tonga. **Vulnerability** – VU.

***Montiporavaughani* Hoffmeister, 1925 (430668)**

*Montiporavaughani* Hoffmeister, 1925 (430668). Reported – Hoffmeister 1925; Lamberts 1983; Fenner 2018; NMNH 2018.

**American Sāmoa status** – Present. **Evidence** – Type specimen location. **Distribution** – American Sāmoa, Tutuila. **Nearest confirmed ecoregion** – Not available. **Vulnerability** – DD. **Notes** – This species is easily identified but has one difference with *M.foveolata*, i.e., rows of corallites that are closer together within rows and farther apart between rows. However, some colonies have areas like this and other areas like *M.foveolata*. Veron et al. (2019) believe this species is probable synonym of *M.foveolata*.

***Montiporavenosa* (Ehrenberg, 1834) (207139)** ^CoTW^

*Montiporvenosa* (Ehrenberg, 1834) (207139) [sic]. Reported – Birkeland et al. 2003.

*Montiporavenosa* (Ehrenberg, 1834) (207139). ^CoTW^ Reported – Hoffmeister 1925; USACE 1980; Lamberts 1983; Birkeland et al. 1987, 2003; Maragos et al. 1994; Green et al. 1997; Green and Hunter 1998; Birkeland and Belliveau 2000; Craig et al. 2001; Fisk and Birkeland 2002; DiDonato et al. 2006; Birkeland 2007a; Fenner et al. 2008; Kenyon et al. 2010; Corals NPAS 2016; Fenner 2018; NMNH 2018. Referenced – Green et al. 1997, 1999; Coles et al. 2003; DiDonato et al. 2006; Birkeland 2007a, 2007b; Lovell and McLardy 2008.

**American Sāmoa status** – Present. **Evidence** – Multiple specimen reports. **Distribution** – American Sāmoa, Aunuʻu, Manuʻa Islands, Ofu, Ofu/Olosega, Olosega, Rose Atoll, Taʻū, Tutuila. **Nearest confirmed ecoregion** – Sāmoa, Tuvalu, and Tonga. **Vulnerability** – NT.

***Montiporaverrilli* Vaughan, 1907 (207136)** ^CoTW^

*Montiporaverilli* Vaughan, 1907 (207136) [sic]. Reported – CRED 2011. Referenced – Lovell and McLardy 2008.

*Montiporaverrilliauaensis* Vaughan, 1907 (207136). Reported – NMNH 2018.

Montiporaverrillivar.auaensis Hoffmeister, 1925 (1262050). Reported – Hoffmeister 1925.

*Montiporaverrilli* cf. Vaughan, 1907 (207136). ^CoTW^ Referenced – Coles et al. 2003.

*Montiporaverrilli* Vaughan, 1907 (207136). ^CoTW^ Reported – Hoffmeister 1925; USACE 1980; Lamberts 1983; Birkeland et al. 1987, 2003; Maragos et al. 1994; Green et al. 1997; Coles et al. 2003; NMNH 2018. Referenced – Green et al. 1997, 1999; Coles et al. 2003; DiDonato et al. 2006; Birkeland 2007a, 2007b.

*Montiporaverrillii* Vaughan, 1907 (207136) [sic]. Reported – Corals NPAS 2016.

**American Sāmoa status** – Present. **Evidence** – Multiple specimen reports. **Distribution** – American Sāmoa, Aunuʻu, Ofu, Ofu/Olosega, Rose Atoll, Tutuila. **Nearest confirmed ecoregion** – Sāmoa, Tuvalu, and Tonga. **Vulnerability** – DD. **Notes** – There appears to be no reliable way to distinguish this species from *Montiporapatula* Verrill, 1870 in spite of claims (Fenner 2005). More analysis of this species and *M.patula* is needed to determine which species is valid. The latter has not been reported from American Sāmoa.

####### Family Agariciidae Gray, 1847

######## Genus *Gardineroseris* Scheer & Pillai, 1974

***Gardineroserisplanulata* (Dana, 1846) (207274)** ^CoTW^

*Gardi*n*eroseris plantuata* (Dana, 1846) (207274) [sic]. Reported – Birkeland et al. 2003.

*Gardineroserisplanulata* (Dana, 1846) (207274). ^CoTW^ Reported – Lamberts 1983; Birkeland et al. 1987, 2003; Maragos et al. 1994; Mundy 1996; Coles et al. 2003; DiDonato et al. 2006; Corals NPAS 2016; Fenner 2018; Montgomery et al. 2019. Referenced – Green et al. 1999; Fisk and Birkeland 2002; Coles et al. 2003; DiDonato et al. 2006; Birkeland 2007a, 2007b; Lovell and McLardy 2008.

*Gardineroserisponderosa* (Gardiner, 1905) (766561) heterotypic synonym. Reported – USACE 1980.

*Gardinoserisplanulata* (Dana, 1846) (207274) [sic]. Reported – Green and Hunter 1998; Fenner et al. 2008.

*Pavonaplanulata* cf. (Dana, 1846) (1263640) homotypic synonym. Reported – USACE 1980.

**American Sāmoa status** – Present. **Evidence** – Multiple specimen reports. **Distribution** – American Sāmoa, Aunuʻu, Manuʻa Islands, Ofu, Ofu/Olosega, Olosega, Tutuila. **Nearest confirmed ecoregion** – Sāmoa, Tuvalu, and Tonga. **Vulnerability** – LC. **Mesophotic record** – 49 m depth (Montgomery et al. 2019).

######## Genus *Leptoseris* Milne Edwards & Haime, 1849

***Leptoserisexplanata* Yabe & Sugiyama, 1941 (207289)** ^CoTW^

*Leptoserisexplanata* Yabe & Sugiyama, 1941 (207289). ^CoTW^ Reported – Maragos et al. 1994; Mundy 1996; Coles et al. 2003; Kenyon et al. 2010; DMWR 2018; Fenner 2018; Montgomery et al. 2019. Referenced – Fisk and Birkeland 2002; Coles et al. 2003; Birkeland 2007b; Lovell and McLardy 2008.

**American Sāmoa status** – Present. **Evidence** – Multiple specimen reports. **Distribution** – American Sāmoa, Manuʻa Islands, Taʻū, Tutuila. **Nearest confirmed ecoregion** – Sāmoa, Tuvalu, and Tonga. **Vulnerability** – LC. **Mesophotic record** – 46 m depth (Montgomery et al. 2019).

***Leptoserisfoliosa* Dinesen, 1980 (207286)** ^CoTW^

*Leptoserisfoliosa* ? Dinesen, 1980 (207286). ^CoTW^ Reported – DMWR 2018.

*Leptoserisfoliosa* Dinesen, 1980 (207286). ^CoTW^ Reported – Mundy 1996; Kenyon et al. 2010; DMWR 2018; Fenner 2018. Referenced – Birkeland 2007b; Lovell and McLardy 2008.

**American Sāmoa status** – Present. **Evidence** – Single specimen report (identified by D Fenner). **Distribution** – American Sāmoa, Rose Atoll, Tutuila. **Nearest confirmed ecoregion** – Sāmoa, Tuvalu, and Tonga. **Vulnerability** – LC.

***Leptoserisgardineri* (van der Horst, 1922) (207284)** ^CoTW^

*Leptoserisgardineri* (van der Horst, 1922) (207284). ^CoTW^ Reported – Hoffmeister 1925; USACE 1980; Lamberts 1983; BPBM 2018; DMWR 2018; NMNH 2018. Referenced – Coles et al. 2003; Lovell and McLardy 2008.

*Leptoserisgardineri* cf. (van der Horst, 1922) (207284). ^CoTW^ Reported – NMNH 2018.

**American Sāmoa status** – Present. **Evidence** – Multiple specimen reports. **Distribution** – American Sāmoa, Tutuila. **Nearest confirmed ecoregion** – Fiji. **Geographical range extension** – East although Veron et al. (2019) strongly predicted the presence of this species in the Sāmoa, Tuvalu, and Tonga ecoregion. **Vulnerability** – LC. **Mesophotic record** – 49, 50 m depth (Hoffmeister 1925; Lamberts 1983).

***Leptoserisincrustans* (Quelch, 1886) (207279)** ^CoTW^

*Leptoserisincrustans* (Quelch, 1886) (207279). ^CoTW^ Reported – Birkeland et al. 1987; Maragos et al. 1994; Coles et al. 2003; Kenyon et al. 2010; CRED 2011; Corals NPAS 2016; DMWR 2018; Fenner 2018. Referenced – DiDonato et al. 2006; Birkeland 2007a, 2007b; Lovell and McLardy 2008; Kenyon et al. 2011.

**American Sāmoa status** – Present. **Evidence** – Single specimen report (identified by D Fenner). **Distribution** – American Sāmoa, Ofu, Ofu/Olosega, Olosega, Rose Atoll, Tutuila. **Nearest confirmed ecoregion** – Sāmoa, Tuvalu, and Tonga. **Vulnerability** – VU.

***Leptoserismycetoseroides* Wells, 1954 (207283)** ^CoTW^

*Leptoserismycetoceroides* Wells, 1954 (207283) [sic]. Reported – Green and Hunter 1998.

*Leptoserismycetoseroides* cf. Wells, 1954 (207283). ^CoTW^ Referenced – Coles et al. 2003.

*Leptoserismycetoseroides* Wells, 1954 (207283). ^CoTW^ Reported – Birkeland et al. 1987; Maragos et al. 1994; Mundy 1996; Craig et al. 2001; Fisk and Birkeland 2002; Coles et al. 2003; Birkeland 2007a; Fenner et al. 2008; Kenyon et al. 2010; CRED 2011; Corals NPAS 2016; DMWR 2018; Fenner 2018. Referenced – Fisk and Birkeland 2002; Coles et al. 2003; DiDonato et al. 2006; Birkeland 2007a, 2007b; Lovell and McLardy 2008.

**American Sāmoa status** – Present. **Evidence** – Single specimen report (identified by D Fenner). **Distribution** – American Sāmoa, Aunuʻu, Ofu, Ofu/Olosega, Olosega, Rose Atoll, Swains, Taʻū, Tutuila. **Nearest confirmed ecoregion** – Sāmoa, Tuvalu, and Tonga. **Vulnerability** – LC.

***Leptoserisscabra* Vaughan, 1907 (207282)** ^CoTW^

*Leptoserisscabra* ? Vaughan, 1907 (207282). ^CoTW^ Reported – DMWR 2018.

*Leptoserisscabra* cf. Vaughan, 1907 (207282). ^CoTW^ Reported – Montgomery et al. 2019.

*Leptoserisscabra* Vaughan, 1907 (207282). ^CoTW^ Reported – Hoffmeister 1925; USACE 1980; Lamberts 1983; Maragos et al. 1994; Green and Hunter 1998; Coles et al. 2003; Kenyon et al. 2010; CRED 2011; DMWR 2018; Fenner 2018; NMNH 2018; Montgomery et al. 2019. Referenced – Coles et al. 2003; Birkeland 2007b; Lovell and McLardy 2008.

**American Sāmoa status** – Present. **Evidence** – Multiple specimen reports. **Distribution** – American Sāmoa, Aunuʻu, Rose Atoll, Tutuila. **Nearest confirmed ecoregion** – Sāmoa, Tuvalu, and Tonga. **Vulnerability** – LC. **Mesophotic record** – 30, 52 m depth (Lamberts 1983; Montgomery et al. 2019).

***Leptoserissolida* (Quelch, 1886) (207290)** ^CoTW^

*Leptoserissolida* (Quelch, 1886) (207290). ^CoTW^ Reported – NMNH 2018.

**American Sāmoa status** – Present. **Evidence** – Single specimen report (identified by D Luck). **Distribution** – Tutuila. **Nearest confirmed ecoregion** – Fiji and Society Islands, French Polynesia. **Geographical range extension** – Between two disjunct ecoregions. **Vulnerability** – LC.

***Leptoseristubulifera* Vaughan, 1907 (207288)** ^CoTW^

*Leptoseristubulifera* Vaughan, 1907 (207288). ^CoTW^ Reported – Montgomery et al. 2019.

**American Sāmoa status** – Present. **Evidence** – Single specimen report (identified by A Montgomery). **Distribution** – Tutuila. **Nearest confirmed ecoregion** – Fiji. **Geographical range extension** – East. **Vulnerability** – LC. **Mesophotic record** – 52 m depth (Montgomery et al. 2019).

***Leptoserisyabei* (Pillai & Scheer, 1976) (207287)** ^CoTW^

*Leptoserisyabei* (Pillai & Scheer, 1976) (207287). ^CoTW^ Reported – Maragos et al. 1994; Kenyon et al. 2010; Corals NPAS 2016; DMWR 2018; Fenner 2018. Referenced – DiDonato et al. 2006; Birkeland 2007a, 2007b; Lovell and McLardy 2008; Kenyon et al. 2011.

**American Sāmoa status** – Present. **Evidence** – Single specimen report (identified by D Fenner). **Distribution** – American Sāmoa, Ofu, Ofu/Olosega, Olosega, Rose Atoll, Tutuila. **Nearest confirmed ecoregion** – Sāmoa, Tuvalu, and Tonga. **Vulnerability** – VU.

######## Genus *Pavona* Lamarck, 1801

***Pavonabipartita* Nemenzo, 1979 (289199)** ^CoTW^

*Pavonabipartita* Nemenzo, 1979 (289199). ^CoTW^ Reported – Fenner 2018.

**American Sāmoa status** – Present. **Evidence** – Single photographic record. **Distribution** – Tutuila. **Nearest confirmed ecoregion** – Sāmoa, Tuvalu, and Tonga. **Vulnerability** – VU.

***Pavonachiriquiensis* Glynn, Maté & Stemann, 2001 (289200)** ^CoTW^

*Pavonachiriquensis* Glynn, Maté & Stemann, 2001 (289200) [sic]. Reported – Fenner and Sudek 2016; DMWR 2018; Fenner 2018.

*Pavonachiriquiensis* Glynn, Maté & Stemann, 2001 (289200). ^CoTW^ Reported – Kenyon et al. 2010; Montgomery et al. 2019.

**American Sāmoa status** – Present. **Evidence** – Multiple specimen reports. **Distribution** – American Sāmoa, Aunuʻu, Ofu/Olosega, Rose Atoll, Swains, Taʻū, Tutuila. **Nearest confirmed ecoregion** – Sāmoa, Tuvalu, and Tonga. **Vulnerability** – LC. **Mesophotic record** – 46 m depth (Montgomery et al. 2019). **Notes** – See note on *Pavonavarians* Verrill, 1864.

***Pavonaclavus* (Dana, 1846) (207318)** ^CoTW^

*Pavonaclavus* (Dana, 1846) (207318). ^CoTW^ Reported – USACE 1980; Lamberts 1983; Birkeland et al. 1987; Hunter et al. 1993; Maragos et al. 1994, 1995; Mundy 1996; Green and Hunter 1998; Coles et al. 2003; Kenyon et al. 2010; CRED 2011; Corals NPAS 2016. Referenced – Fisk and Birkeland 2002; Coles et al. 2003; DiDonato et al. 2006; Birkeland 2007a, 2007b; Lovell and McLardy 2008.

*Pavonalilacaea* (Klunzinger, 1879) (207297) [sic] heterotypic synonym. Reported – Birkeland et al. 1987.

**American Sāmoa status** – Present. **Evidence** – Single specimen report (identified by A Lamberts). **Distribution** – American Sāmoa, Aunuʻu, Manuʻa Islands, Ofu, Ofu/Olosega, Olosega, Rose Atoll, Swains, Taʻū, Tutuila. **Nearest confirmed ecoregion** – Sāmoa, Tuvalu, and Tonga. **Vulnerability** – LC. **Notes** – This is a distinctive species but corallites are near identical to *P.bipartita*.

***Pavonadecussata* (Dana, 1846) (207320)** ^CoTW^

*Pavonadecussata* (Dana, 1846) (207320). ^CoTW^ Reported – Mayor 1924a, 1924b; Hoffmeister 1925; USACE 1980; Lamberts 1983; Birkeland et al. 1987, 2003; Itano and Buckley 1988; Maragos et al. 1994; Mundy 1996; Green et al. 1997; Green and Hunter 1998; Birkeland and Belliveau 2000; Craig et al. 2001; Coles et al. 2003; Birkeland 2007a; Corals NPAS 2016; DMWR 2018; Fenner 2018; NMNH 2018. Referenced – Green et al. 1997; Coles et al. 2003; DiDonato et al. 2006; Birkeland 2007b; Lovell and McLardy 2008; Kenyon et al. 2011.

*Pavonadecussata* ? (Dana, 1846) (207320). ^CoTW^ Reported – NMNH 2018.

**American Sāmoa status** – Present. **Evidence** – Multiple specimen reports. **Distribution** – American Sāmoa, Ofu, Ofu/Olosega, Taʻū, Tutuila. **Nearest confirmed ecoregion** – Sāmoa, Tuvalu, and Tonga. **Vulnerability** – VU.

***Pavonadivaricata* (Lamarck, 1816) (207311)**

*Pavonadivaricata* (Lamarck, 1816) (207311). Reported – Mayor 1924a, 1924b; Hoffmeister 1925; USACE 1980; Lamberts 1983; Birkeland et al. 1987, 2003, 2013; Hunter et al. 1993; Maragos et al. 1994, 1995; Mundy 1996; Green et al. 1997; Birkeland and Belliveau 2000; Craig et al. 2001; Fisk and Birkeland 2002; Coles et al. 2003; DiDonato et al. 2006; Birkeland 2007a; Fenner et al. 2008; Corals NPAS 2016; BPBM 2018; NMNH 2018. Referenced – Green et al. 1997, 1999; Fisk and Birkeland 2002; Coles et al. 2003; DiDonato et al. 2006; Birkeland 2007a, 2007b; Lovell and McLardy 2008.

**American Sāmoa status** – Present. **Evidence** – Multiple specimen reports. **Distribution** – American Sāmoa, Manuʻa Islands, Ofu, Olosega, Sāmoa Islands, Tutuila. **Nearest confirmed ecoregion** – Not available. **Notes** – The coral in the photograph of this species reported in Corals NPAS (2016) appears to be incorrectly identified and should be *Pavonafrondifera* (Lamarck, 1816). Veron et al. (2019) consider *Pavoniadivaricata* Lamarck, 1816 as a synonym of *P.frondifera*.

***Pavonaduerdeni* Vaughan, 1907 (207315)** ^CoTW^

*Pavonaduerdeni* Vaughan, 1907 (207315). ^CoTW^ Reported – USACE 1980; Lamberts 1983; Birkeland et al. 1987, 2003; Fisk and Birkeland 2002; Coles et al. 2003; CRED 2011; Corals NPAS 2016; DMWR 2018; Fenner 2018; NMNH 2018. Referenced – Green et al. 1999; Coles et al. 2003; Birkeland 2007b; Lovell and McLardy 2008.

**American Sāmoa status** – Present. **Evidence** – Multiple specimen reports. **Distribution** – American Sāmoa, Aunuʻu, Manuʻa Islands, Ofu/Olosega, Rose Atoll, Swains, Taʻū, Tutuila. **Nearest confirmed ecoregion** – Sāmoa, Tuvalu, and Tonga. **Vulnerability** – LC. **Mesophotic record** – 30 m depth (Lamberts 1983).

***Pavonaexplanulata* (Lamarck, 1816) (207306)** ^CoTW^

*Pavonaexplanata* (Lamarck, 1816) (207306) [sic]. Referenced – Coles et al. 2003.

*Pavonaexplanulata* (Lamarck, 1816) (207306). ^CoTW^ Reported – Birkeland et al. 1987; Maragos et al. 1994; Mundy 1996; Green and Hunter 1998; Coles et al. 2003; DiDonato et al. 2006; Kenyon et al. 2010; CRED 2011; Corals NPAS 2016; DMWR 2018; Fenner 2018. Referenced – Fisk and Birkeland 2002; DiDonato et al. 2006; Birkeland 2007a, 2007b; Lovell and McLardy 2008.

*Pavonaexplanulata* cf. (Lamarck, 1816) (207306). ^CoTW^ Reported – Maragos et al. 1994.

**American Sāmoa status** – Present. **Evidence** – Single specimen report (identified by D Fenner). **Distribution** – American Sāmoa, Aunuʻu, Manuʻa Islands, Ofu, Ofu/Olosega, Olosega, Rose Atoll, Taʻū, Tutuila. **Nearest confirmed ecoregion** – Sāmoa, Tuvalu, and Tonga. **Vulnerability** – LC.

***Pavonafrondifera* (Lamarck, 1816) (207307)** ^CoTW^

*Pavonafrondifera* (Lamarck, 1816) (207307). ^CoTW^ Reported – Mayor 1924a, 1924b; Hoffmeister 1925; USACE 1980; Lamberts 1983; Maragos et al. 1994; Fisk and Birkeland 2002; DiDonato et al. 2006; Corals NPAS 2016; Fenner and Sudek 2016; DMWR 2018; Fenner 2018; NMNH 2018. Referenced – Dahl and Lamberts 1977; Dahl 1981; Green et al. 1997; Coles et al. 2003; Birkeland 2007b; Lovell and McLardy 2008.

**American Sāmoa status** – Present. **Evidence** – Multiple specimen reports. **Distribution** – American Sāmoa, Manuʻa Islands, Ofu, Ofu/Olosega, Olosega, Tutuila. **Nearest confirmed ecoregion** – Sāmoa, Tuvalu, and Tonga. **Vulnerability** – LC.

***Pavonagigantea* Verrill, 1869 (289201)** ^CoTW^

*Pavonagigantea* cf. Verrill, 1869 (289201). ^CoTW^ Reported – Lamberts 1983.

*Pavonagigantea* Verrill, 1869 (289201). ^CoTW^ Reported – USACE 1980; DMWR 2018; Fenner 2018.

**American Sāmoa status** – Present. **Evidence** – Multiple specimen reports. **Distribution** – American Sāmoa, Aunuʻu, Tutuila. **Nearest confirmed ecoregion** – Marshall Islands and Galapagos Islands. **Geographical range extension** – Between two disjunct ecoregions, significant geographical range extension. **Vulnerability** – LC. **Mesophotic record** – 30 m depth (Lamberts 1983).

***Pavonamaldivensis* (Gardiner, 1905) (207309)** ^CoTW^

*Pavonamaldivensis* (Gardiner, 1905) (207309). ^CoTW^ Reported – USACE 1980; Lamberts 1983; Birkeland et al. 1987; Maragos et al. 1994; Mundy 1996; Fisk and Birkeland 2002; Coles et al. 2003; Fenner et al. 2008; Kenyon et al. 2010; CRED 2011; Corals NPAS 2016; DMWR 2018; Fenner 2018. Referenced – Green et al. 1999; Fisk and Birkeland 2002; Coles et al. 2003; DiDonato et al. 2006; Birkeland 2007a, 2007b; Lovell and McLardy 2008.

*Pavonapollicata* Wells, 1954 (207299) heterotypic synonym. Reported – BPBM 2018; NMNH 2018.

**American Sāmoa status** – Present. **Evidence** – Multiple specimen reports. **Distribution** – American Sāmoa, Aunuʻu, Manuʻa Islands, Ofu, Ofu/Olosega, Olosega, Rose Atoll, Swains, Taʻū, Tutuila. **Nearest confirmed ecoregion** – Sāmoa, Tuvalu, and Tonga. **Vulnerability** – LC.

***Pavonaminuta* Wells, 1954 (207317)** ^CoTW^

*Pavonaminuta* Wells, 1954 (207317). ^CoTW^ Reported – Birkeland et al. 1987; Maragos et al. 1994; Mundy 1996; Craig et al. 2001; Work and Rameyer 2002; Coles et al. 2003; Birkeland 2007a; Kenyon et al. 2010; CRED 2011; Corals NPAS 2016; DMWR 2018; Fenner 2018. Referenced – Fisk and Birkeland 2002; DiDonato et al. 2006; Birkeland 2007a, 2007b; Lovell and McLardy 2008.

**American Sāmoa status** – Present. **Evidence** – Single specimen report (identified by D Fenner). **Distribution** – American Sāmoa, Aunuʻu, Manuʻa Islands, Ofu, Ofu/Olosega, Olosega, Rose Atoll, Taʻū, Tutuila. **Nearest confirmed ecoregion** – Sāmoa, Tuvalu, and Tonga. **Vulnerability** – NT.

***Pavonavarians* Verrill, 1864 (207303)** ^CoTW^

*Pavonavarians* aff. Verrill, 1864 (207303). ^CoTW^ Reported – Coles et al. 2003.

*Pavonavarians* cf. Verrill, 1864 (207303). ^CoTW^ Reported – Maragos et al. 1994.

*Pavonavarians* Verrill, 1864 (207303). ^CoTW^ Reported – USACE 1980; Lamberts 1983; Birkeland et al. 1987, 2003; Hunter et al. 1993; Maragos et al. 1994, 1995; Mundy 1996; Green and Hunter 1998; Birkeland and Belliveau 2000; Craig et al. 2001; Fisk and Birkeland 2002; Coles et al. 2003; DiDonato et al. 2006; Birkeland 2007a; Fenner et al. 2008; Kenyon et al. 2010; CRED 2011; Corals NPAS 2016; Fenner and Sudek 2016; DMWR 2018; Fenner 2018; NMNH 2018; Montgomery et al. 2019. Referenced – Green et al. 1999; Fisk and Birkeland 2002; Coles et al. 2003; DiDonato et al. 2006; Birkeland 2007a, 2007b; Lovell and McLardy 2008.

**American Sāmoa status** – Present. **Evidence** – Multiple specimen reports. **Distribution** – American Sāmoa, Aunuʻu, Manuʻa Islands, Ofu, Ofu/Olosega, Olosega, Rose Atoll, Swains, Taʻū, Tutuila. **Nearest confirmed ecoregion** – Sāmoa, Tuvalu, and Tonga. **Vulnerability** – LC. **Mesophotic record** – 30, 53 m depth (Lamberts 1983; Montgomery et al. 2019). **Notes** – Some earlier identifications prior to and shortly after 2001 of this species likely concern *P.chiriquiensis* because its name was not available or not well known since 2001. However, some reported observations of *Pavona* spp. included potential variations of *P.varians* (Maragos et al. 1994; Coles et al. 2003).

***Pavonavenosa* (Ehrenberg, 1834) (207301)** ^CoTW^

*Pavonavenosa* (Ehrenberg, 1834) (207301). ^CoTW^ Reported – Birkeland et al. 1987, 2003; Hunter et al. 1993; Maragos et al. 1995; Mundy 1996; Green et al. 1997; Green and Hunter 1998; Craig et al. 2001; Fisk and Birkeland 2002; Coles et al. 2003; DiDonato et al. 2006; Birkeland 2007a; Fenner et al. 2008; Kenyon et al. 2010; CRED 2011; Corals NPAS 2016; Fenner 2018. Referenced – Green et al. 1999; Fisk and Birkeland 2002; Coles et al. 2003; DiDonato et al. 2006; Birkeland 2007a, 2007b; Lovell and McLardy 2008; Kenyon et al. 2011.

**American Sāmoa status** – Present. **Evidence** – Multiple photographic records. **Distribution** – American Sāmoa, Aunuʻu, Manuʻa Islands, Ofu, Ofu/Olosega, Olosega, Rose Atoll, Taʻū, Tutuila. **Nearest confirmed ecoregion** – Sāmoa, Tuvalu, and Tonga. **Vulnerability** – VU. **Notes** – The identification of a photographed specimen of this species reported in Corals NPAS (2016) appears to be incorrect and should be *Coscinaraeacolumna* (Dana, 1846).

####### Family Astrocoeniidae Koby, 1890

######## Genus *Stylocoeniella* Yabe & Sugiyama, 1935

***Stylocoeniellaarmata* (Ehrenberg, 1834) (206950)** ^CoTW^

*Stylocoeniaarmata* (Ehrenberg, 1834) (206950) [sic]. Reported – DMWR 2018.

*Stylocoeniellaaramta* (Ehrenberg, 1834) (206950) [sic]. Reported – Birkeland et al. 2003.

*Stylocoeniellaarmata* (Ehrenberg, 1834) (206950). ^CoTW^ Reported – USACE 1980; Lamberts 1983; Birkeland et al. 1987, 2003; Maragos et al. 1994; Mundy 1996; Craig et al. 2001; Fisk and Birkeland 2002; Coles et al. 2003; Birkeland 2007a; Fenner et al. 2008; CRED 2011; Corals NPAS 2016; Fenner 2018. Referenced – Green et al. 1999; Fisk and Birkeland 2002; Coles et al. 2003; DiDonato et al. 2006; Birkeland 2007a, 2007b; Lovell and McLardy 2008.

**American Sāmoa status** – Present. **Evidence** – Multiple specimen reports. **Distribution** – American Sāmoa, Manuʻa Islands, Ofu, Ofu/Olosega, Olosega, Rose Atoll, Taʻū, Tutuila. **Nearest confirmed ecoregion** – Sāmoa, Tuvalu, and Tonga. **Vulnerability** – LC.

***Stylocoeniellaguentheri* (Bassett-Smith, 1890) (206948)** ^CoTW^

*Stylocoeniaguntheri* (Bassett-Smith, 1890) (206948) [sic]. Reported – DMWR 2018.

*Stylocoeniellaguentheri* (Bassett-Smith, 1890) (206948). ^CoTW^ Reported – Fisk and Birkeland 2002; CRED 2011; Corals NPAS 2016; Fenner 2018. Referenced – DiDonato et al. 2006; Birkeland 2007b; Lovell and McLardy 2008.

**American Sāmoa status** – Present. **Evidence** – Single specimen report (identified by D Fenner). **Distribution** – American Sāmoa, Aunuʻu, Manuʻa Islands, Ofu/Olosega, Rose Atoll, Tutuila. **Nearest confirmed ecoregion** – Sāmoa, Tuvalu, and Tonga. **Vulnerability** – LC.

####### Family Coscinaraeidae Benzoni, Arrigoni, Stefani & Stolarski, 2012

######## Genus *Coscinaraea* Milne Edwards & Haime, 1848

***Coscinaraeacolumna* (Dana, 1846) (207256)** ^CoTW^

*Coscinaraeacollumna* (Dana, 1846) (207256) [sic]. Reported – Craig et al. 2001; Fenner et al. 2008; DMWR 2018.

*Coscinaraeacolumn* (Dana, 1846) (207256) [sic]. Reported – USACE 1980.

*Coscinaraeacolumna* (Dana, 1846) (207256). ^CoTW^ Reported – Hoffmeister 1925; Lamberts 1983; Birkeland et al. 1987, 2003; Maragos et al. 1994; Mundy 1996; Green and Hunter 1998; Fisk and Birkeland 2002; DiDonato et al. 2006; Birkeland 2007a; Kenyon et al. 2010; CRED 2011; Corals NPAS 2016; BPBM 2018; Fenner 2018; NMNH 2018; Montgomery et al. 2019. Referenced – Green et al. 1999; Fisk and Birkeland 2002; DiDonato et al. 2006; Birkeland 2007a, 2007b; Lovell and McLardy 2008.

*Coscinareacolumna* (Dana, 1846) (207256) [sic]. Reported – Hunter et al. 1993; Maragos et al. 1995; Fenner et al. 2008.

*Coscinereacolumna* (Dana, 1846) (207256) [sic]. Reported – Mayor 1924b; Coles et al. 2003. Referenced – Coles et al. 2003.

**American Sāmoa status** – Present. **Evidence** – Multiple specimen reports. **Distribution** – American Sāmoa, Aunuʻu, Manuʻa Islands, Ofu, Ofu/Olosega, Olosega, Rose Atoll, Swains, Taʻū, Tutuila. **Nearest confirmed ecoregion** – Sāmoa, Tuvalu, and Tonga. **Vulnerability** – LC. **Mesophotic record** – 46 m depth (Montgomery et al. 2019).

***Coscinaraeaexesa* (Dana, 1846) (287938)** ^CoTW^

*Coscinaraeaexesa* (Dana, 1846) (287938). ^CoTW^ Reported – Green and Hunter 1998; Kenyon et al. 2010; Fenner 2018.

*Coscinareaexesa* (Dana, 1846) (287938) [sic]. Reported – CRED 2011.

**American Sāmoa status** – Present. **Evidence** – Single photographic record. **Distribution** – Rose Atoll, Taʻū, Tutuila. **Nearest confirmed ecoregion** – Sāmoa, Tuvalu, and Tonga. **Vulnerability** – LC.

####### Family Dendrophylliidae Gray, 1847

######## Genus *Endopsammia* Milne Edwards & Haime, 1848

***Endopsammiaregularis* (Gardiner, 1899) (289894)**

*Endopsammiaregularis* (Gardiner, 1899) (289894). Reported – DMWR 2018; Fenner 2018; NMNH 2018.

**American Sāmoa status** – Present. **Evidence** – Multiple specimen reports. **Distribution** – Tutuila. **Nearest confirmed ecoregion** – Not available. **Notes** – This species was collected by D Fenner and identified by S Cairns at the NMNH. Based on the evidence of a collected sample, we accept the presence of this species in American Sāmoa.

######## Genus *Tubastraea* Lesson, 1829

***Tubastraeacoccinea* Lesson, 1829 (291251)**

*Tubastraeaaurea* (Quoy & Gaimard, 1833) (367759) heterotypic synonym. Reported – Birkeland et al. 1987. Referenced – Coles et al. 2003; Birkeland 2007b.

*Tubastraeacoccinea* Lesson, 1829 (291251). Reported – DMWR 2018; Fenner 2018; Montgomery et al. 2019. Referenced – Lovell and McLardy 2008.

*Tubastreacoccinea* Lesson, 1829 (291251) [sic]. Reported – USACE 1980; Lamberts 1983.

**American Sāmoa status** – Present. **Evidence** – Multiple specimen reports. **Distribution** – American Sāmoa, Tutuila. **Nearest confirmed ecoregion** – Not available. **Mesophotic record** – 45 m depth (Montgomery et al. 2019).

***Tubastraeadiaphana* (Dana, 1846) (291252)**

*Dendrophylliadiaphana* Dana, 1846 (210747) homotypic synonym. Reported – Hoffmeister 1925; Lamberts 1983.

*Tubastraeadiaphana* (Dana, 1846) (291252). Reported – NMNH 2018.

**American Sāmoa status** – Present. **Evidence** – Multiple specimen reports. **Distribution** – American Sāmoa, Tutuila. **Nearest confirmed ecoregion** – Not available.

######## Genus *Turbinaria* Oken, 1815

***Turbinariafrondens* (Dana, 1846) (207506)** ^CoTW^

*Turbinareafrondens* ? (Dana, 1846) (207506) [sic]. Reported – Coles et al. 2003.

*Turbinariafrondens* (Dana, 1846) (207506). ^CoTW^ Reported – USACE 1980; Lamberts 1983; Maragos et al. 1994. Referenced – Birkeland 2007b; Lovell and McLardy 2008.

*Turbinariafrondens* cf. (Dana, 1846) (207506). ^CoTW^ Reported – USACE 1980.

**American Sāmoa status** – Present. **Evidence** – Single specimen report (identified by A Lamberts). **Distribution** – American Sāmoa, Olosega, Tutuila. **Nearest confirmed ecoregion** – Sāmoa, Tuvalu, and Tonga. **Vulnerability** – LC.

***Turbinariairregularis* Bernard, 1896 (207505)** ^CoTW^

**Figure 2.**
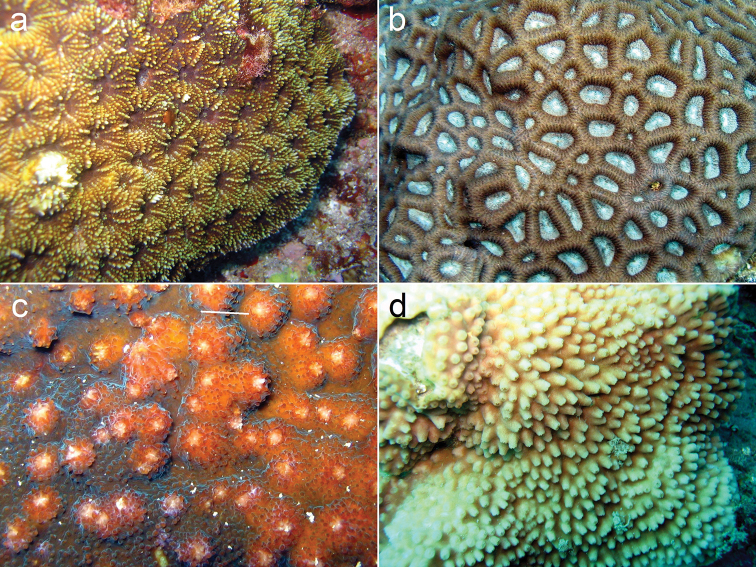
New scleractinian records for American Sāmoa. **a***Acanthastreasubechinata* Veron, 2000 **b***Favitesparaflexuosus* Veron, 2000 **c***Echinophylliaechinoporoides* Veron & Pichon, 1980 **d***Turbinariairregularis* Bernard, 1896. Photographs by D Fenner.

*Turbinariairregularis* Bernard, 1896 (207505). ^CoTW^ Reported – This paper (Figure 2d).

**American Sāmoa status** – Present. **Evidence** – Single photographic record. **Distribution** – Tutuila. **Nearest confirmed ecoregion** – Fiji. **Geographical range extension** – East although Veron et al. (2019) strongly predicted the presence of this species in the Sāmoa, Tuvalu, and Tonga ecoregion. **Vulnerability** – LC. **Notes** – This species is presented here as a new record (Figure 2d).

***Turbinariamesenterina* (Lamarck, 1816) (207511)** ^CoTW^

*Turbinariamesenterina* (Lamarck, 1816) (207511). ^CoTW^ Reported – Green and Hunter 1998; DiDonato et al. 2006; Birkeland 2007a; Corals NPAS 2016; DMWR 2018; Fenner 2018. Referenced – Lovell and McLardy 2008; Kenyon et al. 2011.

**American Sāmoa status** – Present. **Evidence** – Single specimen report (identified by D Fenner). **Distribution** – American Sāmoa, Ofu, Ofu/Olosega, Rose Atoll, Taʻū, Tutuila. **Nearest confirmed ecoregion** – Sāmoa, Tuvalu, and Tonga. **Vulnerability** – VU.

***Turbinariapeltata* (Esper, 1794) (207512)** ^CoTW^

*Turbinariapeltata* (Esper, 1794) (207512). ^CoTW^ Reported – USACE 1980; Lamberts 1983; Maragos et al. 1994; Green and Hunter 1998; Fenner 2018; Montgomery et al. 2019. Referenced – Birkeland 2007b; Lovell and McLardy 2008; Kenyon et al. 2011.

**American Sāmoa status** – Present. **Evidence** – Multiple specimen reports. **Distribution** – American Sāmoa, Aunuʻu, South Bank, Tutuila. **Nearest confirmed ecoregion** – Sāmoa, Tuvalu, and Tonga. **Vulnerability** – VU. **Mesophotic record** – 30, 49 m depth (Lamberts 1983; Montgomery et al. 2019).

***Turbinariareniformis* Bernard, 1896 (207507)** ^CoTW^

*Turbinareareniformis* Bernard, 1896 (207507) [sic]. Reported – Coles et al. 2003. Referenced – Coles et al. 2003.

*Turbinariareniformis* Bernard, 1896 (207507). ^CoTW^ Reported – Birkeland et al. 1987; Itano and Buckley 1988; Hunter et al. 1993; Maragos et al. 1994, 1995; Mundy 1996; Craig et al. 2001; Fisk and Birkeland 2002; DiDonato et al. 2006; Birkeland 2007a; CRED 2011; Corals NPAS 2016; Fenner and Sudek 2016; DMWR 2018; Fenner 2018. Referenced – Green et al. 1999; Fisk and Birkeland 2002; Birkeland 2007a, 2007b; Lovell and McLardy 2008; Kenyon et al. 2011.

*Turbinariaveluta* Bernard, 1896 (767034) possible heterotypic synonym. Reported – Maragos et al. 1994; Corals NPAS 2016. Referenced – DiDonato et al. 2006.

**American Sāmoa status** – Present. **Evidence** – Single specimen report (identified by D Fenner and J Wolstenholme). **Distribution** – American Sāmoa, Aunuʻu, Manuʻa Islands, Ofu, Ofu/Olosega, Olosega, Rose Atoll, Taʻū, Tutuila. **Nearest confirmed ecoregion** – Sāmoa, Tuvalu, and Tonga. **Vulnerability** – VU.

***Turbinariastellulata* (Lamarck, 1816) (207510)** ^CoTW^

*Turbinariastellulata* (Lamarck, 1816) (207510). ^CoTW^ Reported – Maragos et al. 1994, 1995; Green and Hunter 1998; DiDonato et al. 2006; CRED 2011; Corals NPAS 2016; DMWR 2018; Fenner 2018; Montgomery et al. 2019. Referenced – DiDonato et al. 2006; Birkeland 2007a, 2007b; Lovell and McLardy 2008; Kenyon et al. 2011.

**American Sāmoa status** – Present. **Evidence** – Multiple specimen reports. **Distribution** – American Sāmoa, Ofu, Ofu/Olosega, Olosega, Rose Atoll, Taʻū, Tutuila. **Nearest confirmed ecoregion** – Sāmoa, Tuvalu, and Tonga. **Vulnerability** – VU. **Mesophotic record** – 51 m depth (Montgomery et al. 2019).

####### Family Diploastreidae Chevalier & Beauvais, 1987

######## Genus *Diploastrea* Matthai, 1914

***Diploastreaheliopora* (Lamarck, 1816) (207417)** ^CoTW^

*Diploastreaheliopora* (Lamarck, 1816) (207417). ^CoTW^ Reported – Hoffmeister 1925; USACE 1980; Lamberts 1983; Birkeland et al. 1987, 2003; Itano and Buckley 1988; Hunter et al. 1993; Maragos et al. 1994; Mundy 1996; Green and Hunter 1998; Fisk and Birkeland 2002; Work and Rameyer 2002; Coles et al. 2003; Cornish and DiDonato 2004; DiDonato et al. 2006; Fenner et al. 2008; Kenyon et al. 2010; CRED 2011; Corals NPAS 2016; Fenner and Sudek 2016; DMWR 2018; Fenner 2018; NMNH 2018; Gross 2019; Montgomery et al. 2019. Referenced – Fisk and Birkeland 2002; Coles et al. 2003; DiDonato et al. 2006; Birkeland 2007a, 2007b; Lovell and McLardy 2008.

**American Sāmoa status** – Present. **Evidence** – Multiple specimen reports. **Distribution** – American Sāmoa, Manuʻa Islands, Ofu, Ofu/Olosega, Olosega, Taʻū, Tutuila. **Nearest confirmed ecoregion** – Sāmoa, Tuvalu, and Tonga. **Vulnerability** – NT. **Mesophotic record** – 43 m depth (Montgomery et al. 2019). **Notes** – This species is relatively easy to identify.

####### Family Euphylliidae Alloiteau, 1952

######## Genus *Euphyllia* Dana, 1846

***Euphylliaglabrescens* (Chamisso & Eysenhardt, 1821) (207617)** ^CoTW^

*Euphylliaglabrescens* (Chamisso & Eysenhardt, 1821) (207617). ^CoTW^ Reported – Hoffmeister 1925; USACE 1980; Lamberts 1983; Birkeland et al. 1987; Maragos et al. 1994; Coles et al. 2003; DMWR 2018; Fenner 2018; NMNH 2018; Montgomery et al. 2019. Referenced – Green et al. 1999; Coles et al. 2003; Birkeland 2007b; Lovell and McLardy 2008.

**American Sāmoa status** – Present. **Evidence** – Multiple photographic records. **Distribution** – American Sāmoa, Aunuʻu, Tutuila. **Nearest confirmed ecoregion** – Sāmoa, Tuvalu, and Tonga. **Vulnerability** – NT. **Mesophotic record** – 49 m depth (Montgomery et al. 2019). **Notes** – Identification of this species requires both skeleton shape and tentacle shape.

######## Genus *Fimbriaphyllia* Veron & Pichon, 1980

***Fimbriaphylliaparadivisa* (Veron, 1990) (1048080)**

*Euphylliaparadivisa* Veron, 1990 (207615) homotypic synonym. ^CoTW^ Reported – Fenner 2018; Montgomery et al. 2019. Referenced – Kenyon et al. 2011.

**American Sāmoa status** – Present. **Evidence** – Multiple photographic records. **Distribution** – Tutuila. **Nearest confirmed ecoregion** – Sāmoa, Tuvalu, and Tonga. **Vulnerability** – 𝒯. **Mesophotic record** – 49 m depth (Montgomery et al. 2019). **Notes** – The identification of this species is well documented by photographic evidence and is a conclusive identification. Identification of this species requires both skeleton shape and tentacle shape.

######## Genus *Galaxea* Oken, 1815

***Galaxeaastreata* (Lamarck, 1816) (207368)** ^CoTW^

*Galaxeaastreata* (Lamarck, 1816) (207368). ^CoTW^ Reported – Maragos et al. 1994; Mundy 1996; Fisk and Birkeland 2002; Birkeland 2007a; Fenner et al. 2008; Corals NPAS 2016; DMWR 2018; Fenner 2018; Montgomery et al. 2019. Referenced – Fisk and Birkeland 2002; DiDonato et al. 2006; Birkeland 2007a, 2007b; Lovell and McLardy 2008; Kenyon et al. 2011.

*Galaxeaclavus* Dana, 1846 (207367) heterotypic synonym. Reported – USACE 1980; Lamberts 1983.

**American Sāmoa status** – Present. **Evidence** – Multiple specimen reports. **Distribution** – American Sāmoa, Aunuʻu, Manuʻa Islands, Ofu, Ofu/Olosega, Olosega, Taʻū, Tutuila. **Nearest confirmed ecoregion** – Sāmoa, Tuvalu, and Tonga. **Vulnerability** – VU. **Mesophotic record** – 46 m depth (Montgomery et al. 2019).

***Galaxeafascicularis* (Linnaeus, 1767) (207366)** ^CoTW^

*Galaxeafascicularis* (Linnaeus, 1767) (207366). ^CoTW^ Reported – Mayor 1924b; Hoffmeister 1925; USACE 1980; Lamberts 1983; Birkeland et al. 1987, 2003; Itano and Buckley 1988; Hunter et al. 1993; Maragos et al. 1994, 1995; Mundy 1996; Green and Hunter 1998; Craig et al. 2001; Fisk and Birkeland 2002; Coles et al. 2003; DiDonato et al. 2006; Birkeland 2007a; Fenner et al. 2008; Kenyon et al. 2010; CRED 2011; Corals NPAS 2016; Fenner and Sudek 2016; DMWR 2018; Fenner 2018; NMNH 2018; Creuwels 2019; Montgomery et al. 2019. Referenced – Dahl and Lamberts 1977; Dahl 1981; Green et al. 1997, 1999; Fisk and Birkeland 2002; Coles et al. 2003; DiDonato et al. 2006; Birkeland 2007a, 2007b; Lovell and McLardy 2008.

*Galaxeefascicularia* (Linnaeus, 1767) (207366) [sic]. Reported – Birkeland et al. 1987.

*Galaxiafascicularis* (Linnaeus, 1767) (207366) [sic]. Reported – Mayor 1924a.

**American Sāmoa status** – Present. **Evidence** – Multiple specimen reports. **Distribution** – American Sāmoa, Aunuʻu, Manuʻa Islands, Ofu, Ofu/Olosega, Olosega, Rose Atoll, Taʻū, Tutuila. **Nearest confirmed ecoregion** – Sāmoa, Tuvalu, and Tonga. **Vulnerability** – NT. **Mesophotic record** – 46 m depth (Montgomery et al. 2019).

####### Family Fungiidae Dana, 1846

######## Genus *Ctenactis* Verrill, 1864

***Ctenactiscrassa* (Dana, 1846) (288875)** ^CoTW^

*Ctenactiscrassa* (Dana, 1846) (288875). ^CoTW^ Reported – Bare et al. 2010; Fenner 2018. Referenced – Lovell and McLardy 2008.

*Herpetoglosasimplex* (Gardiner, 1905) (211417) [sic] heterotypic synonym. Reported – Birkeland et al. 1987.

*Herpetoqlosasimplex* (Gardiner, 1905) (211417) [sic] heterotypic synonym. Referenced – Birkeland 2007b.

**American Sāmoa status** – Present. **Evidence** – Single photographic record. **Distribution** – American Sāmoa, Aunuʻu, Tutuila. **Nearest confirmed ecoregion** – Sāmoa, Tuvalu, and Tonga. **Vulnerability** – LC.

***Ctenactisechinata* (Pallas, 1766) (216132)** ^CoTW^

*Ctenactisechinata* (Pallas, 1766) (216132). ^CoTW^ Reported – Maragos et al. 1994; Bare et al. 2010; Corals NPAS 2016; Fenner 2018. Referenced – DiDonato et al. 2006; Birkeland 2007a; Lovell and McLardy 2008.

*Fungiaechinata* (Pallas, 1766) (367892) homotypic synonym. Reported – USACE 1980; Lamberts 1983; Coles et al. 2003.

**American Sāmoa status** – Present. **Evidence** – Single specimen report (identified by A Lamberts). **Distribution** – American Sāmoa, Ofu, Ofu/Olosega, Tutuila. **Nearest confirmed ecoregion** – Sāmoa, Tuvalu, and Tonga. **Vulnerability** – LC. **Mesophotic record** – 30 m depth (Lamberts 1983).

######## Genus *Cycloseris* Milne Edwards & Haime, 1849

***Cycloseriscostulata* (Ortmann, 1889) (207325)** ^CoTW^

*Cycloseriscostulata* (Ortmann, 1889) (207325). ^CoTW^ Reported – Fenner 2018; Montgomery et al. 2019.

**American Sāmoa status** – Present. **Evidence** – Multiple photographic records. **Distribution** – Tutuila. **Nearest confirmed ecoregion** – Sāmoa, Tuvalu, and Tonga. **Mesophotic record** – 35 m depth (Montgomery et al. 2019).

***Cycloserisfragilis* (Alcock, 1893) (716448)**

*Cycloserisfragilis* (Alcock, 1893) (716448). Reported – Kenyon et al. 2010.

*Cycloserispatelliformis* (Boschma, 1923) (207329) heterotypic synonym. ^CoTW^ Reported – Maragos et al. 1994; Corals NPAS 2016. Referenced – Coles et al. 2003; DiDonato et al. 2006; Birkeland 2007a, 2007b; Lovell and McLardy 2008.

*Fungiafragilis* (Alcock, 1893) (207333) homotypic synonym. Reported – Hoeksema 1989; NMNH 2018.

*Fungiapatelliformis* Boschma, 1923 (716681) heterotypic synonym. Reported – Mayor 1924a; Hoffmeister 1925; USACE 1980; Lamberts 1983.

**American Sāmoa status** – Present. **Evidence** – Multiple specimen reports. **Distribution** – American Sāmoa, Ofu, Ofu/Olosega, Rose Atoll, Tutuila. **Nearest confirmed ecoregion** – Sāmoa, Tuvalu, and Tonga. **Mesophotic record** – 33, 30 m depth (Hoffmeister 1925; Lamberts 1983). **Notes** – Veron et al. (2019) name this species *Diaserisfragilis* Alcock, 1893.

***Cycloseristenuis* (Dana, 1846) (207324)** ^CoTW^

*Cycloseristenuis* (Dana, 1846) (207324). ^CoTW^ Reported – Fenner 2018.

**American Sāmoa status** – Present. **Evidence** – Single photographic record. **Distribution** – Tutuila. **Nearest confirmed ecoregion** – Fiji and Society Islands, French Polynesia. **Geographical range extension** – Between two disjunct ecoregions although Veron et al. (2019) strongly predicted the presence of this species in the Sāmoa, Tuvalu, and Tonga ecoregion.

***Cycloserisvaughani* (Boschma, 1923) (207327)** ^CoTW^

*Cycloserisvaughani* (Boschma, 1923) (207327). ^CoTW^ Reported – Montgomery et al. 2019.

*Cycloserisvaughani* cf. (Boschma, 1923) (207327). ^CoTW^ Reported – DMWR 2018.

**American Sāmoa status** – Present. **Evidence** – Single specimen report (identified by D Fenner). **Distribution** – Tutuila. **Nearest confirmed ecoregion** – Sāmoa, Tuvalu, and Tonga. **Mesophotic record** – 47 m depth (Montgomery et al. 2019). **Notes** – Other mesophotic records are known from eastern Indonesia, eastern Australia and Easter Island (Hoeksema 2012c; Muir et al. 2018; Hoeksema et al. 2019).

######## Genus *Danafungia* Wells, 1966

***Danafungiahorrida* (Dana, 1846) (716608)**

*Fungiadanae* Milne Edwards & Haime, 1851 (716867) heterotypic synonym. Reported – Maragos et al. 1994; Corals NPAS 2016.

*Fungiadanai* Milne Edwards & Haime, 1851 (207343) heterotypic synonym, wrong species spelling. ^CoTW^ Reported – Birkeland et al. 1987, 2003; Mundy 1996; Coles et al. 2003. Referenced – Fisk and Birkeland 2002; Coles et al. 2003; DiDonato et al. 2006; Birkeland 2007a, 2007b; Lovell and McLardy 2008.

*Fungiahorrida* Dana, 1846 (207355) homotypic synonym. ^CoTW^ Reported – Maragos et al. 1994; Mundy 1996; Fisk and Birkeland 2002; Kenyon et al. 2010; Corals NPAS 2016; Fenner 2018; Montgomery et al. 2019. Referenced – Fisk and Birkeland 2002; Coles et al. 2003; DiDonato et al. 2006; Birkeland 2007a, 2007b; Lovell and McLardy 2008.

*Fungiaklunzingeri* cf. Döderlein, 1901 (207354) heterotypic synonym. ^CoTW^ Reported – DMWR 2018.

*Fungiaklunzingeri* Döderlein, 1901 (207354) heterotypic synonym. ^CoTW^ Reported – Maragos et al. 1994; Mundy 1996; Fenner et al. 2008; DMWR 2018. Referenced – Birkeland 2007b; Lovell and McLardy 2008.

*Fungiavalida* Verrill, 1864 (207358) heterotypic synonym. Reported – Maragos et al. 1994.

**American Sāmoa status** – Present. **Evidence** – Single specimen report (synonym *Fungiaklunzingeri* identified by D Fenner), Multiple photographic records. **Distribution** – American Sāmoa, Aunuʻu, Manuʻa Islands, Ofu, Ofu/Olosega, Olosega, Taʻū, Tutuila. **Nearest confirmed ecoregion** – Sāmoa, Tuvalu, and Tonga. **Mesophotic record** – 39 m depth (Montgomery et al. 2019).

***Danafungiascruposa* (Klunzinger, 1879) (716609)**

*Fungiascruposa* Klunzinger, 1879 (207340) homotypic synonym. ^CoTW^ Reported – Fisk and Birkeland 2002; Fenner 2018.

**American Sāmoa status** – Present. **Evidence** – Single photographic record. **Distribution** – Aunuʻu, Manuʻa Islands, Olosega, Tutuila. **Nearest confirmed ecoregion** – Sāmoa, Tuvalu, and Tonga.

######## Genus *Fungia* Lamarck, 1801

***Fungiafungites* (Linnaeus, 1758) (207350)** ^CoTW^

*Fungiafungites* (Linnaeus, 1758) (207350). ^CoTW^ Reported – Hoffmeister 1925; USACE 1980; Lamberts 1983; Birkeland et al. 1987, 2003; Hoeksema 1989; Hunter et al. 1993; Maragos et al. 1994, 1995; Mundy 1996; Green and Hunter 1998; Craig et al. 2001; Fisk and Birkeland 2002; Coles et al. 2003; DiDonato et al. 2006; Birkeland 2007a; Fenner et al. 2008; Kenyon et al. 2010; CRED 2011; Corals NPAS 2016; Fenner and Sudek 2016; DMWR 2018; Fenner 2018; NMNH 2018. Referenced – Green et al. 1997, 1999; Fisk and Birkeland 2002; Coles et al. 2003; DiDonato et al. 2006; Birkeland 2007a, 2007b; Lovell and McLardy 2008.

**American Sāmoa status** – Present. **Evidence** – Multiple specimen reports. **Distribution** – American Sāmoa, Aunuʻu, Manuʻa Islands, Ofu, Ofu/Olosega, Olosega, Rose Atoll, Taʻū, Tutuila. **Nearest confirmed ecoregion** – Sāmoa, Tuvalu, and Tonga. **Vulnerability** – NT.

######## Genus *Halomitra* Dana, 1846

***Halomitrapileus* (Linnaeus, 1758) (207361)** ^CoTW^

*Halomitrapileus* (Linnaeus, 1758) (207361). ^CoTW^ Reported – USACE 1980; Birkeland et al. 1987; Maragos et al. 1994; Mundy 1996; Green and Hunter 1998; Coles et al. 2003; Corals NPAS 2016; BPBM 2018; DMWR 2018; Fenner 2018. Referenced – Fisk and Birkeland 2002; DiDonato et al. 2006; Birkeland 2007a, 2007b; Lovell and McLardy 2008.

**American Sāmoa status** – Present. **Evidence** – Multiple specimen reports. **Distribution** – American Sāmoa, Aunuʻu, Manuʻa Islands, Ofu, Ofu/Olosega, Sāmoa Islands, Taʻū, Tutuila. **Nearest confirmed ecoregion** – Sāmoa, Tuvalu, and Tonga. **Vulnerability** – LC.

######## Genus *Herpolitha* Eschscholtz, 1825

***Herpolithalimax* (Esper, 1797) (207363)** ^CoTW^

*Herpolithalimax* (Esper, 1797) (207363). ^CoTW^ Reported – USACE 1980; Lamberts 1983; Birkeland et al. 1987; Maragos et al. 1994; Green and Hunter 1998; Coles et al. 2003; Kenyon et al. 2010; CRED 2011; Corals NPAS 2016; DMWR 2018; Fenner 2018; Montgomery et al. 2019. Referenced – Coles et al. 2003; DiDonato et al. 2006; Birkeland 2007a, 2007b; Lovell and McLardy 2008.

*Herpolithaweberi* cf. (van der Horst, 1921) (411207) heterotypic synonym. ^CoTW^ Reported – DMWR 2018.

**American Sāmoa status** – Present. **Evidence** – Multiple specimen reports. **Distribution** – American Sāmoa, Aunuʻu, Ofu/Olosega, Rose Atoll, Taʻū, Tutuila. **Nearest confirmed ecoregion** – Sāmoa, Tuvalu, and Tonga. **Vulnerability** – LC. **Mesophotic record** – 30, 47 m depth (Lamberts 1983; Montgomery et al. 2019). **Notes** – Veron et al. (2019) report this species is distinguishable from *H.weberi* only when they co-occur, although they were synonymized by Hoeksema (1989) who considered the two dredged fragmented type specimens of *H.weberi* to represent a deep-water ecomorph of *H.limax*. It is possible that the specimens identified by Veron (2000) are thick, juvenile specimens and therefore do not show the character of full-grown corals while the types are very thin and from deeper (maybe silty) substrates (B Hoeksema pers. comm.). More work should be done on these two species.

######## Genus *Lithophyllon* Rehberg, 1892

***Lithophyllonconcinna* (Verrill, 1864) (716645)**

*Fungiaconcinna* Verrill, 1864 (207353) homotypic synonym. ^CoTW^ Reported – USACE 1980; Lamberts 1983; Birkeland et al. 1987; Maragos et al. 1994; Mundy 1996; Green and Hunter 1998; Fisk and Birkeland 2002; Fenner et al. 2008; Kenyon et al. 2010; Corals NPAS 2016; DMWR 2018; Fenner 2018. Referenced – Fisk and Birkeland 2002; Coles et al. 2003; DiDonato et al. 2006; Birkeland 2007a, 2007b; Lovell and McLardy 2008.

*Fungiaconncina* Verrill, 1864 (207353) [sic] homotypic synonym. Reported – Fenner et al. 2008.

*Lithophyllonconcinna* (Verrill, 1864) (716645). Reported – Montgomery et al. 2019.

**American Sāmoa status** – Present. **Evidence** – Multiple specimen reports. **Distribution** – American Sāmoa, Aunuʻu, Manuʻa Islands, Ofu, Ofu/Olosega, Olosega, Rose Atoll, Swains, Taʻū, Tutuila. **Nearest confirmed ecoregion** – Sāmoa, Tuvalu, and Tonga. **Mesophotic record** – 49 m depth (Montgomery et al. 2019).

***Lithophyllonrepanda* (Dana, 1846) (716653)**

*Fungiarepanda* Dana, 1846 (207359) homotypic synonym. ^CoTW^ Reported – USACE 1980; Lamberts 1983; Birkeland et al. 1987, 2003; Hoeksema 1989; Maragos et al. 1994; Mundy 1996; Coles et al. 2003; Kenyon et al. 2010; CRED 2011; Corals NPAS 2016; DMWR 2018; Fenner 2018; NMNH 2018. Referenced – Green et al. 1999; Fisk and Birkeland 2002; Coles et al. 2003; DiDonato et al. 2006; Birkeland 2007a, 2007b; Lovell and McLardy 2008.

**American Sāmoa status** – Present. **Evidence** – Multiple specimen reports. **Distribution** – American Sāmoa, Aunuʻu, Manuʻa Islands, Ofu, Ofu/Olosega, Olosega, Rose Atoll, Taʻū, Tutuila. **Nearest confirmed ecoregion** – Sāmoa, Tuvalu, and Tonga.

######## Genus *Lobactis* Verrill, 1864

***Lobactisscutaria* (Lamarck, 1801) (716542)**

*Fungiascutaria* Lamarck, 1801 (207341) homotypic synonym. ^CoTW^ Reported – USACE 1980; Lamberts 1983; Birkeland et al. 1987, 2003; Hoeksema 1989; Hunter et al. 1993; Maragos et al. 1994; Mundy 1996; Green and Hunter 1998; Craig et al. 2001; Fisk and Birkeland 2002; Coles et al. 2003; Birkeland 2007a; Fenner et al. 2008; Kenyon et al. 2010; CRED 2011; Corals NPAS 2016; DMWR 2018; Fenner 2018; NMNH 2018. Referenced – Green et al. 1999; Fisk and Birkeland 2002; Coles et al. 2003; DiDonato et al. 2006; Birkeland 2007a, 2007b; Lovell and McLardy 2008.

*Fungisscutaria* Lamarck, 1801 (207341) [sic] homotypic synonym. Reported – Birkeland et al. 1987.

*Lobactisscutaria* (Lamarck, 1801) (716542). Reported – Montgomery et al. 2019.

**American Sāmoa status** – Present. **Evidence** – Multiple specimen reports. **Distribution** – American Sāmoa, Aunuʻu, Manuʻa Islands, Ofu, Ofu/Olosega, Olosega, Rose Atoll, Swains, Taʻū, Tutuila. **Nearest confirmed ecoregion** – Sāmoa, Tuvalu, and Tonga. **Mesophotic record** – 39 m depth (Montgomery et al. 2019). **Notes** – This species is relatively easy to identify.

######## Genus *Pleuractis* Verrill, 1864

***Pleuractisgranulosa* (Klunzinger, 1879) (716549)**

*Fungiagransulosa* Klunzinger, 1879 (207348) [sic] homotypic synonym. Reported – USACE 1980.

*Fungiagranulosa* Klunzinger, 1879 (207348) homotypic synonym. ^CoTW^ Reported – Lamberts 1983; Kenyon et al. 2010; CRED 2011; DMWR 2018; Fenner 2018. Referenced – Lovell and McLardy 2008.

*Pleuractisgranulosa* (Klunzinger, 1879) (716549). Reported – Creuwels 2019; Montgomery et al. 2019.

**American Sāmoa status** – Present. **Evidence** – Multiple specimen reports. **Distribution** – American Sāmoa, Ofu/Olosega, Rose Atoll, Taʻū, Tutuila. **Nearest confirmed ecoregion** – Sāmoa, Tuvalu, and Tonga. **Mesophotic record** – 30, 44 m depth (Lamberts 1983; Montgomery et al. 2019).

***Pleuractisgravis* (Nemenzo, 1955) (716550)**

*Fungiagravis* Nemenzo, 1955 (288853) homotypic synonym. ^CoTW^ Reported – Fenner 2018.

**American Sāmoa status** – Present. **Evidence** – Single photographic record. **Distribution** – Tutuila. **Nearest confirmed ecoregion** – Fiji and Society Islands, French Polynesia. **Geographical range extension** – Between two disjunct ecoregions although Veron et al. (2019) strongly predicted the presence of this species in the Sāmoa, Tuvalu, and Tonga ecoregion.

***Pleuractismoluccensis* (Van der Horst, 1919) (716545)**

*Fungiamolluccensis* Van der Horst, 1919 (207337) [sic] homotypic synonym. Reported – Fisk and Birkeland 2002.

*Fungiamoloccensis* Van der Horst, 1919 (207337) [sic] homotypic synonym. Reported – DMWR 2018.

*Fungiamoluccensis* Van der Horst, 1919 (207337) homotypic synonym. ^CoTW^ Reported – Hoeksema 1989; Fenner 2018; NMNH 2018.

*Pleuractismoluccensis* (Van der Horst, 1919) (716545). Reported – Montgomery et al. 2019.

**American Sāmoa status** – Present. **Evidence** – Multiple specimen reports. **Distribution** – Manuʻa Islands, Tutuila. **Nearest confirmed ecoregion** – Sāmoa, Tuvalu, and Tonga. **Mesophotic record** – 48 m depth (Montgomery et al. 2019).

***Pleuractispaumotensis* (Stutchbury, 1833) (716547)**

*Fungiapaumotensis* Stutchbury, 1833 (207339) homotypic synonym. ^CoTW^ Reported – Mayor 1924b; Hoffmeister 1925; USACE 1980; Lamberts 1983; Maragos et al. 1994; Coles et al. 2003; Corals NPAS 2016; DMWR 2018; Fenner 2018. Referenced – DiDonato et al. 2006; Birkeland 2007a, 2007b; Lovell and McLardy 2008.

**American Sāmoa status** – Present. **Evidence** – Multiple specimen reports. **Distribution** – American Sāmoa, Aunuʻu, Ofu, Ofu/Olosega, Rose Atoll, Taʻū, Tutuila. **Nearest confirmed ecoregion** – Sāmoa, Tuvalu, and Tonga.

######## Genus *Polyphyllia* Blainville, 1830

***Polyphyllianovaehiberniae* (Lesson, 1831) (289231)** ^CoTW^

*Lithactinianovaehiberniae* Lesson, 1831 (717282) homotypic synonym. Reported – Lamberts 1983.

*Polyphyllianovae-hiberniae* (Lesson, 1831) (289231) [sic]. Reported – USACE 1980.

*Polyphyllianovaehibernae* (Lesson, 1831) (289231) [sic]. Reported – Fenner 2018.

*Polyphyllianovaehiberniae* (Lesson, 1831) (289231). ^CoTW^ Reported – NMNH 2018. Referenced – Lovell and McLardy 2008.

*Polyphyllianovohibernae* (Lesson, 1831) (289231) [sic]. Reported – DMWR 2018.

**American Sāmoa status** – Present. **Evidence** – Multiple specimen reports. **Distribution** – American Sāmoa, Tutuila. **Nearest confirmed ecoregion** – Sāmoa, Tuvalu, and Tonga. **Vulnerability** – NT.

######## Genus *Sandalolitha* Quelch, 1884

***Sandalolithadentata* Quelch, 1884 (291009)** ^CoTW^

*Sandalolithadentata* Quelch, 1884 (291009). ^CoTW^ Reported – DMWR 2018; Fenner 2018; Montgomery et al. 2019.

*Sandalothiadentata* Quelch, 1884 (291009) [sic]. Reported – Bare et al. 2010.

**American Sāmoa status** – Present. **Evidence** – Single specimen report (identified by D Fenner). **Distribution** – Aunuʻu, Tutuila. **Nearest confirmed ecoregion** – Sāmoa, Tuvalu, and Tonga. **Vulnerability** – LC. **Mesophotic record** – 47 m depth (Montgomery et al. 2019).

***Sandalolitharobusta* (Quelch, 1886) (291010)** ^CoTW^

*Sandalolitharobusta* (Quelch, 1886) (291010). ^CoTW^ Reported – Birkeland et al. 1987; Maragos et al. 1994; Mundy 1996; Fisk and Birkeland 2002; Corals NPAS 2016; DMWR 2018; Fenner 2018; Montgomery et al. 2019. Referenced – Fisk and Birkeland 2002; DiDonato et al. 2006; Birkeland 2007a, 2007b; Lovell and McLardy 2008.

*Sandolitharobusta* (Quelch, 1886) (291010) [sic]. Reported – Coles et al. 2003. Referenced – Coles et al. 2003.

**American Sāmoa status** – Present. **Evidence** – Single specimen report (identified by J Wolstenholme). **Distribution** – American Sāmoa, Aunuʻu, Manuʻa Islands, Ofu, Ofu/Olosega, Olosega, Rose Atoll, South Bank, Taʻū, Tutuila. **Nearest confirmed ecoregion** – Sāmoa, Tuvalu, and Tonga. **Vulnerability** – LC. **Mesophotic record** – 39 m depth (Montgomery et al. 2019).

####### Family Lobophylliidae Dai & Horng, 2009

######## Genus *Acanthastrea* Milne Edwards & Haime, 1848

***Acanthastreabrevis* Milne Edwards & Haime, 1849 (430639)** ^CoTW^

*Acanthastreabrevis* cf. Milne Edwards & Haime, 1849 (430639). ^CoTW^ Reported – Montgomery et al. 2019.

*Acanthastreabrevis* Milne Edwards & Haime, 1849 (430639). ^CoTW^ Reported – DMWR 2018; Fenner 2018; Montgomery et al. 2019. Referenced – Kenyon et al. 2011.

**American Sāmoa status** – Present. **Evidence** – Single specimen report (identified by D Fenner). **Distribution** – Ofu/Olosega, Rose Atoll, Taʻū, Tutuila. **Nearest confirmed ecoregion** – Sāmoa, Tuvalu, and Tonga. **Vulnerability** – VU. **Mesophotic record** – 46 m depth (Montgomery et al. 2019).

***Acanthastreaechinata* (Dana, 1846) (207384)** ^CoTW^

*Acanthastreaechinata* (Dana, 1846) (207384). ^CoTW^ Reported – USACE 1980; Lamberts 1983; Birkeland et al. 1987; Maragos et al. 1994; Mundy 1996; Fisk and Birkeland 2002; Coles et al. 2003; DiDonato et al. 2006; Birkeland 2007a; Kenyon et al. 2010; Corals NPAS 2016; DMWR 2018; Fenner 2018; Montgomery et al. 2019. Referenced – Green et al. 1999; Fisk and Birkeland 2002; Coles et al. 2003; DiDonato et al. 2006; Birkeland 2007a, 2007b; Lovell and McLardy 2008.

**American Sāmoa status** – Present. **Evidence** – Multiple specimen reports. **Distribution** – American Sāmoa, Aunuʻu, Manuʻa Islands, Ofu, Ofu/Olosega, Olosega, Rose Atoll, Taʻū, Tutuila. **Nearest confirmed ecoregion** – Sāmoa, Tuvalu, and Tonga. **Vulnerability** – LC. **Mesophotic record** – 39 m depth (Montgomery et al. 2019).

***Acanthastreahemprichii* (Ehrenberg, 1834) (288878)** ^CoTW^

*Acanthastreahemprichii* (Ehrenberg, 1834) (288878). ^CoTW^ Reported – Fenner 2018. Referenced – Kenyon et al. 2011.

**American Sāmoa status** – Present. **Evidence** – Single photographic record. **Distribution** – Tutuila. **Nearest confirmed ecoregion** – Fiji. **Geographical range extension** – East although Veron et al. (2019) strongly predicted the presence of this species in the Sāmoa, Tuvalu, and Tonga ecoregion. **Vulnerability** – VU. **Notes** – This species is documented by clear photographic evidence (Fenner 2018) to support the record of its presence in American Sāmoa.

***Acanthastreasubechinata* Veron, 2000 (288885)** ^CoTW^

*Acanthastreasubechinata* Veron, 2000 (288885). ^CoTW^ Reported – This paper (Figure 2a).

**American Sāmoa status** – Present. **Evidence** – Single photographic record. **Distribution** – Ofu, Taʻū, Tutuila. **Nearest confirmed ecoregion** – Solomon Islands and Bougainville. **Geographical range extension** – East. **Vulnerability** – NT. **Notes** – This species is presented here as a new record (Figure 2a).

######## Genus *Echinomorpha* Veron, 2000

***Echinomorphanishihirai* (Veron, 1990) (289877)** ^CoTW^

*Echinomorphanishihirai* (Veron, 1990) (289877). ^CoTW^ Reported – Fenner 2018.

**American Sāmoa status** – Present. **Evidence** – Single photographic record. **Distribution** – Ofu/Olosega, Tutuila. **Nearest confirmed ecoregion** – Sāmoa, Tuvalu, and Tonga. **Vulnerability** – NT.

######## Genus *Echinophyllia* Klunzinger, 1879

***Echinophylliaaspera* (Ellis & Solander, 1786) (207370)** ^CoTW^

*Echinophylliaaspera* (Ellis & Solander, 1786) (207370). ^CoTW^ Reported – USACE 1980; Birkeland et al. 1987; Maragos et al. 1994; Mundy 1996; Fisk and Birkeland 2002; Coles et al. 2003; Kenyon et al. 2010; Corals NPAS 2016; DMWR 2018; Fenner 2018; Montgomery et al. 2019. Referenced – Green et al. 1999; Fisk and Birkeland 2002; Coles et al. 2003; DiDonato et al. 2006; Birkeland 2007a, b; Lovell and McLardy 2008.

*Echinoporaaspera* (Ellis & Solander, 1786) (766286) homotypic synonym. Reported – Lamberts 1983.

**American Sāmoa status** – Present. **Evidence** – Single specimen report (identified by D Fenner). **Distribution** – American Sāmoa, Aunuʻu, Manuʻa Islands, Ofu, Ofu/Olosega, Olosega, Rose Atoll, Taʻū, Tutuila. **Nearest confirmed ecoregion** – Sāmoa, Tuvalu, and Tonga. **Vulnerability** – LC. **Mesophotic record** – 30, 40 m depth (Lamberts 1983; Montgomery et al. 2019).

***Echinophylliaechinoporoides* Veron & Pichon, 1980 (287973)** ^CoTW^

*Echinophylliaechinoporoides* Veron & Pichon, 1980 (287973). ^CoTW^ Reported – This paper (Figure 2c).

**American Sāmoa status** – Present. **Evidence** – Single photographic record. **Distribution** – Tutuila. **Nearest confirmed ecoregion** – Sāmoa, Tuvalu, and Tonga. **Vulnerability** – LC. **Notes** – This species is presented here as a new record (Figure 2c).

######## Genus *Lobophyllia* de Blainville, 1830

***Lobophylliaagaricia* (Milne Edwards & Haime, 1849) (888135)**

*Symphylliaagaricia* Milne Edwards & Haime, 1849 (288082) homotypic synonym. ^CoTW^ Reported – Fenner 2018.

**American Sāmoa status** – Present. **Evidence** – Single photographic record. **Distribution** – Rose Atoll, Tutuila. **Nearest confirmed ecoregion** – Sāmoa, Tuvalu, and Tonga.

***Lobophylliacorymbosa* (Forskål, 1775) (207391)** ^CoTW^

*Lobophylliacorymbosa* (Forskål, 1775) (207391). ^CoTW^ Reported – Lamberts 1983; Birkeland et al. 1987; Maragos et al. 1994; Green and Hunter 1998; Craig et al. 2001; Fisk and Birkeland 2002; Coles et al. 2003; Birkeland 2007a; Kenyon et al. 2010; Corals NPAS 2016. Referenced – Green et al. 1999; Coles et al. 2003; DiDonato et al. 2006; Birkeland 2007a, 2007b; Lovell and McLardy 2008.

**American Sāmoa status** – Present. **Evidence** – Single specimen report (identified by A Lamberts). **Distribution** – American Sāmoa, Aunuʻu, Manuʻa Islands, Ofu, Ofu/Olosega, Olosega, Rose Atoll, Tutuila. **Nearest confirmed ecoregion** – Sāmoa, Tuvalu, and Tonga. **Vulnerability** – LC.

***Lobophylliacostata* (Dana, 1846) (207393)**

*Lobophylliacostata* (Dana, 1846) (207393). Reported – USACE 1980; Lamberts 1983; Birkeland et al. 1987. Referenced – Green et al. 1999; Coles et al. 2003.

**American Sāmoa status** – Present. **Evidence** – Single specimen report (identified by A Lamberts). **Distribution** – American Sāmoa, Tutuila. **Nearest confirmed ecoregion** – Not available. **Notes** – Lamberts (1983) reported this species as common. Veron et al. (2019) report this species as a synonym of *Lobophylliahemprichii* (Ehrenberg, 1834).

***Lobophylliahemprichii* (Ehrenberg, 1834) (207392)** ^CoTW^

*Lobophylliahemprichi* (Ehrenberg, 1834) (207392) [sic]. Reported – Hunter et al. 1993; Green and Hunter 1998.

*Lobophylliahemprichii* (Ehrenberg, 1834) (207392). ^CoTW^ Reported – Birkeland et al. 1987, 2003; Maragos et al. 1994, 1995; Mundy 1996; Mundy and Green 1999; Craig et al. 2001; Fisk and Birkeland 2002; Work and Rameyer 2002; Coles et al. 2003; DiDonato et al. 2006; Birkeland 2007a; Fenner et al. 2008; Kenyon et al. 2010; Corals NPAS 2016; Fenner and Sudek 2016; DMWR 2018; Fenner 2018; Montgomery et al. 2019. Referenced – Green et al. 1999; Fisk and Birkeland 2002; Coles et al. 2003; DiDonato et al. 2006; Birkeland 2007a, 2007b; Lovell and McLardy 2008.

**American Sāmoa status** – Present. **Evidence** – Single specimen report (identifier unknown). **Distribution** – American Sāmoa, Aunuʻu, Manuʻa Islands, Ofu, Ofu/Olosega, Olosega, Rose Atoll, Taʻū, Tutuila. **Nearest confirmed ecoregion** – Sāmoa, Tuvalu, and Tonga. **Vulnerability** – LC. **Mesophotic record** – 48 m depth (Montgomery et al. 2019).

***Lobophylliaishigakiensis* (Veron, 1990) (888146)**

*Acanthastreaishigakiensis* Veron, 1990 (288879) homotypic synonym. ^CoTW^ Reported – Fenner 2018. Referenced – Kenyon et al. 2011.

**American Sāmoa status** – Present. **Evidence** – Single photographic record. **Distribution** – Tutuila. **Nearest confirmed ecoregion** – Sāmoa, Tuvalu, and Tonga.

***Lobophylliarecta* (Dana, 1846) (888140)**

*Symphilliarecta* (Dana, 1846) (207399) [sic] homotypic synonym. Reported – CRED 2011.

*Symphyllianobilis* (Dana, 1846) (207396) heterotypic synonym. Reported – Hoffmeister 1925; USACE 1980; Lamberts 1983; NMNH 2018.

*Symphylliarecta* (Dana, 1846) (207399) homotypic synonym. ^CoTW^ Reported – Birkeland et al. 1987; Maragos et al. 1994; Mundy 1996; Green and Hunter 1998; Craig et al. 2001; Fisk and Birkeland 2002; Coles et al. 2003; Birkeland 2007a; Corals NPAS 2016. Referenced – Green et al. 1999; Coles et al. 2003; DiDonato et al. 2006; Birkeland 2007a, 2007b; Lovell and McLardy 2008.

**American Sāmoa status** – Present. **Evidence** – Multiple specimen reports. **Distribution** – American Sāmoa, Aunuʻu, Manuʻa Islands, Ofu, Ofu/Olosega, Olosega, Taʻū, Tutuila. **Nearest confirmed ecoregion** – Sāmoa, Tuvalu, and Tonga.

######## Genus *Oxypora* Saville-Kent, 1871

***Oxyporacrassispinosa* Nemenzo, 1979 (288351)** ^CoTW^

*Oxyporacrassispinosa* Nemenzo, 1979 (288351). ^CoTW^ Reported – DMWR 2018; Fenner 2018; Montgomery et al. 2019.

**American Sāmoa status** – Present. **Evidence** – Multiple specimen reports. **Distribution** – Tutuila. **Nearest confirmed ecoregion** – Sāmoa, Tuvalu, and Tonga. **Vulnerability** – LC. **Mesophotic record** – 47 m depth (Montgomery et al. 2019). **Notes** – This species sensu Veron (2000) appears to be different, suggesting the original description and type need to be examined.

***Oxyporalacera* (Verrill, 1864) (207374)** ^CoTW^

*Oxyporalacera* (Verrill, 1864) (207374). ^CoTW^ Reported – USACE 1980; Lamberts 1983; Birkeland et al. 1987; Maragos et al. 1994; Mundy 1996; Mundy and Green 1999; Fisk and Birkeland 2002; Coles et al. 2003; Corals NPAS 2016; DMWR 2018; Fenner 2018; NMNH 2018. Referenced – Fisk and Birkeland 2002; Coles et al. 2003; DiDonato et al. 2006; Birkeland 2007a, 2007b; Lovell and McLardy 2008.

**American Sāmoa status** – Present. **Evidence** – Multiple specimen reports. **Distribution** – American Sāmoa, Aunuʻu, Manuʻa Islands, Ofu, Ofu/Olosega, Olosega, Taʻū, Tutuila. **Nearest confirmed ecoregion** – Sāmoa, Tuvalu, and Tonga. **Vulnerability** – LC. **Mesophotic record** – 43 m depth (Montgomery et al. 2019).

####### Family Merulinidae Verrill, 1865

######## Genus *Astrea* Lamarck, 1801

***Astreaannuligera* Milne Edwards & Haime, 1849 (762420)** ^CoTW^

*Astreaannuligera* Milne Edwards & Haime, 1849 (762420). ^CoTW^ Reported – Fenner 2018; NMNH 2018.

*Montastraeaannuligera* (Milne Edwards & Haime, 1849) (207484) homotypic synonym. Reported – Fisk and Birkeland 2002; Corals NPAS 2016. Referenced – Green et al. 1999; Fisk and Birkeland 2002; DiDonato et al. 2006; Birkeland 2007b.

*Montastreaannuligera* (Milne Edwards & Haime, 1849) (764065) homotypic synonym, wrong genus spelling. Reported – Birkeland et al. 1987, 2003; Mundy 1996; Fenner et al. 2008; Kenyon et al. 2010; DMWR 2018. Referenced – Coles et al. 2003; Lovell and McLardy 2008.

**American Sāmoa status** – Present. **Evidence** – Multiple specimen reports. **Distribution** – American Sāmoa, Aunuʻu, Ofu/Olosega, Rose Atoll, Taʻū, Tutuila. **Nearest confirmed ecoregion** – Sāmoa, Tuvalu, and Tonga.

***Astreacurta* Dana, 1846 (762421)** ^CoTW^

*Astreacurta* Dana, 1846 (762421). ^CoTW^ Reported – Fenner 2018; NMNH 2018; Montgomery et al. 2019.

*Montastraeacurta* (Dana, 1846) (207481) homotypic synonym. Reported – Craig et al. 2001; Fisk and Birkeland 2002; DiDonato et al. 2006; Birkeland 2007a; Fenner et al. 2008; Corals NPAS 2016. Referenced – Green et al. 1999; Fisk and Birkeland 2002; DiDonato et al. 2006; Birkeland 2007a, 2007b.

*Montastreacurta* (Dana, 1846) (764064) homotypic synonym, wrong genus spelling. Reported – USACE 1980; Lamberts 1983; Birkeland et al. 1987, 2003; Maragos et al. 1994, 1995; Mundy 1996; Green and Hunter 1998; Coles et al. 2003; Fenner et al. 2008; Kenyon et al. 2010; CRED 2011; Fenner and Sudek 2016; DMWR 2018. Referenced – Coles et al. 2003; Lovell and McLardy 2008.

*Orbicellacurta* Dana, 1846 (766045) homotypic synonym. Reported – Hoffmeister 1925; Lamberts 1983.

**American Sāmoa status** – Present. **Evidence** – Multiple specimen reports. **Distribution** – American Sāmoa, Aunuʻu, Manuʻa Islands, Ofu, Ofu/Olosega, Olosega, Rose Atoll, Taʻū, Tutuila. **Nearest confirmed ecoregion** – Sāmoa, Tuvalu, and Tonga. **Mesophotic record** – 42 m depth (Montgomery et al. 2019).

######## Genus *Caulastraea* Dana, 1846

***Caulastraeafurcata* Dana, 1846 (289577)**

*Caulastreafurcata* Dana, 1846 (207412) wrong genus spelling. ^CoTW^ Reported – Birkeland et al. 1987; Mundy 1996; Fisk and Birkeland 2002; Coles et al. 2003; Fenner et al. 2008; DMWR 2018. Referenced – Green et al. 1999; Fisk and Birkeland 2002; Coles et al. 2003; Birkeland 2007b; Lovell and McLardy 2008.

*Caulastreafurreata* Dana, 1846 (207412) [sic] wrong genus spelling. Reported – Birkeland et al. 2003.

**American Sāmoa status** – Present. **Evidence** – Single specimen report (identified by J Wolstenholme). **Distribution** – American Sāmoa, Manuʻa Islands, Tutuila. **Nearest confirmed ecoregion** – Sāmoa, Tuvalu, and Tonga.

######## Genus *Coelastrea* Verrill, 1866

***Coelastreapalauensis* (Yabe & Sugiyama, 1936) (762428)**

*Goniastreapalauenis* (Yabe & Sugiyama, 1936) (207458) [sic] homotypic synonym. Reported – Green and Hunter 1998.

*Goniastreapalauensis* (Yabe & Sugiyama, 1936) (207458) homotypic synonym. ^CoTW^ Reported – USACE 1980; Lamberts 1983; Maragos et al. 1994. Referenced – Birkeland 2007b.

**American Sāmoa status** – Present. **Evidence** – Single specimen report (identified by A Lamberts). **Distribution** – American Sāmoa, Tutuila. **Nearest confirmed ecoregion** – New Caledonia. **Geographical range extension** – East. **Mesophotic record** – 30 m depth (Lamberts 1983).

######## Genus *Cyphastrea* Milne Edwards & Haime, 1848

***Cyphastreachalcidicum* (Forskål, 1775) (207415)** ^CoTW^

*Cyphastreachalcidicum* (Forskål, 1775) (207415). ^CoTW^ Reported – Lamberts 1983; Maragos et al. 1994; Mundy 1996; Birkeland et al. 2003; Coles et al. 2003; Kenyon et al. 2010; Corals NPAS 2016. Referenced – Green et al. 1999; Fisk and Birkeland 2002; Coles et al. 2003; DiDonato et al. 2006; Birkeland 2007a, 2007b; Lovell and McLardy 2008.

*Cyphastreachalcidium* (Forskål, 1775) (207415) [sic]. Reported – Fisk and Birkeland 2002.

*Cyphastreachalcidium* ? (Forskål, 1775) (207415) [sic]. Reported – DMWR 2018.

**American Sāmoa status** – Present. **Evidence** – Single specimen report (identified by A Lamberts). **Distribution** – American Sāmoa, Aunuʻu, Manuʻa Islands, Ofu, Ofu/Olosega, Olosega, Rose Atoll, Taʻū, Tutuila. **Nearest confirmed ecoregion** – Sāmoa, Tuvalu, and Tonga. **Vulnerability** – LC. **Mesophotic record** – 30 m depth (Lamberts 1983).

***Cyphastreamicrophthalma* (Lamarck, 1816) (207416)** ^CoTW^

*Cyphastreagardineri* cf. Matthai, 1914 (766209) heterotypic synonym. Reported – Lamberts 1983.

*Cyphastreamicrophthalma* (Lamarck, 1816) (207416). ^CoTW^ Reported – Hoffmeister 1925; USACE 1980; Lamberts 1983; Birkeland et al. 1987; Maragos et al. 1994; Craig et al. 2001; Fisk and Birkeland 2002; Coles et al. 2003; Birkeland 2007a; Kenyon et al. 2010; Corals NPAS 2016; BPBM 2018; DMWR 2018; NMNH 2018. Referenced – Fisk and Birkeland 2002; Coles et al. 2003; DiDonato et al. 2006; Birkeland 2007a, 2007b; Lovell and McLardy 2008.

*Cyphastreamicropthalma* (Lamarck, 1816) (207416) [sic]. Reported – Fisk and Birkeland 2002.

*Cyphastreamicropthalma* cf. (Lamarck, 1816) (207416) [sic]. Reported – Hunter et al. 1993.

**American Sāmoa status** – Present. **Evidence** – Multiple specimen reports. **Distribution** – American Sāmoa, Aunuʻu, Manuʻa Islands, Ofu, Olosega, Rose Atoll, Taʻū, Tutuila. **Nearest confirmed ecoregion** – Sāmoa, Tuvalu, and Tonga. **Vulnerability** – LC. **Mesophotic record** – 33, 35 m depth (Hoffmeister 1925; Lamberts 1983).

######## Genus *Dipsastraea* Blainville, 1830

***Dipsastraeafavus* (Forskål, 1775) (718748)**

*Dipsastraeafavus* (Forskål, 1775) (718748). Reported – NMNH 2018.

*Faviafavulus* (Forskål, 1775) (207435) [sic] homotypic synonym. Reported – Fisk and Birkeland 2002.

*Faviafavus* (Forskål, 1775) (207435) homotypic synonym. ^CoTW^ Reported – Hoffmeister 1925; USACE 1980; Lamberts 1983; Birkeland et al. 1987, 2003; Maragos et al. 1994; Mundy 1996; Craig et al. 2001; Fisk and Birkeland 2002; Coles et al. 2003; Birkeland 2007a; Kenyon et al. 2010; CRED 2011; Corals NPAS 2016. Referenced – Green et al. 1999; Fisk and Birkeland 2002; Coles et al. 2003; DiDonato et al. 2006; Birkeland 2007a, 2007b; Lovell and McLardy 2008.

**American Sāmoa status** – Present. **Evidence** – Single specimen report (identified by S Cairns). **Distribution** – American Sāmoa, Manuʻa Islands, Ofu, Ofu/Olosega, Olosega, Rose Atoll, Taʻū, Tutuila. **Nearest confirmed ecoregion** – Sāmoa, Tuvalu, and Tonga.

***Dipsastraealaxa* (Klunzinger, 1879) (758235)**

*Dipsastraealaxa* (Klunzinger, 1879) (758235). Reported – Gross 2019.

*Favialaxa* (Klunzinger, 1879) (207430) homotypic synonym. ^CoTW^ Reported – USACE 1980; Lamberts 1983; Maragos et al. 1994; Mundy 1996; Fisk and Birkeland 2002; Corals NPAS 2016. Referenced – Fisk and Birkeland 2002; Coles et al. 2003; DiDonato et al. 2006; Birkeland 2007a, 2007b; Lovell and McLardy 2008.

**American Sāmoa status** – Present. **Evidence** – Multiple specimen reports. **Distribution** – American Sāmoa, Manuʻa Islands, Ofu, Olosega, Taʻū, Tutuila. **Nearest confirmed ecoregion** – Sāmoa, Tuvalu, and Tonga.

***Dipsastraeamatthaii* (Vaughan, 1918) (758240)**

*Dipsastraeamatthaii* (Vaughan, 1918) (758240). Reported – NMNH 2018; Gross 2019.

*Faviamatthai* ? Vaughan, 1918 (207437) [sic] homotypic synonym. Reported – DMWR 2018.

*Faviamatthai* Vaughan, 1918 (207437) [sic] homotypic synonym. Reported – Green and Hunter 1998; Corals NPAS 2016.

*Faviamatthaii* Vaughan, 1918 (207437) homotypic synonym. ^CoTW^ Reported – Birkeland et al. 1987, 2003; Maragos et al. 1994, 1995; Mundy 1996; Craig et al. 2001; Fisk and Birkeland 2002; Coles et al. 2003; DiDonato et al. 2006; Birkeland 2007a; Kenyon et al. 2010; CRED 2011. Referenced – Green et al. 1999; Fisk and Birkeland 2002; Coles et al. 2003; DiDonato et al. 2006; Birkeland 2007a, 2007b; Lovell and McLardy 2008.

**American Sāmoa status** – Present. **Evidence** – Multiple specimen reports. **Distribution** – American Sāmoa, Aunuʻu, Manuʻa Islands, Ofu, Ofu/Olosega, Olosega, Rose Atoll, Taʻū, Tutuila. **Nearest confirmed ecoregion** – Sāmoa, Tuvalu, and Tonga.

***Dipsastraeapallida* (Dana, 1846) (758233)**

*Dipsastraeapallida* (Dana, 1846) (758233). Reported – NMNH 2018; Gross 2019.

*Faviapallida* (Dana, 1846) (207440) homotypic synonym. ^CoTW^ Reported – Hoffmeister 1925; USACE 1980; Lamberts 1983; Birkeland et al. 1987, 2003; Maragos et al. 1994; Mundy 1996; Green and Hunter 1998; Craig et al. 2001; Fisk and Birkeland 2002; Coles et al. 2003; DiDonato et al. 2006; Birkeland 2007a; Fenner et al. 2008; Kenyon et al. 2010; CRED 2011; Corals NPAS 2016; DMWR 2018. Referenced – Green et al. 1999; Fisk and Birkeland 2002; Coles et al. 2003; DiDonato et al. 2006; Birkeland 2007a, 2007b; Lovell and McLardy 2008.

**American Sāmoa status** – Present. **Evidence** – Multiple specimen reports. **Distribution** – American Sāmoa, Aunuʻu, Manuʻa Islands, Ofu, Ofu/Olosega, Olosega, Rose Atoll, Taʻū, Tutuila. **Nearest confirmed ecoregion** – Sāmoa, Tuvalu, and Tonga.

***Dipsastraearotumana* (Gardiner, 1899) (758237)**

*Dipsastraearotumana* (Gardiner, 1899) (758237). Reported – NMNH 2018; Gross 2019.

*Faviarotumana* (Gardiner, 1899) (207438) homotypic synonym. ^CoTW^ Reported – Hoffmeister 1925; USACE 1980; Lamberts 1983; Birkeland et al. 1987, 2003; Coles et al. 2003; Kenyon et al. 2010; Corals NPAS 2016. Referenced – Green et al. 1999; Coles et al. 2003; DiDonato et al. 2006; Birkeland 2007b; Lovell and McLardy 2008.

*Favitesrotumana* (Gardiner, 1899) (207438) [sic] homotypic synonym. Reported – USACE 1980.

**American Sāmoa status** – Present. **Evidence** – Multiple specimen reports. **Distribution** – American Sāmoa, Aunuʻu, Rose Atoll, Tutuila. **Nearest confirmed ecoregion** – Sāmoa, Tuvalu, and Tonga.

***Dipsastraeaspeciosa* (Dana, 1846) (758219)**

*Dipsastraeaspeciosa* (Dana, 1846) (758219). Reported – NMNH 2018.

*Faviaspeciosa* (Dana, 1846) (207425) homotypic synonym. ^CoTW^ Reported – USACE 1980; Lamberts 1983; Maragos et al. 1994; Mundy 1996; Craig et al. 2001; Fisk and Birkeland 2002; Birkeland et al. 2003; Coles et al. 2003; Birkeland 2007a; Kenyon et al. 2010; Corals NPAS 2016. Referenced – Green et al. 1999; Fisk and Birkeland 2002; Coles et al. 2003; DiDonato et al. 2006; Birkeland 2007a, 2007b; Lovell and McLardy 2008.

**American Sāmoa status** – Present. **Evidence** – Multiple specimen reports. **Distribution** – American Sāmoa, Aunuʻu, Manuʻa Islands, Ofu, Ofu/Olosega, Olosega, Rose Atoll, Taʻū, Tutuila. **Nearest confirmed ecoregion** – Sāmoa, Tuvalu, and Tonga.

***Dipsastraeatruncata* (Veron, 2000) (758228)**

*Faviatruncatus* Veron, 2000 (288076) homotypic synonym, wrong species spelling. ^CoTW^ Reported – Fenner 2018.

**American Sāmoa status** – Present. **Evidence** – Single photographic record. **Distribution** – Ofu/Olosega. **Nearest confirmed ecoregion** – Fiji. **Geographical range extension** – East although Veron et al. (2019) strongly predicted the presence of this species in the Sāmoa, Tuvalu, and Tonga ecoregion.

######## Genus *Echinopora* Lamarck, 1816

***Echinoporagemmacea* (Lamarck, 1816) (207418)** ^CoTW^

*Echinoporagemmacea* (Lamarck, 1816) (207418). ^CoTW^ Reported – Coles et al. 2003; DiDonato et al. 2006; Fenner et al. 2008; CRED 2011; Corals NPAS 2016; DMWR 2018; Paulay and Brown 2019. Referenced – DiDonato et al. 2006; Lovell and McLardy 2008.

*Echinoporagemmacea* ? (Lamarck, 1816) (207418). ^CoTW^ Reported – DMWR 2018.

**American Sāmoa status** – Present. **Evidence** – Multiple specimen reports. **Distribution** – American Sāmoa, Ofu, Ofu/Olosega, Taʻū, Tutuila. **Nearest confirmed ecoregion** – Sāmoa, Tuvalu, and Tonga. **Vulnerability** – LC. **Notes** – The photographed specimen in Corals NPAS (2016) appears to belong to *Echinoporahirsutissima* Milne Edwards & Haime, 1849.

***Echinoporahirsutissima* Milne Edwards & Haime, 1849 (207420)** ^CoTW^

*Echinoporahirsutissima* ? Milne Edwards & Haime, 1849 (207420). ^CoTW^ Reported – Coles et al. 2003.

*Echinoporahirsutissima* Milne Edwards & Haime, 1849 (207420). ^CoTW^ Reported – Birkeland et al. 1987, 2003; Mundy 1996; Fisk and Birkeland 2002; DiDonato et al. 2006; Birkeland 2007a; Fenner et al. 2008; Corals NPAS 2016; DMWR 2018; Fenner 2018. Referenced – Green et al. 1999; Fisk and Birkeland 2002; Coles et al. 2003; DiDonato et al. 2006; Birkeland 2007a, 2007b; Lovell and McLardy 2008.

**American Sāmoa status** – Present. **Evidence** – Single specimen report (identified by D Fenner and J Wolstenholme). **Distribution** – American Sāmoa, Aunuʻu, Manuʻa Islands, Ofu, Ofu/Olosega, Olosega, Rose Atoll, Taʻū, Tutuila. **Nearest confirmed ecoregion** – Sāmoa, Tuvalu, and Tonga. **Vulnerability** – LC.

***Echinoporalamellosa* (Esper, 1795) (207421)** ^CoTW^

*Echinoporalamellosa* (Esper, 1795) (207421). ^CoTW^ Reported – USACE 1980; Lamberts 1983; Birkeland et al. 1987; Maragos et al. 1994, 1995; Mundy 1996; Fisk and Birkeland 2002; Work and Rameyer 2002; Coles et al. 2003; DiDonato et al. 2006; Birkeland 2007a; Fenner et al. 2008; Kenyon et al. 2010; Corals NPAS 2016; DMWR 2018; Fenner 2018. Referenced – Green et al. 1999; Fisk and Birkeland 2002; Coles et al. 2003; DiDonato et al. 2006; Birkeland 2007a, 2007b; Lovell and McLardy 2008.

*Echinopralamellosa* (Esper, 1795) (207421) [sic]. Reported – CRED 2011.

**American Sāmoa status** – Present. **Evidence** – Multiple specimen reports. **Distribution** – American Sāmoa, Aunuʻu, Manuʻa Islands, Ofu, Ofu/Olosega, Olosega, Rose Atoll, Taʻū, Tutuila. **Nearest confirmed ecoregion** – Sāmoa, Tuvalu, and Tonga. **Vulnerability** – LC.

######## Genus *Favites* Link, 1807

***Favitesabdita* (Ellis & Solander, 1786) (207449)** ^CoTW^

*Favitesabdita* (Ellis & Solander, 1786) (207449). ^CoTW^ Reported – Mayor 1924b; Hoffmeister 1925; USACE 1980; Lamberts 1983; Birkeland et al. 1987, 2003; Maragos et al. 1994; Mundy 1996; Green and Hunter 1998; Craig et al. 2001; Fisk and Birkeland 2002; Coles et al. 2003; DiDonato et al. 2006; Birkeland 2007a; Fenner et al. 2008; Kenyon et al. 2010; CRED 2011; Corals NPAS 2016; DMWR 2018; Fenner 2018; NMNH 2018; Gross 2019. Referenced – Green et al. 1997, 1999; Fisk and Birkeland 2002; Coles et al. 2003; DiDonato et al. 2006; Birkeland 2007a, 2007b; Lovell and McLardy 2008.

**American Sāmoa status** – Present. **Evidence** – Multiple specimen reports. **Distribution** – American Sāmoa, Aunuʻu, Manuʻa Islands, Ofu, Ofu/Olosega, Olosega, Taʻū, Tutuila. **Nearest confirmed ecoregion** – Sāmoa, Tuvalu, and Tonga. **Vulnerability** – NT.

***Faviteschinensis* (Verrill, 1866) (207451)** ^CoTW^

*Faviteschinensis* (Verrill, 1866) (207451). ^CoTW^ Reported – USACE 1980; Lamberts 1983; Maragos et al. 1994; DiDonato et al. 2006; Corals NPAS 2016. Referenced – DiDonato et al. 2006; Lovell and McLardy 2008.

*Faviteschinesis* (Verrill, 1866) (207451) [sic]. Reported – Birkeland 2007a. Referenced – Birkeland 2007b.

**American Sāmoa status** – Present. **Evidence** – Single specimen report (identified by A Lamberts). **Distribution** – American Sāmoa, Manuʻa Islands, Ofu, Tutuila. **Nearest confirmed ecoregion** – Sāmoa, Tuvalu, and Tonga. **Vulnerability** – NT.

***Favitesflexuosa* (Dana, 1846) (207444)** ^CoTW^

*Favitesflexuosa* (Dana, 1846) (207444). ^CoTW^ Reported – Birkeland et al. 1987, 2003; Maragos et al. 1994, 1995; Mundy 1996; Green and Hunter 1998; Craig et al. 2001; Fisk and Birkeland 2002; Coles et al. 2003; DiDonato et al. 2006; Birkeland 2007a; Kenyon et al. 2010; Corals NPAS 2016; DMWR 2018. Referenced – Green et al. 1999; Fisk and Birkeland 2002; Coles et al. 2003; DiDonato et al. 2006; Birkeland 2007a, 2007b; Lovell and McLardy 2008.

**American Sāmoa status** – Present. **Evidence** – Single specimen report (identified by D Fenner). **Distribution** – American Sāmoa, Aunuʻu, Manuʻa Islands, Ofu, Ofu/Olosega, Olosega, Rose Atoll, Taʻū, Tutuila. **Nearest confirmed ecoregion** – Sāmoa, Tuvalu, and Tonga. **Vulnerability** – NT.

***Faviteshalicora* (Ehrenberg, 1834) (207447)** ^CoTW^

*Faviahalicora* (Ehrenberg, 1834) (765790) homotypic synonym. Reported – Green and Hunter 1998.

*Faviteshalicora* (Ehrenberg, 1834) (207447). ^CoTW^ Reported – Hoffmeister 1925; USACE 1980; Lamberts 1983; Birkeland et al. 1987, 2003; Maragos et al. 1994; Mundy 1996; Craig et al. 2001; Fisk and Birkeland 2002; Coles et al. 2003; DiDonato et al. 2006; Birkeland 2007a; Kenyon et al. 2010; Corals NPAS 2016; DMWR 2018; NMNH 2018; Gross 2019. Referenced – Fisk and Birkeland 2002; Coles et al. 2003; DiDonato et al. 2006; Birkeland 2007a, 2007b; Lovell and McLardy 2008.

*Faviteshalicora* cf. (Ehrenberg, 1834) (207447). ^CoTW^ Reported – Birkeland et al. 1987. Referenced – Green et al. 1999; Coles et al. 2003.

**American Sāmoa status** – Present. **Evidence** – Multiple specimen reports. **Distribution** – American Sāmoa, Aunuʻu, Manuʻa Islands, Ofu, Ofu/Olosega, Olosega, Rose Atoll, Taʻū, Tutuila. **Nearest confirmed ecoregion** – Sāmoa, Tuvalu, and Tonga. **Vulnerability** – NT. **Mesophotic record** – 30 m depth (Lamberts 1983).

***Favitesparaflexuosus* Veron, 2000 (288822)**

*Favitesparafl*e*xuosus* Veron, 2000 (288822). Reported – This paper (Figure 2b).

**American Sāmoa status** – Present. **Evidence** – Single photographic record. **Distribution** – Ofu, Olosega, Taʻū, Tutuila. **Nearest confirmed ecoregion** – Solomon Islands and Bougainville. **Geographical range extension** – East. **Notes** – This species is presented here as a new record (Figure 2b). Veron et al. (2019) recognize the spelling of this species as *Favitesparaflexuosa* Veron, 2000 even though the spelling was corrected in ICZN (2011).

***Favitespentagona* (Esper, 1795) (207446)** ^CoTW^

*Favitespe*n*tagona* (Esper, 1795) (207446). ^CoTW^ Reported – Green et al. 1997; Birkeland and Belliveau 2000; Birkeland et al. 2003; Coles et al. 2003; DMWR 2018; Fenner 2018. Referenced – Green et al. 1999; Coles et al. 2003; Birkeland 2007b.

*Favitespentagonia* (Esper, 1795) (207446) [sic]. Reported – CRED 2011.

**American Sāmoa status** – Present. **Evidence** – Single specimen report (identified by D Fenner). **Distribution** – Taʻū, Tutuila. **Nearest confirmed ecoregion** – Sāmoa, Tuvalu, and Tonga. **Vulnerability** – LC.

######## Genus *Goniastrea* Milne Edwards & Haime, 1848

***Goniastreaedwardsi* Chevalier, 1971 (207466)** ^CoTW^

*Goniastreaedwardsi* Chevalier, 1971 (207466). ^CoTW^ Reported – USACE 1980; Lamberts 1983; Birkeland et al. 1987, 2003; Maragos et al. 1994; Mundy 1996; Birkeland and Belliveau 2000; Craig et al. 2001; Fisk and Birkeland 2002; Coles et al. 2003; Birkeland 2007a; CRED 2011; Corals NPAS 2016; DMWR 2018; Fenner 2018; NMNH 2018. Referenced – Green et al. 1999; Fisk and Birkeland 2002; Coles et al. 2003; DiDonato et al. 2006; Birkeland 2007a, 2007b; Lovell and McLardy 2008.

*Goniastreaedwardsii* Chevalier, 1971 (207466) [sic]. Reported – Fisk and Birkeland 2002.

**American Sāmoa status** – Present. **Evidence** – Multiple specimen reports. **Distribution** – American Sāmoa, Aunuʻu, Manuʻa Islands, Ofu, Ofu/Olosega, Olosega, Taʻū, Tutuila. **Nearest confirmed ecoregion** – Sāmoa, Tuvalu, and Tonga. **Vulnerability** – LC.

***Goniastreafavulus* (Dana, 1846) (288026)** ^CoTW^

*Goniastreafavulus* (Dana, 1846) (288026). ^CoTW^ Reported – USACE 1980; Lamberts 1983; Birkeland et al. 1987, 2003; Craig et al. 2001; Fisk and Birkeland 2002; DiDonato et al. 2006; Birkeland 2007a; Corals NPAS 2016; Fenner 2018; Gross 2019. Referenced – Green et al. 1999; Coles et al. 2003; DiDonato et al. 2006; Birkeland 2007a, 2007b; Lovell and McLardy 2008.

*Goniastreafavulus* ? (Dana, 1846) (288026). ^CoTW^ Reported – DMWR 2018.

*Goniastreafavulus* cf. (Dana, 1846) (288026). ^CoTW^ Reported – DMWR 2018.

**American Sāmoa status** – Present. **Evidence** – Multiple specimen reports. **Distribution** – American Sāmoa, Aunuʻu, Manuʻa Islands, Ofu, Ofu/Olosega, Olosega, Taʻū, Tutuila. **Nearest confirmed ecoregion** – Sāmoa, Tuvalu, and Tonga. **Vulnerability** – NT. **Notes** – The photographed specimen of this species in Corals NPAS (2016) appears to be incorrectly identified and should be *Goniastreapectinata* (Ehrenberg, 1834).

***Goniastreaminuta* Veron, 2000 (288027)** ^CoTW^

*Goniastreaminuta* ? Veron, 2000 (288027). ^CoTW^ Reported – DMWR 2018.

*Goniastreaminuta* Veron, 2000 (288027). ^CoTW^ Reported – DiDonato et al. 2006; Corals NPAS 2016; DMWR 2018; Fenner 2018. Referenced – Lovell and McLardy 2008.

**American Sāmoa status** – Present. **Evidence** – Single specimen report (identified by D Fenner). **Distribution** – American Sāmoa, Aunuʻu, Ofu, Ofu/Olosega, Taʻū, Tutuila. **Nearest confirmed ecoregion** – New Caledonia. **Geographical range extension** – East. **Vulnerability** – NT.

***Goniastreapectinata* (Ehrenberg, 1834) (207464)** ^CoTW^

*Goniastreapectinata /retiformis* (Ehrenberg, 1834) (207464). Reported – DMWR 2018.

*Goniastreapectinata* (Ehrenberg, 1834) (207464). ^CoTW^ Reported – Hoffmeister 1925; USACE 1980; Lamberts 1983; Birkeland et al. 1987, 2003; Hunter et al. 1993; Maragos et al. 1994, 1995; Mundy 1996; Green and Hunter 1998; Craig et al. 2001; Fisk and Birkeland 2002; Coles et al. 2003; DiDonato et al. 2006; Birkeland 2007a; Kenyon et al. 2010; CRED 2011; Corals NPAS 2016; DMWR 2018; Fenner 2018; NMNH 2018. Referenced – Green et al. 1999; Fisk and Birkeland 2002; Coles et al. 2003; DiDonato et al. 2006; Birkeland 2007a, 2007b; Lovell and McLardy 2008.

*Goninstreapectinata* (Ehrenberg, 1834) (207464) [sic]. Reported – Hunter et al. 1993.

**American Sāmoa status** – Present. **Evidence** – Multiple specimen reports. **Distribution** – American Sāmoa, Aunuʻu, Manuʻa Islands, Ofu, Ofu/Olosega, Olosega, Rose Atoll, Taʻū, Tutuila. **Nearest confirmed ecoregion** – Sāmoa, Tuvalu, and Tonga. **Vulnerability** – LC. **Notes** – The photographed specimen of this species in Corals NPAS (2016) appears to be incorrectly identified and should be *Goniastrearetiformis* (Lamarck, 1816).

***Goniastrearetiformis* (Lamarck, 1816) (207461)** ^CoTW^

*Goniastrearetifirmis* (Lamarck, 1816) (207461) [sic]. Reported – Mundy 1996.

*Goniastrearetifonnis* (Lamarck, 1816) (207461) [sic]. Reported – Hunter et al. 1993.

*Goniastrearetiformis* (Lamarck, 1816) (207461). ^CoTW^ Reported – Hoffmeister 1925; USACE 1980; Lamberts 1983; Birkeland et al. 1987, 2003; Hunter et al. 1993; Maragos et al. 1994, 1995; Green and Hunter 1998; Craig et al. 2001; Fisk and Birkeland 2002; Coles et al. 2003; Cornish and DiDonato 2004; DiDonato et al. 2006; Birkeland 2007a; Fenner et al. 2008; Kenyon et al. 2010; CRED 2011; Corals NPAS 2016; DMWR 2018; Fenner 2018; NMNH 2018. Referenced – Green et al. 1997, 1999; Fisk and Birkeland 2002; Coles et al. 2003; DiDonato et al. 2006; Birkeland 2007a, 2007b; Lovell and McLardy 2008.

*Goniastrearetortiformis* (Lamarck, 1816) (207461) [sic]. Reported – NMNH 2018.

*Goniostrearetiformis* (Lamarck, 1816) (207461) [sic]. Reported – Hunter et al. 1993.

**American Sāmoa status** – Present. **Evidence** – Multiple specimen reports. **Distribution** – American Sāmoa, Aunuʻu, Manuʻa Islands, Ofu, Ofu/Olosega, Olosega, Rose Atoll, Taʻū, Tutuila. **Nearest confirmed ecoregion** – Sāmoa, Tuvalu, and Tonga. **Vulnerability** – LC. **Notes** – The photographed specimen of this species in Corals NPAS (2016) appears to be incorrectly identified and is likely a *Porites* sp.

***Goniastreastelligera* (Dana, 1846) (763067)**

*Faviastelliger* (Dana, 1846) (207441) [sic] homotypic synonym. Reported – Work and Rameyer 2002.

*Faviastelligera* (Dana, 1846) (207441) homotypic synonym. ^CoTW^ Reported – Hoffmeister 1925; USACE 1980; Lamberts 1983; Birkeland et al. 1987, 2003; Hunter et al. 1993; Maragos et al. 1994, 1995; Mundy 1996; Green and Hunter 1998; Craig et al. 2001; Fisk and Birkeland 2002; Coles et al. 2003; DiDonato et al. 2006; Birkeland 2007a; Fenner et al. 2008; Kenyon et al. 2010; CRED 2011; Corals NPAS 2016; DMWR 2018; Fenner 2018. Referenced – Green et al. 1999; Fisk and Birkeland 2002; Coles et al. 2003; DiDonato et al. 2006; Birkeland 2007a, 2007b; Lovell and McLardy 2008.

*Goniastreastelligera* (Dana, 1846) (763067). Reported – NMNH 2018; Gross 2019; Montgomery et al. 2019.

**American Sāmoa status** – Present. **Evidence** – Multiple specimen reports. **Distribution** – American Sāmoa, Aunuʻu, Manuʻa Islands, Ofu, Ofu/Olosega, Olosega, Rose Atoll, Swains, Taʻū, Tutuila. **Nearest confirmed ecoregion** – Sāmoa, Tuvalu, and Tonga. **Mesophotic record** – 43 m depth (Montgomery et al. 2019).

######## Genus *Hydnophora* Fischer von Waldheim, 1807

***Hydnophoraexesa* (Pallas, 1766) (207403)** ^CoTW^

*Hydnophoraexaesa* (Pallas, 1766) (207403) [sic]. Reported – Fisk and Birkeland 2002.

*Hydnophoraexesa* (Pallas, 1766) (207403). ^CoTW^ Reported – USACE 1980; Lamberts 1983; Birkeland et al. 1987, 2003; Itano and Buckley 1988; Hunter et al. 1993; Maragos et al. 1994; Mundy 1996; Green and Hunter 1998; Craig et al. 2001; Fisk and Birkeland 2002; Coles et al. 2003; DiDonato et al. 2006; Birkeland 2007a; Fenner et al. 2008; Kenyon et al. 2010; CRED 2011; Corals NPAS 2016; DMWR 2018; Fenner 2018; NMNH 2018; Montgomery et al. 2019. Referenced – Green et al. 1999; Fisk and Birkeland 2002; Coles et al. 2003; DiDonato et al. 2006; Birkeland 2007a, 2007b; Lovell and McLardy 2008.

*Hydrophoraexesa* (Pallas, 1766) (207403) [sic]. Reported – Birkeland et al. 2003.

**American Sāmoa status** – Present. **Evidence** – Multiple specimen reports. **Distribution** – American Sāmoa, Aunuʻu, Manuʻa Islands, Ofu, Ofu/Olosega, Olosega, Rose Atoll, Taʻū, Tutuila. **Nearest confirmed ecoregion** – Sāmoa, Tuvalu, and Tonga. **Vulnerability** – NT. **Mesophotic record** – 30, 38 m depth (Lamberts 1983; Montgomery et al. 2019).

***Hydnophoramicroconos* (Lamarck, 1816) (207402)** ^CoTW^

*Hydnophoramicroconnos* (Lamarck, 1816) (207402) [sic]. Reported – CRED 2011.

*Hydnophoramicroconos grade rigida* (Lamarck, 1816) (207402). Reported – Hoffmeister 1925.

*Hydnophoramicroconos* (Lamarck, 1816) (207402). ^CoTW^ Reported – Mayor 1924a; Hoffmeister 1925; USACE 1980; Lamberts 1983; Birkeland et al. 1987, 2003; Itano and Buckley 1988; Maragos et al. 1994, 1995; Craig et al. 2001; Fisk and Birkeland 2002; Work and Rameyer 2002; Coles et al. 2003; DiDonato et al. 2006; Birkeland 2007a; Fenner et al. 2008; Corals NPAS 2016; DMWR 2018; Fenner 2018; NMNH 2018. Referenced – Green et al. 1997, 1999; Coles et al. 2003; DiDonato et al. 2006; Birkeland 2007a, 2007b; Lovell and McLardy 2008.

*Hydnophoramicroconus* (Lamarck, 1816) (207402) [sic]. Reported – Hunter et al. 1993; Green and Hunter 1998.

**American Sāmoa status** – Present. **Evidence** – Multiple specimen reports. **Distribution** – American Sāmoa, Aunuʻu, Manuʻa Islands, Ofu, Ofu/Olosega, Taʻū, Tutuila. **Nearest confirmed ecoregion** – Sāmoa, Tuvalu, and Tonga. **Vulnerability** – NT. **Mesophotic record** – 30 m depth (Lamberts 1983).

***Hydnophorarigida* (Dana, 1846) (207406)** ^CoTW^

*Hydnophorarigida* (Dana, 1846) (207406). ^CoTW^ Reported – Birkeland et al. 1987, 2003; Maragos et al. 1994, 1995; Mundy 1996; Fisk and Birkeland 2002; DiDonato et al. 2006; Fenner et al. 2008; Corals NPAS 2016; DMWR 2018; Fenner 2018. Referenced – Green et al. 1999; Fisk and Birkeland 2002; Coles et al. 2003; DiDonato et al. 2006; Birkeland 2007b; Lovell and McLardy 2008.

**American Sāmoa status** – Present. **Evidence** – Single specimen report (identified by J Wolstenholme). **Distribution** – American Sāmoa, Aunuʻu, Manuʻa Islands, Ofu, Tutuila. **Nearest confirmed ecoregion** – Sāmoa, Tuvalu, and Tonga. **Vulnerability** – LC. **Notes** – The photographed coral of this species in Corals NPAS (2016) appears to be incorrectly identified.

######## Genus *Leptoria* Milne Edwards & Haime, 1848

***Leptoriaphrygia* (Ellis & Solander, 1786) (207477)** ^CoTW^

*Leptoriaphrygia /gracilis* (Ellis & Solander, 1786) (207477). Referenced – Green et al. 1997.

*Leptoriaphrygia grade gracilis* cf. (Ellis & Solander, 1786) (207477). Reported – Hoffmeister 1925.

*Leptoriaphrygia* (Ellis & Solander, 1786) (207477). ^CoTW^ Reported – Hoffmeister 1925; USACE 1980; Lamberts 1983; Birkeland et al. 1987, 2003; Itano and Buckley 1988; Hunter et al. 1993; Maragos et al. 1994, 1995; Mundy 1996; Green and Hunter 1998; Craig et al. 2001; Fisk and Birkeland 2002; Work and Rameyer 2002; Coles et al. 2003; DiDonato et al. 2006; Birkeland 2007a; Fenner et al. 2008; Kenyon et al. 2010; Corals NPAS 2016; Fenner and Sudek 2016; DMWR 2018; Fenner 2018; NMNH 2018; Montgomery et al. 2019. Referenced – Green et al. 1999; Fisk and Birkeland 2002; Coles et al. 2003; DiDonato et al. 2006; Birkeland 2007a, 2007b; Lovell and McLardy 2008.

*Leptoriaphyrgia* (Ellis & Solander, 1786) (207477) [sic]. Reported – Hunter et al. 1993.

*Leptoriaphyrygia* (Ellis & Solander, 1786) (207477) [sic]. Reported – Fisk and Birkeland 2002.

*Leptoriatenuis* (Dana, 1846) (367855) heterotypic synonym. Reported – Hoffmeister 1925; Lamberts 1983; NMNH 2018.

**American Sāmoa status** – Present. **Evidence** – Multiple specimen reports. **Distribution** – American Sāmoa, Aunuʻu, Manuʻa Islands, Ofu, Ofu/Olosega, Olosega, Taʻū, Tutuila. **Nearest confirmed ecoregion** – Sāmoa, Tuvalu, and Tonga. **Vulnerability** – NT. **Mesophotic record** – 30, 42 m depth (Lamberts 1983; Montgomery et al. 2019).

######## Genus *Merulina* Ehrenberg, 1834

***Merulinaampliata* (Ellis & Solander, 1786) (207407)** ^CoTW^

*Merulinaampliata* (Ellis & Solander, 1786) (207407). ^CoTW^ Reported – USACE 1980; Lamberts 1983; Birkeland et al. 1987; Maragos et al. 1994; Mundy 1996; Mundy and Green 1999; Fisk and Birkeland 2002; Coles et al. 2003; DiDonato et al. 2006; Birkeland 2007a; Fenner et al. 2008; Kenyon et al. 2010; CRED 2011; Corals NPAS 2016; DMWR 2018; Fenner 2018; Montgomery et al. 2019. Referenced – Green et al. 1999; Fisk and Birkeland 2002; Coles et al. 2003; DiDonato et al. 2006; Birkeland 2007a, 2007b; Lovell and McLardy 2008.

*Merulinavaughani* Van der Horst, 1921 (758411) heterotypic synonym. Reported – Hoffmeister 1925; Birkeland et al. 1987; NMNH 2018. Referenced – Green et al. 1999; Coles et al. 2003.

**American Sāmoa status** – Present. **Evidence** – Multiple specimen reports. **Distribution** – American Sāmoa, Aunuʻu, Manuʻa Islands, Ofu, Ofu/Olosega, Olosega, Rose Atoll, Taʻū, Tutuila. **Nearest confirmed ecoregion** – Sāmoa, Tuvalu, and Tonga. **Vulnerability** – LC. **Mesophotic record** – 42 m depth (Montgomery et al. 2019).

***Merulinascabricula* Dana, 1846 (289198)** ^CoTW^

*Merulinascabicula* Dana, 1846 (289198) [sic]. Referenced – Birkeland 2007b.

*Merulinascabricula* Dana, 1846 (289198). ^CoTW^ Reported – Maragos et al. 1994; Green and Hunter 1998; Coles et al. 2003; DiDonato et al. 2006; Fenner et al. 2008; Corals NPAS 2016; DMWR 2018; Montgomery et al. 2019. Referenced – Coles et al. 2003; Lovell and McLardy 2008.

**American Sāmoa status** – Present. **Evidence** – Single specimen report (identified by D Fenner). **Distribution** – American Sāmoa, Aunuʻu, Tutuila. **Nearest confirmed ecoregion** – Sāmoa, Tuvalu, and Tonga. **Vulnerability** – LC. **Mesophotic record** – 39 m depth (Montgomery et al. 2019). **Notes** – Some taxonomic disagreement exists for this genus as Huang et al. (2014) recognize *Paraclavarina* as a synonym of *Merulina* while Veron et al. (2019) do not.

######## Genus *Mycedium* Milne Edwards & Haime, 1851

***Mycediumelephantotus* (Pallas, 1766) (207373)** ^CoTW^

*Mycediumelephantotum* (Pallas, 1766) (207373) [sic]. Reported – Hunter et al. 1993.

*Mycediumelephantotus* (Pallas, 1766) (207373). ^CoTW^ Reported – Birkeland et al. 1987; Itano and Buckley 1988; Maragos et al. 1994; Mundy 1996; Green and Hunter 1998; Fisk and Birkeland 2002; Coles et al. 2003; DiDonato et al. 2006; CRED 2011; Corals NPAS 2016; DMWR 2018; Fenner 2018. Referenced – Fisk and Birkeland 2002; Coles et al. 2003; DiDonato et al. 2006; Birkeland 2007a, 2007b; Lovell and McLardy 2008.

**American Sāmoa status** – Present. **Evidence** – Single specimen report (identified by D Fenner). **Distribution** – American Sāmoa, Aunuʻu, Manuʻa Islands, Ofu, Ofu/Olosega, Taʻū, Tutuila. **Nearest confirmed ecoregion** – Sāmoa, Tuvalu, and Tonga. **Vulnerability** – LC.

***Mycediumrobokaki* Moll & Best, 1984 (287735)** ^CoTW^

*Mycediumrobokakai* Moll & Best, 1984 (287735) [sic]. Reported – Fenner 2018.

*Mycediumrobokaki* Moll & Best, 1984 (287735). ^CoTW^ Reported – Fisk and Birkeland 2002.

**American Sāmoa status** – Present. **Evidence** – Single photographic record. **Distribution** – Manuʻa Islands, Tutuila. **Nearest confirmed ecoregion** – Fiji. **Geographical range extension** – East. **Vulnerability** – LC.

######## Genus *Oulophyllia* Milne Edwards & Haime, 1848

***Oulophylliacrispa* (Lamarck, 1816) (207485)** ^CoTW^

*Oulophylliacrispa /bennettae* (Lamarck, 1816) (207485). Reported – DMWR 2018.

*Oulophylliacrispa* (Lamarck, 1816) (207485). ^CoTW^ Reported – USACE 1980; Lamberts 1983; Birkeland et al. 1987; Maragos et al. 1994; Mundy 1996; Coles et al. 2003; Kenyon et al. 2010; CRED 2011; Corals NPAS 2016; DMWR 2018; Fenner 2018. Referenced – Fisk and Birkeland 2002; Coles et al. 2003; DiDonato et al. 2006; Birkeland 2007a, 2007b; Lovell and McLardy 2008.

**American Sāmoa status** – Present. **Evidence** – Multiple specimen reports. **Distribution** – American Sāmoa, Aunuʻu, Manuʻa Islands, Ofu, Ofu/Olosega, Rose Atoll, Taʻū, Tutuila. **Nearest confirmed ecoregion** – Sāmoa, Tuvalu, and Tonga. **Vulnerability** – NT.

######## Genus *Paragoniastrea* Huang, Benzoni & Budd, 2014

***Paragoniastrearusselli* (Wells, 1954) (817179)**

*Favitesrussell* (Wells, 1954) (207454) [sic] homotypic synonym. Reported – Birkeland et al. 1987.

*Favitesrusselli* (Wells, 1954) (207454) homotypic synonym. ^CoTW^ Reported – USACE 1980; Lamberts 1983; Birkeland et al. 1987, 2003; Maragos et al. 1994; Mundy 1996; Craig et al. 2001; Fisk and Birkeland 2002; Coles et al. 2003; DiDonato et al. 2006; Birkeland 2007a; Kenyon et al. 2010; Corals NPAS 2016. Referenced – Green et al. 1999; Fisk and Birkeland 2002; Coles et al. 2003; DiDonato et al. 2006; Birkeland 2007a, 2007b; Lovell and McLardy 2008.

**American Sāmoa status** – Present. **Evidence** – Single specimen report (identified by A Lamberts). **Distribution** – American Sāmoa, Manuʻa Islands, Ofu, Ofu/Olosega, Olosega, Rose Atoll, Taʻū, Tutuila. **Nearest confirmed ecoregion** – Sāmoa, Tuvalu, and Tonga. **Mesophotic record** – 30 m depth (Lamberts 1983).

######## Genus *Platygyra* Ehrenberg, 1834

***Platygyracontorta* Veron, 1990 (289205)** ^CoTW^

*Platygyracontorta* Veron, 1990 (289205). ^CoTW^ Reported – Fisk and Birkeland 2002; DiDonato et al. 2006; Corals NPAS 2016. Referenced – Lovell and McLardy 2008.

**American Sāmoa status** – Present. **Evidence** – Single photographic record. **Distribution** – American Sāmoa, Ofu. **Nearest confirmed ecoregion** – Sāmoa, Tuvalu, and Tonga. **Vulnerability** – LC.

***Platygyradaedalea* (Ellis & Solander, 1786) (207489)** ^CoTW^

*Meandraesperi* (Milne Edwards & Haime, 1849) (1262040) heterotypic synonym. Reported – Hoffmeister 1925; Lamberts 1983.

*Platygyradadaelea* (Ellis & Solander, 1786) (207489) [sic]. Reported – Fenner et al. 2008.

*Platygyradaedala* (Ellis & Solander, 1786) (207489) [sic]. Reported – Work and Rameyer 2002.

*Platygyradaedalea (esperi)* (Ellis & Solander, 1786) (207489). Reported – Kenyon et al. 2010.

*Platygyradaedalea* (Ellis & Solander, 1786) (207489). ^CoTW^ Reported – Birkeland et al. 1987, 2003; Hunter et al. 1993; Maragos et al. 1994, 1995; Mundy 1996; Green and Hunter 1998; Craig et al. 2001; Fisk and Birkeland 2002; Coles et al. 2003; DiDonato et al. 2006; Birkeland 2007a; Fenner et al. 2008; CRED 2011; Corals NPAS 2016; DMWR 2018; Fenner 2018; NMNH 2018. Referenced – Green et al. 1999; Fisk and Birkeland 2002; Coles et al. 2003; DiDonato et al. 2006; Birkeland 2007a, 2007b; Lovell and McLardy 2008.

*Platygyradaedallea* (Ellis & Solander, 1786) (207489) [sic]. Reported – Hunter et al. 1993.

*Platygyrarustica* (Dana, 1846) (411248) heterotypic synonym. Reported – USACE 1980.

*Platygyrusdaedalea* (Ellis & Solander, 1786) (207489) [sic]. Reported – Lamberts 1983.

*Platygyrusrustica* (Dana, 1846) (411248) [sic] heterotypic synonym. Reported – Lamberts 1983.

**American Sāmoa status** – Present. **Evidence** – Multiple specimen reports. **Distribution** – American Sāmoa, Aunuʻu, Manuʻa Islands, Ofu, Ofu/Olosega, Olosega, Rose Atoll, Taʻū, Tutuila. **Nearest confirmed ecoregion** – Sāmoa, Tuvalu, and Tonga. **Vulnerability** – LC.

***Platygyralamellina* (Ehrenberg, 1834) (207487)** ^CoTW^

*Meandralamellina* Ehrenberg, 1834 (1262039) homotypic synonym. Reported – Hoffmeister 1925.

*Platygyralamellina* (Ehrenberg, 1834) (207487). ^CoTW^ Reported – USACE 1980; Birkeland et al. 1987; Maragos et al. 1994; Birkeland 2007a; Kenyon et al. 2010; NMNH 2018. Referenced – Coles et al. 2003; Birkeland 2007a, 2007b; Lovell and McLardy 2008.

*Platygyralamellina* ? (Ehrenberg, 1834) (207487). ^CoTW^ Reported – Coles et al. 2003.

*Platygyruslamellina* (Ehrenberg, 1834) (207487) [sic]. Reported – Lamberts 1983.

**American Sāmoa status** – Present. **Evidence** – Multiple specimen reports. **Distribution** – American Sāmoa, Ofu, Ofu/Olosega, Rose Atoll, Taʻū, Tutuila. **Nearest confirmed ecoregion** – Sāmoa, Tuvalu, and Tonga. **Vulnerability** – NT.

***Platygyrapini* Chevalier, 1975 (207490)** ^CoTW^

*Platygyiapini* Chevalier, 1975 (207490) [sic]. Reported – Birkeland et al. 2003.

*Platygyrapini* Chevalier, 1975 (207490). ^CoTW^ Reported – Birkeland et al. 1987, 2003; Maragos et al. 1994; Mundy 1996; Green and Hunter 1998; Craig et al. 2001; Fisk and Birkeland 2002; Coles et al. 2003; DiDonato et al. 2006; Birkeland 2007a; CRED 2011; Corals NPAS 2016; DMWR 2018; NMNH 2018. Referenced – Green et al. 1999; Fisk and Birkeland 2002; Coles et al. 2003; DiDonato et al. 2006; Birkeland 2007a, 2007b; Lovell and McLardy 2008.

**American Sāmoa status** – Present. **Evidence** – Multiple specimen reports. **Distribution** – American Sāmoa, Manuʻa Islands, Ofu, Ofu/Olosega, Olosega, Taʻū, Tutuila. **Nearest confirmed ecoregion** – Sāmoa, Tuvalu, and Tonga. **Vulnerability** – LC.

***Platygyrasinensis* (Milne Edwards & Haime, 1849) (207486)** ^CoTW^

*Platygyrasinensis* (Milne Edwards & Haime, 1849) (207486). ^CoTW^ Reported – Maragos et al. 1994, 1995; Mundy 1996; Fisk and Birkeland 2002; DiDonato et al. 2006; Fenner et al. 2008; Kenyon et al. 2010; Corals NPAS 2016. Referenced – Fisk and Birkeland 2002; Coles et al. 2003; DiDonato et al. 2006; Birkeland 2007a, 2007b; Lovell and McLardy 2008.

**American Sāmoa status** – Present. **Evidence** – Single photographic record. **Distribution** – American Sāmoa, Manuʻa Islands, Ofu, Ofu/Olosega, Olosega, Taʻū, Tutuila. **Nearest confirmed ecoregion** – Sāmoa, Tuvalu, and Tonga. **Vulnerability** – LC. **Notes** – The photographed coral of this species in Corals NPAS (2016) appears to be uncertain, but there are multiple other reports of this species.

######## Genus *Scapophyllia* Milne Edwards & Haime, 1848

***Scapophylliacylindrica* Milne Edwards & Haime, 1849 (291024)** ^CoTW^

*Scapophylliacylindrica* Milne Edwards & Haime, 1849 (291024). ^CoTW^ Reported – Maragos et al. 1994; Mundy 1996; Green and Hunter 1998; Coles et al. 2003; Corals NPAS 2016; DMWR 2018; Fenner 2018. Referenced – Fisk and Birkeland 2002; Coles et al. 2003; DiDonato et al. 2006; Birkeland 2007a, 2007b; Lovell and McLardy 2008.

**American Sāmoa status** – Present. **Evidence** – Single specimen report (identified by D Fenner). **Distribution** – American Sāmoa, Aunuʻu, Manuʻa Islands, Ofu, Ofu/Olosega, Olosega, Rose Atoll, Taʻū, Tutuila. **Nearest confirmed ecoregion** – Sāmoa, Tuvalu, and Tonga. **Vulnerability** – LC.

####### Family Plesiastreidae Dai & Horng, 2009

######## Genus *Plesiastrea* Milne Edwards & Haime, 1848

***Plesiastreaversipora* (Lamarck, 1816) (207494)** ^CoTW^

*Pleasiastreaversipora* (Lamarck, 1816) (207494) [sic]. Referenced – Birkeland 2007b.

*Plesiastreaveripora* (Lamarck, 1816) (207494) [sic]. Reported – USACE 1980.

*Plesiastreaversipora* (Lamarck, 1816) (207494). ^CoTW^ Reported – Lamberts 1983; Birkeland et al. 1987; Maragos et al. 1994; Fisk and Birkeland 2002; Coles et al. 2003; DMWR 2018; Fenner 2018; Montgomery et al. 2019. Referenced – Coles et al. 2003; Lovell and McLardy 2008.

**American Sāmoa status** – Present. **Evidence** – Multiple specimen reports. **Distribution** – American Sāmoa, Aunuʻu, Manuʻa Islands, Olosega, Rose Atoll, South Bank, Taʻū, Tutuila. **Nearest confirmed ecoregion** – Sāmoa, Tuvalu, and Tonga. **Vulnerability** – LC. **Mesophotic record** – 43 m depth (Montgomery et al. 2019).

####### Family Pocilloporidae Gray, 1840

######## Genus *Pocillopora* Lamarck, 1816

***Pocilloporaankeli* Scheer & Pillai, 1974 (430671)** ^CoTW^

*Pocilloporaankeli* Scheer & Pillai, 1974 (430671). ^CoTW^ Reported – USACE 1980; Lamberts 1983; Birkeland et al. 1987; Corals NPAS 2016. Referenced – Green et al. 1999; Coles et al. 2003; DiDonato et al. 2006; Birkeland 2007b; Lovell and McLardy 2008.

**American Sāmoa status** – Present. **Evidence** – Single specimen report (identified by A Lamberts). **Distribution** – American Sāmoa, Tutuila. **Nearest confirmed ecoregion** – Solomon Islands and Bougainville. **Geographical range extension** – Southeast. **Vulnerability** – VU.

***Pocilloporabrevicornis* Lamarck, 1816 (206951)** ^CoTW^

*Pocilloporabrevicornis* Lamarck, 1816 (206951). ^CoTW^ Reported – Mayor 1924b; Hoffmeister 1925; USACE 1980; Lamberts 1983; Maragos et al. 1994; Kenyon et al. 2010; NMNH 2018. Referenced – Dahl and Lamberts 1977; Dahl 1981; Green et al. 1997.

*Pocilloporasetcheli* Hoffmeister, 1925 (206967) [sic] heterotypic synonym. Reported – Corals NPAS 2016.

*Pocilloporasetchelli* cf. Hoffmeister, 1925 (206967) heterotypic synonym. Reported – USACE 1980; Lamberts 1983.

*Pocilloporasetchelli* Hoffmeister, 1925 (206967) heterotypic synonym. Reported – USACE 1980; Birkeland et al. 1987, 2003; Coles et al. 2003; Fenner 2018. Referenced – Green et al. 1999; Coles et al. 2003; DiDonato et al. 2006; Birkeland 2007b.

*Pocilloporasetichelli* Hoffmeister, 1925 (206967) [sic] heterotypic synonym. Reported – DMWR 2018.

**American Sāmoa status** – Present. **Evidence** – Type specimen location (synonym *Pocilloporasetchelli*). **Distribution** – American Sāmoa, Aunuʻu, Ofu, Ofu/Olosega, Rose Atoll, Swains, Taʻū, Tutuila. **Nearest confirmed ecoregion** – Sāmoa, Tuvalu, and Tonga.

***Pocilloporadamicornis* (Linnaeus, 1758) (206953)** ^CoTW^

Pocilliporadamicornisvar.cespitosa (Linnaeus, 1758) (206953) [sic]. Reported – Hoffmeister 1925.

*Pocilloporadamicomis* (Linnaeus, 1758) (206953) [sic]. Reported – Hunter et al. 1993.

*Pocilloporadamicorniscaespitosa* Dana, 1846 (545628). Reported – Mayor 1924b; NMNH 2018.

Pocilloporadamicornisvar.bulbosa (Linnaeus, 1758) (206953). Reported – Hoffmeister 1925.

Pocilloporadamicornisvar.cespitosa Dana, 1846 (818848). Reported – Mayor 1924b; Hoffmeister 1925. Referenced – Green et al. 1997.

*Pocilloporadamicornis* (Linnaeus, 1758) (206953). ^CoTW^ Reported – Mayor 1924a; Hoffmeister 1925; USACE 1980; Lamberts 1983; Birkeland et al. 1987, 2003, 2013; Hunter et al. 1993; Maragos et al. 1994, 1995; Mundy 1996; Green et al. 1997; Birkeland and Belliveau 2000; Craig et al. 2001; Fisk and Birkeland 2002; Coles et al. 2003; Cornish and DiDonato 2004; DiDonato et al. 2006; Birkeland 2007a; Fenner et al. 2008; Kenyon et al. 2010; CRED 2011; Corals NPAS 2016; Fenner and Sudek 2016; DMWR 2018; Fenner 2018; NMNH 2018; Montgomery et al. 2019; Paulay and Brown 2019. Referenced – Dahl and Lamberts 1977; Dahl 1981; Green et al. 1997, 1999; Fisk and Birkeland 2002; Coles et al. 2003; DiDonato et al. 2006; Birkeland 2007a, 2007b; Lovell and McLardy 2008.

**American Sāmoa status** – Present. **Evidence** – Multiple specimen reports. **Distribution** – American Sāmoa, Aunuʻu, Manuʻa Islands, Ofu, Ofu/Olosega, Olosega, Rose Atoll, Taʻū, Tutuila. **Nearest confirmed ecoregion** – Sāmoa, Tuvalu, and Tonga. **Vulnerability** – LC. **Mesophotic record** – 50 m depth (Montgomery et al. 2019). **Notes** – See note for *P.acuta*.

***Pocilloporaelegans* Dana, 1846 (206956)** ^CoTW^

*Pocilloporaelegans* ? Dana, 1846 (206956). ^CoTW^ Reported – DMWR 2018.

*Pocilloporaelegans* Dana, 1846 (206956). ^CoTW^ Reported – Birkeland et al. 1987, 2003; Coles et al. 2003; CRED 2011; Corals NPAS 2016; DMWR 2018. Referenced – Green et al. 1999; Coles et al. 2003; DiDonato et al. 2006; Birkeland 2007b; Lovell and McLardy 2008; Kenyon et al. 2011.

**American Sāmoa status** – Present. **Evidence** – Single specimen report (identified by D Fenner). **Distribution** – American Sāmoa, Aunuʻu, Rose Atoll, Swains, Tutuila. **Nearest confirmed ecoregion** – Sāmoa, Tuvalu, and Tonga. **Vulnerability** – VU.

***Pocilloporagrandis* Dana, 1846 (206952)**

*Pocilloporaedouxi* Milne Edwards, 1860 (206958) [sic] heterotypic synonym. Reported – Fenner et al. 2008.

*Pocilloporaeydouxi* cf. Milne Edwards, 1860 (206958) heterotypic synonym. ^CoTW^ Reported – USACE 1980.

*Pocilloporaeydouxi* Milne Edwards, 1860 (206958) heterotypic synonym. ^CoTW^ Reported – Mayor 1924b; Hoffmeister 1925; USACE 1980; Lamberts 1983; Birkeland et al. 1987, 2003; Hunter et al. 1993; Maragos et al. 1994, 1995; Mundy 1996; Green and Hunter 1998; Birkeland and Belliveau 2000; Craig et al. 2001; Fisk and Birkeland 2002; Work and Rameyer 2002; Coles et al. 2003; DiDonato et al. 2006; Birkeland 2007a; Fenner et al. 2008; Kenyon et al. 2010; CRED 2011; Corals NPAS 2016; DMWR 2018; Fenner 2018; NMNH 2018. Referenced – Dahl and Lamberts 1977; Dahl 1981; Green et al. 1997, 1999; Fisk and Birkeland 2002; Coles et al. 2003; DiDonato et al. 2006; Birkeland 2007a, 2007b; Lovell and McLardy 2008.

*Pocilloporaeydoxi* Milne Edwards, 1860 (206958) [sic] heterotypic synonym. Reported – Birkeland et al. 1987.

*Pocilloporagrandis* Dana, 1846 (206952). Reported – QM 2018; Montgomery et al. 2019.

**American Sāmoa status** – Present. **Evidence** – Multiple specimen reports. **Distribution** – American Sāmoa, Aunuʻu, Manuʻa Islands, Ofu, Ofu/Olosega, Olosega, Rose Atoll, Swains, Taʻū, Tutuila. **Nearest confirmed ecoregion** – Sāmoa, Tuvalu, and Tonga. **Mesophotic record** – 49 m depth (Montgomery et al. 2019).

***Pocilloporaligulata* Dana, 1846 (206959)** ^CoTW^

*Pocilloporaligulata* Dana, 1846 (206959). ^CoTW^ Reported – Birkeland et al. 1987, 2003; Coles et al. 2003; Corals NPAS 2016; DMWR 2018; Fenner 2018. Referenced – Green et al. 1999; Coles et al. 2003; DiDonato et al. 2006; Birkeland 2007b; Lovell and McLardy 2008.

**American Sāmoa status** – Present. **Evidence** – Single specimen report (identified by D Fenner). **Distribution** – American Sāmoa, Tutuila. **Nearest confirmed ecoregion** – Sāmoa, Tuvalu, and Tonga. **Vulnerability** – LC.

***Pocilloporameandrina* Dana, 1846 (206964)** ^CoTW^

*Pocilloporameandrina* cf. Dana, 1846 (206964). ^CoTW^ Reported – Birkeland et al. 2003.

*Pocilloporameandrina* Dana, 1846 (206964). ^CoTW^ Reported – USACE 1980; Birkeland et al. 1987, 2003; Hunter et al. 1993; Maragos et al. 1994, 1995; Mundy 1996; Green and Hunter 1998; Birkeland and Belliveau 2000; Craig et al. 2001; Fisk and Birkeland 2002; Work and Rameyer 2002; Coles et al. 2003; DiDonato et al. 2006; Birkeland 2007a; Fenner et al. 2008; Kenyon et al. 2010; CRED 2011; Corals NPAS 2016; DMWR 2018; Fenner 2018. Referenced – Green et al. 1999; Fisk and Birkeland 2002; Coles et al. 2003; DiDonato et al. 2006; Birkeland 2007a, 2007b; Lovell and McLardy 2008.

*Pocilloporameandrioa* Dana, 1846 (206964) [sic]. Reported – Hunter et al. 1993.

**American Sāmoa status** – Present. **Evidence** – Single specimen report (identified by D Fenner). **Distribution** – American Sāmoa, Aunuʻu, Manuʻa Islands, Ofu, Ofu/Olosega, Olosega, Rose Atoll, South Bank, Swains, Taʻū, Tutuila. **Nearest confirmed ecoregion** – Sāmoa, Tuvalu, and Tonga. **Vulnerability** – 𝒞, LC.

***Pocilloporaverrucosa* (Ellis & Solander, 1786) (206954)** ^CoTW^

*Pocilloporadanae* cf. Verrill, 1864 (206963) heterotypic synonym. ^CoTW^ Reported – Montgomery et al. 2019.

*Pocilloporadanae* Verrill, 1864 (206963) heterotypic synonym. ^CoTW^ Reported – USACE 1980; Lamberts 1983; Birkeland et al. 1987, 2003; Green et al. 1997; Birkeland and Belliveau 2000; Craig et al. 2001; Fisk and Birkeland 2002; Coles et al. 2003; Birkeland 2007a; Corals NPAS 2016. Referenced – Green et al. 1999; Coles et al. 2003; DiDonato et al. 2006; Birkeland 2007b; Lovell and McLardy 2008; Kenyon et al. 2011.

*Pocilloporaverrucosa* (Ellis & Solander, 1786) (206954). ^CoTW^ Reported – USACE 1980; Lamberts 1983; Birkeland et al. 1987, 2003; Hunter et al. 1993; Maragos et al. 1994; Mundy 1996; Green et al. 1997; Green and Hunter 1998; Birkeland and Belliveau 2000; Craig et al. 2001; Fisk and Birkeland 2002; Work and Rameyer 2002; Coles et al. 2003; DiDonato et al. 2006; Birkeland 2007a; Fenner et al. 2008; Kenyon et al. 2010; CRED 2011; Corals NPAS 2016; Fenner and Sudek 2016; DMWR 2018; Fenner 2018; Montgomery et al. 2019. Referenced – Green et al. 1999; Fisk and Birkeland 2002; Coles et al. 2003; DiDonato et al. 2006; Birkeland 2007a, 2007b; Lovell and McLardy 2008.

**American Sāmoa status** – Present. **Evidence** – Multiple specimen reports. **Distribution** – American Sāmoa, Aunuʻu, Manuʻa Islands, Ofu, Ofu/Olosega, Olosega, Rose Atoll, Swains, Taʻū, Tutuila. **Nearest confirmed ecoregion** – Sāmoa, Tuvalu, and Tonga. **Vulnerability** – LC. **Mesophotic record** – 48 m depth (Montgomery et al. 2019).

***Pocilloporawoodjonesi* Vaughan, 1918 (289252)** ^CoTW^

*Pocilloporawoodjonesi* Vaughan, 1918 (289252). ^CoTW^ Reported – Lamberts 1983; Fisk and Birkeland 2002; Birkeland 2007a; Fenner et al. 2008; CRED 2011; Corals NPAS 2016; Fenner 2018; QM 2018. Referenced – DiDonato et al. 2006; Birkeland 2007b; Lovell and McLardy 2008.

*Pocilloporawoodjonsi* Vaughan, 1918 (289252) [sic]. Reported – USACE 1980.

**American Sāmoa status** – Present. **Evidence** – Multiple specimen reports. **Distribution** – American Sāmoa, Aunuʻu, Manuʻa Islands, Ofu, Ofu/Olosega, Swains, Taʻū, Tutuila. **Nearest confirmed ecoregion** – Sāmoa, Tuvalu, and Tonga. **Vulnerability** – LC.

######## Genus *Seriatopora* Lamarck, 1816

***Seriatoporahystrix* Dana, 1846 (206973)** ^CoTW^

*Seriatoporaangulata* Klunzinger, 1879 (206971) heterotypic synonym. Reported – BPBM 2018. Referenced – Coles et al. 2003.

*Seriatoporahystrix* Dana, 1846 (206973). ^CoTW^ Reported – USACE 1980; Lamberts 1983; Birkeland et al. 1987; Maragos et al. 1994. Referenced – Coles et al. 2003; Birkeland 2007b; Lovell and McLardy 2008.

**American Sāmoa status** – Present. **Evidence** – Multiple specimen reports. **Distribution** – American Sāmoa, Aunuʻu, Tutuila. **Nearest confirmed ecoregion** – Sāmoa, Tuvalu, and Tonga. **Vulnerability** – LC.

######## Genus *Stylophora* Schweigger, 1820

***Stylophorapistillata* Esper, 1797 (206982)** ^CoTW^

*Stylophoramordax* (Dana, 1846) (206981) heterotypic synonym. Reported – USACE 1980; Lamberts 1983; Birkeland et al. 1987; Itano and Buckley 1988; Fisk and Birkeland 2002; Coles et al. 2003; Birkeland 2007a; NMNH 2018. Referenced – Green et al. 1999; Coles et al. 2003; Birkeland 2007a, 2007b.

*Stylophoramordax* cf. (Dana, 1846) (206981) heterotypic synonym. Reported – USACE 1980.

*Stylophorapistiliata* Esper, 1797 (206982) [sic]. Reported – Hunter et al. 1993.

*Stylophorapistillata* Esper, 1797 (206982). ^CoTW^ Reported – Hunter et al. 1993; Maragos et al. 1994; Mundy 1996; Bare et al. 2010; CRED 2011; Birkeland et al. 2013; Corals NPAS 2016; DMWR 2018; Fenner 2018; NMNH 2018; Montgomery et al. 2019. Referenced – Fisk and Birkeland 2002; DiDonato et al. 2006; Lovell and McLardy 2008.

*Stylophorapistllata* Esper, 1797 (206982) [sic]. Referenced – Coles et al. 2003.

**American Sāmoa status** – Present. **Evidence** – Multiple specimen reports. **Distribution** – American Sāmoa, Aunuʻu, Manuʻa Islands, Ofu, Ofu/Olosega, Olosega, Rose Atoll, Swains, Tutuila. **Nearest confirmed ecoregion** – Sāmoa, Tuvalu, and Tonga. **Vulnerability** – NT. **Mesophotic record** – 50 m depth (Montgomery et al. 2019). **Notes** – Eight species or forms of *Stylophora* are found in the Indian Ocean and Red Sea. *Stylophora* spp. decrease in number from west to east and only the especially thick-branched and non-red-tinged form of *Stylophorapistillata* Esper, 1797 has made it to American Sāmoa.

####### Family Poritidae Gray, 1840

######## Genus *Goniopora* de Blainville, 1830

***Gonioporacolumna* Dana, 1846 (207221)** ^CoTW^

*Gonioporacollumna* Dana, 1846 (207221) [sic]. Reported – DMWR 2018.

*Gonioporacolumna* Dana, 1846 (207221). ^CoTW^ Reported – Birkeland et al. 1987; Coles et al. 2003; Fenner 2018. Referenced – Birkeland 2007b; Lovell and McLardy 2008.

**American Sāmoa status** – Present. **Evidence** – Single specimen report (identified by D Fenner). **Distribution** – American Sāmoa, Tutuila. **Nearest confirmed ecoregion** – Fiji. **Geographical range extension** – East. **Vulnerability** – NT.

***Gonioporafruticosa* Saville-Kent, 1891 (288272)** ^CoTW^

*Gonioporafruiticosa* Saville-Kent, 1891 (288272) [sic]. Reported – Corals NPAS 2016; DMWR 2018.

*Gonioporafruticosa* Saville-Kent, 1891 (288272). ^CoTW^ Reported – DiDonato et al. 2006; Fenner 2018. Referenced – Lovell and McLardy 2008.

**American Sāmoa status** – Present. **Evidence** – Single specimen report (identified by D Fenner). **Distribution** – American Sāmoa, Aunuʻu, Tutuila. **Nearest confirmed ecoregion** – Sāmoa, Tuvalu, and Tonga. **Vulnerability** – LC.

***Gonioporapandoraensis* Veron & Pichon, 1982 (288275)** ^CoTW^

*Gonioporapandaorensis* Veron & Pichon, 1982 (288275) [sic]. Reported – DMWR 2018.

**American Sāmoa status** – Present. **Evidence** – Single specimen report (identified by D Fenner). **Distribution** – Tutuila. **Nearest confirmed ecoregion** – Vanuatu. **Geographical range extension** – East. **Vulnerability** – LC.

***Gonioporasomaliensis* Vaughan, 1907 (207212)** ^CoTW^

*Goniopo*r*a somaliensis* cf. Vaughan, 1907 (207212). ^CoTW^ Reported – USACE 1980; Lamberts 1983; Montgomery et al. 2019.

*Gonioporasomaliensis* Vaughan, 1907 (207212). ^CoTW^ Reported – Birkeland et al. 1987, 2003; Mundy 1996; Coles et al. 2003; Kenyon et al. 2010; Corals NPAS 2016; DMWR 2018. Referenced – Green et al. 1999; Fisk and Birkeland 2002; Coles et al. 2003; DiDonato et al. 2006; Birkeland 2007a, 2007b; Lovell and McLardy 2008.

**American Sāmoa status** – Present. **Evidence** – Multiple specimen reports. **Distribution** – American Sāmoa, Manuʻa Islands, Rose Atoll, Taʻū, Tutuila. **Nearest confirmed ecoregion** – Sāmoa, Tuvalu, and Tonga. **Vulnerability** – LC. **Mesophotic record** – 47 m depth (Montgomery et al. 2019).

######## Genus *Porites* Link, 1807

***Poritesannae* Crossland, 1952 (288886)** ^CoTW^

*Poritesannae* Crossland, 1952 (288886). ^CoTW^ Reported – Birkeland et al. 1987, 2003; Hunter et al. 1993; Maragos et al. 1994; Mundy 1996; Green and Hunter 1998; Craig et al. 2001; Coles et al. 2003; DiDonato et al. 2006; Birkeland 2007a; Fenner et al. 2008; Forsman et al. 2009; Corals NPAS 2016; DMWR 2018; Fenner 2018. Referenced – Green et al. 1999; Fisk and Birkeland 2002; Coles et al. 2003; DiDonato et al. 2006; Birkeland 2007a, 2007b; Lovell and McLardy 2008.

**American Sāmoa status** – Present. **Evidence** – Single specimen report (identified by D Fenner). **Distribution** – American Sāmoa, Aunuʻu, Manuʻa Islands, Ofu, Ofu/Olosega, Olosega, Rose Atoll, Taʻū, Tutuila. **Nearest confirmed ecoregion** – Sāmoa, Tuvalu, and Tonga. **Vulnerability** – NT.

***Poritesarnaudi* Reyes-Bonilla & Carricart-Ganivet, 2000 (288888)** ^CoTW^

*Poritesarnaudi* cf. Reyes-Bonilla & Carricart-Ganivet, 2000 (288888). ^CoTW^ Reported – DMWR 2018.

*Poritesarnaudi* Reyes-Bonilla & Carricart-Ganivet, 2000 (288888). ^CoTW^ Reported – Fenner 2018; Montgomery et al. 2019.

**American Sāmoa status** – Present. **Evidence** – Multiple specimen reports. **Distribution** – Aunuʻu, Olosega, Tutuila. **Nearest confirmed ecoregion** – Clipperton Atoll, east Pacific. **Geographical range extension** – Southwest, Veron et al. (2019) strongly predicted this species in the Society Islands, French Polynesia indicating it exists further west than Clipperton Atoll. **Vulnerability** – LC. **Mesophotic record** – 49 m depth (Montgomery et al. 2019).

***Poritescylindrica* Dana, 1846 (207229)** ^CoTW^

*Poritesandrewsi* Vaughan, 1918 (207252) heterotypic synonym. Reported – Mayor 1924a, 1924b; Hoffmeister 1925; USACE 1980; Lamberts 1983; BPBM 2018; NMNH 2018. Referenced – Dahl and Lamberts 1977; Dahl 1981; Green et al. 1997.

*Poritescapricornis* Rehberg, 1892 (760262) heterotypic synonym. Reported – NMNH 2018.

*Poritescylindrica* ? Dana, 1846 (207229). ^CoTW^ Reported – DMWR 2018.

*Poritescylindrica* Dana, 1846 (207229). ^CoTW^ Reported – Birkeland et al. 1987, 2003, 2013; Hunter et al. 1993; Maragos et al. 1994, 1995; Mundy 1996; Green and Hunter 1998; Mundy and Green 1999; Birkeland and Belliveau 2000; Craig et al. 2001; Fisk and Birkeland 2002; Coles et al. 2003; Cornish and DiDonato 2004; DiDonato et al. 2006; Birkeland 2007a; Fenner et al. 2008; Forsman et al. 2009; CRED 2011; Corals NPAS 2016; Fenner and Sudek 2016; DMWR 2018; Fenner 2018. Referenced – Green et al. 1999; Fisk and Birkeland 2002; Coles et al. 2003; DiDonato et al. 2006; Birkeland 2007a, 2007b; Lovell and McLardy 2008.

*Portiescylindrica* Dana, 1846 (207229) [sic]. Reported – Maragos et al. 1995.

**American Sāmoa status** – Present. **Evidence** – Multiple specimen reports. **Distribution** – American Sāmoa, Aunuʻu, Manuʻa Islands, Ofu, Ofu/Olosega, Olosega, Rose Atoll, Taʻū, Tutuila. **Nearest confirmed ecoregion** – Sāmoa, Tuvalu, and Tonga. **Vulnerability** – NT.

***Poritesevermanni* Vaughan, 1907 (288900)** ^CoTW^

*Poritesevermanni* Vaughan, 1907 (288900). ^CoTW^ Reported – Fenner 2018.

**American Sāmoa status** – Present. **Evidence** – Single photographic record. **Distribution** – Aunuʻu, Ofu/Olosega, Rose Atoll, Swains, Taʻū, Tutuila. **Nearest confirmed ecoregion** – New Caledonia and Kiribati west, Gilbert Islands. **Geographical range extension** – East and Southeast. **Vulnerability** – DD.

***Poriteshorizontalata* Hoffmeister, 1925 (207237)** ^CoTW^

*Poriteshorizontalata* cf. Hoffmeister, 1925 (207237). ^CoTW^ Reported – Fisk and Birkeland 2002.

*Poriteshorizontalata* Hoffmeister, 1925 (207237). ^CoTW^ Reported – Hoffmeister 1925; USACE 1980; Birkeland et al. 1987; Coles et al. 2003; Corals NPAS 2016; Fenner 2018; NMNH 2018. Referenced – Coles et al. 2003; DiDonato et al. 2006; Birkeland 2007b; Lovell and McLardy 2008; Kenyon et al. 2011.

*Poriteshorizontallata* Hoffmeister, 1925 (207237) [sic]. Reported – DMWR 2018.

*Synaraeahorizontalata* Hoffmeister, 1925 (207237) [sic]. Reported – USACE 1980; Lamberts 1983.

**American Sāmoa status** – Present. **Evidence** – Type specimen location. **Distribution** – American Sāmoa, Manuʻa Islands, Tutuila. **Nearest confirmed ecoregion** – Sāmoa, Tuvalu, and Tonga. **Vulnerability** – VU. **Mesophotic record** – 33, 30 m depth (Hoffmeister 1925; Lamberts 1983).

***Poriteslatistellata* Quelch, 1886 (869070)** ^CoTW^

*Poriteslatistella* Quelch, 1886 (288906) wrong species spelling. Reported – USACE 1980; Lamberts 1983. Referenced – Lovell and McLardy 2008.

**American Sāmoa status** – Present. **Evidence** – Single specimen report (identified by A Lamberts). **Distribution** – American Sāmoa, Tutuila. **Nearest confirmed ecoregion** – Vanuatu and Society Islands, French Polynesia. **Geographical range extension** – Between two disjunct ecoregions.

***Poriteslichen* Dana, 1846 (207228)** ^CoTW^

*Poriteslichen /randalli* ? Dana, 1846 (207228). Reported – DMWR 2018.

*Poriteslichen* Dana, 1846 (207228). ^CoTW^ Reported – Lamberts 1983; Birkeland et al. 1987, 2003; Hunter et al. 1993; Maragos et al. 1994; Mundy 1996; Craig et al. 2001; Fisk and Birkeland 2002; Coles et al. 2003; Birkeland 2007a; Fenner et al. 2008; Forsman et al. 2009; Kenyon et al. 2010; CRED 2011; Corals NPAS 2016; DMWR 2018; Fenner 2018. Referenced – Green et al. 1999; Fisk and Birkeland 2002; Coles et al. 2003; DiDonato et al. 2006; Birkeland 2007a, 2007b; Lovell and McLardy 2008.

*Portieslichen* Dana, 1846 (207228) [sic]. Reported – USACE 1980.

**American Sāmoa status** – Present. **Evidence** – Multiple specimen reports. **Distribution** – American Sāmoa, Aunuʻu, Manuʻa Islands, Ofu, Ofu/Olosega, Olosega, Rose Atoll, Swains, Taʻū, Tutuila. **Nearest confirmed ecoregion** – Sāmoa, Tuvalu, and Tonga. **Vulnerability** – LC. **Mesophotic record** – 30 m depth (Lamberts 1983).

***Poriteslobata* Dana, 1846 (207225)** ^CoTW^

*Poriteslobata /lutea* Dana, 1846 (207225). Reported – Hunter et al. 1993.

*Poriteslobata forma nodulosa* Dana, 1846 (207225). Reported – Hoffmeister 1925. Referenced – Green et al. 1997.

*Poriteslobatanodulosa* Dana, 1846 (207225). Reported – NMNH 2018.

*Poriteslobata* aff. Dana, 1846 (207225). ^CoTW^ Reported – USACE 1980.

*Poriteslobata* cf. Dana, 1846 (207225). ^CoTW^ Reported – USACE 1980; Maragos et al. 1994; Fisk and Birkeland 2002; Cornish and DiDonato 2004; DMWR 2018.

*Poriteslobata* Dana, 1846 (207225). ^CoTW^ Reported – Hoffmeister 1925; USACE 1980; Lamberts 1983; Birkeland et al. 1987, 2003; Maragos et al. 1994, 1995; Green and Hunter 1998; Coles et al. 2003; DiDonato et al. 2006; Birkeland 2007a; Forsman et al. 2009;Kenyon et al. 2010; CRED 2011; Corals NPAS 2016; DMWR 2018. Referenced – Green et al. 1999; Coles et al. 2003; DiDonato et al. 2006; Birkeland 2007a, 2007b; Lovell and McLardy 2008.

**American Sāmoa status** – Present. **Evidence** – Multiple specimen reports. **Distribution** – American Sāmoa, Aunuʻu, Manuʻa Islands, Ofu, Ofu/Olosega, Olosega, Rose Atoll, Swains, Taʻū, Tutuila. **Nearest confirmed ecoregion** – Sāmoa, Tuvalu, and Tonga. **Vulnerability** – NT. **Notes** – The photo of this species reported in Corals NPAS (2016) appears to be uncertain.

***Poriteslutea* Milne Edwards & Haime, 1851 (207246)** ^CoTW^

*Poritesarenosa* (Esper, 1797) (207241) heterotypic synonym. Reported – USACE 1980.

*Poriteslutea /evermanni* Milne Edwards & Haime, 1851 (207246). Reported – DMWR 2018.

*Poritesluteahaddoni* Vaughan, 1918 (994051). Reported – NMNH 2018.

Poritesluteavar.haddoni aff. Milne Edwards & Haime, 1851 (207246). Reported – Mayor 1924b.

Poritesluteavar.haddoni Milne Edwards & Haime, 1851 (207246). Reported – Mayor 1924b; Hoffmeister 1925. Referenced – Green et al. 1997.

*Poriteslutea* ? Milne Edwards & Haime, 1851 (207246). ^CoTW^ Reported – DMWR 2018.

*Poriteslutea* aff. Milne Edwards & Haime, 1851 (207246). ^CoTW^ Reported – Mayor 1924b; USACE 1980.

*Poriteslutea* cf. Milne Edwards & Haime, 1851 (207246). ^CoTW^ Reported – USACE 1980; Fisk and Birkeland 2002.

*Poriteslutea* Milne Edwards & Haime, 1851 (207246). ^CoTW^ Reported – Mayor 1924a, 1924b; Hoffmeister 1925; USACE 1980; Lamberts 1983; Birkeland et al. 1987, 2003; Itano and Buckley 1988; Maragos et al. 1994, 1995; Mundy 1996; Green et al. 1997; Green and Hunter 1998; Fisk and Birkeland 2002; Coles et al. 2003; DiDonato et al. 2006; Birkeland 2007a; Forsman et al. 2009; Kenyon et al. 2010; CRED 2011; Corals NPAS 2016; BPBM 2018; DMWR 2018; NMNH 2018. Referenced – Dahl and Lamberts 1977; Dahl 1981; Green et al. 1997, 1999; Fisk and Birkeland 2002; Coles et al. 2003; DiDonato et al. 2006; Birkeland 2007a, 2007b; Lovell and McLardy 2008.

Poritesvar.haddoni Vaughan, 1918 (760256) heterotypic synonym. Reported – USACE 1980.

*Portieslutea* Milne Edwards & Haime, 1851 (207246) [sic]. Reported – Maragos et al. 1995.

**American Sāmoa status** – Present. **Evidence** – Multiple specimen reports. **Distribution** – American Sāmoa, Aunuʻu, Manuʻa Islands, Ofu, Ofu/Olosega, Olosega, Rose Atoll, Swains, Taʻū, Tutuila. **Nearest confirmed ecoregion** – Sāmoa, Tuvalu, and Tonga. **Vulnerability** – LC. **Mesophotic record** – 30 m depth (Lamberts 1983). **Notes** – The septal pattern of *P.evermanni* and *P.lutea* are essentially identical (Fenner 2005). Colonies of *P.lutea* collected and analyzed molecularly from American Sāmoa fell into three distinct clades that included colonies identified as *P.evermanni* (Forsman et al. 2009). The type specimen of *P.lutea* has not been examined, and E Turak (pers. comm.) indicates that the label on the presumed type in the Paris Natural History Museum may have been moved from a different skeleton. Thus, there are significant taxonomic challenges for this species and more work needed to sort the differences.

***Poritesmonticulosa* Dana, 1846 (367816)** ^CoTW^

*Poritesmonticulosa* Dana, 1846 (367816). ^CoTW^ Reported – Birkeland et al. 1987; CRED 2011; Corals NPAS 2016; DMWR 2018; Fenner 2018. Referenced – Coles et al. 2003; DiDonato et al. 2006; Birkeland 2007b; Lovell and McLardy 2008.

**American Sāmoa status** – Present. **Evidence** – Single specimen report (identified by D Fenner). **Distribution** – American Sāmoa, Swains, Tutuila. **Nearest confirmed ecoregion** – Sāmoa, Tuvalu, and Tonga. **Vulnerability** – LC. **Notes** – This species is very similar to *Poritesrus* (Forskål, 1775). The type specimen of *P.monticulosa* in the Yale Peabody Museum is a short, thick, rounded column. It can be reliably separated from *P.rus* in many locations in the Pacific, including American Sāmoa and Hawaii (Fenner 2005). However, there are no reliable microscopic features that distinguish it from *P.rus* and the species concept sensu Veron (2000) and Veron et al. (2019).

***Poritesmurrayensis* Vaughan, 1918 (207232)** ^CoTW^

*Poritesmurraensis* Vaughan, 1918 (207232) [sic]. Referenced – Green et al. 1997.

*Poritesmurrayensis* ? Vaughan, 1918 (207232). ^CoTW^ Reported – DMWR 2018.

*Poritesmurrayensis* Vaughan, 1918 (207232). ^CoTW^ Reported – Hoffmeister 1925; USACE 1980; Lamberts 1983; Birkeland et al. 1987, 2003; Hunter et al. 1993; Maragos et al. 1994; Green and Hunter 1998; Coles et al. 2003; CRED 2011; Corals NPAS 2016; NMNH 2018. Referenced – Green et al. 1999; Coles et al. 2003; DiDonato et al. 2006; Birkeland 2007a, 2007b; Lovell and McLardy 2008.

**American Sāmoa status** – Present. **Evidence** – Multiple specimen reports. **Distribution** – American Sāmoa, Aunuʻu, Ofu, Ofu/Olosega, Olosega, Tutuila. **Nearest confirmed ecoregion** – Sāmoa, Tuvalu, and Tonga. **Vulnerability** – NT.

***Poritesrandalli* Forsman & Birkeland, 2009 (758221)** ^CoTW^

*Poritesrandalli* Forsman & Birkeland, 2009 (758221). ^CoTW^ Reported – Forsman and Birkeland 2009; BPBM 2018; DMWR 2018; Fenner 2018; NMNH 2018; Paulay and Brown 2019.

**American Sāmoa status** – Present. **Evidence** – Type specimen location. **Distribution** – American Sāmoa, Aunuʻu, Ofu, Ofu/Olosega, Olosega, Taʻū, Tutuila. **Nearest confirmed ecoregion** – Sāmoa, Tuvalu, and Tonga.

***Poritesrus* (Forskål, 1775) (207231)** ^CoTW^

*Poritesconvexa* (Verrill, 1864) (207230) heterotypic synonym. Reported – Birkeland et al. 1987, 2003; Coles et al. 2003; BPBM 2018. Referenced – Green et al. 1999; Coles et al. 2003.

*Poritesfaustinoi* Hoffmeister, 1925 (207239) heterotypic synonym. Reported – Hoffmeister 1925; NMNH 2018.

*Poritesrus* (Forskål, 1775) (207231). ^CoTW^ Reported – Birkeland et al. 1987, 2003; Itano and Buckley 1988; Hunter et al. 1993; Maragos et al. 1994, 1995; Mundy 1996; Green et al. 1997; Green and Hunter 1998; Birkeland and Belliveau 2000; Fisk and Birkeland 2002; Coles et al. 2003; Cornish and DiDonato 2004; DiDonato et al. 2006; Birkeland 2007a; Fenner et al. 2008; Kenyon et al. 2010; CRED 2011; Corals NPAS 2016; Fenner and Sudek 2016; DMWR 2018; Fenner 2018; NMNH 2018; Montgomery et al. 2019. Referenced – Green et al. 1999; Fisk and Birkeland 2002; Coles et al. 2003; DiDonato et al. 2006; Birkeland 2007a, 2007b; Lovell and McLardy 2008.

*Poritesundulata* (Verrill, 1864) (207243) heterotypic synonym. Reported – Hoffmeister 1925; USACE 1980; NMNH 2018. Referenced – Green et al. 1997.

*Synaraeafaustino* Hoffmeister, 1925 (207239) [sic] heterotypic synonym. Reported – Lamberts 1983.

*Synaraeaundulata* Klunzinger, 1879 (760291) heterotypic synonym. Reported – USACE 1980; Lamberts 1983.

**American Sāmoa status** – Present. **Evidence** – Multiple specimen reports. **Distribution** – American Sāmoa, Aunuʻu, Manuʻa Islands, Ofu, Ofu/Olosega, Olosega, Rose Atoll, Swains, Taʻū, Tutuila. **Nearest confirmed ecoregion** – Sāmoa, Tuvalu, and Tonga. **Vulnerability** – LC. **Mesophotic record** – 47 m depth (Montgomery et al. 2019). **Notes** – Veron et al. (2019) report *P.faustinoi* as a synonym of *P.horizontalata*.

***Poritesstephensoni* Crossland, 1952 (288915)** ^CoTW^

*Poritesstephensoni* Crossland, 1952 (288915). ^CoTW^ Reported – Birkeland et al. 1987; Fenner 2018. Referenced – Birkeland 2007b; Lovell and McLardy 2008.

**American Sāmoa status** – Present. **Evidence** – Single photographic record. **Distribution** – American Sāmoa, Aunuʻu, Ofu/Olosega, Tutuila. **Nearest confirmed ecoregion** – Vanuatu. **Geographical range extension** – East. **Vulnerability** – NT. **Notes** – This is a small, distinctive, massive species that only lives on reef flats and is not difficult to identify.

######## Genus *Stylaraea* Milne Edwards & Haime, 1851

***Stylaraeapunctata* (Linnaeus, 1758) (212178)** ^CoTW^

*Stylaraeapunctata* (Linnaeus, 1758) (212178). ^CoTW^ Reported – Green et al. 1997; Birkeland and Belliveau 2000; Birkeland et al. 2013; BPBM 2018; DMWR 2018; Fenner 2018. Referenced – Birkeland 2007b; Lovell and McLardy 2008.

*Stylareapunctata* (Linnaeus, 1758) (212178) [sic]. Reported – Coles et al. 2003; Corals NPAS 2016. Referenced – Coles et al. 2003; DiDonato et al. 2006.

**American Sāmoa status** – Present. **Evidence** – Multiple specimen reports. **Distribution** – American Sāmoa, Ofu, Ofu/Olosega, Tutuila. **Nearest confirmed ecoregion** – Sāmoa, Tuvalu, and Tonga. **Vulnerability** – DD.

####### Family Psammocoridae Chevalier & Beauvais, 1987

######## Genus *Psammocora* Dana, 1846

***Psammocoracontigua* (Esper, 1794) (207267)** ^CoTW^

*Psammacoracontigua* (Esper, 1794) (207267) [sic]. Reported – USACE 1980.

*Psammocoracontiguatutuilensis* (Esper, 1794) (207267). Reported – NMNH 2018.

Psammocoracontiguavar.maldivensis (Esper, 1794) (207267). Reported – Mayor 1924b; Hoffmeister 1925.

Psammocoracontiguavar.tutuilensis Hoffmeister, 1925 (869367). Reported – Hoffmeister 1925. Referenced – Green et al. 1997.

*Psammocoracontigua* (Esper, 1794) (207267). ^CoTW^ Reported – Mayor 1924b; Hoffmeister 1925; USACE 1980; Lamberts 1983; Birkeland et al. 1987, 2003; Maragos et al. 1994, 1995; Mundy 1996; Green et al. 1997; Birkeland and Belliveau 2000; Craig et al. 2001; Fisk and Birkeland 2002; Coles et al. 2003; Birkeland 2007a; Fenner et al. 2008; Kenyon et al. 2010; Corals NPAS 2016; BPBM 2018; DMWR 2018; Fenner 2018; NMNH 2018. Referenced – Dahl and Lamberts 1977; Dahl 1981; Green et al. 1997, 1999; Fisk and Birkeland 2002; Coles et al. 2003; DiDonato et al. 2006; Birkeland 2007a, 2007b; Lovell and McLardy 2008.

*Psammocoraobtusangula* cf. (Lamarck, 1816) (287783) heterotypic synonym. ^CoTW^ Reported – Coles et al. 2003.

**American Sāmoa status** – Present. **Evidence** – Multiple specimen reports. **Distribution** – American Sāmoa, Manuʻa Islands, Ofu, Ofu/Olosega, Olosega, Rose Atoll, Taʻū, Tutuila. **Nearest confirmed ecoregion** – Sāmoa, Tuvalu, and Tonga. **Vulnerability** – NT.

***Psammocoradigitata* Milne Edwards & Haime, 1851 (207260)** ^CoTW^

*Psammocoradigitata* Milne Edwards & Haime, 1851 (207260). ^CoTW^ Reported – Coles et al. 2003; DiDonato et al. 2006; Corals NPAS 2016; Fenner 2018. Referenced – Lovell and McLardy 2008.

**American Sāmoa status** – Present. **Evidence** – Multiple photographic records. **Distribution** – American Sāmoa, Aunuʻu, Ofu, Ofu/Olosega, Taʻū, Tutuila. **Nearest confirmed ecoregion** – Vanuatu. **Geographical range extension** – East although Veron et al. (2019) strongly predicted the presence of this species in the Sāmoa, Tuvalu, and Tonga ecoregion. **Vulnerability** – NT. **Notes** – This species forms massive colonies, which can be very large, and not columnar. Historically, reports under *P.digitata* should be considered *Psammocorahaimiana* Milne Edwards & Haime, 1851, and vice versa (Benzoni et al. 2010; Veron et al. 2019). However, the name *P.haimiana* has never been reported in American Sāmoa. See note on *P.haimiana*.

***Psammocoranierstraszi* Van der Horst, 1921 (207261)** ^CoTW^

*Psammacoranietstraszi* Van der Horst, 1921 (207261) [sic]. Reported – USACE 1980.

*Psammocoraneirstraszi* Van der Horst, 1921 (207261) [sic]. Reported – Birkeland et al. 1987. Referenced – Green et al. 1999; Birkeland 2007b.

*Psammocoranierstraszi* aff. Van der Horst, 1921 (207261). ^CoTW^ Reported – Coles et al. 2003.

*Psammocoranierstraszi* Van der Horst, 1921 (207261). ^CoTW^ Reported – Lamberts 1983; Maragos et al. 1994; Green and Hunter 1998; Fisk and Birkeland 2002; Birkeland et al. 2003; Coles et al. 2003; Fenner et al. 2008; Kenyon et al. 2010; CRED 2011; DMWR 2018; Fenner 2018; Montgomery et al. 2019. Referenced – Coles et al. 2003; Lovell and McLardy 2008.

*Psammocoranierstrazi* Van der Horst, 1921 (207261) [sic]. Reported – Fisk and Birkeland 2002.

**American Sāmoa status** – Present. **Evidence** – Multiple specimen reports. **Distribution** – American Sāmoa, Aunuʻu, Manuʻa Islands, Ofu/Olosega, Olosega, Rose Atoll, Swains, Taʻū, Tutuila. **Nearest confirmed ecoregion** – Sāmoa, Tuvalu, and Tonga. **Vulnerability** – LC. **Mesophotic record** – 47 m depth (Montgomery et al. 2019).

***Psammocoraprofundacella* Gardiner, 1898 (207271)** ^CoTW^

*Psammacorasuperficialis* Gardiner, 1898 (207270) [sic] heterotypic synonym. Reported – USACE 1980; CRED 2011.

*Psammocoraprofundacella* Gardiner, 1898 (207271). ^CoTW^ Reported – Maragos et al. 1994, 1995; Mundy 1996; Fisk and Birkeland 2002; Fenner et al. 2008; Benzoni et al. 2010; Corals NPAS 2016; DMWR 2018; Fenner 2018; NMNH 2018; Montgomery et al. 2019. Referenced – Fisk and Birkeland 2002; DiDonato et al. 2006; Lovell and McLardy 2008.

*Psammocoraprofundicella* Gardiner, 1898 (207271) [sic]. Reported – Green and Hunter 1998; Coles et al. 2003; Birkeland 2007a. Referenced – Coles et al. 2003; Birkeland 2007a, 2007b.

*Psammocorasamoaensis* Hoffmeister, 1925 (718645) [sic] heterotypic synonym. Reported – NMNH 2018.

*Psammocorasamoensis* Hoffmeister, 1925 (718645) heterotypic synonym. Reported – Hoffmeister 1925; Lamberts 1983; Birkeland et al. 1987, 2003; Green et al. 1997. Referenced – Green et al. 1999; Coles et al. 2003.

*Psammocorasuperficiales* Gardiner, 1898 (207270) [sic] heterotypic synonym. Reported – Birkeland et al. 1987.

*Psammocorasuperficialis /nierstraszi* Gardiner, 1898 (207270) heterotypic synonym. Reported – DMWR 2018.

*Psammocorasuperficialis* Gardiner, 1898 (207270) heterotypic synonym. Reported – Hoffmeister 1925; Lamberts 1983; Maragos et al. 1994; Mundy 1996; Green and Hunter 1998; Fisk and Birkeland 2002; Birkeland et al. 2003; Benzoni et al. 2010; Corals NPAS 2016; DMWR 2018. Referenced – Green et al. 1999; Fisk and Birkeland 2002; Coles et al. 2003; DiDonato et al. 2006; Birkeland 2007a, 2007b; Lovell and McLardy 2008.

**American Sāmoa status** – Present. **Evidence** – Multiple specimen reports. **Distribution** – American Sāmoa, Aunuʻu, Manuʻa Islands, Ofu, Ofu/Olosega, Olosega, Rose Atoll, Taʻū, Tutuila. **Nearest confirmed ecoregion** – Sāmoa, Tuvalu, and Tonga. **Vulnerability** – LC. **Mesophotic record** – 48 m depth (Montgomery et al. 2019). **Notes** – See note for *P.haimiana* for more details.

###### Scleractinian genera *incertae sedis*

####### Genus *Leptastrea* Milne Edwards & Haime, 1849

***Leptastreabewickensis* Veron, Pichon & Best, 1977 (287822)** ^CoTW^

*Leptastreabewickensis* ? Veron, Pichon & Best, 1977 (287822). ^CoTW^ Reported – Coles et al. 2003.

*Leptastreabewickensis* Veron, Pichon & Best, 1977 (287822). ^CoTW^ Reported – Fisk and Birkeland 2002; Birkeland 2007a; Kenyon et al. 2010; CRED 2011; Corals NPAS 2016; DMWR 2018; Fenner 2018. Referenced – DiDonato et al. 2006; Birkeland 2007a, 2007b; Lovell and McLardy 2008.

**American Sāmoa status** – Present. **Evidence** – Single specimen report (identified by D Fenner). **Distribution** – American Sāmoa, Manuʻa Islands, Ofu, Ofu/Olosega, Olosega, Rose Atoll, Taʻū, Tutuila. **Nearest confirmed ecoregion** – Sāmoa, Tuvalu, and Tonga. **Vulnerability** – NT. **Notes** – The photo of this species reported in Corals NPAS (2016) appears to be incorrect and may be another *Leptastrea* sp.

***Leptastreabottae* (Milne Edwards & Haime, 1849) (207476)** ^CoTW^

*Leptastreabottae* (Milne Edwards & Haime, 1849) (207476). ^CoTW^ Reported – Lamberts 1983.

*Leptastreaimmersa* Klunzinger, 1879 (207473) heterotypic synonym. Reported – Birkeland et al. 1987.

**American Sāmoa status** – Present. **Evidence** – Single specimen report (identified by A Lamberts). **Distribution** – Tutuila. **Nearest confirmed ecoregion** – New Caledonia and Society Islands, French Polynesia. **Geographical range extension** – Between two disjunct ecoregions. **Vulnerability** – NT. **Notes** – Lamberts (1983) reported this species as rare, and Birkeland et al. (1987) reported it from three sites as the synonym *L.immersa*.

***Leptastreapruinosa* Crossland, 1952 (207472)** ^CoTW^

*Leptastreapruinosa* ? Crossland, 1952 (207472). ^CoTW^ Reported – Coles et al. 2003.

*Leptastreapruinosa* Crossland, 1952 (207472). ^CoTW^ Reported – Kenyon et al. 2010; CRED 2011; Fenner 2018; NMNH 2018; Montgomery et al. 2019.

**American Sāmoa status** – Present. **Evidence** – Single specimen report (identifier unknown). **Distribution** – Aunuʻu, Ofu/Olosega, Rose Atoll, Taʻū, Tutuila. **Nearest confirmed ecoregion** – Sāmoa, Tuvalu, and Tonga. **Vulnerability** – LC. **Mesophotic record** – 32 m depth (Montgomery et al. 2019). **Notes** – The color/tissue seen on living colonies makes identification from photographs easier than from skeleton. The species is documented by both specimens and photographs.

***Leptastreapurpurea* (Dana, 1846) (207470)** ^CoTW^

*Leptastreapurpurea* (Dana, 1846) (207470). ^CoTW^ Reported – Mayor 1924a, 1924b; Hoffmeister 1925; USACE 1980; Lamberts 1983; Birkeland et al. 1987, 2003, 2013; Maragos et al. 1994, 1995; Mundy 1996; Green et al. 1997; Green and Hunter 1998; Birkeland and Belliveau 2000; Craig et al. 2001; Fisk and Birkeland 2002; Coles et al. 2003; DiDonato et al. 2006; Birkeland 2007a; Fenner et al. 2008; Kenyon et al. 2010; CRED 2011; Corals NPAS 2016; BPBM 2018; Fenner 2018; NMNH 2018. Referenced – Dahl and Lamberts 1977; Dahl 1981; Green et al. 1997, 1999; Fisk and Birkeland 2002; Coles et al. 2003; DiDonato et al. 2006; Birkeland 2007a, 2007b; Lovell and McLardy 2008.

*Letpastreapurpurea* (Dana, 1846) (207470) [sic]. Reported – DMWR 2018.

*Letpastreapurpurea* cf. (Dana, 1846) (207470) [sic]. Reported – DMWR 2018.

**American Sāmoa status** – Present. **Evidence** – Multiple specimen reports. **Distribution** – American Sāmoa, Aunuʻu, Manuʻa Islands, Ofu, Ofu/Olosega, Olosega, Rose Atoll, Swains, Taʻū, Tutuila. **Nearest confirmed ecoregion** – Sāmoa, Tuvalu, and Tonga. **Vulnerability** – LC. **Mesophotic record** – 52 m depth (Montgomery et al. 2019).

***Leptastreatransversa* Klunzinger, 1879 (207474)** ^CoTW^

*Leptastreatransversa* Klunzinger, 1879 (207474). ^CoTW^ Reported – Birkeland et al. 1987, 2003; Itano and Buckley 1988; Maragos et al. 1994; Mundy 1996; Fisk and Birkeland 2002; Coles et al. 2003; DiDonato et al. 2006; Birkeland 2007a; Fenner et al. 2008; Kenyon et al. 2010; Corals NPAS 2016; DMWR 2018; Fenner 2018; Montgomery et al. 2019. Referenced – Green et al. 1999; Fisk and Birkeland 2002; Coles et al. 2003; DiDonato et al. 2006; Birkeland 2007a, 2007b; Lovell and McLardy 2008.

**American Sāmoa status** – Present. **Evidence** – Single specimen report (identified by D Fenner and J Wolstenholme). **Distribution** – American Sāmoa, Aunuʻu, Manuʻa Islands, Ofu, Ofu/Olosega, Olosega, Rose Atoll, Taʻū, Tutuila. **Nearest confirmed ecoregion** – Sāmoa, Tuvalu, and Tonga. **Vulnerability** – LC. **Mesophotic record** – 34 m depth (Montgomery et al. 2019).

####### Genus *Pachyseris* Milne Edwards & Haime, 1849

***Pachyserisgemmae* Nemenzo, 1955 (288721)** ^CoTW^

*Pachyserisgemmae* Nemenzo, 1955 (288721). ^CoTW^ Reported – DMWR 2018; Fenner 2018.

**American Sāmoa status** – Present. **Evidence** – Single specimen report (identified by D Fenner). **Distribution** – Tutuila. **Nearest confirmed ecoregion** – Sāmoa, Tuvalu, and Tonga. **Vulnerability** – NT.

***Pachyserisrugosa* (Lamarck, 1801) (207292)** ^CoTW^

*Pachyseriscarinata* Brüggemann, 1879 (766851) heterotypic synonym. Reported – Hoffmeister 1925; USACE 1980; Lamberts 1983.

*Pachyserisrugosa* (Lamarck, 1801) (207292). ^CoTW^ Reported – Birkeland et al. 1987; Maragos et al. 1994; Green and Hunter 1998; DMWR 2018; Fenner 2018; NMNH 2018. Referenced – Coles et al. 2003; Birkeland 2007b; Lovell and McLardy 2008; Kenyon et al. 2011.

**American Sāmoa status** – Present. **Evidence** – Multiple specimen reports. **Distribution** – American Sāmoa, Aunuʻu, Tutuila. **Nearest confirmed ecoregion** – Sāmoa, Tuvalu, and Tonga. **Vulnerability** – VU.

***Pachyserisspeciosa* (Dana, 1846) (207293)** ^CoTW^

*Pachyserislevicollis* (Dana, 1846) (207294) heterotypic synonym. Reported – Hoffmeister 1925; USACE 1980; Lamberts 1983.

*Pachyserisspeciosa* (Dana, 1846) (207293). ^CoTW^ Reported – Hoffmeister 1925; USACE 1980; Lamberts 1983; Birkeland et al. 1987; Maragos et al. 1994; Mundy 1996; Fisk and Birkeland 2002; Coles et al. 2003; Corals NPAS 2016; BPBM 2018; DMWR 2018; Fenner 2018; NMNH 2018; Montgomery et al. 2019. Referenced – Fisk and Birkeland 2002; Coles et al. 2003; DiDonato et al. 2006; Birkeland 2007b; Lovell and McLardy 2008.

**American Sāmoa status** – Present. **Evidence** – Multiple specimen reports. **Distribution** – American Sāmoa, Aunuʻu, Manuʻa Islands, Tutuila. **Nearest confirmed ecoregion** – Sāmoa, Tuvalu, and Tonga. **Vulnerability** – LC. **Mesophotic record** – 33, 30, 52 m depth (Hoffmeister 1925; Lamberts 1983; Montgomery et al. 2019).

####### Genus *Plerogyra* Milne Edwards & Haime, 1848

***Plerogyrasimplex* Rehberg, 1892 (287848)** ^CoTW^

*Plerogyrasimplex* Rehberg, 1892 (287848). ^CoTW^ Reported – USACE 1980; Lamberts 1983; DMWR 2018; Fenner 2018.

**American Sāmoa status** – Present. **Evidence** – Multiple specimen reports. **Distribution** – American Sāmoa, Tutuila. **Nearest confirmed ecoregion** – Fiji. **Geographical range extension** – East. **Vulnerability** – NT.

***Plerogyrasinuosa* (Dana, 1846) (207498)** ^CoTW^

*Plerogyrasinuosa* (Dana, 1846) (207498). ^CoTW^ Reported – Maragos et al. 1994; Corals NPAS 2016; Fenner 2018. Referenced – DiDonato et al. 2006; Birkeland 2007a.

**American Sāmoa status** – Present. **Evidence** – Multiple photographic records. **Distribution** – Ofu, Ofu/Olosega, Olosega, Tutuila. **Nearest confirmed ecoregion** – Sāmoa, Tuvalu, and Tonga. **Vulnerability** – NT.

####### Subclass Octocorallia Haeckel, 1866

######## Order Helioporacea Bock, 1938

######### Family Helioporidae Moseley, 1876

########## Genus *Heliopora* de Blainville, 1830

***Helioporacoerulea* (Pallas, 1766) (210725)**

*Helioporacoerulca* (Pallas, 1766) (210725) [sic]. Reported – Hunter et al. 1993.

*Helioporacoerulea* (Pallas, 1766) (210725). Reported – USACE 1980; Lamberts 1983; Itano and Buckley 1988; Hunter et al. 1993; Maragos et al. 1994, 1995; Craig et al. 2001; Coles et al. 2003; DiDonato et al. 2006; Birkeland 2007a; CRED 2011; Corals NPAS 2016; Fenner 2018. Referenced – DiDonato et al. 2006; Birkeland 2007a; Lovell and McLardy 2008; Kenyon et al. 2011.

*Heliporacoerulea* (Pallas, 1766) (210725) [sic]. Reported – DMWR 2018.

**American Sāmoa status** – Present. **Evidence** – Single specimen report (identified by A Lamberts). **Distribution** – American Sāmoa, Ofu, Ofu/Olosega, Olosega, South Bank, Swains, Taʻū, Tutuila. **Nearest confirmed ecoregion** – Not available. **Vulnerability** – VU.

####### Class Hydrozoa Owen, 1843

######## Order Anthoathecata Cornelius, 1992

######### Family Milleporidae Fleming, 1828

########## Genus *Millepora* Linnaeus, 1758

***Milleporadichotoma* Forskål, 1775 (210733)**

*Milleporadichotoma* cf. Forskål, 1775 (210733). Reported – Birkeland et al. 1987.

*Milleporadichotoma* Forskål, 1775 (210733). Reported – Birkeland et al. 1987, 2003; Itano and Buckley 1988; Hunter et al. 1993; Craig et al. 2001; Coles et al. 2003; DiDonato et al. 2006; Birkeland 2007a; Fenner et al. 2008; Corals NPAS 2016; DMWR 2018; Fenner 2018. Referenced – Green et al. 1999; Coles et al. 2003; DiDonato et al. 2006; Birkeland 2007a, 2007b; Lovell and McLardy 2008.

**American Sāmoa status** – Present. **Evidence** – Single specimen report (identified by D Fenner). **Distribution** – American Sāmoa, Aunuʻu, Ofu, Ofu/Olosega, Tutuila. **Nearest confirmed ecoregion** – Not available. **Vulnerability** – LC.

***Milleporaexaesa* Forsskål, 1775 (210728)**

*Milleporaexaesa* Forsskål, 1775 (210728). Reported – Green et al. 1997; Fisk and Birkeland 2002; DiDonato et al. 2006; Birkeland 2007a; Fenner et al. 2008; Corals NPAS 2016; Fenner 2018. Referenced – Fisk and Birkeland 2002; Coles et al. 2003; DiDonato et al. 2006; Birkeland 2007a, 2007b; Lovell and McLardy 2008.

*Milleporaexesa* Forsskål, 1775 (210728) [sic]. Reported – DMWR 2018.

*Milleporatuberosa* Boschma, 1966 (210732) heterotypic synonym. Reported – Birkeland et al. 1987, 2003; Birkeland and Belliveau 2000; Coles et al. 2003; DMWR 2018; Fenner 2018. Referenced – Green et al. 1999; Coles et al. 2003; Birkeland 2007b; Lovell and McLardy 2008; Kenyon et al. 2011.

**American Sāmoa status** – Present. **Evidence** – Single specimen report (identified by D Fenner). **Distribution** – American Sāmoa, Aunuʻu, Manuʻa Islands, Ofu, Ofu/Olosega, Olosega, Taʻū, Tutuila. **Nearest confirmed ecoregion** – Not available. **Vulnerability** – LC. **Notes** – *Milleporaexaesa* is yellow-brown with occasional light green or pink, encrusts rubble, can have larger bumps, and found in lagoons. The synonym *Milleporatuberosa* Boschma, 1966 is purple encrusting sheets on hard substrate that can grow quite large and is found on slopes. Skeletons can easily be confused, but live colonies are distinguishable. For taxonomic details, see Randall and Cheng (1984). We believe that the taxonomy of this group needs to be revised and that the synonym *M.tuberosa* Boschma, 1966 may deserve to be resurrected for specimens outside the Red Sea (Arrigoni et al. 2018b). The photographed specimen of this species reported in Corals NPAS (2016) appears to have an incorrect identification and should be *Milleporaplatyphylla* Hemprich & Ehrenberg, 1834.

***Milleporaintricata* Milne Edwards, 1860 (210727)**

*Milleporaintricata* Milne Edwards, 1860 (210727). Referenced – Lovell and McLardy 2008.

*Milleporamurrayi* Quelch, 1884 (292201) possible heterotypic synonym. Reported – DiDonato et al. 2006; Corals NPAS 2016; DMWR 2018; Fenner 2018. Referenced – Lovell and McLardy 2008.

**American Sāmoa status** – Present. **Evidence** – Single specimen report (identified by D Fenner). **Distribution** – American Sāmoa, Ofu, Tutuila. **Nearest confirmed ecoregion** – Not available. **Vulnerability** – LC. **Notes** – Colonies of *M.intricata* have less obvious ogives while the synonym *Milleporamurrayi* Quelch, 1884 has very obvious ogives, which are downward curving branches with upward growing branches on the upper edge. Fenner has seen *M.intricata* in the Philippines which could be mistaken for *M.murrayi*. Razak and Hoeksema (2003) were correct that the colonies reported from Indonesia were *M.intricata*, but it is possible that *M.murrayi* is also present and valid. We believe this group needs to be revisited and the synonym *M.murrayi* may deserve to be resurrected. For more taxonomic details, see Randall and Cheng (1984).

***Milleporaplatyphylla* Hemprich & Ehrenberg, 1834 (210730)**

*Milleporaplatyphylla* cf. Hemprich & Ehrenberg, 1834 (210730). Reported – DMWR 2018.

*Milleporaplatyphylla* Hemprich & Ehrenberg, 1834 (210730). Reported – USACE 1980; Lamberts 1983; Birkeland et al. 1987, 2003; Itano and Buckley 1988; Hunter et al. 1993; Maragos et al. 1994, 1995; Birkeland and Belliveau 2000; Craig et al. 2001; Coles et al. 2003; DiDonato et al. 2006; Birkeland 2007a; Fenner et al. 2008; Kenyon et al. 2010; Corals NPAS 2016; Fenner and Sudek 2016; BPBM 2018; Fenner 2018. Referenced – Green et al. 1999; Coles et al. 2003; Birkeland 2007a, 2007b; Lovell and McLardy 2008.

*Milleporaplatyphyllia* cf. Hemprich & Ehrenberg, 1834 (210730) [sic]. Reported – Fisk and Birkeland 2002.

**American Sāmoa status** – Present. **Evidence** – Multiple specimen reports. **Distribution** – American Sāmoa, Aunuʻu, Ofu, Ofu/Olosega, Rose Atoll, Sāmoa Islands, Taʻū, Tutuila. **Nearest confirmed ecoregion** – Not available. **Vulnerability** – LC.

### Possibly present

#### Class Anthozoa Ehrenberg, 1834

##### Subclass Hexacorallia Haeckel, 1896

###### Order Scleractinia Bourne, 1900

####### Family Acroporidae Verrill, 1902

######## Genus *Acropora* Oken, 1815

***Acroporabushyensis* Veron & Wallace, 1984 (206999)** ^CoTW CCW^

*Acroporabushiensis* Veron & Wallace, 1984 (206999) [sic]. Reported – Mundy 1996. Referenced – Birkeland 2007b.

*Acroporabushyensis* Veron & Wallace, 1984 (206999). ^CoTW CCW^ Referenced – Lovell and McLardy 2008.

**American Sāmoa status** – Possibly present. **Evidence** – Single report. **Distribution** – American Sāmoa, Tutuila. **Nearest confirmed ecoregion** – New Caledonia. **Geographical range extension** – East. **Vulnerability** – LC.

***Acroporaechinata* (Dana, 1846) (207069)** ^CoTW CCW^

*Acroporaechinata* (Dana, 1846) (207069). ^CoTW CCW^ Reported – Maragos et al. 1994; BPBM 2018. Referenced – Hoffmeister 1925; Coles et al. 2003; Birkeland 2007a, 2007b.

**American Sāmoa status** – Possibly present. **Evidence** – Single specimen report (identifier unknown). **Distribution** – Ofu/Olosega, Sāmoa Islands, Tutuila. **Nearest confirmed ecoregion** – Sāmoa, Tuvalu, and Tonga. **Vulnerability** – VU. **Notes** – This species is reported in American Sāmoa based on four specimens labeled as *A.echinata* in the BPBM collection. However, the specimens listed at BPBM do not show documentation about the person that provided the identification. The referenced reports are based on Maragos et al. (1994) except for Hoffmeister (1925), which references this species presence in the Sāmoa Islands from Brook (with uncertain location). Based on the limited observations and the lack of a confirmed identification in the BPBM collection, we consider this species as possibly present in American Sāmoa.

***Acroporaelseyi* (Brook, 1892) (207113)** ^CoTW CCW^

*Acroporaelseyi* (Brook, 1892) (207113). ^CoTW CCW^ Reported – Craig et al. 2001; Corals NPAS 2016. Referenced – DiDonato et al. 2006; Lovell and McLardy 2008.

**American Sāmoa status** – Possibly present. **Evidence** – Single photographic record. **Distribution** – American Sāmoa, Ofu. **Nearest confirmed ecoregion** – Sāmoa, Tuvalu, and Tonga. **Vulnerability** – LC. **Notes** – This species was reported by Craig et al. (2001) and photo-documented in Corals NPAS 2016 (2016). Craig et al. (2001) reported this coral outside a belt transect area during surveys in the Ofu pools indicating this species has a low abundance. DiDonato et al. (2006) references to Craig et al. (2001), but the referenced document of this species in Lovell and McLardy (2008) could not be located. This species is similar to *A.carduus*. Based on these limited observations and its close similarity to another species, we determined this species as possibly present in American Sāmoa.

***Acroporaglauca* (Brook, 1893) (207017)** ^CoTW CCW^

*Acroporaglauca* (Brook, 1893) (207017). ^CoTW CCW^ Reported – Fisk and Birkeland 2002; DiDonato et al. 2006; Corals NPAS 2016. Referenced – Birkeland 2007b; Lovell and McLardy 2008.

**American Sāmoa status** – Possibly present. **Evidence** – Single photographic record. **Distribution** – American Sāmoa, Ofu, Taʻū, Tutuila. **Nearest confirmed ecoregion** – Sāmoa, Tuvalu, and Tonga. **Vulnerability** – NT. **Notes** – The identity of the photographed specimen in Corals NPAS (2016) appears to be incorrect and should be *A.clathrata*.

***Acroporahorrida* (Dana, 1846) (207006)** ^CoTW CCW^

*Acroporahorrida* (Dana, 1846) (207006). ^CoTW CCW^ Reported – USACE 1980; Lamberts 1983; Craig et al. 2001; Birkeland 2007a; Corals NPAS 2016. Referenced – DiDonato et al. 2006; Lovell and McLardy 2008; Kenyon et al. 2011.

*Acroporahorrida* ? (Dana, 1846) (207006). ^CoTW CCW^ Reported – Coles et al. 2003.

**American Sāmoa status** – Possibly present. **Evidence** – Single specimen report (identified by A Lamberts). **Distribution** – American Sāmoa, Ofu, Tutuila. **Nearest confirmed ecoregion** – Sāmoa, Tuvalu, and Tonga. **Vulnerability** – VU.

***Acroporakirstyae* Veron & Wallace, 1984 (288215)** ^CoTW CCW^

*Acroporakirstyae* Veron & Wallace, 1984 (288215). ^CoTW CCW^ Reported – Birkeland 2007a; Kenyon et al. 2010.

**American Sāmoa status** – Possibly present. **Evidence** – Multiple reports. **Distribution** – Ofu, Rose Atoll. **Nearest confirmed ecoregion** – New Caledonia. **Geographical range extension** – East although Veron et al. (2019) strongly predicted the presence of this species in the Sāmoa, Tuvalu, and Tonga ecoregion. **Vulnerability** – VU. **Notes** – This species is fairly distinctive.

***Acroporaloripes* (Brook, 1892) (207074)** ^CoTW CCW^

*Acroporaloripes* (Brook, 1892) (207074). ^CoTW CCW^ Reported – Birkeland et al. 2003; Fenner et al. 2008; Kenyon et al. 2010. Referenced – Green et al. 1999; Coles et al. 2003; Lovell and McLardy 2008.

*Acroporarosaria* (Dana, 1846) (207029) possible heterotypic synonym. ^CoTW^ Reported – Fisk and Birkeland 2002. Referenced – Hoffmeister 1925.

**American Sāmoa status** – Possibly present. **Evidence** – Multiple reports. **Distribution** – American Sāmoa, Rose Atoll, Sāmoa Islands, Taʻū, Tutuila. **Nearest confirmed ecoregion** – Sāmoa, Tuvalu, and Tonga. **Vulnerability** – NT.

***Acroporamicrophthalma* (Verrill, 1870) (207046)** ^CoTW CCW^

*Acroporamicrophthalma* (Verrill, 1870) (207046). ^CoTW CCW^ Reported – Craig et al. 2001; Fisk and Birkeland 2002; Corals NPAS 2016. Referenced – DiDonato et al. 2006; Lovell and McLardy 2008.

*Acroporamicropthalma* (Verrill, 1870) (207046) [sic]. Reported – Birkeland 2007a. Referenced – Birkeland 2007b.

**American Sāmoa status** – Possibly present. **Evidence** – Single photographic record. **Distribution** – American Sāmoa, Ofu, Tutuila. **Nearest confirmed ecoregion** – Sāmoa, Tuvalu, and Tonga. **Vulnerability** – LC. **Notes** – Veron et al. (2019) report this species is readily confused with other *Acropora* species with a staghorn-like form. Wallace (1999) reports this species is difficult to distinguish from *Acroporamuricata* (Linnaeus, 1758). Despite the limited number of observations of this species and the difficulty in its identification, we believe that the photographic record from Corals NPAS (2016) is plausible evidence of its presence.

***Acroporasarmentosa* (Brook, 1892) (288244)** ^CoTW CCW^

*Acroporasarmentosa* (Brook, 1892) (288244). ^CoTW CCW^ Reported – Fisk and Birkeland 2002.

**American Sāmoa status** – Possibly present. **Evidence** – Multiple reports. **Distribution** – Taʻū. **Nearest confirmed ecoregion** – Sāmoa, Tuvalu, and Tonga. **Vulnerability** – LC. **Notes** – *Acroporasarmentosa* is very distinctive and reported to be common by Veron et al. (2019). Given it has been reported by so few papers, we label this species as possibly present. *Acroporaverweyi* Veron & Wallace, 1984 is somewhat similar and present.

***Acroporaspicifera* (Dana, 1846) (207087)** ^CoTW CCW^

*Acroporaspicefera* (Dana, 1846) (207087) [sic]. Reported – USACE 1980.

*Acroporaspicifera* (Dana, 1846) (207087). ^CoTW CCW^ Reported – Lamberts 1983; Maragos et al. 1994; Corals NPAS 2016. Referenced – DiDonato et al. 2006; Birkeland 2007a, 2007b; Lovell and McLardy 2008.

**American Sāmoa status** – Possibly present. **Evidence** – Single specimen report (identified by A Lamberts). **Distribution** – American Sāmoa, Ofu, Ofu/Olosega, Tutuila. **Nearest confirmed ecoregion** – Sāmoa, Tuvalu, and Tonga. **Vulnerability** – VU. **Notes** – *Acroporaspicifera* is a difficult species to identify in situ, but a sample has been identified by A Lamberts. Veron et al. (2019) report this species to be uncommon outside Australia.

***Acroporasquarrosa* (Ehrenberg, 1834) (207053)** ^CoTW CCW^

*Acroporasquarrosa* (Ehrenberg, 1834) (207053). ^CoTW CCW^ Reported – USACE 1980; Lamberts 1983; Birkeland et al. 1987; Maragos et al. 1994; Kenyon et al. 2010; Corals NPAS 2016. Referenced – Coles et al. 2003; DiDonato et al. 2006; Birkeland 2007b.

*Acroporasquarrosa* cf. (Ehrenberg, 1834) (207053). ^CoTW CCW^ Reported – Birkeland et al. 1987.

**American Sāmoa status** – Possibly present. **Evidence** – Single specimen report (identified by A Lamberts). **Distribution** – American Sāmoa, Aunuʻu, Rose Atoll, Tutuila. **Nearest confirmed ecoregion** – Madagascar north. **Geographical range extension** – East although Veron et al. (2019) stated this species distribution was uncertain due to taxonomic uncertainties, significant geographical range extension. **Vulnerability** – LC. **Notes** – *Acroporasquarrosa* is a Red Sea and western Indian Ocean species, and both Wallace (1999) and Veron (2000) report that it is endemic to the Red Sea. Based on a sample identified by A Lamberts and in situ reports (Birkeland et al. 1987; Maragos et al. 1994), we believe that this species is possibly present. However, caution may be warranted on this species due to the later work not available to Lamberts (1983), Birkeland et al. 1987, and Maragos et al. (1994).

***Acroporastriata* (Verrill, 1866) (207081)** ^CoTW CCW^

*Acroporastriata* (Verrill, 1866) (207081). ^CoTW CCW^ Reported – Fisk and Birkeland 2002; Birkeland 2007a. Referenced – Kenyon et al. 2011.

**American Sāmoa status** – Possibly present. **Evidence** – Multiple reports. **Distribution** – Manuʻa Islands, Ofu. **Nearest confirmed ecoregion** – Sāmoa, Tuvalu, and Tonga. **Vulnerability** – VU.

***Acroporasubglabra* (Brook, 1891) (288250)** ^CoTW CCW^

*Acroporasubglabra* (Brook, 1891) (288250). ^CoTW CCW^ Reported – Birkeland et al. 2003.

**American Sāmoa status** – Possibly present. **Evidence** – Single report. **Distribution** – Tutuila. **Nearest confirmed ecoregion** – Sāmoa, Tuvalu, and Tonga. **Vulnerability** – LC. **Notes** – This species is similar to *A.echinata* and *A.carduus*.

***Acroporavalenciennesi* (Milne Edwards, 1860) (206995)** ^CoTW CCW^

*Acroporasplendida* Nemenzo, 1967 (740875) heterotypic synonym. Reported – USACE 1980; Lamberts 1983.

*Acroporavalenciennesi* (Milne Edwards, 1860) (206995). ^CoTW CCW^ Referenced – Lovell and McLardy 2008.

**American Sāmoa status** – Possibly present. **Evidence** – Single specimen report (identified by A Lamberts). **Distribution** – American Sāmoa, Tutuila. **Nearest confirmed ecoregion** – Sāmoa, Tuvalu, and Tonga. **Vulnerability** – LC.

***Acroporavaughani* Wells, 1954 (288262)** ^CoTW CCW^

*Acroporavaughani* Wells, 1954 (288262). ^CoTW CCW^ Reported – Maragos et al. 1994; DiDonato et al. 2006; Birkeland 2007a; Corals NPAS 2016. Referenced – Birkeland 2007a, 2007b; Lovell and McLardy 2008; Kenyon et al. 2011.

**American Sāmoa status** – Possibly present. **Evidence** – Single photographic record. **Distribution** – American Sāmoa, Aunuʻu, Ofu, Ofu/Olosega, Tutuila. **Nearest confirmed ecoregion** – Sāmoa, Tuvalu, and Tonga. **Vulnerability** – VU.

***Acroporayongei* Veron & Wallace, 1984 (207032)** ^CoTW CCW^

*Acroporayongei /pulchra* Veron & Wallace, 1984 (207032). Reported – DMWR 2018.

*Acroporayongei* ? Veron & Wallace, 1984 (207032). ^CoTW CCW^ Reported – Coles et al. 2003.

*Acroporayongei* Veron & Wallace, 1984 (207032). ^CoTW CCW^ Reported – Birkeland et al. 1987, 2003; Mundy 1996; DiDonato et al. 2006; Birkeland 2007a; Corals NPAS 2016. Referenced – Green et al. 1999; Coles et al. 2003; Birkeland 2007b; Lovell and McLardy 2008.

**American Sāmoa status** – Possibly present. **Evidence** – Single photographic record. **Distribution** – American Sāmoa, Ofu, Tutuila. **Nearest confirmed ecoregion** – Sāmoa, Tuvalu, and Tonga. **Vulnerability** – LC.

######## Genus *Alveopora* Blainville, 1830

***Alveoporaexcelsa* Verrill, 1864 (289255)** ^CoTW^

*Alveoporaexcelsa* Verrill, 1864 (289255). ^CoTW^ Reported – Montgomery et al. 2019.

**American Sāmoa status** – Possibly present. **Evidence** – Single photographic record. **Distribution** – Tutuila. **Nearest confirmed ecoregion** – Raja Ampat, Papua. **Geographical range extension** – East. **Vulnerability** – EN. **Mesophotic record** – 52 m depth (Montgomery et al. 2019). **Notes** – *Alveopora* species can be difficult to identify from photographs or even skeletons.

***Alveoporaspongiosa* Dana, 1846 (207198)** ^CoTW^

*Alveoporaspongiosa* ? Dana, 1846 (207198). ^CoTW^ Reported – DMWR 2018.

*Alveoporaspongiosa* cf. Dana, 1846 (207198). ^CoTW^ Reported – Mundy 1996. Referenced – Fisk and Birkeland 2002.

*Alveoporaspongiosa* Dana, 1846 (207198). ^CoTW^ Reported – Fisk and Birkeland 2002; Birkeland 2007a; Montgomery et al. 2019. Referenced – Birkeland 2007b.

**American Sāmoa status** – Possibly present. **Evidence** – Single photographic record. **Distribution** – American Sāmoa, Manuʻa Islands, Ofu, Tutuila. **Nearest confirmed ecoregion** – Sāmoa, Tuvalu, and Tonga. **Vulnerability** – NT. **Mesophotic record** – 46 m depth (Montgomery et al. 2019).

***Alveoporasuperficialis* Pillai & Scheer, 1976 (207199)**

*Alveoporasuperficiales* Pillai & Scheer, 1976 (207199) [sic]. Reported – Birkeland et al. 1987. Referenced – Green et al. 1999; Birkeland 2007b.

*Alveoporasuperficialis* Pillai & Scheer, 1976 (207199). Reported – Birkeland et al. 1987; Coles et al. 2003. Referenced – Coles et al. 2003.

**American Sāmoa status** – Possibly present. **Evidence** – Multiple reports. **Distribution** – Tutuila. **Nearest confirmed ecoregion** – Not available. **Notes** – Veron et al. (2019) consider this species a synonym of *A.spongiosa*.

######## Genus *Astreopora* Blainville, 1830

***Astreoporaexplanata* Veron, 1985 (287944)**

*Astreopora* e*xplanata* Veron, 1985 (287944). Reported – Maragos et al. 1994. Referenced – Birkeland 2007b.

**American Sāmoa status** – Possibly present. **Evidence** – Single report. **Distribution** – Tutuila. **Nearest confirmed ecoregion** – Not available. **Notes** – This species is considered a synonym of *A.expansa* by Veron et al. (2019).

***Astreoporaincrustans* Bernard, 1896 (207131)** ^CoTW^

*Astreoporaincrustans* Bernard, 1896 (207131). ^CoTW^ Reported – NMNH 2018.

**American Sāmoa status** – Possibly present. **Evidence** – Single specimen report (Identifier unknown). **Distribution** – Tutuila. **Nearest confirmed ecoregion** – Solomon Islands and Bougainville. **Geographical range extension** – East. **Vulnerability** – VU.

***Astreoporaocellata* Bernard, 1896 (207123)** ^CoTW^

*Astreoporaocellata* Bernard, 1896 (207123). ^CoTW^ Reported – Maragos et al. 1994; Fisk and Birkeland 2002. Referenced – Birkeland 2007a, 2007b.

**American Sāmoa status** – Possibly present. **Evidence** – Multiple reports. **Distribution** – Manuʻa Islands, Taʻū, Tutuila. **Nearest confirmed ecoregion** – New Caledonia. **Geographical range extension** – East. **Vulnerability** – LC. **Notes** – This species is subtly different from *A.myriophthalma*.

######## Genus *Isopora* Studer, 1879

***Isoporacuneata* (Dana, 1846) (730687)** ^CoTW CCW^

*Acroporacuneata* (Dana, 1846) (206997) homotypic synonym. Reported – Hunter et al. 1993; Maragos et al. 1994; Corals NPAS 2016. Referenced – DiDonato et al. 2006; Birkeland 2007a, 2007b; Lovell and McLardy 2008.

*Isoporacuneata* (Dana, 1846) (730687). ^CoTW CCW^ Referenced – Kenyon et al. 2011.

**American Sāmoa status** – Possibly present. **Evidence** – Multiple reports. **Distribution** – American Sāmoa, Aunuʻu, Ofu, Ofu/Olosega, Tutuila. **Nearest confirmed ecoregion** – Sāmoa, Tuvalu, and Tonga. **Vulnerability** – VU. **Notes** – See note of *I.crateriformis*.

######## Genus *Montipora* Blainville, 1830

***Montiporaangulata* (Lamarck, 1816) (287691)** ^CoTW^

*Montiporaangulata* (Lamarck, 1816) (287691). ^CoTW^ Reported – Kenyon et al. 2010. Referenced – Kenyon et al. 2011.

**American Sāmoa status** – Possibly present. **Evidence** – Single report. **Distribution** – Rose Atoll. **Nearest confirmed ecoregion** – Solomon Islands and Bougainville. **Geographical range extension** – East. **Vulnerability** – VU. **Notes** – This species was only reported by Kenyon et al. (2010) from Rose Atoll, and is reported to be very rare (Veron et al. 2019).

***Montiporacalcarea* Bernard, 1897 (287695)**

*Montiporacalcarea* Bernard, 1897 (287695). Reported – Fisk and Birkeland 2002; Kenyon et al. 2010; Corals NPAS 2016. Referenced – DiDonato et al. 2006; Birkeland 2007a, 2007b; Lovell and McLardy 2008; Kenyon et al. 2011.

**American Sāmoa status** – Possibly present. **Evidence** – Single photographic record. **Distribution** – American Sāmoa, Manuʻa Islands, Rose Atoll, Taʻū, Tutuila. **Nearest confirmed ecoregion** – Not available. **Vulnerability** – VU. **Notes** – Veron et al. (2019) consider this species unresolved and *M.calcarea* sensu Veron (2000) as an undefined separate species.

***Montiporaconicula* Wells, 1954 (1263760)**

*Montiporaconicula* Wells, 1954 (1263760). Reported – Coles et al. 2003; Corals NPAS 2016. Referenced – DiDonato et al. 2006.

**American Sāmoa status** – Possibly present. **Evidence** – Multiple reports. **Distribution** – American Sāmoa, Ofu, Tutuila. **Nearest confirmed ecoregion** – Not available. **Notes** – This species has been reported by two sources, but there is no photo-documentation or collection material available for this species. However, both sources for this species are from well-known coral experts, so we accept this species presence in American Sāmoa. Veron et al. (2019) consider this species unresolved.

***Montiporacorbettensis* Veron & Wallace, 1984 (287701)** ^CoTW^

*Montiporacorbettensis* Veron & Wallace, 1984 (287701). ^CoTW^ Reported – Mundy 1996; Fisk and Birkeland 2002; Birkeland 2007a; Corals NPAS 2016. Referenced – Fisk and Birkeland 2002; DiDonato et al. 2006; Birkeland 2007b; Lovell and McLardy 2008.

**American Sāmoa status** – Possibly present. **Evidence** – Multiple reports. **Distribution** – American Sāmoa, Aunuʻu, Ofu, Tutuila. **Nearest confirmed ecoregion** – Sāmoa, Tuvalu, and Tonga. **Vulnerability** – VU.

***Montiporadanae* Milne Edwards & Haime, 1851 (207152)** ^CoTW^

*Montiporadanae* Milne Edwards & Haime, 1851 (207152). ^CoTW^ Reported – Maragos et al. 1994; Mundy 1996; Green and Hunter 1998; Fisk and Birkeland 2002; Kenyon et al. 2010. Referenced – Fisk and Birkeland 2002; Birkeland 2007a, 2007b; Lovell and McLardy 2008.

**American Sāmoa status** – Possibly present. **Evidence** – Multiple reports. **Distribution** – American Sāmoa, Aunuʻu, Manuʻa Islands, Ofu, Olosega, Rose Atoll, Taʻū, Tutuila. **Nearest confirmed ecoregion** – Sāmoa, Tuvalu, and Tonga. **Vulnerability** – LC.

***Montiporadigitata* (Dana, 1846) (207185)** ^CoTW^

*Montiporadigitata* (Dana, 1846) (207185). ^CoTW^ Reported – Green and Hunter 1998.

**American Sāmoa status** – Possibly present. **Evidence** – Single report. **Distribution** – Tutuila. **Nearest confirmed ecoregion** – Sāmoa, Tuvalu, and Tonga. **Vulnerability** – LC. **Notes** – Hunter and Green (1998) reported this species with an occasional abundance at two sites.

***Montiporaeffusa* (Dana, 1846) (207169)** ^CoTW^

*Montiporaeffusa* (Dana, 1846) (207169). ^CoTW^ Reported – Fisk and Birkeland 2002; Birkeland 2007a; Corals NPAS 2016. Referenced – DiDonato et al. 2006; Birkeland 2007b; Lovell and McLardy 2008.

**American Sāmoa status** – Possibly present. **Evidence** – Multiple reports. **Distribution** – American Sāmoa, Aunuʻu, Ofu, Tutuila. **Nearest confirmed ecoregion** – Vanuatu and Society Islands, French Polynesia. **Geographical range extension** – Between two disjunct ecoregions. **Vulnerability** – NT.

***Montiporafloweri* Wells, 1954 (287707)** ^CoTW^

*Montiporafloweri* Wells, 1954 (287707). ^CoTW^ Reported – Mundy 1996; Fisk and Birkeland 2002; Birkeland et al. 2003; Coles et al. 2003; Birkeland 2007a. Referenced – Green et al. 1999; Fisk and Birkeland 2002; Coles et al. 2003; Birkeland 2007a, 2007b; Lovell and McLardy 2008.

**American Sāmoa status** – Possibly present. **Evidence** – Multiple reports. **Distribution** – American Sāmoa, Aunuʻu, Manuʻa Islands, Ofu, Olosega, Taʻū, Tutuila. **Nearest confirmed ecoregion** – Sāmoa, Tuvalu, and Tonga. **Vulnerability** – LC.

***Montiporahispida* (Dana, 1846) (207164)** ^CoTW^

*Montiporahispida* (Dana, 1846) (207164). ^CoTW^ Reported – Birkeland et al. 1987; Maragos et al. 1994; Green et al. 1997; Birkeland and Belliveau 2000; Fenner et al. 2008; Corals NPAS 2016. Referenced – Coles et al. 2003; DiDonato et al. 2006; Birkeland 2007a, 2007b; Lovell and McLardy 2008.

**American Sāmoa status** – Possibly present. **Evidence** – Multiple reports. **Distribution** – American Sāmoa, Aunuʻu, Ofu, Ofu/Olosega, Tutuila. **Nearest confirmed ecoregion** – Sāmoa, Tuvalu, and Tonga. **Vulnerability** – LC. **Notes** – This species has a distinctive colony morphology of thin plates and columns.

***Montiporahoffmeisteri* Wells, 1954 (287713)** ^CoTW^

*Montiporahoffmeisteri* cf. Wells, 1954 (287713). ^CoTW^ Reported – Coles et al. 2003.

*Montiporahoffmeisteri* Wells, 1954 (287713). ^CoTW^ Reported – USACE 1980; Birkeland et al. 1987, 2003; Maragos et al. 1994; Mundy 1996; Fisk and Birkeland 2002; Coles et al. 2003; Kenyon et al. 2010; Corals NPAS 2016. Referenced – Fisk and Birkeland 2002; Coles et al. 2003; DiDonato et al. 2006; Birkeland 2007a, 2007b; Lovell and McLardy 2008.

**American Sāmoa status** – Possibly present. **Evidence** – Single photographic record. **Distribution** – American Sāmoa, Aunuʻu, Manuʻa Islands, Ofu, Ofu/Olosega, Olosega, Rose Atoll, Taʻū, Tutuila. **Nearest confirmed ecoregion** – Sāmoa, Tuvalu, and Tonga. **Vulnerability** – LC.

***Montiporalobulata* Bernard, 1897 (207141)** ^CoTW^

*Montiporalobulata* Bernard, 1897 (207141). ^CoTW^ Reported – Birkeland et al. 1987; Fisk and Birkeland 2002; Coles et al. 2003; Kenyon et al. 2010. Referenced – Green et al. 1999; Coles et al. 2003; Birkeland 2007a, 2007b; Kenyon et al. 2011.

**American Sāmoa status** – Possibly present. **Evidence** – Multiple reports. **Distribution** – Manuʻa Islands, Rose Atoll, Taʻū, Tutuila. **Nearest confirmed ecoregion** – Sāmoa, Tuvalu, and Tonga. **Vulnerability** – VU.

***Montiporamillepora* Crossland, 1952 (207190)** ^CoTW^

*Montiporamillepora* Crossland, 1952 (207190). ^CoTW^ Reported – Maragos et al. 1994; Mundy 1996; Fisk and Birkeland 2002; Birkeland 2007a; Kenyon et al. 2010; Corals NPAS 2016. Referenced – Fisk and Birkeland 2002; DiDonato et al. 2006; Birkeland 2007a, 2007b; Lovell and McLardy 2008.

**American Sāmoa status** – Possibly present. **Evidence** – Multiple reports. **Distribution** – American Sāmoa, Manuʻa Islands, Ofu, Taʻū, Tutuila. **Nearest confirmed ecoregion** – Sāmoa, Tuvalu, and Tonga. **Vulnerability** – LC.

***Montiporamollis* Bernard, 1897 (207137)** ^CoTW^

*Montiporamollis* Bernard, 1897 (207137). ^CoTW^ Reported – Fisk and Birkeland 2002; Birkeland 2007a; Corals NPAS 2016. Referenced – DiDonato et al. 2006; Birkeland 2007a; Lovell and McLardy 2008.

**American Sāmoa status** – Possibly present. **Evidence** – Multiple reports. **Distribution** – American Sāmoa, Manuʻa Islands, Ofu. **Nearest confirmed ecoregion** – Sāmoa, Tuvalu, and Tonga. **Vulnerability** – LC.

***Montiporamonasteriata* (Forskål, 1775) (207153)** ^CoTW^

*Montiporamonasteriata* (Forskål, 1775) (207153). ^CoTW^ Reported – Maragos et al. 1995; Mundy 1996; Craig et al. 2001; Fisk and Birkeland 2002; Birkeland et al. 2003; Coles et al. 2003; DiDonato et al. 2006; Birkeland 2007a; Corals NPAS 2016. Referenced – Green et al. 1999; Fisk and Birkeland 2002; Coles et al. 2003; DiDonato et al. 2006; Birkeland 2007a, 2007b; Lovell and McLardy 2008.

**American Sāmoa status** – Possibly present. **Evidence** – Single photographic record. **Distribution** – American Sāmoa, Aunuʻu, Manuʻa Islands, Ofu, Olosega, Taʻū, Tutuila. **Nearest confirmed ecoregion** – Sāmoa, Tuvalu, and Tonga. **Vulnerability** – LC.

***Montiporanodosa* (Dana, 1846) (287719)** ^CoTW^

*Montiporanodosa* (Dana, 1846) (287719). ^CoTW^ Reported – Mundy 1996; Fisk and Birkeland 2002; Work and Rameyer 2002; DiDonato et al. 2006; Birkeland 2007a; Fenner et al. 2008; Kenyon et al. 2010; Corals NPAS 2016. Referenced – Fisk and Birkeland 2002; Coles et al. 2003; DiDonato et al. 2006; Birkeland 2007a, 2007b; Lovell and McLardy 2008.

*Montiporanodosa* cf. (Dana, 1846) (287719). ^CoTW^ Reported – DMWR 2018.

**American Sāmoa status** – Possibly present. **Evidence** – Single photographic record. **Distribution** – American Sāmoa, Aunuʻu, Manuʻa Islands, Ofu, Olosega, Rose Atoll, Taʻū, Tutuila. **Nearest confirmed ecoregion** – Sāmoa, Tuvalu, and Tonga. **Vulnerability** – NT. **Notes** – Corals in some published photographs of this species (Veron 2000) appear to be incorrectly identified, showing no papillae between lumps, while this species has papillae between lumps. The photographs appear to be of *Montiporaturgescens* Bernard, 1897. Identifications based on Veron (2000) may be of *Montiporaturgescens* Bernard, 1897 due to this error.

***Montiporapeltiformis* Bernard, 1897 (207180)** ^CoTW^

*Montiporapeltiformis* Bernard, 1897 (207180). ^CoTW^ Reported – Fisk and Birkeland 2002; DiDonato et al. 2006; Kenyon et al. 2010; CRED 2011; Corals NPAS 2016. Referenced – DiDonato et al. 2006; Birkeland 2007b; Lovell and McLardy 2008.

**American Sāmoa status** – Possibly present. **Evidence** – Single photographic record. **Distribution** – American Sāmoa, Manuʻa Islands, Olosega, Rose Atoll, Taʻū, Tutuila. **Nearest confirmed ecoregion** – Sāmoa, Tuvalu, and Tonga. **Vulnerability** – NT.

***Montiporaundata* Bernard, 1897 (207167)** ^CoTW^

*Montiporacolei* Wells, 1954 (759740) heterotypic synonym. Reported – Birkeland et al. 1987. Referenced – Birkeland 2007b.

*Montiporaundata* Bernard, 1897 (207167). ^CoTW^ Reported – Kenyon et al. 2010. Referenced – Lovell and McLardy 2008.

**American Sāmoa status** – Possibly present. **Evidence** – Multiple reports. **Distribution** – American Sāmoa, Rose Atoll, Tutuila. **Nearest confirmed ecoregion** – Sāmoa, Tuvalu, and Tonga. **Vulnerability** – NT.

***Montiporaverrucosa* (Lamarck, 1816) (207146)** ^CoTW^

*Montiporaverrucosa* (Lamarck, 1816) (207146). ^CoTW^ Reported – Maragos et al. 1994; Mundy 1996; Green and Hunter 1998; Craig et al. 2001; Fisk and Birkeland 2002; Birkeland et al. 2003; Coles et al. 2003; DiDonato et al. 2006; Birkeland 2007a; Kenyon et al. 2010; CRED 2011; Corals NPAS 2016. Referenced – Green et al. 1999; Fisk and Birkeland 2002; Coles et al. 2003; DiDonato et al. 2006; Birkeland 2007a, 2007b; Lovell and McLardy 2008.

**American Sāmoa status** – Possibly present. **Evidence** – Multiple photographic records. **Distribution** – American Sāmoa, Manuʻa Islands, Ofu, Ofu/Olosega, Olosega, Rose Atoll, Taʻū, Tutuila. **Nearest confirmed ecoregion** – Sāmoa, Tuvalu, and Tonga. **Vulnerability** – LC. **Notes** – *Montiporacapitata* has commonly been reported as *M.verrucosa*, especially in Hawaii (Fenner 2005).

####### Family Agariciidae Gray, 1847

######## Genus *Coeloseris* Vaughan, 1918

***Coeloserismayeri* Vaughan, 1918 (207613)** ^CoTW^

*Coeloserismayeri* Vaughan, 1918 (207613). ^CoTW^ Reported – Mundy 1996; Coles et al. 2003; Kenyon et al. 2010. Referenced – Fisk and Birkeland 2002; Birkeland 2007a, 2007b; Lovell and McLardy 2008.

**American Sāmoa status** – Possibly present. **Evidence** – Multiple reports. **Distribution** – American Sāmoa, Manuʻa Islands, Ofu, Rose Atoll, Taʻū, Tutuila. **Nearest confirmed ecoregion** – Sāmoa, Tuvalu, and Tonga. **Vulnerability** – LC.

######## Genus *Pavona* Lamarck, 1801

***Pavonacactus* (Forskål, 1775) (207312)** ^CoTW^

*Pavonacactus* (Forskål, 1775) (207312). ^CoTW^ Reported – Craig et al. 2001; Birkeland 2007a; CRED 2011; Corals NPAS 2016. Referenced – DiDonato et al. 2006; Lovell and McLardy 2008; Kenyon et al. 2011.

*Pavonafoliosa* Dana, 1846 (766375) [sic] heterotypic synonym. Reported – Mayor 1924b.

**American Sāmoa status** – Possibly present. **Evidence** – Multiple reports. **Distribution** – American Sāmoa, Ofu, Ofu/Olosega, Olosega, Tutuila. **Nearest confirmed ecoregion** – Sāmoa, Tuvalu, and Tonga. **Vulnerability** – VU. **Notes** – The identification of a coral in a photo of this species reported in Corals NPAS (2016) appears to be incorrect and should be *Leptoserisgardineri* (van der Horst, 1922). Otherwise, this species has only been observed by Craig et al. (2001) as an off transect observation and by Birkeland (2007a).

***Pavonadiffluens* (Lamarck, 1816) (207295)** ^CoTW^

*Pavonadiffluens* (Lamarck, 1816) (207295). ^CoTW^ Reported – Corals NPAS 2016; DMWR 2018; Fenner 2018. Referenced – DiDonato et al. 2006; Birkeland 2007b; Lovell and McLardy 2008; Kenyon et al. 2011.

*Pavonadiffluens* cf. (Lamarck, 1816) (207295). ^CoTW^ Reported – Birkeland et al. 1987; Montgomery et al. 2019. Referenced – Coles et al. 2003.

**American Sāmoa status** – Possibly present. **Evidence** – Single specimen report (identified by D Fenner). **Distribution** – American Sāmoa, Ofu/Olosega, Olosega, Taʻū, Tutuila. **Nearest confirmed ecoregion** – Socotra Archipelago. **Geographical range extension** – Southeast, significant geographical range extension. **Vulnerability** – 𝒯, VU. **Mesophotic record** – 44 m depth (Montgomery et al. 2019). **Notes** – This species is similar to *Pavonaexplanulata* (Lamarck, 1816) and *Pavonagigantea* Verrill, 1869. The type locality of this species is the Red Sea, from where it is well known. Randall (1995, 2003) has also reported this species from Guam and the Marianas. Veron (2014) reports that colonies in the Pacific Ocean are likely belonging to an undescribed species that is similar to *P.diffluens*, but with different corallite sizes. D Fenner (pers. comm.) reports that there appear to be no differences in corallite sizes or features between the specimen in the NOAA 2014). Given the protection status of *P.diffluens* under the ESA, more analysis is warranted on its type and samples from American Sāmoa. Here, we apply the precautionary principle to this species as possibly present in American Sāmoa until Pacific specimens are confirmed to belong to *P.diffluens* or can be described as a new species.

####### Family Dendrophylliidae Gray, 1847

######## Genus *Rhizopsammia* Verrill, 1870

***Rhizopsammiaverrilli* van der Horst, 1922 (210737)**

*Rhizopsammiaverrilli* van der Horst, 1922 (210737). Reported – Fenner 2018.

**American Sāmoa status** – Possibly present. **Evidence** – Single photographic record. **Distribution** – Tutuila. **Nearest confirmed ecoregion** – Not available. **Notes** – This species has been documented by Fenner (2018), but no samples have been identified. An analysis of the skeletal characteristics is warranted before a firm conclusion on species presence can be made. Arrigoni et al. (2014: fig. 10M–O) provide more information on and illustrations of this species.

######## Genus *Turbinaria* Oken, 1815

***Turbinariaradicalis* Bernard, 1896 (289213)** ^CoTW^

*Turbinariaradicalis* Bernard, 1896 (289213). ^CoTW^ Reported – NMNH 2018.

**American Sāmoa status** – Possibly present. **Evidence** – Single specimen report (identifier unknown). **Distribution** – Tutuila. **Nearest confirmed ecoregion** – Bismarck Sea, New Guinea and Great Barrier Reef south. **Geographical range extension** – East. **Vulnerability** – NT. **Notes** – This species is documented from American Sāmoa from a sample in the NMNH, but the expert that made the identification is not listed within the NMNH (2018) database.

####### Family Euphylliidae Alloiteau, 1952

######## Genus *Euphyllia* Dana, 1846

***Euphylliacristata* Chevalier, 1971 (289214)** ^CoTW^

*Euphylliacristata* Chevalier, 1971 (289214). ^CoTW^ Reported – Fisk and Birkeland 2002.

**American Sāmoa status** – Possibly present. **Evidence** – Single report. **Distribution** – Manuʻa Islands, Tutuila. **Nearest confirmed ecoregion** – Fiji. **Geographical range extension** – East. **Vulnerability** – VU.

**Genus *Galaxea* Oken, 1815**

***Galaxeahorrescens* (Dana, 1846) (707460)** ^CoTW^

*Acrheliahorrescens* (Dana, 1846) (289335) homotypic synonym. Reported – USACE 1980; Lamberts 1983.

*Galaxeahorrescens* (Dana, 1846) (707460). ^CoTW^ Referenced – Lovell and McLardy 2008.

**American Sāmoa status** – Possibly present. **Evidence** – Single report. **Distribution** – American Sāmoa, Tutuila. **Nearest confirmed ecoregion** – Sāmoa, Tuvalu, and Tonga. **Vulnerability** – LC. **Notes** – This observation was from dredged material from the construction of the Pago Pago airport (Lamberts 1983). Separately from the report of *A.horrescens*, Lamberts (1983) stated that fossil corals of the genus *Acrhelia* were found in the airport dredged material. The reference to the fossil coral may have concerned *A.horrescens*, presently known as *G.horrescens*.

####### Family Fungiidae Dana, 1846

######## Genus *Cycloseris* Milne Edwards & Haime, 1849

***Cycloserisexplanulata* (van der Horst, 1922) (716292)**

*Psammocoraexplanulata* van der Horst, 1922 (207265) homotypic synonym. ^CoTW^ Reported – Coles et al. 2003; Corals NPAS 2016.

**American Sāmoa status** – Possibly present. **Evidence** – Single report. **Distribution** – American Sāmoa, Tutuila. **Nearest confirmed ecoregion** – New Caledonia and Society Islands, French Polynesia. **Geographical range extension** – Between two disjunct ecoregions. **Notes** – There remains some significant taxonomic disagreement with the placement of *Psammocoraexplanulata* van der Horst, 1922 in the genus *Cycloseris*. Benzoni et al. (2012a) moved this species into *Cycloseris* based on genetic evidence, but Veron et al. (2019) state that this species does not have the genus-level characters of *Cycloseris* and therefore retains this species in *Psammocora*. However, original genus descriptions are often simple and based on the characters of the type species. They may need to be revised based on new information and the addition of other species. A taxonomic revision seems warranted in this circumstance (Benzoni et al. 2012). The identification of this species can be similar to *Cycloseriswellsi* (Veron & Pichon, 1980) (see the note for that species for further information), and Benzoni et al. (2012) report it may be confused with *Cycloserismokai* (Hoeksema, 1989) although field identifications are distinct.

***Cycloseriswellsi* (Veron & Pichon, 1980) (716291)**

*Coscinaraeawellsi* Veron & Pichon, 1980 (207257) homotypic synonym. ^CoTW^ Reported – Maragos et al. 1994. Referenced – Birkeland 2007b.

*Coscinereawellsi* Veron & Pichon, 1980 (207257) [sic] homotypic synonym. Referenced – Coles et al. 2003.

**American Sāmoa status** – Possibly present. **Evidence** – Single report. **Distribution** – Tutuila. **Nearest confirmed ecoregion** – Fiji. **Geographical range extension** – East although Veron et al. (2019) strongly predicted the presence of this species in the Sāmoa, Tuvalu, and Tonga ecoregion. **Notes** – Similar to *C.explanulata*, this species was moved to the genus *Cycloseris* from *Coscinaraea* (Benzoni et al. 2012). See the note under *C.explanulata* for more details.

######## Genus *Lithophyllon* Rehberg, 1892

***Lithophyllonscabra* (Döderlein, 1901) (716611)**

*Fungiascabra* Döderlein, 1901 (288856) homotypic synonym. ^CoTW^ Reported – Maragos et al. 1994. Referenced – Birkeland 2007b.

**American Sāmoa status** – Possibly present. **Evidence** – Single report. **Distribution** – Tutuila. **Nearest confirmed ecoregion** – Vanuatu and Society Islands, French Polynesia. **Geographical range extension** – Between two disjunct ecoregions. **Notes** – This species is similar to *Lithophyllonconcinna* (Verrill, 1864) and considered rare by Veron et al. (2019), although it has been observed as an abundant species at shallow depths on nearshore reefs in Indonesia and also in shallow water in eastern Australia (Hoeksema 2012c, 2012d, 2015).

***Lithophyllonundulatum* Rehberg, 1892 (290309)** ^CoTW^

*Lithophyllonundulatum* Rehberg, 1892 (290309). ^CoTW^ Reported – Fisk and Birkeland 2002.

**American Sāmoa status** – Possibly present. **Evidence** – Single report. **Distribution** – Tutuila. **Nearest confirmed ecoregion** – Sāmoa, Tuvalu, and Tonga. **Vulnerability** – NT. **Notes** – This species is fairly distinctive, but there has only been a single observation (Fisk and Birkeland 2002). This observation included only a single colony within a quantitative study.

####### Family Lobophylliidae Dai & Horng, 2009

######## Genus *Echinophyllia* Klunzinger, 1879

***Echinophylliaechinata* (Saville-Kent, 1871) (287972)** ^CoTW^

*Echinophylliaechinata* (Saville-Kent, 1871) (287972). ^CoTW^ Reported – Coles et al. 2003; CRED 2011. Referenced – Coles et al. 2003.

**American Sāmoa status** – Possibly present. **Evidence** – Single report. **Distribution** – Tutuila. **Nearest confirmed ecoregion** – Sāmoa, Tuvalu, and Tonga. **Vulnerability** – LC. **Notes** – Coles et al. (2003) erroneously referenced Birkeland et al. 1987; Birkeland et al. (1987) did not report this species.

######## Genus *Lobophyllia* de Blainville, 1830

***Lobophylliahataii* Yabe & Sugiyama, 1936 (207390)** ^CoTW^

*Lobophylliahataii* Yabe & Sugiyama, 1936 (207390). ^CoTW^ Reported – Kenyon et al. 2010.

**American Sāmoa status** – Possibly present. **Evidence** – Single report. **Distribution** – Taʻū. **Nearest confirmed ecoregion** – Sāmoa, Tuvalu, and Tonga. **Vulnerability** – LC.

***Lobophylliaradians* (Milne Edwards & Haime, 1849) (888141)**

*Symphylliaradians* Milne Edwards & Haime, 1849 (207401) homotypic synonym. ^CoTW^ Reported – Fenner et al. 2008.

**American Sāmoa status** – Possibly present. **Evidence** – Single report. **Distribution** – Tutuila. **Nearest confirmed ecoregion** – Sāmoa, Tuvalu, and Tonga.

***Lobophylliarobusta* Yabe & Sugiyama, 1936 (288104)** ^CoTW^

*Lobophylli*a robusta cf. Yabe & Sugiyama, 1936 (288104). ^CoTW^ Reported – DMWR 2018; Montgomery et al. 2019.

*Lobophylliarobusta* Yabe & Sugiyama, 1936 (288104). ^CoTW^ Reported – Kenyon et al. 2010; DMWR 2018.

**American Sāmoa status** – Possibly present. **Evidence** – Single specimen report (identified by D Fenner). **Distribution** – Taʻū, Tutuila. **Nearest confirmed ecoregion** – Sāmoa, Tuvalu, and Tonga. **Vulnerability** – LC. **Mesophotic record** – 40 m depth (Montgomery et al. 2019). **Notes** – The specimen identified by D Fenner remains an uncertain identification.

***Lobophylliasinuosa* (Quoy & Gaimard, 1833) (888144)**

*Lobophylliasinosa* (Quoy & Gaimard, 1833) (888144) [sic]. Reported – USACE 1980.

**American Sāmoa status** – Possibly present. **Evidence** – Single report. **Distribution** – Aunuʻu. **Nearest confirmed ecoregion** – Not available. **Notes** – Veron et al. (2019) maintain this species in the genus *Symphyllia* and reports this species as a probable synonym of *S.recta*.

***Lobophylliavalenciennesii* (Milne Edwards & Haime, 1849) (888145)**

*Symphylliavalenciennesii* Milne Edwards & Haime, 1849 (207398) homotypic synonym. Reported – Birkeland et al. 1987. Referenced – Birkeland 2007b.

**American Sāmoa status** – Possibly present. **Evidence** – Multiple reports. **Distribution** – Tutuila. **Nearest confirmed ecoregion** – Fiji. **Geographical range extension** – East. **Notes** – Veron et al. (2019) maintain this species in the genus *Symphyllia*, but also accept the incorrect species spelling of *S.valenciennesi*.

####### Family Merulinidae Verrill, 1865

######## Genus *Coelastrea* Verrill, 1866

***Coelastreaaspera* (Verrill, 1866) (762427)**

*Coelastreaaspera* (Verrill, 1866) (762427). Reported – Montgomery et al. 2019.

*Goniastreaaspera* ? Verrill, 1866 (207467) homotypic synonym. ^CoTW^ Reported – Coles et al. 2003.

*Goniastreaaspera* Verrill, 1866 (207467) homotypic synonym. ^CoTW^ Reported – Fisk and Birkeland 2002; CRED 2011; Corals NPAS 2016. Referenced – DiDonato et al. 2006; Birkeland 2007a; Lovell and McLardy 2008.

**American Sāmoa status** – Possibly present. **Evidence** – Single photographic record. **Distribution** – American Sāmoa, Manuʻa Islands, Ofu, Ofu/Olosega, Taʻū, Tutuila. **Nearest confirmed ecoregion** – Sāmoa, Tuvalu, and Tonga. **Mesophotic record** – 47 m depth (Montgomery et al. 2019).

######## Genus *Cyphastrea* Milne Edwards & Haime, 1848

***Cyphastreadecadia* Moll & Best, 1984 (288920)** ^CoTW^

*Cyphastreadecadia* Moll & Best, 1984 (288920). ^CoTW^ Reported – Kenyon et al. 2010.

**American Sāmoa status** – Possibly present. **Evidence** – Single report. **Distribution** – Rose Atoll. **Nearest confirmed ecoregion** – Sāmoa, Tuvalu, and Tonga. **Vulnerability** – LC. **Notes** – This species is fairly distinctive and was only reported by one source from Rose Atoll where surveys have been limited. Colonies from the lagoon of Rose Atoll found by D Fenner have a colony shape intermediate between that of this species and other species.

***Cyphastreaserailia* (Forskål, 1775) (207413)** ^CoTW^

*Cyphastreaserailia* (Forskål, 1775) (207413). ^CoTW^ Reported – Birkeland et al. 1987, 2003; Maragos et al. 1994; Mundy 1996; Fisk and Birkeland 2002; Coles et al. 2003; Fenner et al. 2008; Kenyon et al. 2010; Corals NPAS 2016. Referenced – Green et al. 1999; Fisk and Birkeland 2002; Coles et al. 2003; DiDonato et al. 2006; Birkeland 2007a, 2007b; Lovell and McLardy 2008.

*Cyphastreaserailia* cf. (Forskål, 1775) (207413). ^CoTW^ Reported – Hunter et al. 1993.

*Cyphastreaseralia* (Forskål, 1775) (207413) [sic]. Reported – Fenner et al. 2008.

*Cyphastreaseralia* ? (Forskål, 1775) (207413) [sic]. Reported – DMWR 2018.

*Cyphastreaserilia* cf. (Forskål, 1775) (207413) [sic]. Reported – Hunter et al. 1993.

**American Sāmoa status** – Possibly present. **Evidence** – Multiple reports. **Distribution** – American Sāmoa, Aunuʻu, Manuʻa Islands, Ofu, Ofu/Olosega, Olosega, Rose Atoll, Taʻū, Tutuila. **Nearest confirmed ecoregion** – Sāmoa, Tuvalu, and Tonga. **Vulnerability** – LC.

######## Genus *Dipsastraea* Blainville, 1830

***Dipsastraeaamicorum* (Milne Edwards & Haime, 1849) (762753)**

*Dipsastraeaamicorum* (Milne Edwards & Haime, 1849) (762753). Reported – Gross 2019.

**American Sāmoa status** – Possibly present. **Evidence** – Single specimen report (unknown identifier). **Distribution** – Tutuila. **Nearest confirmed ecoregion** – Sāmoa, Tuvalu, and Tonga. **Notes** – The record of this species is only based on a single specimen in the University of California of Paleontology (Gross 2019). Veron et al. (2019) classify this species in the genus *Favia*, which is an Atlantic taxon according to others (Budd et al. 2012; Baron-Szabo 2018).

***Dipsastraeahelianthoides* (Wells, 1954) (758239)**

*Faviahelianthila* Wells, 1954 (207429) [sic] homotypic synonym. Reported – USACE 1980.

*Faviahelianthoides* Wells, 1954 (207429) homotypic synonym. ^CoTW^ Reported – Birkeland et al. 1987; Craig et al. 2001; Birkeland 2007a; Corals NPAS 2016. Referenced – DiDonato et al. 2006; Birkeland 2007b; Lovell and McLardy 2008.

*Faviaheliantoides* Wells, 1954 (207429) [sic] homotypic synonym. Reported – Coles et al. 2003.

**American Sāmoa status** – Possibly present. **Evidence** – Multiple reports. **Distribution** – American Sāmoa, Ofu, Tutuila. **Nearest confirmed ecoregion** – Sāmoa, Tuvalu, and Tonga.

***Dipsastraealaddi* (Wells, 1954) (762754)**

*Barabattoialaddi* (Wells, 1954) (271296) homotypic synonym. Reported – Kenyon et al. 2010. Referenced – Kenyon et al. 2011.

**American Sāmoa status** – Possibly present. **Evidence** – Single report. **Distribution** – Rose Atoll. **Nearest confirmed ecoregion** – Solomon Islands and Bougainville and Cook Islands, central Pacific. **Geographical range extension** – Between two disjunct ecoregions. **Notes** – This species is very rarely reported from anywhere in the world and in American Sāmoa only from Rose Atoll during limited surveys. Veron et al. (2019) report the species *B.laddi* as a historical generic designation for *Favialaddi*.

***Dipsastraeamaxima* (Veron, Pichon & Wijsman-Best, 1977) (758230)**

*Faviamaxima* Veron, Pichon & Wijsman-Best, 1977 (207428) homotypic synonym. ^CoTW^ Reported – Kenyon et al. 2010.

**American Sāmoa status** – Possibly present. **Evidence** – Single report. **Distribution** – Rose Atoll. **Nearest confirmed ecoregion** – Sāmoa, Tuvalu, and Tonga. **Notes** – This species has only been recorded from Rose Atoll from limited surveys.

######## Genus *Echinopora* Lamarck, 1816

***Echinoporahorrida* Dana, 1846 (288342)** ^CoTW^

*Echinoporahorrida* Dana, 1846 (288342). ^CoTW^ Reported – Maragos et al. 1994; Mundy 1996. Referenced – Fisk and Birkeland 2002; Birkeland 2007a, 2007b; Lovell and McLardy 2008.

**American Sāmoa status** – Possibly present. **Evidence** – Multiple reports. **Distribution** – American Sāmoa, Manuʻa Islands, Ofu, Olosega, Tutuila. **Nearest confirmed ecoregion** – Sāmoa, Tuvalu, and Tonga. **Vulnerability** – NT.

***Echinoporapacifica* Veron, 1990 (1341443)**

*Echinoporapacifica* Veron, 1990 (1341443). Reported – Fisk and Birkeland 2002.

*Echinoporapacificus* Veron, 1990 (288345) wrong species spelling. ^CoTW^ Reported – Fisk and Birkeland 2002.

**American Sāmoa status** – Possibly present. **Evidence** – Single report. **Distribution** – Manuʻa Islands, Ofu, Tutuila. **Nearest confirmed ecoregion** – Vanuatu. **Geographical range extension** – East. **Vulnerability** – NT. **Notes** – This species was only recorded by a single study and the number of colonies reported was limited (Fisk and Birkeland 2002). Veron et al. (2019) report *Echinoporalamellosa* (Esper, 1795) as a similar species.

######## Genus *Favites* Link, 1807

***Favitescolemani* (Veron, 2000) (763489)**

*Montastraeacolemani* Veron, 2000 (289299) [sic] homotypic synonym. Reported – Birkeland 2007a.

*Montastreacolemani* ? Veron, 2000 (289299) homotypic synonym, wrong genus spelling. Reported – DMWR 2018.

**American Sāmoa status** – Possibly present. **Evidence** – Single specimen report (identified by D Fenner). **Distribution** – Ofu, Taʻū. **Nearest confirmed ecoregion** – Solomon Islands and Bougainville. **Geographical range extension** – East. **Notes** – Veron et al. (2019) accept this species as *Phymastreacolemani* (Veron, 2000). This species is documented by single specimen (DMWR 2018) with an uncertain identification in addition to a single in situ report (Birkeland 2007a).

***Favitescomplanata* (Ehrenberg, 1834) (207455)** ^CoTW^

*Favitescomplanata* (Ehrenberg, 1834) (207455). ^CoTW^ Reported – Birkeland et al. 1987, 2003; Mundy 1996; Craig et al. 2001; Fisk and Birkeland 2002; Coles et al. 2003; DiDonato et al. 2006; Birkeland 2007a; Fenner et al. 2008; CRED 2011; Corals NPAS 2016. Referenced – Fisk and Birkeland 2002; Coles et al. 2003; DiDonato et al. 2006; Birkeland 2007a, 2007b; Lovell and McLardy 2008.

*Favitescomplanata* cf. (Ehrenberg, 1834) (207455). ^CoTW^ Reported – Birkeland et al. 1987; DMWR 2018. Referenced – Green et al. 1999; Coles et al. 2003.

**American Sāmoa status** – Possibly present. **Evidence** – Single photographic record. **Distribution** – American Sāmoa, Aunuʻu, Manuʻa Islands, Ofu, Ofu/Olosega, Taʻū, Tutuila. **Nearest confirmed ecoregion** – Sāmoa, Tuvalu, and Tonga. **Vulnerability** – NT. **Notes** – The record of this species was based on a single specimen with an uncertain identification and the single photographic record (Corals NPAS 2016) appears to be uncertain. There remain multiple reports of this species, so we list this species as possibly present.

***Favitesrotundata* Veron, Pichon & Wijsman-Best, 1977 (207445)**

*Faviarotundata* (Veron, Pichon & Wijsman-Best, 1977) (207427) homotypic synonym. ^CoTW^ Reported – Corals NPAS 2016.

**American Sāmoa status** – Possibly present. **Evidence** – Single photographic record. **Distribution** – American Sāmoa. **Nearest confirmed ecoregion** – Sāmoa, Tuvalu, and Tonga.

***Favitesvalenciennesi* (Milne Edwards & Haime, 1849) (763525)**

*Montastraeavalciennesi* (Milne Edwards & Haime, 1849) (207482) [sic] homotypic synonym. Reported – Fisk and Birkeland 2002.

*Montastraeavalenciennesi* (Milne Edwards & Haime, 1849) (207482) homotypic synonym. Reported – Fisk and Birkeland 2002; CRED 2011; Corals NPAS 2016. Referenced – Fisk and Birkeland 2002; DiDonato et al. 2006; Birkeland 2007a.

*Montastreavalenciennesi* (Milne Edwards & Haime, 1849) (764067) homotypic synonym, wrong genus spelling. Reported – Mundy 1996; Kenyon et al. 2010. Referenced – Lovell and McLardy 2008.

**American Sāmoa status** – Possibly present. **Evidence** – Multiple reports. **Distribution** – American Sāmoa, Manuʻa Islands, Ofu, Ofu/Olosega, Olosega, Taʻū. **Nearest confirmed ecoregion** – Sāmoa, Tuvalu, and Tonga. **Notes** – Veron et al. (2019) accept this species as *Phymastreavalenciennesi* (Milne Edwards & Haime, 1849). This species is very similar to *Favitescolemani* (Veron, 2000), differing in the size of corallites.

**Genus *Hydnophora* Fischer von Waldheim, 1807**

***Hydnophoragrandis* Gardiner, 1904 (287996)**

*Hydnophoragrandis* Gardiner, 1904 (287996). Reported – DMWR 2018.

**American Sāmoa status** – Possibly present. **Evidence** – Single specimen report (identified by D Fenner). **Distribution** – Tutuila. **Nearest confirmed ecoregion** – Not available. **Vulnerability** – LC. **Notes** – Veron et al. (2019) report that *H.grandis* is a synonym of *H.exesa*, but *H.grandis* sensu Veron (2000) is an unnamed species.

######## Genus *Merulina* Ehrenberg, 1834

***Merulinatriangularis* (Veron & Pichon, 1980) (739884)**

*Clavarinatriangularis* Veron & Pichon, 1980 (739878) homotypic synonym. Reported – Birkeland et al. 1987. Referenced – Birkeland 2007b.

*Paraclavarinatriangularis* (Veron & Pichon, 1980) (290627) homotypic synonym. ^CoTW^ Referenced – Lovell and McLardy 2008.

**American Sāmoa status** – Possibly present. **Evidence** – Multiple reports. **Distribution** – American Sāmoa, Aunuʻu, Tutuila. **Nearest confirmed ecoregion** – Fiji. **Geographical range extension** – East.

######## Genus *Oulophyllia* Milne Edwards & Haime, 1848

***Oulophylliabennettae* (Veron, Pichon & Wijsman-Best, 1977) (288394)** ^CoTW^

*Oulophylliabennettae* (Veron, Pichon & Wijsman-Best, 1977) (288394). ^CoTW^ Reported – Fisk and Birkeland 2002; Fenner 2018.

*Oulophylliabennetti* (Veron, Pichon & Wijsman-Best, 1977) (288394) [sic]. Reported – Birkeland 2007a.

**American Sāmoa status** – Possibly present. **Evidence** – Multiple reports. **Distribution** – Manuʻa Islands, Ofu, Ofu/Olosega, Olosega, Taʻū, Tutuila. **Nearest confirmed ecoregion** – Sāmoa, Tuvalu, and Tonga. **Vulnerability** – NT. **Notes** – Veron et al. (2019) report this species as ‘uncommon, but conspicuousʻ. However, this species appears identical to *Oulophylliacrispa* (Lamarck, 1816), except that it has two or fewer corallites in one valley, while *O.crispa* is meandroid with many corallites in a valley. In American Sāmoa, many colonies appear to have intermediate-length valleys.

######## Genus *Paragoniastrea* Huang, Benzoni & Budd, 2014

***Paragoniastreaaustralensis* (Milne Edwards & Haime, 1857) (817177)**

*Goniastrea*australensis (Milne Edwards & Haime, 1857) (207460) homotypic synonym. ^CoTW^ Reported – Mundy 1996; Fisk and Birkeland 2002; Kenyon et al. 2010. Referenced – Fisk and Birkeland 2002; Birkeland 2007a, 2007b; Lovell and McLardy 2008.

*Goniastreaaustraliensis* (Milne Edwards & Haime, 1857) (207460) [sic] homotypic synonym. Reported – Birkeland et al. 1987.

*Goniastreaaustraliensis* ? (Milne Edwards & Haime, 1857) (207460) [sic] homotypic synonym. Reported – DMWR 2018.

**American Sāmoa status** – Possibly present. **Evidence** – Single specimen report (identified by D Fenner). **Distribution** – American Sāmoa, Aunuʻu, Manuʻa Islands, Ofu, Olosega, Taʻū, Tutuila. **Nearest confirmed ecoregion** – Sāmoa, Tuvalu, and Tonga.

######## Genus *Platygyra* Ehrenberg, 1834

***Platygyraryukyuensis* Yabe & Sugiyama, 1935 (289206)** ^CoTW^

*Platygyraryukyuensis* Yabe & Sugiyama, 1935 (289206). ^CoTW^ Reported – Kenyon et al. 2010.

**American Sāmoa status** – Possibly present. **Evidence** – Single report. **Distribution** – Rose Atoll. **Nearest confirmed ecoregion** – Sāmoa, Tuvalu, and Tonga. **Vulnerability** – NT.

####### Family Pocilloporidae Gray, 1840

######## Genus *Pocillopora* Lamarck, 1816

***Pocilloporaacuta* Lamarck, 1816 (759099)** ^CoTW^

*Pocilloporabulbosa* cf. Ehrenberg, 1834 (206968) heterotypic synonym. Reported – USACE 1980; Lamberts 1983.

*Pocilloporadamicornisbulbosa* Ehrenburg, 1834 (224196) heterotypic synonym. Reported – NMNH 2018.

**American Sāmoa status** – Possibly present. **Evidence** – Single specimen report (identifier unknown). **Distribution** – American Sāmoa, Tutuila. **Nearest confirmed ecoregion** – Kiribati, north-east Line Islands. **Geographical range extension** – Southwest although Veron et al. (2019) strongly predicted the presence of this species in the Sāmoa, Tuvalu, and Tonga ecoregion. **Notes** – This species was observed by Lamberts (1983), but within an uncertain identification. A sample exists in the NMNH listed as *Pocilloporadamicornisbulbosa* Ehrenburg, 1834, but this database entry does not list the expert who made the identification. Recent genetic and morphological evidence in Hawaii and Singapore suggests common misidentification between *Pocilloporadamicornis* (Linnaeus, 1758) and *P.acuta* (Poquita-Du et al. 2017, 2019; Johnston et al. 2018).

####### Family Poritidae Gray, 1840

######## Genus *Goniopora* de Blainville, 1830

***Gonioporadjiboutiensis* Vaughan, 1907 (207210)** ^CoTW^

*Gonioporadjiboutiensis* cf. Vaughan, 1907 (207210). ^CoTW^ Reported – Montgomery et al. 2019.

*Gonioporadjiboutiensis* Vaughan, 1907 (207210). ^CoTW^ Reported – Mundy 1996. Referenced – Birkeland 2007b; Lovell and McLardy 2008.

**American Sāmoa status** – Possibly present. **Evidence** – Single photographic record. **Distribution** – American Sāmoa, Tutuila. **Nearest confirmed ecoregion** – Sāmoa, Tuvalu, and Tonga. **Vulnerability** – LC. **Mesophotic record** – 47 m depth (Montgomery et al. 2019).

***Gonioporalobata* Milne Edwards, 1860 (207208)** ^CoTW^

*Gonioporalobata* cf. Milne Edwards, 1860 (207208). ^CoTW^ Reported – Coles et al. 2003.

*Gonioporalobata* Milne Edwards, 1860 (207208). ^CoTW^ Referenced – Birkeland 2007b.

*Gonioporatraceyi* cf. Wells, 1954 (759973) heterotypic synonym. Reported – Lamberts 1983.

**American Sāmoa status** – Possibly present. **Evidence** – Single specimen report (identified by A Lamberts). **Distribution** – Olosega, Tutuila. **Nearest confirmed ecoregion** – Sāmoa, Tuvalu, and Tonga. **Vulnerability** – NT. **Notes** – This species is similar to *Gonioporacolumna* Dana, 1846.

***Gonioporapedunculata* Quoy & Gaimard, 1833 (759975)**

*Gonioporaminor* cf. Crossland, 1952 (207217) heterotypic synonym. ^CoTW^ Reported – Montgomery et al. 2019.

*Gonioporaminor* Crossland, 1952 (207217) heterotypic synonym. ^CoTW^ Reported – Coles et al. 2003; CRED 2011.

**American Sāmoa status** – Possibly present. **Evidence** – Single photographic record. **Distribution** – Ofu/Olosega, Tutuila. **Nearest confirmed ecoregion** – Fiji. **Geographical range extension** – East. **Mesophotic record** – 48 m depth (Montgomery et al. 2019). **Notes** – This species is similar to *Gonioporatenuidens* (Quelch, 1886). Veron et al. (2019) report this as an unrecognized species.

***Gonioporatenuidens* (Quelch, 1886) (207211)** ^CoTW^

*Gonioporatenuidens* (Quelch, 1886) (207211). ^CoTW^ Reported – Birkeland et al. 1987.

*Gonioporatenuidens* cf. (Quelch, 1886) (207211). ^CoTW^ Reported – DMWR 2018.

**American Sāmoa status** – Possibly present. **Evidence** – Single specimen report (identified by D Fenner). **Distribution** – Ofu, Tutuila. **Nearest confirmed ecoregion** – Fiji. **Geographical range extension** – East. **Vulnerability** – LC. **Notes** – This species is similar to *G.pedunculata* (*Gonioporaminor* Crossland, 1952 as a synonym).

######## Genus *Porites* Link, 1807

***Poritesdensa* Vaughan, 1918 (288897)** ^CoTW^

*Poritesdensa* Vaughan, 1918 (288897). ^CoTW^ Reported – Mundy 1996; CRED 2011. Referenced – Fisk and Birkeland 2002; Birkeland 2007a; Lovell and McLardy 2008.

**American Sāmoa status** – Possibly present. **Evidence** – Single report. **Distribution** – American Sāmoa, Manuʻa Islands, Ofu, Rose Atoll, Tutuila. **Nearest confirmed ecoregion** – Solomon Islands and Bougainville. **Geographical range extension** – East. **Vulnerability** – NT. **Notes** – This species has only been reported as a new record by a single study (Mundy 1996). Most *Porites* spp. can be difficult to identify without a skeletal sample.

***Poritesmyrmidonensis* Veron, 1985 (288908)** ^CoTW^

*Poritesmyrmidonensis* Veron, 1985 (288908). ^CoTW^ Reported – Montgomery et al. 2019.

**American Sāmoa status** – Possibly present. **Evidence** – Single photographic record. **Distribution** – Tutuila. **Nearest confirmed ecoregion** – Coral Sea. **Geographical range extension** – East. **Vulnerability** – LC. **Mesophotic record** – 44 m depth (Montgomery et al. 2019).

***Poritesnapopora* Veron, 2000 (288909)** ^CoTW^

*Poritesnapopora* Veron, 2000 (288909). ^CoTW^ Reported – Fisk and Birkeland 2002. Referenced – Birkeland 2007b; Kenyon et al. 2011.

**American Sāmoa status** – Possibly present. **Evidence** – Single report. **Distribution** – Manuʻa Islands, Tutuila. **Nearest confirmed ecoregion** – Pohnpei and Kosrae, Micronesia. **Geographical range extension** – Southeast. **Vulnerability** – 𝒯, VU. **Notes** – This species has only been reported by a single study with only two colonies observed. Veron (2014) reports that this species does not occur within the Sāmoa, Tuvalu, and Tonga region. NOAA (2018) reports that this species is possibly present in American Sāmoa. Fenner (2015) reports this species with a moderate level of species identity while Veron (2014) reports this species is distinctive.

***Poritesnigrescens* Dana, 1848 (207234)** ^CoTW^

*Poritesnigrescens* Dana, 1848 (207234). ^CoTW^ Reported – Maragos et al. 1994, 1995; Mundy 1996; Corals NPAS 2016. Referenced – Fisk and Birkeland 2002; DiDonato et al. 2006; Birkeland 2007a, 2007b; Lovell and McLardy 2008; Kenyon et al. 2011.

**American Sāmoa status** – Possibly present. **Evidence** – Multiple reports. **Distribution** – American Sāmoa, Manuʻa Islands, Ofu, Tutuila. **Nearest confirmed ecoregion** – Sāmoa, Tuvalu, and Tonga. **Vulnerability** – VU.

***Poritespukoensis* Vaughan, 1907 (207250)** ^CoTW^

*Poritespukoensis* Vaughan, 1907 (207250). ^CoTW^ Reported – Hoffmeister 1925; USACE 1980; Lamberts 1983; NMNH 2018. Referenced – Kenyon et al. 2011.

**American Sāmoa status** – Possibly present. **Evidence** – Multiple specimen reports. **Distribution** – American Sāmoa, Rose Atoll, Tutuila. **Nearest confirmed ecoregion** – Hawaii east. **Geographical range extension** – South. **Vulnerability** – CE. **Notes** – Skeleton identification is the strongest evidence of a species presence short of a type locality, but massive *Porites* are difficult to identify. The occurrence of *P.pukoensis* has only been confirmed from the Hawaiian Archipelago despite these reports. However, additional analysis of the specimen within the NMNH collection should occur to make a final conclusion of the presence of species. Veron et al. (2019) show the Sāmoa, Tuvalu, and Tonga region to be doubtful for this species presence.

***Poritessolida* (Forskål, 1775) (207227)** ^CoTW^

*Poritessolida* (Forskål, 1775) (207227). ^CoTW^ Reported – Maragos et al. 1995; Coles et al. 2003; DiDonato et al. 2006; Birkeland 2007a; Forsman et al. 2009; Kenyon et al. 2010; Corals NPAS 2016; DMWR 2018. Referenced – DiDonato et al. 2006; Birkeland 2007a, 2007b; Lovell and McLardy 2008.

*Poritessolida* cf. (Forskål, 1775) (207227). ^CoTW^ Reported – Fisk and Birkeland 2002.

**American Sāmoa status** – Possibly present. **Evidence** – Single specimen report (identified by J Wolstenholme). **Distribution** – American Sāmoa, Aunuʻu, Manuʻa Islands, Ofu, Olosega, Rose Atoll, Taʻū, Tutuila. **Nearest confirmed ecoregion** – Sāmoa, Tuvalu, and Tonga. **Vulnerability** – LC. **Notes** – Large massive *Porites* spp. are the most difficult of all coral species to identify, even with skeleton samples examined by experts. The identify of the photographed specimen of this species in Corals NPAS (2016) appears to be uncertain.

***Poritessuperfusa* Gardiner, 1898 (759336)** ^CoTW^

*Poritessuperfusa* Gardiner, 1898 (759336). ^CoTW^ Reported – Birkeland et al. 2003; Coles et al. 2003; Kenyon et al. 2010; Corals NPAS 2016. Referenced – Green et al. 1999; Coles et al. 2003; DiDonato et al. 2006.

**American Sāmoa status** – Possibly present. **Evidence** – Multiple reports. **Distribution** – Ofu, Rose Atoll, Tutuila. **Nearest confirmed ecoregion** – Sāmoa, Tuvalu, and Tonga.

***Poritesvaughani* Crossland, 1952 (288918)** ^CoTW^

*Poritesvaughani* Crossland, 1952 (288918). ^CoTW^ Reported – Maragos et al. 1994; Fisk and Birkeland 2002; Birkeland et al. 2003; Coles et al. 2003; Fenner et al. 2008; Kenyon et al. 2010; CRED 2011; Corals NPAS 2016. Referenced – Green et al. 1999; Coles et al. 2003; DiDonato et al. 2006; Birkeland 2007a, 2007b; Lovell and McLardy 2008.

**American Sāmoa status** – Possibly present. **Evidence** – Multiple reports. **Distribution** – American Sāmoa, Manuʻa Islands, Ofu, Ofu/Olosega, Olosega, Rose Atoll, Tutuila. **Nearest confirmed ecoregion** – Sāmoa, Tuvalu, and Tonga. **Vulnerability** – LC.

####### Family Psammocoridae Chevalier & Beauvais, 1987

######## Genus *Psammocora* Dana, 1846

***Psammocorahaimiana* Milne Edwards & Haime, 1851 (718603)** ^CoTW^

*Psammacorafolium* Umbgrove, 1939 (207259) [sic] heterotypic synonym. Reported – USACE 1980.

*Psammocorafolium* Umbgrove, 1939 (207259) heterotypic synonym. Reported – Lamberts 1983.

*Psammocorahaimeana* Milne Edwards & Haime, 1851 (207262) wrong species spelling. Reported – Birkeland et al. 1987, 2003; Maragos et al. 1994; Mundy 1996; Fisk and Birkeland 2002; Coles et al. 2003; Birkeland 2007a; Fenner et al. 2008; Kenyon et al. 2010; CRED 2011; Corals NPAS 2016; DMWR 2018; NMNH 2018. Referenced – Green et al. 1999; Fisk and Birkeland 2002; Coles et al. 2003; DiDonato et al. 2006; Birkeland 2007a, 2007b; Lovell and McLardy 2008.

*Psammocorahameana* Milne Edwards & Haime, 1851 (207262) [sic] wrong species spelling. Reported – Fenner et al. 2008.

**American Sāmoa status** – Possibly present. **Evidence** – Single specimen report (identified by A Lamberts). **Distribution** – American Sāmoa, Aunuʻu, Manuʻa Islands, Ofu, Ofu/Olosega, Olosega, Rose Atoll, Swains, Taʻū, Tutuila. **Nearest confirmed ecoregion** – Vanuatu and Tuamotu Archipelago west, central Pacific. **Geographical range extension** – Between two disjunct ecoregions although Veron et al. (2019) strongly predicted the presence of this species in the Sāmoa, Tuvalu, and Tonga ecoregion. **Notes** – Benzoni et al. (2010) discuss at length several species of *Psammocora*. They reported that the species name *P.haimeana* sensu Klunzinger 1879 seems to be an incorrect spelling of *P.haimiana*, but that this misspelling has been propagated in the literature since Veron and Pichon (1976). However, the name *P.haimeana* is not as simple as an incorrect spelling as they document through morphological and molecular analyses that specimens identified as *P.haimeana* are actually *Psammocoraprofundacella* Gardiner, 1898. This indicates that all the colonies listed here as *P.haimeana* are likely *P.profundacella* and should not be considered as evidence for the presence of *P.haimiana*. However, A Lamberts identified a specimen as *P.folium* which appears to be a synonym of *P.haimiana*, which Benzoni et al. (2010) confirm as correct. Despite this sample, we believe that the uncertainty of the identity of this species leaves uncertainty of its presence in American Sāmoa. We should further note that the name *P.haimiana* has not been directly reported in American Sāmoa, which historically has been used incorrectly for the species *P.digitata* (see note under *P.digitata*).

#### Class Hydrozoa Owen, 1843

##### Order Anthoathecata Cornelius, 1992

###### Family Milleporidae Fleming, 1828

####### Genus *Millepora* Linnaeus, 1758

***Milleporatenera* Boschma, 1949 (210729)**

*Milleporatenella* Ortmann, 1892 (287427) heterotypic synonym. Reported – Maragos et al. 1995; Corals NPAS 2016. Referenced – DiDonato et al. 2006; Birkeland 2007b; Lovell and McLardy 2008.

*Milleporatenera* Boschma, 1949 (210729). Reported – USACE 1980; Lamberts 1983; Maragos et al. 1994.

*Milleporatortuosa* Dana, 1848 (735836) heterotypic synonym. Reported – USACE 1980.

*Milliporinatortuosa* Dana, 1848 (735836) [sic] heterotypic synonym. Reported – BPBM 2018.

**American Sāmoa status** – Possibly present. **Evidence** – Single specimen report (identifier unknown). **Distribution** – American Sāmoa, Ofu, Sāmoa Islands, Tutuila. **Nearest confirmed ecoregion** – Not available. **Vulnerability** – LC. **Notes** – This is a branching species and it is possible that *M.dichotoma* in American Sāmoa may have been identified as *M.tenera* erroneously.

### Uncertain

#### Class Anthozoa Ehrenberg, 1834

##### Subclass Hexacorallia Haeckel, 1896

###### Order Scleractinia Bourne, 1900

####### Family Acroporidae Verrill, 1902

######## Genus *Acropora* Oken, 1815

***Acroporacophodactyla* (Brook, 1892) (430641)** ^CoTW^ **taxon inquirendum**

*Acroporacophodactyla /lutkeni* (Brook, 1892) (430641). Reported – DMWR 2018.

*Acroporacophodactyla* (Brook, 1892) (430641). ^CoTW^ Reported – DiDonato et al. 2006; Corals NPAS 2016; DMWR 2018. Referenced – Lovell and McLardy 2008.

*Acroporacophodactyla* aff. (Brook, 1892) (430641). ^CoTW^ Reported – Coles et al. 2003.

**American Sāmoa status** – Uncertain. **Evidence** – Single specimen report (identified by D Fenner). **Distribution** – American Sāmoa, Ofu, Tutuila. **Nearest confirmed ecoregion** – Sāmoa, Tuvalu, and Tonga. **Vulnerability** – DD. **Notes** – *Acroporacophodactyla* has some taxonomic uncertainty, but Veron et al. (2019) recognize this as a valid species. Based on this uncertain taxonomic status, we regard the presence of this species in American Sāmoa as uncertain. However, a number of reports have reported this species or a species similar to *A.cophodactyla*.

***Acroporadendrum* (Bassett-Smith, 1890) (288195)** ^CoTW CCW^

*Acroporadendrum* (Bassett-Smith, 1890) (288195). ^CoTW CCW^ Reported – Fisk and Birkeland 2002.

**American Sāmoa status** – Uncertain. **Evidence** – Single report. **Distribution** – Tutuila. **Nearest confirmed ecoregion** – Vanuatu. **Geographical range extension** – East although Veron et al. (2019) strongly predicted the presence of this species in the Sāmoa, Tuvalu, and Tonga ecoregion. **Vulnerability** – VU. **Notes** – This species has only been reported by Fisk and Birkeland (2002). Wallace (1999) considers this species a difficult and uncertain species rarely reported. Based on this, we have uncertainties about the presence of this species in American Sāmoa.

***Acroporaexquisita* Nemenzo, 1971 (288202)**

*Acroporaexquisita* Nemenzo, 1971 (288202). Reported – Kenyon et al. 2010.

**American Sāmoa status** – Uncertain. **Evidence** – Single report. **Distribution** – Rose Atoll. **Nearest confirmed ecoregion** – Not available. **Vulnerability** – DD. **Notes** – This species is not accepted by Veron et al. (2019), Wallace (1999), or Wallace et al. (2012). Veron et al. (2019) report this species sensu Veron (2000) as unresolved and that it concerns an undescribed species. Based on this uncertainty and only a single observation by Kenyon et al. (2010), we are uncertain of its presence in American Sāmoa.

***Acroporasubulata* (Dana, 1846) (368478)** ^CoTW CCW^

*Acroporasubulata* (Dana, 1846) (368478). ^CoTW CCW^ Reported – Mundy 1996; DiDonato et al. 2006; Corals NPAS 2016; DMWR 2018. Referenced – Fisk and Birkeland 2002; DiDonato et al. 2006; Birkeland 2007b; Lovell and McLardy 2008.

*Acroporasubulata* cf. (Dana, 1846) (368478). ^CoTW CCW^ Reported – Corals NPAS 2016.

**American Sāmoa status** – Uncertain. **Evidence** – Single specimen report (identified by D Fenner). **Distribution** – American Sāmoa, Manuʻa Islands, Tutuila. **Nearest confirmed ecoregion** – Sāmoa, Tuvalu, and Tonga. **Vulnerability** – LC. **Notes** – While a specimen has been identified as *A.subulata* by D Fenner, recent examination and consideration of the previous identification suggests that the specimen may belong to *A.surculosa*.

######## Genus *Astreopora* Blainville, 1830

***Astreoporaexpansa* (Brüggemann, 1877) (207129)** ^CoTW^

*Astreoporaexpansa* (Brüggemann, 1877) (207129). ^CoTW^ Reported – Fisk and Birkeland 2002.

**American Sāmoa status** – Uncertain. **Evidence** – Single report. **Distribution** – Manuʻa Islands, Tutuila. **Nearest confirmed ecoregion** – Vanuatu. **Vulnerability** – NT. **Notes** – Veron et al. (2019) report this species as distinctive, but the species has only been reported by one study. Based on this uncertainty, we believe that its presence in American Sāmoa is uncertain.

######## Genus *Montipora* Blainville, 1830

***Montiporaorientalis* Nemenzo, 1967 (287720)** ^CoTW^

*Montiporaorientalis* ? Nemenzo, 1967 (287720). ^CoTW^ Referenced – Birkeland 2007b.

*Montiporaorientalis* cf. Nemenzo, 1967 (287720). ^CoTW^ Reported – Fisk and Birkeland 2002.

**American Sāmoa status** – Uncertain. **Evidence** – Multiple reports. **Distribution** – Tutuila. **Nearest confirmed ecoregion** – Solomon Islands and Bougainville. **Geographical range extension** – East. **Vulnerability** – VU. **Notes** – This species was reported by two studies both with uncertain identification (Birkeland 2007b, Fisk and Birkeland 2002), indicating that there has been no confirmed observations. Also, this is a rare species (Veron et al. 2019). Based on the lack of a confirmed identification, we consider this species presence as uncertain.

####### Family Agariciidae Gray, 1847

######## Genus *Leptoseris* Milne Edwards & Haime, 1849

***Leptoserisstriata* Fenner & Veron, 2000 (288719)** ^CoTW^

Leptoserisstriata Fenner & Veron, 2000 (288719). ^CoTW^ Reported – Bare et al. 2010.

**American Sāmoa status** – Uncertain. **Evidence** – Single report. **Distribution** – Tutuila. **Nearest confirmed ecoregion** – New Caledonia. **Vulnerability** – NT. **Notes** – Bare et al. (2010) is the only reference to this species and that study has tentative identifications based on video footage taken from a towed camera, which is usually very blurry. Evidence for the presence of this species in American Sāmoa is therefore uncertain.

####### Family Lobophylliidae Dai & Horng, 2009

######## Genus *Homophyllia* Brüggemann, 1877

***Homophylliabowerbanki* (Milne Edwards & Haime, 1857) (886931)**

*Acanthastreahillae* Wells, 1955 (207381) heterotypic synonym. ^CoTW^ Reported – Mundy 1996. Referenced – Fisk and Birkeland 2002; Birkeland 2007a, 2007b; Lovell and McLardy 2008.

**American Sāmoa status** – Uncertain. **Evidence** – Single report. **Distribution** – American Sāmoa, Aunuʻu, Manuʻa Islands, Tutuila. **Nearest confirmed ecoregion** – Sāmoa, Tuvalu, and Tonga. **Notes** – Mundy (1996) reported this species as a new record for American Sāmoa and remarked on its distinctive morphology. Veron et al. (2019) reported that this species may be difficult to distinguish from *Lobophylliaishigakiensis* (Veron, 1990) and *Sclerophylliamaxima* (Sheppard & Salm, 1988), the latter not reported in American Sāmoa. It is possible that this report was a misidentified colony of *L.ishigakiensis*. Veron et al. (2019) maintain *A.hillae* as valid and recognizes *Acanthastreabowerbanki* Milne Edwards & Haime, 1857 as a separate and valid species. Arrigoni et al. (2016) synonymized both species under *H.bowerbanki*.

######## Genus *Oxypora* Saville-Kent, 1871

***Oxyporaglabra* Nemenzo, 1959 (207375)** ^CoTW^

*Oxyporaglabra* Nemenzo, 1959 (207375). ^CoTW^ Reported – Green and Hunter 1998.

**American Sāmoa status** – Uncertain. **Evidence** – Single report. **Distribution** – Tutuila. **Nearest confirmed ecoregion** – Sāmoa, Tuvalu, and Tonga. **Vulnerability** – LC. **Notes** – This species was reported at two sites by Green and Hunter (1999) and no other *Oxypora* spp. were reported. Their observation could have been confused with a different *Oxypora* sp.

####### Family Merulinidae Verrill, 1865

######## Genus *Caulastraea* Dana, 1846

***Caulastraeaechinulata* (Milne Edwards & Haime, 1849) (289576)**

*Caulastreaechinulata /furcata* (Milne Edwards & Haime, 1849) (411159) wrong genus spelling. Reported – Fenner 2018.

**American Sāmoa status** – Uncertain. **Evidence** – Single photographic record. **Distribution** – Tutuila. **Nearest confirmed ecoregion** – Fiji. **Notes** – Veron et al. (2019) maintain the incorrect genus spelling of *Caulastrea*.

######## Genus *Dipsastraea* Blainville, 1830

***Dipsastraeadanai* (Milne Edwards & Haime, 1857) (758238)**

*Faviadanae* ? Verrill, 1872 (764061) homonym, heterotypic synonym. Reported – Coles et al. 2003.

*Faviadanae* Verrill, 1872 (764061) homonym, heterotypic synonym. Reported – CRED 2011.

**American Sāmoa status** – Uncertain. **Evidence** – Single report. **Distribution** – Ofu/Olosega, Rose Atoll, Taʻū, Tutuila. **Nearest confirmed ecoregion** – Sāmoa, Tuvalu, and Tonga. **Notes** – Coles et al. (2003) reported a questionable identification as the only observation of this species in American Sāmoa. Based on this uncertainty, we find the presence of this species uncertain. Veron et al. (2019) maintain *Faviadanai* as the name for this species.

######## Genus *Favites* Link, 1807

***Favitesspinosa* (Klunzinger, 1879) (430662)** ^CoTW^

*Favitesspinosa* cf. (Klunzinger, 1879) (430662). ^CoTW^ Reported – Fisk and Birkeland 2002.

**American Sāmoa status** – Uncertain. **Evidence** – Single report. **Distribution** – Manuʻa Islands, Tutuila. **Nearest confirmed ecoregion** – Bismarck Sea, New Guinea. **Vulnerability** – VU. **Notes** – The identification of this species was listed as cf. by Fisk and Birkeland (2002). Based on the uncertainty of this single observation, we list the presence of this species as uncertain.

######## Genus *Pectinia* Blainville, 1825

***Pectinialactuca* (Pallas, 1766) (207378)** ^CoTW^

*Pectinialactuca* (Pallas, 1766) (207378). ^CoTW^ Reported – Work and Rameyer 2002.

**American Sāmoa status** – Uncertain. **Evidence** – Single report. **Distribution** – American Sāmoa. **Nearest confirmed ecoregion** – Fiji. **Geographical range extension** – East. **Vulnerability** – VU. **Notes** – This species is rather recognizable, but it has not been observed by any other study than by Work and Rameyer (2002), who are not coral experts. Based on this uncertainty, we list this species presence as uncertain.

####### Family Pocilloporidae Gray, 1840

######## Genus *Pocillopora* Lamarck, 1816

***Pocilloporamolokensis* Vaughan, 1907 (411253)** ^CoTW^

*Pocilloporamolokensis* Vaughan, 1907 (411253). ^CoTW^ Reported – Kenyon et al. 2010.

**American Sāmoa status** – Uncertain. **Evidence** – Single report. **Distribution** – Rose Atoll. **Nearest confirmed ecoregion** – Kiribati, north-east Line Islands. **Geographical range extension** – Southwest. **Vulnerability** – DD. **Notes** – *Pocilloporamolokensis* is known as a mesophotic species, and this observation was from shallow water at Rose Atoll. Veron et al. (2019) report a similar species as *Pocilloporaeffusus* Veron, 2000, which occurs in wave-washed habitats that reflect those of Rose Atoll. *Pocilloporaeffusus* is only known from the eastern Pacific (Veron et al. 2019). We conclude that the presence of this species in American Sāmoa is uncertain.

####### Family Poritidae Gray, 1840

######## Genus *Porites* Link, 1807

***Poritesaustraliensis* Vaughan, 1918 (207249)** ^CoTW^

*Poritesaustraliensis* cf. Vaughan, 1918 (207249). ^CoTW^ Reported – Fisk and Birkeland 2002.

*Poritesaustraliensis* Vaughan, 1918 (207249). ^CoTW^ Reported – Maragos et al. 1994, 1995; Coles et al. 2003; DiDonato et al. 2006; Birkeland 2007a; Kenyon et al. 2010; CRED 2011; Corals NPAS 2016; BPBM 2018. Referenced – Coles et al. 2003; DiDonato et al. 2006; Birkeland 2007a, 2007b; Lovell and McLardy 2008.

**American Sāmoa status** – Uncertain. **Evidence** – Single specimen report (identifier unknown). **Distribution** – American Sāmoa, Aunuʻu, Manuʻa Islands, Ofu, Ofu/Olosega, Rose Atoll, Swains, Taʻū, Tutuila. **Nearest confirmed ecoregion** – Sāmoa, Tuvalu, and Tonga. **Vulnerability** – LC. **Notes** – This species is notoriously difficult to identify or separate from *Poriteslobata* Dana, 1846. The single specimen collected is located in the BPBM collection, but the identifying person is not listed within the BPBM database. The specimen in the photograph of this species reported in Corals NPAS (2016) appears to be unidentifiable.

####### Family Psammocoridae Chevalier & Beauvais, 1987

######## Genus *Psammocora* Dana, 1846

***Psammocorastellata* (Verrill, 1866) (287784)** ^CoTW^

*Psammocorastellata* (Verrill, 1866) (287784). ^CoTW^ Reported – CRED 2011.

**American Sāmoa status** – Uncertain. **Evidence** – Single report. **Distribution** – Rose Atoll, Swains. **Nearest confirmed ecoregion** – Kiribati central, Phoenix Islands. **Geographical range extension** – South. **Vulnerability** – VU. **Notes** – The only evidence for this observation came from CRED (2011) during a belt transect survey. More evidence is needed to determine the presence of this species.

#### Class Hydrozoa Owen, 1843

##### Order Anthoathecata Cornelius, 1992

###### Family Stylasteridae Gray, 1847

####### Genus *Distichopora* Lamarck, 1816

***Distichoporagracilis* Dana, 1848 (288326)**

*Distichoporagracilis* Dana, 1848 (288326). Reported – Birkeland et al. 1987; Corals NPAS 2016. Referenced – Coles et al. 2003; DiDonato et al. 2006; Birkeland 2007b; Lovell and McLardy 2008.

**American Sāmoa status** – Uncertain. **Evidence** – Multiple reports. **Distribution** – American Sāmoa, Tutuila. **Nearest confirmed ecoregion** – Not available. **Notes** – Species identifications of corals within *Distichopora* are difficult, so we list this species presence as uncertain. It is possible that reports of *D.gracilis* are of *Distichoporaviolacea* (Pallas, 1766) as they are difficult to distinguish.

***Distichoporaviolacea* (Pallas, 1766) (210734)**

*Distichoporaviolacea* (Pallas, 1766) (210734). Reported – Fenner 2018.

**American Sāmoa status** – Uncertain. **Evidence** – Single photographic record. **Distribution** – Tutuila. **Nearest confirmed ecoregion** – Not available. **Notes** – Species identifications of corals within *Distichopora* are difficult, so we list this species presence as uncertain.

####### Genus *Stylaster* Gray, 1831

***Stylastergracilis* Milne Edwards & Haime, 1850 (285880)**

*Stylastergracilis* cf. Milne Edwards & Haime, 1850 (285880). Reported – Birkeland et al. 1987. Referenced – Green et al. 1999.

*Stylastergracilis* Milne Edwards & Haime, 1850 (285880). Reported – Birkeland et al. 1987; Corals NPAS 2016. Referenced – Coles et al. 2003; DiDonato et al. 2006; Birkeland 2007b; Lovell and McLardy 2008.

**American Sāmoa status** – Uncertain. **Evidence** – Single photographic record. **Distribution** – American Sāmoa, Tutuila. **Nearest confirmed ecoregion** – Not available. **Notes** – Species identifications of corals within *Stylaster* are uncertain, so we list this species as uncertain.

***Stylastersanguineus* Valenciennes in Milne Edwards & Haime, 1850 (285906)**

*Stylasterelegans* Verrill, 1864 (527670) heterotypic synonym. Reported – Maragos et al. 1994; Corals NPAS 2016. Referenced – DiDonato et al. 2006; Birkeland 2007a.

**American Sāmoa status** – Uncertain. **Evidence** – Single photographic record. **Distribution** – Ofu, Ofu/Olosega, Olosega. **Nearest confirmed ecoregion** – Not available. **Notes** – Species identifications of corals within *Stylaster* are uncertain, so we list this species as uncertain.

### Likely not present

#### Class Anthozoa Ehrenberg, 1834

##### Subclass Hexacorallia Haeckel, 1896

###### Order Scleractinia Bourne, 1900

####### Family Acroporidae Verrill, 1902

######## Genus *Acropora* Oken, 1815

***Acroporagrandis* (Brook, 1892) (207031)** ^CoTW CCW^

*Acroporagrandis* (Brook, 1892) (207031). ^CoTW CCW^ Reported – Craig et al. 2001; Corals NPAS 2016. Referenced – DiDonato et al. 2006; Lovell and McLardy 2008.

**American Sāmoa status** – Likely not present. **Evidence** – Multiple reports. **Distribution** – American Sāmoa, Ofu. **Nearest confirmed ecoregion** – Sāmoa, Tuvalu, and Tonga. **Vulnerability** – LC. **Notes** – It is possible that this record concerns a mis-identification of *Acroporaintermedia* (Brook, 1891). Based on this, we consider this species not likely present in American Sāmoa.

***Acroporahemprichii* (Ehrenberg, 1834) (288207)** ^CoTW CCW^

*Acroporahemprichii* cf. (Ehrenberg, 1834) (288207). ^CoTW CCW^ Reported – Fisk and Birkeland 2002.

**American Sāmoa status** – Likely not present. **Evidence** – Single report. **Distribution** – Manuʻa Islands, Ofu. **Nearest confirmed ecoregion** – Sri Lanka south. **Vulnerability** – VU. **Notes** – This species was reported in a single study (Fisk and Birkeland 2002) and was identified as cf., with no photograph or skeleton specimen to support this identification. Based on this single uncertain observation and the fact that this species is only reported from the Red Sea and the Indian Ocean (Wallace 1999; Veron et al. 2019), we believe that it is not likely present in American Sāmoa.

***Acroporahumilis* (Dana, 1846) (207094)** ^CoTW CCW^

*Acroporafructicosa* Brook, 1892 (740120) [sic] heterotypic synonym. Reported – Mayor 1924b; Hoffmeister 1925; Lamberts 1983.

*Acroporafruticosa* Brook, 1892 (740120) heterotypic synonym. Reported – USACE 1980.

*Acroporahumilis* (Dana, 1846) (207094). ^CoTW CCW^ Reported – USACE 1980; Lamberts 1983; Birkeland et al. 1987, 2003, 2013; Itano and Buckley 1988; Hunter et al. 1993; Maragos et al. 1994, 1995; Mundy 1996; Green and Hunter 1998; Craig et al. 2001; Fisk and Birkeland 2002; Coles et al. 2003; Wolstenholme et al. 2003; DiDonato et al. 2006; Birkeland 2007a; Fenner et al. 2008; Bare et al. 2010; Kenyon et al. 2010; CRED 2011; Corals NPAS 2016; AM 2018; BPBM 2018; NMNH 2018; QM 2018. Referenced – Dahl and Lamberts 1977; Dahl 1981; Green et al. 1997, 1999; Fisk and Birkeland 2002; Coles et al. 2003; DiDonato et al. 2006; Birkeland 2007a, 2007b; Lovell and McLardy 2008.

*Acroporahumilis* aff. (Dana, 1846) (207094). ^CoTW CCW^ Reported – Mayor 1924b.

*Acroporaocellata* (Klunzinger, 1879) (207115) heterotypic synonym. ^CoTW^ Reported – Birkeland et al. 1987, 2003; Kenyon et al. 2010; Corals NPAS 2016; Fenner 2018. Referenced – Green et al. 1999; DiDonato et al. 2006; Birkeland 2007b.

*Acroporaocellata* cf. (Klunzinger, 1879) (207115) heterotypic synonym. ^CoTW^ Reported – Coles et al. 2003.

**American Sāmoa status** – Likely not present. **Evidence** – Multiple specimen reports. **Distribution** – American Sāmoa, Aunuʻu, Manuʻa Islands, Ofu, Ofu/Olosega, Olosega, Rose Atoll, Taʻū, Tutuila. **Nearest confirmed ecoregion** – Sāmoa, Tuvalu, and Tonga. **Vulnerability** – NT. **Notes** – This species has often been reported in American Sāmoa (see notes for *A.globiceps*). Based on the historical mis-identification of *A.humilis*, we believe that this species in not likely present in American Sāmoa. The synonym *Acroporaocellata* (Klunzinger, 1879) is recognized by Veron et al. (2019) as a valid species, which therefore needs additional taxonomic study.

***Acroporapharaonis* (Milne Edwards, 1860) (207059)** ^CoTW CCW^

*Acroporapharaonis* (Milne Edwards, 1860) (207059). ^CoTW CCW^ Referenced – Kenyon et al. 2011.

*Acroporapharoensis* (Milne Edwards, 1860) (207059) [sic]. Reported – DMWR 2018.

**American Sāmoa status** – Likely not present. **Evidence** – Single specimen report (identified by D Fenner). **Distribution** – Aunuʻu, Tutuila. **Nearest confirmed ecoregion** – Sri Lanka south. **Vulnerability** – 𝒯, VU. **Notes** – The sample identification was made by D Fenner in the DMWR collection. However, subsequent work has indicated that this identification was incorrect and the sample may represent a new, undescribed species (D Fenner, pers. comm.). Based on this uncertainty, we believe that *A.pharaonis* is likely not present in American Sāmoa.

***Acroporarudis* (Rehberg, 1892) (288241)** ^CoTW CCW^

Acroporarudis (Rehberg, 1892) (288241). ^CoTW CCW^ Reported – DMWR 2018. Referenced – Kenyon et al. 2011.

*Acroporarudis* cf. (Rehberg, 1892) (288241). ^CoTW CCW^ Reported – DMWR 2018.

**American Sāmoa status** – Likely not present. **Evidence** – Single specimen report (identified by D Fenner). **Distribution** – Tutuila. **Nearest confirmed ecoregion** – Sumatra west. **Vulnerability** – 𝒯, EN. **Notes** – The sample identification was made by D Fenner in the DMWR collection, but subsequent work has indicated this identification was incorrect (D Fenner, pers. comm.). Based on this uncertainty, we believe this species is likely not present in American Sāmoa.

######## Genus *Montipora* Blainville, 1830

***Montiporaaustraliensis* Bernard, 1897 (287693)** ^CoTW^

*Montiporaaustraliensis* Bernard, 1897 (287693). ^CoTW^ Reported – Birkeland 2007a. Referenced – Kenyon et al. 2011.

**American Sāmoa status** – Likely not present. **Evidence** – Single report. **Distribution** – Ofu. **Nearest confirmed ecoregion** – Coral Sea and Tuamotu Archipelago south-east and Pitcairn Islands. **Vulnerability** – 𝒯, VU. **Notes** – Veron (2014) reported that this species can be misidentified as other *Montipora* spp. and does not report this as species present within the Sāmoa, Tuvalu, and Tonga region. Fenner (2014) reported this species with a high degree of identification uncertainty, which therefore also applies to American Sāmoa. The only record in American Sāmoa is by Birkeland (2007a). NOAA (2015b) reports the distribution of this species within American Sāmoa as possible, but not confirmed. Based on this information, we conclude that this species is likely absent within American Sāmoa.

***Montiporabilaminata* Bernard, 1897 (1263759)**^CoTW^ taxon inquirendum

*Montiporabilamina* Bernard, 1897 (1263759) [sic]. Reported – Lamberts 1983.

*Montiporabilaminata* Bernard, 1897 (1263759). ^CoTW^ Reported – USACE 1980.

**American Sāmoa status** – Likely not present. **Evidence** – Single specimen report (identified by A Lamberts). **Distribution** – American Sāmoa, Tutuila. **Nearest confirmed ecoregion** – South China Sea.

####### Family Fungiidae Dana, 1846

######## Genus *Polyphyllia* Blainville, 1830

***Polyphylliatalpina* (Lamarck, 1801) (211418)** ^CoTW^

*Polyphylliatalpina* (Lamarck, 1801) (211418). ^CoTW^ Referenced – Lovell and McLardy 2008.

**American Sāmoa status** – Likely not present. **Evidence** – Referenced only. **Distribution** – American Sāmoa. **Nearest confirmed ecoregion** – Sāmoa, Tuvalu, and Tonga. **Vulnerability** – LC. **Notes** – This species has not been directly reported from American Sāmoa and a single study references this species to the United Nations Environment Programme (UNEP) World Conservation Monitoring Centre (WCMC). Based on this limited evidence, we conclude this species presence is likely not present.

####### Family Poritidae Gray, 1840

######## Genus *Porites* Link, 1807

***Poritescompressa* Dana, 1846 (207236)** ^CoTW^

*Poritescompressa* Dana, 1846 (207236). ^CoTW^ Reported – BPBM 2018.

**American Sāmoa status** – Likely not present. **Evidence** – Single specimen report (identifier unknown). **Distribution** – Tutuila. **Nearest confirmed ecoregion** – Hawaii east. **Vulnerability** – LC. **Notes** – The source of this observation is a sample within the BPBM collection, but the identifying person is not listed within the BPBM database. This species could be confused with *Poritescylindrica* Dana, 1846, and *Porites* spp. can be difficult to identify even with a sample in hand. Given *P.compressa* is believed to be restricted to the Hawaiian Islands and that it is similar to a common species, *P.cylindrica*, we believe this species is likely not present in American Sāmoa.

### Not present

#### Class Anthozoa Ehrenberg, 1834

##### Subclass Hexacorallia Haeckel, 1896

###### Order Scleractinia Bourne, 1900

####### Family Acroporidae Verrill, 1902

######## Genus *Acropora* Oken, 1815

***Acroporaplantaginea* (Lamarck, 1816) (207042)**^CoTW^ taxon inquirendum

*Acroporaplantaginea* (Lamarck, 1816) (207042). ^CoTW^ Referenced – Hoffmeister 1925.

**American Sāmoa status** – Not present. **Evidence** – Referenced only. **Distribution** – Sāmoa Islands. **Nearest confirmed ecoregion** – Seychelles south. **Vulnerability** – DD. **Notes** – Veron et al. (2019) accept this species as valid; however, given the taxonomic uncertainty, we consider this species as not present until more information is available.

***Acroporaprolifera* (Lamarck, 1816) (288235)** ^CoTW CCW^

*Acroporaprolifera* (Lamarck, 1816) (288235). ^CoTW CCW^ Reported – Fisk and Birkeland 2002.

**American Sāmoa status** – Not present. **Evidence** – Single report. **Distribution** – Manuʻa Islands, Taʻū. **Nearest confirmed ecoregion** – Belize and west Caribbean. **Notes** – *Acroporaprolifera* is exclusively a Caribbean hybrid. This record may be a simple typo meant to be *Acroporapalifera* (Lamarck, 1816), now known as *Isoporapalifera* (Lamarck, 1816). Given a single reference using this name, we assume it to be an error (verified by C Birkeland, pers. comm.) and hence conclude that it is not present in American Sāmoa.

######## Genus *Alveopora* Blainville, 1830

***Alveoporaexplanata* Hoffmeister, 1945 (1263757)**

*Alveoporaexplanata* Hoffmeister, 1945 (1263757). Reported – Green and Hunter 1998.

**American Sāmoa status** – Not present. **Evidence** – Single report. **Distribution** – Tutuila. **Nearest confirmed ecoregion** – Not available. **Notes** – This species is considered extinct (Hoeksema and Cairns 2018).

####### Family Fungiidae Dana, 1846

######## Genus *Pleuractis* Verrill, 1864

***Pleuractisseychellensis* (Hoeksema, 1993) (716548)**

*Fungiaseychellensis* Hoeksema, 1993 (207345) homotypic synonym. ^CoTW^ Reported – Fenner 2018.

**American Sāmoa status** – Not present. **Evidence** – Single photographic record. **Distribution** – Tutuila. **Nearest confirmed ecoregion** – Chagos Archipelago. **Notes** – This species has only been documented by Fenner (2018) with photographic evidence. However, D Fenner (pers. comm.) reports this identification is incorrect.

####### Family Merulinidae Verrill, 1865

######## Genus *Orbicella* Dana, 1846

***Orbicellaannularis* (Ellis & Solander, 1786) (758260)** ^CoTW^

*Montastraeaannularis* (Ellis & Solander, 1786) (207479) homotypic synonym. Reported – Fisk and Birkeland 2002.

**American Sāmoa status** – Not present. **Evidence** – Single report. **Distribution** – Tutuila. **Nearest confirmed ecoregion** – Belize and west Caribbean. **Vulnerability** – 𝒯. **Notes** – This species is known to be restricted to the Atlantic Ocean and is most likely meant to be *Astreaannuligera* Milne Edwards & Haime, 1849 formerly known as *Montastreaannuligera* (Milne Edwards & Haime, 1849). We assume this to be a simple error as verified by C Birkeland (pers. comm.).

#### Class Hydrozoa Owen, 1843

##### Order Anthoathecata Cornelius, 1992

###### Family Milleporidae Fleming, 1828

####### Genus *Millepora* Linnaeus, 1758

***Milleporaalcicornis* Linnaeus, 1758 (210726)**

*Milleporaalcicornis* Linnaeus, 1758 (210726). Reported – Hoffmeister 1925; Lamberts 1983.

**American Sāmoa status** – Not present. **Evidence** – Multiple specimen reports. **Distribution** – Tutuila. **Nearest confirmed ecoregion** – Not available. **Vulnerability** – LC. **Notes** – Hoffmeister (1925) identified a sample as this species in American Sāmoa. However, this is an Atlantic species that is not been documented to be present anywhere in the Indo-Pacific. The sample was likely *Milleporadichotoma* Forskål, 1775 (see Razak and Hoeksema 2003), which has been documented in American Sāmoa.

### Scleractinia names that are not valid

***Acroporacaniculata* nomen dubium**

*Acroporacaniculata*. Reported – DiDonato et al. 2006; Corals NPAS 2016.

**Closest name** – *Acroporanasuta* (Dana, 1846). **Notes** – The closest name to this report is *Acroporapaniculata*, but this was reported by Corals NPAS (2016) with photographic evidence. Based on this photograph, the colony clearly does not belong to *A.paniculata*. An additional fuzzy match for the species names is *Acroporacanaliculata*, which is a synonym of *Acroporanasuta*. This specimen in the photo more closely matches *A.nasuta*.

**Acroporadamicornisvar.gracilis nomen dubium**

Acroporadamicornisvar.gracilis. Reported – Mayor 1924b.

**Closest name** – *Pocilloporadamicornis* (Linnaeus, 1758). **Notes** – Likely wrong genus name recorded, although the subspecies is unknown.

***Acroporaexigua* (Dana, 1846) (367985) taxon inquirendum**

*Acroporaexigua* (Dana, 1846) (367985). Reported – Hoffmeister 1925; USACE 1980; Lamberts 1983; NMNH 2018.

***Acroporahaimei* Milne Edwards, 1860 (207110) taxon inquirendum**

*Acroporahaimii* Milne Edwards, 1860 (207110) [sic]. Reported – Mayor 1924b.

***Acroporasuperficialis* nomen dubium**

*Acroporasuperficialis*. Reported – Birkeland et al. 1987.

**Closest name** – *Alveoporasuperficialis* Pillai & Scheer, 1976 or *Psammocorasuperficialis* Gardiner, 1898. **Notes** – *Acropora* is very different than *Alveopora* and *Psammocora*, so it seems likely neither of the closest names is correct. The intended identification is not clear, but it is possible the wrong genus name was accidently recorded.

***Cyphastreaimmersa* nomen dubium**

*Cyphastreaimmersa*. Reported – DMWR 2018.

**Closest name** – *Lepastreaimmersa* Klunzinger, 1879. **Notes** – Likely wrong genus name recorded. *L.immersa* is accepted as *Leptastreabottae*.

***Faviachinensis* nomen dubium**

*Faviachinensis*. Reported – Maragos et al. 1995.

**Closest name** – *Faviteschinensis* (Verrill, 1866). **Notes** – Likely wrong genus name recorded.

***Faviafavites* nomen dubium**

*Faviafavites*. Reported – Fisk and Birkeland 2002.

**Notes** – This name was most likely meant to represent an unidentified merulinid species.

***Faviaspinosa* nomen dubium**

*Faviaspinosa* ?. Referenced – Birkeland 2007b.

**Closest name** – *Favitesspinosa* (Klunzinger, 1879). **Notes** – Birkeland (2007b) references Fisk and Birkeland (2002). Likely wrong genus name recorded.

***Gonioporagracilis* (Milne Edwards & Haime, 1849) (207222) taxon inquirendum**

*Gonioporagracilis* cf. (Milne Edwards & Haime, 1849) (207222). Reported – Lamberts 1983.

***Gonioporaparvistella* Ortmann, 1888 (207215) taxon inquirendum**

*Gonioporaparvastella* Ortmann, 1888 (207215) [sic]. Reported – Lamberts 1983.

*Gonioporaparvistella* Ortmann, 1888 (207215). Reported – USACE 1980.

***Gonioporaretiformis* nomen dubium**

*Gonioporaretiformis*. Reported – BPBM 2018.

**Closest name** – *Goniastrearetiformis* (Lamarck, 1816). **Notes** – Likely wrong genus name recorded.

***Gonioporasamoa* nomen dubium**

*Gonioporasamoa*. Reported – Lamberts 1983.

**Closest name** – *Gonioporasomaliensis* Vaughan, 1907. **Notes** – Likely wrong spelling of the species name.

***Herpolithacrassa* nomen dubium**

*Herpolithacrassa*. Reported – USACE 1980; Lamberts 1983.

**Closest name** – *Ctenactiscrassa* (Dana, 1846). **Notes** – Likely wrong genus name recorded.

***Madreporarosacea* Esper, 1791 (1262328) taxon inquirendum**

*Acroporarosacea* Esper, 1791 (1262328). Referenced – Hoffmeister 1925.

**Notes** – Hoffmeister (1925) references Studer (1901).

***Madreporasecunda* Dana, 1846 (815921) taxon inquirendum**

*Acroporasecunda* Dana, 1846 (815921) [sic]. Referenced – Hoffmeister 1925.

**Notes** – Hoffmeister (1925) references Studer (1901).

***Montiporaculiculata* nomen nudum**

*Montiporaculiculata* Bernard (207189). Reported – Birkeland et al. 2003.

***Montiporacurta* nomen dubium**

*Montiporacurta*. Reported – Maragos et al. 1995.

**Closest name** – *Montastreacurta* (Dana, 1846). **Notes** – Likely wrong genus name recorded. *Montastreacurta* is accepted as *Astreacurta*.

***Montiporaelschneri* Vaughan, 1918 (207181) taxon inquirendum**

*Montiporaelschneri* Vaughan, 1918 (207181). Reported – Hoffmeister 1925; USACE 1980; Lamberts 1983; Birkeland et al. 1987, 2003; Green et al. 1997; NMNH 2018. Referenced – Green et al. 1997, 1999; Birkeland 2007b.

*Montiporaelshneri* Vaughan, 1918 (207181) [sic]. Reported – Coles et al. 2003; Corals NPAS 2016. Referenced – Coles et al. 2003; DiDonato et al. 2006.

***Montiporagranulosa* Bernard, 1897 (207171) taxon inquirendum**

*Montiporagranulosa* Bernard, 1897 (207171). Reported – Birkeland et al. 1987, 2003; Green et al. 1997; Birkeland and Belliveau 2000. Referenced – Green et al. 1999; Coles et al. 2003; Birkeland 2007b.

*Montiporagranulosa* cf. Bernard, 1897 (207171). Reported – Birkeland et al. 1987.

**Closest name** – *Montiporagrisea* Bernard, 1897. **Notes** – Likely the wrong species name was recorded.

***Montiporamonticulosa* Studer, 1880 (873507) taxon inquirendum**

*Montiporamonticulosa* Studer, 1880 (873507). Reported – Maragos et al. 1994; Corals NPAS 2016. Referenced – DiDonato et al. 2006; Birkeland 2007a.

**Closest name** – *Montiporamonasteriata* (Forskål, 1775). **Notes** – Likely the wrong species name was recorded.

***Montiporapagoensis* nomen dubium**

*Montiporapagoensis* ?. Reported – Birkeland et al. 2003.

**Closest name** – *Acroporapagoensis* Hoffmeister, 1925. **Notes** – *Acropora* and *Montipora* are quite different, but we suspect the wrong genus name was recorded.

***Montiporascabricula* (Dana, 1846) (759851) taxon inquirendum**

*Montiporascabricula* (Dana, 1846) (759851). Reported – Kenyon et al. 2010.

**Closest name** – *Merulinascabricula* Dana, 1846. **Notes** – Likely wrong genus name recorded.

***Montiporastuderi* Vaughan, 1907 (411237) taxon inquirendum**

*Montiporastuderi* Vaughan, 1907 (411237). Reported – Maragos et al. 1994. Referenced – Birkeland 2007b.

***Montiporatruncata* Zou, Song & Ma, 1975 (1317852) taxon inquirendum**

*Montiporatruncata* Zou, Song & Ma, 1975 (1317852). Referenced – Green et al. 1997.

***Mussasinuosa* (Lamarck, 1816) (1262046) taxon inquirendum**

*Mussasinuosa* (Lamarck, 1816) (1262046). Reported – Hoffmeister 1925; Lamberts 1983.

***Pavonahaimeana* nomen dubium**

*Pavonahaimeana* ?. Reported – Birkeland et al. 2003.

**Closest name** – *Psammocorahaimeana* Milne Edwards & Haime, 1851. **Notes** – Likely wrong genus name recorded.

***Plesiastreacurta* nomen dubium**

*Plesiastreacurta*. Reported – NMNH 2018.

**Closest name** – *Astreacurta* Dana, 1846. **Notes** – *Astrea* was formerly known as *Montastrea*.

***Poritesbernardi* Vaughan, 1907 (869037) taxon inquirendum**

*Poritesbernardi* cf. Vaughan, 1907 (869037). Reported – DMWR 2018.

***Poritesmatthaii* nomen dubium**

*Poritesmatthaii*. Reported – USACE 1980; Lamberts 1983.

**Closest name** – *Faviamatthaii* Vaughan, 1918. **Notes** – Likely wrong genus name recorded.

***Poritesqueenslandiseptima* nomen nudum**

*Poritesqueenslandiseptima*. Reported – USACE 1980; Lamberts 1983.

**Notes** – This name was used by Bernard (1905), who described various varieties of *Porites* colonies using numbers. These varieties are not valid taxa under the rules set forth by the ICZN (B Hoeksema, pers. comm.).

***Psammacora* var . *tutuilensis* nomen dubium**

Psammacoravar.tutuilensis. Reported – USACE 1980.

**Closest name** – Psammocoracontiguavar.tutuilensis Hoffmeister, 1925.

***Scapophylliapistillata* nomen dubium**

*Scapophylliapistillata*. Reported – Green and Hunter 1998.

**Closest name** – *Stylophorapistillata* Esper, 1797. **Notes** – Likely wrong genus name recorded.

***Seriatoporacrassa* Quelch, 1886 (411280) taxon inquirendum**

*Seriatoporacrassa* Quelch, 1886 (411280). Reported – Birkeland et al. 1987. Referenced – Birkeland 2007b.

***Stylasteraurea* nomen dubium**

*Stylasteraurea*. Reported – USACE 1980.

**Closest name** – *Tubastraeaaurea* (Quoy & Gaimard, 1833). **Notes** – *Tubastraea* and *Stylaster* are quite different, but we suspect the wrong genus name was recorded.

***Stylocoracontigua* nomen dubium**

*Stylocoracontigua* ?. Reported – Birkeland et al. 2003.

**Closest name** – *Psammocoracontigua* (Esper, 1794). **Notes** – Likely wrong genus name recorded.

### Phylum Bryozoa

Class Stenolaemata Borg, 1926, Order Cyclostomatida Busk, 1852, Family Lichenoporidae Smitt, 1867, *Domoporatruncata* (Jameson, 1811) (868511) *Milleporatruncata* Jameson, 1811 (1293355) or Class Gymnolaemata Allman, 1856, Order Cheilostomatida Busk, 1852, Family Myriaporidae Gray, 1841, *Myriaporatruncata* (Pallas, 1766) (111435) *Milleporatruncata* Pallas, 1766 (210731) homotypic synonym. Reported – Mayor 1924b, Hoffmeister 1925, Lamberts 1983; **Distribution** – Tutuila; **Notes** – This species was later determined to be a bryozoan, but it is not clear to which species it actually refers. We include this species in the list so one can track the name *M.truncata*.

## Discussion

There can be considerable uncertainty in identifying corals due to several underlying problems (Bernard 1902; Veron 1993, 1995, 2015; Forsman et al. 2015). Some species are inherently difficult to identify or to discern from congeners based on minor morphological differences or plastic characters making taxonomic differences difficult to discern (Todd et al. 2008). Additionally, people may have considerable variation in identification skills and taxonomic knowledge leading to incorrect identifications. Finally, names of species or a species concept can change over time, particularly in groups that have a historical nomenclatural confusion such as *Psammocorahaimeana* as discussed below. This makes it difficult to judge if species identifications are correct without documentation of the observation. For specimens observed in situ, there is no way to truly verify the observation as correct, so we can only rely on the expertise of the observer, the frequency of the species observed, and a subjective likelihood of the species observation. For reports that have photographic documentation, the species observation may be verifiable, but photographic documentation is often inconclusive as the appropriate species level characters are not always visible. Reports that rely on the identification of a collected specimen can be the most powerful for conclusive documentation. However, even with collections, caution should still be exercised because individuals vary in their ability and experience with coral taxonomy and identification. The most conclusive documentation of a species presence is the collection of a specimen that becomes the type specimen of that species. This also includes species and its type that are synonymized with another species. Type specimens described from American Sāmoa include *Acroporatutuilensis* (synonym *Acroporaabrotanoides*), *Acroporapagoensis* (synonym *Acroporaeurystoma*) , *Alveoporaallingi*, *Astreoporacucullata*, *Montiporaberryi*, *Montiporavaughani*, *Pocilloporasetchelli* (synonym *Pocilloporabrevicornis*), *Poriteshorizontalata*, and *Poritesrandalli*.

The spelling of species names and the interpretation of the exact spelling or the assumed name can also be important in determining the likelihood of the presence of that given species. In the course of this analysis, two examples demonstrate the need for keeping track of the spellings and how to determine the meaning of the intended species. One example is the reported species name *Acroporacaniculata* (DiDonato et al. 2006; Corals NPAS 2016) which is not a valid species name. The fuzzy match algorithm used in the WoRMS REST webservice suggested that the best match is *Acroporapaniculata* which is reasonable if one assumes this is a simple typo. However, Corals NPAS (2016) provides photographic evidence of this species and the photo is clearly not *A.paniculata*. Instead, the specimen in the photo more closely resembles *Acroporanasuta*, which has a synonym of *A.canaliculata* that may serve as the likely misspelled name. This highlights the need to carefully review all fuzzy name matches, particularly when a species is reported with no verifiable evidence. Another example is the reported name *Psammocorahaimeana* which had been a historical name used and reported. However, we now know that *P.haimeana* has been a misspelled name of *P.haimiana* starting with Klunzinger (1879) and perpetuated since. Further evidence now shows that colonies reported as *P.haimeana* are actually *Psammocoraprofundacella* (Benzoni et al. 2010). This shows that published names, especially those that do not have type specimens, further complicating proper identification, should be used carefully.

Coral taxonomy and identification are fraught with complex difficulties and highly variable, and sometimes poorly documented characters. Taxonomy is largely based on the morphology of type specimens; therefore, additional comparison between a specimen and the species type description is usually needed to confirm an identification. An example of this situation is *Acroporahumilis* and *Acroporaglobiceps* where we believe all previous reports of *A.humilis* in American Sāmoa are actually *A.globiceps* based on evaluation of the type specimens of both species by D Fenner. These comparisons are rarely done due to researchers being unaware of how to find the type specimens or to the inability to access type specimens, if they are even available at all. Some coral type specimens are of poor quality and increase the likelihood of different interpretations (Veron et al. 2019).

Further complicating coral taxonomy and identification is the uncertain evolutionary history of this group, and the general lack of concordance between morphological and molecular systematics of scleractinians (Fukami et al. 2004). Coral taxonomy is based on morphological characters of the skeleton; however, these characters do not always delineate families or species well. For example, early application of molecular phylogenetic analyses to scleractinian corals revealed two major groups, complex and robust, based on DNA that did not correspond to morphologically-based suborders (Romano and Palumbi 1996; Romano and Cairns 2000). Subsequent studies found that most families of corals based on morphological characters were not monophyletic based on genetic data (Fukami et al. 2008). Adding to the confusion, there is concordance between molecular data and morphological characters in some groups (Wallace et al. 2007; Flot et al. 2008; Forsman et al. 2010; Benzoni et al. 2012b; Marti-Puig et al. 2014; Forsman et al. 2015), but not others and even variable conclusions among studies using the same groups of species (Miller and Benzie 1997; Forsman et al. 2009, 2017; Pinzón et al. 2013; Combosch and Vollmer 2015; Johnston et al. 2017). Veron (1995) proposed this inconsistency originates from corals having undergone reticulate evolution where species populations hybridized during periods of overlap separated by periods of isolation confounding species boundaries. A range of contradictory studies argue in support of or against reticulate evolution in corals (Johnston et al. 2017a; van Oppen et al. 2001; Vollmer and Palumbi 2002; Flot et al. 2011; Combosch and Vollmer 2015). Ultimately, whatever the cause, all morphological traits are based on the underlying genetic code with environmental influences, so morphological and molecular characters have to agree at some level, and there is a need to combine both approaches to further our understanding of species boundaries and resolve the ongoing “species problem” (Bernard 1902) in scleractinian corals (Fukami et al. 2004, 2008; Forsman et al. 2010; Stat et al. 2012; Kitahara et al. 2016; Johnston et al. 2017). Studies that show a discordance between morphological and molecular approaches should be viewed with caution due to the genes examined, false assumptions of the mechanism of evolution, and/or the plasticity and appropriateness of both the morphological and molecular characters examined (Losos et al. 2012). We believe this annotated checklist provides a foundation for further morphological and genetic analysis of the corals present within American Sāmoa.

Despite the difficulties of identification, this checklist is our best estimate of the species we believe are present in American Sāmoa given the caveat of different levels of identification uncertainty. These results provide a comprehensive list of species in an orderly fashion that can be further analyzed and/or reinterpreted by others interested in coral species distribution. We report there has been 745 unique names and spellings of species used for American Sāmoa. Of these, 538 represent valid species names (including synonyms), of which 377 are currently accepted names. Among these 377 species, we conclude that there are 251 species present and 91 species possibly present. In addition, there are 20 species of uncertain presence, nine species likely not present, and six species considered incorrectly reported and not present. A significant factor in determining the number of species in any location is a consistent use of accepted taxonomy.

If we consider differences in the taxonomy, the number of species present in American Sāmoa can change. The main differences between differing taxonomies include two distinct types of synonyms. One includes homotypic synonyms where the species identification is not in dispute, but rather the placement of that species within a certain genus, thereby creating a dispute in species name, but no dispute in that species being a discrete taxonomic entity. Of these species presented here, there are 54 homotypic species with CoTW disagreeing on 40 species. Thirty-four include species where CoTW does not acknowledge the movement of a species to a different genus and six species where CoTW moves species to a different genus but is not currently recognized by WLS. The other type of synonym difference includes heterotypic synonyms where two names are based on two type specimens that have been combined into a single species. In this circumstance, different experts based on their experience and knowledge of that species may have differing opinions on the validity of the synonyms. Of these species presented here, there are 72 heterotypic species with CoTW disagreeing on 20 species split in WLS and eight species that CoTW splits but are not in WLS. A fundamental difference is that taxonomic changes in WLS are based on references in peer-reviewed journals and those in CoTW are not. This is important since taxonomic changes based on the ICZN need to be published in printed media such as journals and books or they need to have a Zoobank registration, while those in electronic media only without a Zoobank registration are not valid. While the on-line version of CoTW (Veron et al. 2019) is not currently compliant with ICZN, it does provide insight to different expert opinion on species concepts.

Here we report new records for American Sāmoa. *Montiporamarshallensis* Wells, 1954 is reported as a probable synonym of *Montiporacrassituberculata* Bernard, 1897 by CoTW and is included in the eight heterotypic synonyms discussed previously, but this synonymization creates the first time this name to be used in relation to American Sāmoa and is not included in the totals listed in this study. Additionally, we report four new records documented by D Fenner not previously reported in American Sāmoa. These records include *Acanthastreasubechinata* Veron, 2000 (Figure 2a), *Favitesparaflexuosus* Veron, 2000 (Figure 2b), *Echinophylliaechinoporoides* Veron & Pichon, 1980 (Figure 2c), and *Turbinariairregularis* Bernard, 1896 (Figure 2d). The evidence presented here is sufficient to conclude these species are present.

We report a total of 342 species present or possibly present for American Sāmoa. If one were to accept the species with taxonomic differences of opinion from Veron et al. (2019), the species number decreases by approximately 12 species. Further, Veron et al. (2019) included only zooxanthellate scleractinian corals thereby reducing the number by another eight species (four azooxanhellate dendrophyllids and four milleporids). This allows comparable numbers of 322 species to the 313 species reported by Veron et al. (2019) from the Sāmoa, Tuvalu, Tonga ecoregion. Presumably, any in-depth analysis for the other islands within this ecoregion will report other species not found in American Sāmoa. This would indicate that the species richness reported in Veron et al. (2019) is likely an underestimate for this ecoregion. It is difficult to determine the amount of this underestimate, but may indicate that the species richness is closer to that found in Micronesia, the Coral Sea, or Vanuatu (Table 1). This also indicates that the species richness is still much lower than the Coral Triangle and the broader geographical pattern of species richness across the Pacific remains (Veron et al. 2015). The known diversity of an area is largely a product of effort. With increasing effort, more species are documented up to an asymptote (Colwell et al. 2012). Both the present list and the estimates in Veron et al. (2019) are likely to be underestimates of the true diversity within any single region.

**Table 2.** The number of corals assessed for extinction risk. The number of corals and the percentages (Unknown (Unk) = DD + NE; Threatened (Thr) = VU + EN + CE).

Species status	DD	LC	NT	VU	EN	CE	NE	Total	Unk	NT + Thr	Thr
Present	5	104	55	41	0	0	46	251	20.3%	38.2%	16.3%
Possibly present	0	32	16	17	1	1	24	91	26.4%	38.5%	20.9%
Uncertain	3	3	2	5	0	0	7	20	50.0%	35.0%	25.0%
Likely not present	0	3	1	3	1	0	1	9	11.1%	55.6%	44.4%
Not present	1	1	0	0	0	0	6	8	87.5%	0.0%	0.0%
Present and possibly present	5	136	72	58	1	1	67	342	21.9%	38.3%	17.5%
MCE	0	47	12	12	1	0	18	90	20.0%	27.8%	14.4%
Global	141	297	176	201	25	5	0	845	16.7%	48.2%	27.3%

Using the ecoregions from Veron et al. (2019), we show geographical range extension records for 66 species considered present or possibly present. The direction of these range extensions are 61% to the east, while 21% close the gap between two disjunct ecoregions. Fewer species are extended south (3%), southeast (11%), and southwest (4%). There are no species that are only extended to the west. Of these range extensions, three species were considerable. The nearest confirmed ecoregion for *Acroporasquarrosa* was Madagascar north, while *Pavonagigantea* was Marshall Islands and Galapagos Islands and *Pavonadiffluens* was Socotra Archipelago in the Indian Ocean.

While shallow coral reef corals are relatively well described, mesophotic corals are poorly described in American Sāmoa. The maximum reported depth of a zooxanthellate coral is 165 m at Johnston Atoll (Kahng and Maragos 2006), and 19 of 66 coral species were reported from mesophotic depths in the Hawaiian Archipelgo (Spalding et al. 2019). Of the 51 reviewed studies that report corals from American Sāmoa, only four have reported corals from mesophotic depths (Hoffmeister 1925; Lamberts 1983; Bare et al. 2010; Montgomery et al. 2019). These limited results report 90 mesophotic species to a maximum depth of 53 m, leaving a significant portion of MCE depths unexplored and hence a large information gap in the coral species diversity. Bare et al. (2010) reported additional species and colonies deeper than previously reported, but the species are tentative identifications from low-resolution video so are not included here. Most of the mesophotic corals reported are considered depth generalist species with almost all of them reported from shallow reefs. Given the maximum depth of these species reports, it is very likely that the number of species recorded from American Sāmoa will increase with more surveys on MCEs, particularly from the lower mesophotic depth range.

Corals have long been threatened from many sources of anthropogenic factors including overfishing, land-based sources of pollution, development, and climate change (Pandolfi 2003; Bellwood et al. 2004; Halpern et al. 2007; Brainard et al. 2013). Based on these threats, 845 scleractinian, helioporid, tubiporid, and milleporid coral species were assessed for extinction risk using the IUCN Red List Categories and Criteria. Carpenter et al. (2008) estimated that 27% of the global species of corals were threatened (Table 2). Of the species considered present or possibly present in American Sāmoa, we estimate that 17.5% of the species are considered threatened while only 14% of the known mesophotic corals are categorized as threatened. This seems in line with the determination that American Sāmoaʻs coral reefs are in “good” condition (NOAA and UM-CEP 2018). Overall, this seems to suggest the corals in American Sāmoa may be doing better than corals on a global scale; however, it should be noted that there is a higher percentage of corals that have an unknown assessment. The vertical distribution of species may play an important role in their potential risk of extinction, particularly for species that are considered depth generalist in which most of the mesophotic corals reported in American Sāmoa are considered.

NOAA has also listed 18 Indo-Pacific species (including three species recorded outside U.S. waters; *Cantharellusnoumeae* Hoeksema & Best, 1984, *Siderastreaglynni* Budd & Guzman, 1994, and *Tubastraeafloreana* Wells, 1982) under the ESA (16 U.S.C. § 1531) and of these species, six have been confirmed in American Sāmoa (*Acroporaglobiceps, Acroporajacquelineae*, *Acroporaretusa*, *Acroporaspeciosa*, *Fimbriaphylliaparadivisa*, and *Isoporacrateriformis*; NOAA 2015a) with another six species (*Acroporalokani*, *Acroporatenella*, *Anacroporaspinosa*, *Montiporaaustraliensis* Bernard, 1897, *Poritesnapopora*, and *Seriatoporaaculeata* considered possibly present (Table 3). In addition, one species, *Pocilloporameandrina*, is a candidate species for listing and it widely reported from American Sāmoa (USACE 1980; Birkeland et al. 1987; Hunter et al. 1993; Maragos et al. 1994, 1995; Mundy 1996; Green and Hunter 1998; Birkeland 2001, 2007a; Craig et al. 2001; Fisk and Birkeland 2002; Work and Rameyer 2002; Birkeland et al. 2003; Coles et al. 2003; DiDonato et al. 2006; Fenner et al. 2008; Kenyon et al. 2010; Corals NPAS 2016; DMWR 2018; Fenner 2018). Of these seven species considered confirmed, we validate that all seven are in American Sāmoa, but also believe two others are possibly present (*Pavonadiffluens* and *Poritesnapopora*). Of the five remaining species (*Acroporalokani*, *Acroporatenella*, *Anacroporaspinosa*, *Montiporaaustraliensis*, and *Seriatoporaaculeata* that NOAA considers possibly present, we believe one, *Montiporaaustraliensis* Bernard, 1897 is likely absent. The four remaining species (*Acroporalokani*, *Acroporatenella*, *Anacroporaspinosa*, and *Seriatoporaaculeata* have not been reported within American Sāmoa. These reports provide resource managers additional information to further evaluate species distributions.

**Table 3.** Corals listed under the ESA. The coral species listed under the ESA with the likely presence reported in this study and NOAA’s report of species occurrence (NOAA 2015b). Listing status is noted 𝒯 for threatened and 𝒞 for candidate. Species reported from mesophotic depths are noted by *.

ESA Species	Listing Status	Presence	NOAA listed Occurrence
*Acroporaglobiceps* (Dana, 1846)	𝒯	Present	Confirmed
*Acroporajacquelineae* Wallace, 1994	𝒯	Present	Confirmed
*Acroporalokani* Wallace, 1994	𝒯	Not reported	Possible
*Acroporapharaonis* (Milne Edwards, 1860)	𝒯	Likely not present	Unlikely
*Acroporaretusa* (Dana, 1846)	𝒯	Present	Confirmed
*Acroporarudis* (Rehberg, 1892)	𝒯	Likely not present	Unlikely
*Acroporaspeciosa (Quelch, 1886)	𝒯	Present	Confirmed
*Acroporatenella* (Brook, 1892)	𝒯	Not reported	Possible
*Anacroporaspinosa* Rehberg, 1892	𝒯	Not reported	Possible
*Fimbriaphylliaparadivisa (Veron, 1990)	𝒯	Present	Confirmed
*Isoporacrateriformis* (Gardiner, 1898)	𝒯	Present	Confirmed
*Montiporaaustraliensis* Bernard, 1897	𝒯	Likely not present	Possible
*Orbicellaannularis* (Ellis & Solander, 1786)	𝒯	Not present	Caribbean species, not in Indo-Pacific
*Pavonadiffluens (Lamarck, 1816)	𝒯	Possibly present	Unlikely
*Pocilloporameandrina* Dana, 1846	𝒞	Present	
*Poritesnapopora* Veron, 2000	𝒯	Possibly present	Possible
*Seriatoporaaculeata* Quelch, 1886	𝒯	Not reported	Possible
